# How Atomic Bonding Plays the Hardness Behavior in the Al–Co–Cr–Cu–Fe–Ni High Entropy Family

**DOI:** 10.1002/smsc.202300225

**Published:** 2023-12-07

**Authors:** Andrea Fantin, Giovanni O. Lepore, Michael Widom, Sergey Kasatikov, Anna M. Manzoni

**Affiliations:** ^1^ Department of Materials Engineering Federal Institute of Materials Research and Testing (BAM) Unter der Eichen 87 12205 Berlin Germany; ^2^ Department of Microstructure and Residual Stress Analysis Helmholtz-Zentrum Berlin Hahn-Meitner-Platz 1 14109 Berlin Germany; ^3^ Department of Earth Science University of Florence Via G. La Pira 4 50121 Firenze Italy; ^4^ Department of Physics Carnegie Mellon University Pittsburgh PA 15217 USA

**Keywords:** hardness, high entropy alloys, local lattice distortions, Monte Carlo molecular dynamics, short-range order

## Abstract

A systematic study on a face‐centered cubic‐based compositionally complex alloy system Al**–**Co**–**Cr**–**Cu**–**Fe**–**Ni in its single‐phase state is carried out, where a mother senary compound Al_8_Co_17_Cr_17_Cu_8_Fe_17_Ni_33_ and five of its suballoys, obtained by removing one element at a time, are investigated and exhaustively analyzed determining the contribution of each alloying element in the solid solution. The senary and the quinaries are compared using experimental techniques including X‐ray absorption spectroscopy, X‐ray diffraction, transmission electron microscopy, and first principles hybrid Monte Carlo/molecular dynamics simulations. Chemical short‐range order and bond length distances have been determined both at the experimental and computational level. Electronic structure and local atomic distortions up to 5.2 Å have been correlated to the microhardness values. A linear regression model connecting hardness with local lattice distortions is presented.

## Graphical abstract

This research on the face‐centered cubic Al–Cr–Fe–Co–Ni–Cu high‐entropy alloy family focuses on a senary and, removing one element at a time, five quinary systems. X‐ray absorption spectroscopy reveals the contribution of each alloying element to the solid solution in terms of short‐range order and local distortion. The latter correlates with microhardness values showing two separate trends, with and without Al.

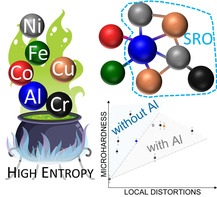

## 1 Introduction

The high‐entropy alloy (HEA) world is barely 20 years old. HEAs have been shown to be solid solutions of mainly simple crystal structures such as face‐centered cubic (fcc), body‐centered cubic (bcc), and hexagonal close packed (hcp).^[^ ^1^ ^]^ Most of the focus has been concentrated so far on the study of mechanical properties^[^ 2, 3, 4 ^]^ from experimental and modeling^[^ ^5^ ^]^ perspectives, also with machine learning algorithms.^[^ ^6^ ^]^ Properties like high resistance to corrosion^[^ 7, 8 ^]^ and irradiation,^[^ ^9^ ^]^ high hardness,^[^ ^10^ ^]^ thermal stability,^[^ ^11^ ^]^ and high‐temperature strength^[^ 11, 12 ^]^ make HEAs interesting for a wide range of industrial applications^[^ ^13^ ^]^ such as in catalysis,^[^ ^14^ ^]^ hydrogen gas or energy storage,^[^ ^15^ ^]^ electromagnetic wave absorption,^[^ ^16^ ^]^ thermoelectric materials,^[^ ^17^ ^]^ radiation protection,^[^ ^18^ ^]^ magnetocaloric materials,^[^ ^19^ ^]^ superconducting materials,^[^ ^20^ ^]^ or shape memory materials.^[^ 21, 22 ^]^

Though macroscopic properties are extensively investigated, studies regarding atomic and electronic rearrangements in solid solutions cover just a small fraction of the whole research on high entropy alloys at the time of writing. Even fewer work is based on experiments, as only cutting‐edge research and nonstandard techniques could provide detailed atomic scale information. Synchrotron radiation^[^ 23, 24, 25 ^]^ has been used for these purposes on few occasions on refractory bcc‐based medium‐ and high‐entropy alloys: correlations have been found between microhardness and pair distribution function peak widths,^[^ ^23^ ^]^ yield strength, and Debye–Waller factors from several techniques,^[^ ^24^ ^]^ and between a tensile tested/as‐prepared specimen and atomic pair distances from extended X‐ray absorption fine structure (EXAFS).^[^ ^25^ ^]^

A Cantor‐related Al*–*Co–Cr–Cu–Fe–Ni system, already widely investigated in literature by coauthors,^[^ 26, 27, 28, 29, 30 ^]^ was chosen for extending the knowledge on the high entropy in terms of lattice distortion and electronic structure effects in fcc systems, and for correlating such knowledge with chemical short‐range order (SRO) information. Individual alloying elements in a senary compositionally complex alloy Al_8_Co_17_Cr_17_Cu_8_Fe_17_Ni_33_ (CCA) were removed one at a time preserving the ratio between alloying elements, so that a systematic study on the CCA and its five fcc‐structured quinary subsystems can be carried out, ideally, by isolating the role of each element in the solid solution. The higher Ni content in the alloys contributes to keep the structure fcc, a concept that was stated in the very early years of high entropy research,^[^ ^31^ ^]^ keeping Al or Cu low avoids the formation of Ni–Al‐rich γ′^[^ ^32^ ^]^ or Cu‐rich^[^ ^32^ ^]^ phases, while Co, Cr, and Fe should also not be too high to prevent the formation of a Co–Cr–Fe rich phase.^[^ ^33^ ^]^ The specific focus of this work is assessing experimentally the contribution of each element to a finite alloy. As electronic interactions between atoms in an alloy are the elemental bricks backing some of the concepts describing HEAs and their unique properties, specific tools are needed to bridge the gap between the unit‐cell level and the macroscopic level. Ad‐hoc experimental methods such as synchrotron‐based X‐ray absorption spectroscopies and conventional X‐ray diffraction (XRD) will be combined and a correlation between atomic, electronic structures and microhardness values in the Al–Co–Cr–Cu–Fe–Ni system will be discussed.

## 2 Experimental Section

### 2.1 Alloy Preparation

All alloys were prepared in a vacuum induction furnace with constituents of 99.99% purity. The cast alloys are the senary alloy Al_8_Co_17_Cr_17_Cu_8_Fe_17_Ni_33_ (at%) or Al_0.5_CoCrCu_0.5_FeNi_2_ (molar denomination), from hereon called “the CCA,” and the quinary suballoys, established by removing from the senary Al (CoCrCu_0.5_FeNi_2,_ from hereon called CCA_sans_Al), Cr (Al_0.5_CoCu_0.5_FeNi_2,_ CCA_sans_Cr), Co (Al_0.5_CrCu_0.5_FeNi_2,_ CCA_sans_Co), Cu (Al_0.5_CoCrFeNi_2,_ CCA_sans_Cu), and Fe (Al_0.5_CoCrCu_0.5_Ni_2,_ CCA_sans_Fe). All ingots underwent the same homogenization treatment of 1250 °C, for 12 h under Ar atmosphere and subsequent quenching in iced water. These conditions were deemed appropriate for obtaining a single phase according to earlier works on the senary compound Al_8_Co_17_Cr_17_Cu_8_Fe_17_Ni_33_ and close compositions.^[^ 26, 29, 30 ^]^

### 2.2 Alloy Precharacterization

#### 2.2.1 Sample Preparation

Specimens for optical microscopy (OM), scanning electron microscopy (SEM), XRD, and hardness tests were mechanically ground and polished down to a final polishing step with a 50 nm sized OP‐U colloidal silica suspension.

#### 2.2.2 Optical Microscopy

OM was performed on a Zeiss Axiophot.

#### 2.2.3 Scanning Electron Microscopy

Samples were analyzed using SEM coupled with energy‐dispersive spectroscopy (EDS) and electron backscatter diffraction (EBSD) on an EVO‐MA15 Zeiss model, equipped with an Oxford ULTIM MAX 40 mm^2^ EDS detector and Oxford Symmetry EBSD detector. Samples were metalized with a few nm of graphite and analyzed at 15 kV acceleration voltage. Details on chemical analysis are reported in **Table** **1** and S1, Supporting Information. EBSD/EDS mappings were performed on a ≈2.8 × 2.0 mm^2^ area with 7 μm step size.

**Table 1:** Sample labels of the CCA_sans_ *X* series (*X* = Ø, Al, Cr, Fe, Co, Cu), composition established by EDS (in at%), microhardness values (HV0.02/20), and lattice parameters refined through the *Fm* $\bar{3}$ *m* space group. In the molar denomination, the “__” placeholder refers to the element removed from the senary composition

Sample name	Molar denomination	Composition (EDS, ±0.2/0.4 at%)						HV [0.02/20]	Lattice [Å]
		**Al**	**Cr**	**Fe**	**Co**	**Ni**	**Cu**		
CCA	Al_0.5_CoCrCu_0.5_FeNi_2_	7.2	16.2	17.0	16.4	34.8	8.5	139(2)	3.588(1)
CCA_sans_Al	__CoCrCu_0.5_FeNi_2_	–	18.1	19.1	19.8	35.0	8.0	119(3)	3.575(1)
CCA_sans_Cr	Al_0.5_Co__Cu_0.5_FeNi_2_	9.2	–	19.7	20.0	38.2	12.9	139(5)	3.587(1)
CCA_sans_Fe	Al_0.5_CoCrCu_0.5___Ni_2_	9.0	19.8	–	20.4	38.9	11.8	198(9)	3.584(1)
CCA_sans_Co	Al_0.5___CrCu_0.5_FeNi_2_	9.2	19.8	20.0	–	38.8	12.2	169(9)	3.604(1)
CCA_sans_Cu	Al_0.5_CoCr__FeNi_2_	7.2	18.3	18.7	20.1	35.8	–	138(4)	3.590(1)

#### 2.2.4 Transmission Electron Microscopy

Samples for transmission electron microscopy (TEM) were mechanically ground to a thickness of 140 μm and then punched out to a disk diameter of 3 mm. Thinning was done by electropolishing the disks in two instruments. The first is a Tenupol 5, using an electrolyte consisting of ethanol, butoxyethanol, and perchloric acid under a voltage of 40 V and at −10 °C. The second is a Tenupol 3 and the electrolyte is a mix of ethanol, glycerol, and perchloric acid, used at −7 °C and 20 V. The TEM used was a JEOL JEM‐2200FS field‐emission TEM, operated at 200 kV. Samples were investigated via bright field (BF), dark field (DF), and selected‐area diffraction (SAD).

#### 2.2.5 X‐ray Diffractiion

XRD was performed in Bragg–Brentano geometry with a Bruker D8 Advance instrument, equipped with a LYNXEYE detector and a nickel filter (0.5 μm). The characteristic radiation lines used were Cu Kα1 (1.5406 Å) and Cu Kα2 (1.5444 Å). Phase identification was carried out with the ICDD PDF2 database in the EVA14 software. Structural refinements were carried out using the software TOPAS.^[^ ^34^ ^]^

#### 2.2.6 Hardness

Hardness data were collected via Vickers microhardness measurements using an Anton Paar MHT‐10 tester in combination with a Reichert–Jung Polyvar Met optical microscope. The Vickers microhardness values (HV) were determined using an indentation force of 0.2 N s^−1^ (20 pond s^−1^).

### 2.3 Simulations Monte Carlo/Molecular Dynamics

First principles hybrid Monte Carlo/molecular dynamics (MC/MD) runs^[^ ^35^ ^]^ were performed at temperature *T* = 1523 K (homogenization temperature) on a 256‐atom 4 × 4 × 4 fcc supercell at the CCA nominal composition Al_8_Co_17_Cr_17_Cu_8_Fe_17_Ni_33_. Spin‐polarized density functional theory calculations utilized the Vienna Ab initio Simulation Package (VASP) code^[^ ^36^ ^]^ with projector‐augmented wave potentials^[^ ^37^ ^]^ in the Perdew–Burke–Ernzerhof (PBE) generalized gradient approximation.^[^ ^38^ ^]^ density functional theory (DFT) calculations utilized a single electronic k‐point and default energy cutoff of 295.4 eV. The simulations alternated 20 fs of molecular dynamics with five attempted Monte Carlo swaps. The data collection run reached a total of 500 attempted Monte Carlo steps and 2 ps of molecular dynamics subsequent to preliminary equilibration of an order of magnitude greater. Eleven independent structures were selected for conventional MD runs at 300 K using the experimentally observed lattice constant.

### 2.4 Synchrotron Data Collection and Analysis

Prior to X‐ray absorption spectroscopy (XAS) measurements, specimen surfaces were accurately polished, and unnecessary oxygen exposure was avoided. Data at the transition metal edges were collected at the LISA CRG beamline (BM‐08^[^ ^39^ ^]^) at the European Synchrotron Radiation Facility (ESRF, Grenoble, France), while data at the Al K‐edge were collected at the PHOENIX beamline (X07MA B^−1^, SLS, Villigen, Switzerland). Standard procedures^[^ ^40^ ^]^ were followed to extract the structural EXAFS signal (*k*·χ(*k*)): pre‐edge background removal, spline modeling of bare atomic background, edge step normalization using a polynomial function interpolated far above the edge region, and energy calibration using the software ATHENA.^[^ ^41^ ^]^ Model atomic clusters centered on the absorber atom were obtained by ATOMS;^[^ ^42^ ^]^ theoretical amplitude and phase functions were generated using the FEFF8 code.^[^ ^43^ ^]^ EXAFS spectra were fitted through the ARTEMIS software^[^ ^41^ ^]^ in the Fourier transform (FT) space.

#### 2.4.1 LISA CRG—ESRF

Samples were measured using a pair of Si (311) flat monochromator crystals; higher harmonics rejection was obtained through Si‐coated collimating/focusing mirrors (*E*_cutoff_ ≈ 15 keV). Data were acquired in total electron yield (TEY) mode and in fluorescence yield mode (FY). TEY mode was acquired using an ad‐hoc apparatus developed by the authors for this purpose, collecting secondary electrons using an anode at a moderate voltage (≈+17 V) in the measurement chamber filled with He gas at 0.8 bar for signal amplification.^[^ ^44^ ^]^ TEY was employed for ascertaining any self‐absorption effect in FY mode. FY was collected simultaneously to TEY with a 13‐channel germanium detector. Data on pure metal foils of Cr, Fe, Co, Ni, and Cu were measured in transmission mode. Spectra were measured at room temperature with a fixed *k* step of 0.05 Å^−1^ up to a maximum *k* value of about *k*_max_ = 12.2 Å^−1^, as intrinsic range limitations were present for Fe, Co, and Ni K‐edges given by the presence of the neighboring *Z* + 1 element (*Z*: atomic number) in the alloy. For Cr and Cu, a maximum data collection value of about *k*_max_ = 12.5 Å^−1^ was taken for consistent comparison of the results obtained for Fe, Co, and Ni elements.

#### 2.4.2 PHOENIX—SLS

The PHOENIX beamline at the Swiss Light Source covers the tender X‐ray range (0.3–8 keV) and offers XAS and scanning microscopy with a beam spot size as low as 2–4 μm. Measurement modes are transmission, TEY, and total fluorescence. The beamline offers two branch lines: the high‐energy branch covers 2–8 keV, and uses a double crystal monochromator, and the low‐energy branch line (0.3–2 keV) uses a planar grating monochromator. The end station of the low‐energy branch is located at the exit of the XTreme beamline.^[^ ^45^ ^]^ The source of the beamline is an elliptical undulator (APPLE II, UE54), providing high flux. At the PHOENIX I beamline at the Swiss Light Source, samples were measured at the 3^rd^ harmonic. Measurements were performed simultaneously in TEY mode and FY mode, once more to ascertain self‐absorption effects. The incident intensity can be measured by taking the TEY signal from a nickel‐coated 0.5 μm thick polyester foil, which is located about 2 m upstream of the sample. Spectra normalization had to be performed onto the broad fluorescence transition metal L‐edges peak, in the case of all CCA measurements, and onto the Cu fluorescence signal (specimen holder) for the X‐ray absorption near‐edge structure (XANES) of pure Al. Data in fluorescence mode were acquired using a single‐channel silicon drift detector (SDD) detector (manufacturer: Ketek) up to *k* = 10.8 Å^−1^

## 3 Results

### 3.1 Precharacterization

Specimens’ details resuming labeling, composition (EDS), microhardness results (HV), and lattice parameters (XRD) are jointly reported in Table 1.

#### 3.1.1 Optical Microscopy

**Figure 1:**
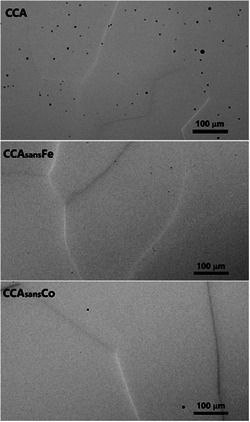
Optical microscopy images for three selected specimens: CCA, CCA_sans_Fe, and CCA_sans_Co.

Selected specimens observed by OM are shown in **Figure** **1**: images of CCA, CCA_sans_Fe, and CCA_sans_ Co are representative of the whole CCA_sans_ *X* series. Grain boundaries and pores (black dots) are visible in every specimen, homogeneous at the 10–100 μm scale.

#### 3.1.2 X‐ray Diffraction

**Figure 2:**
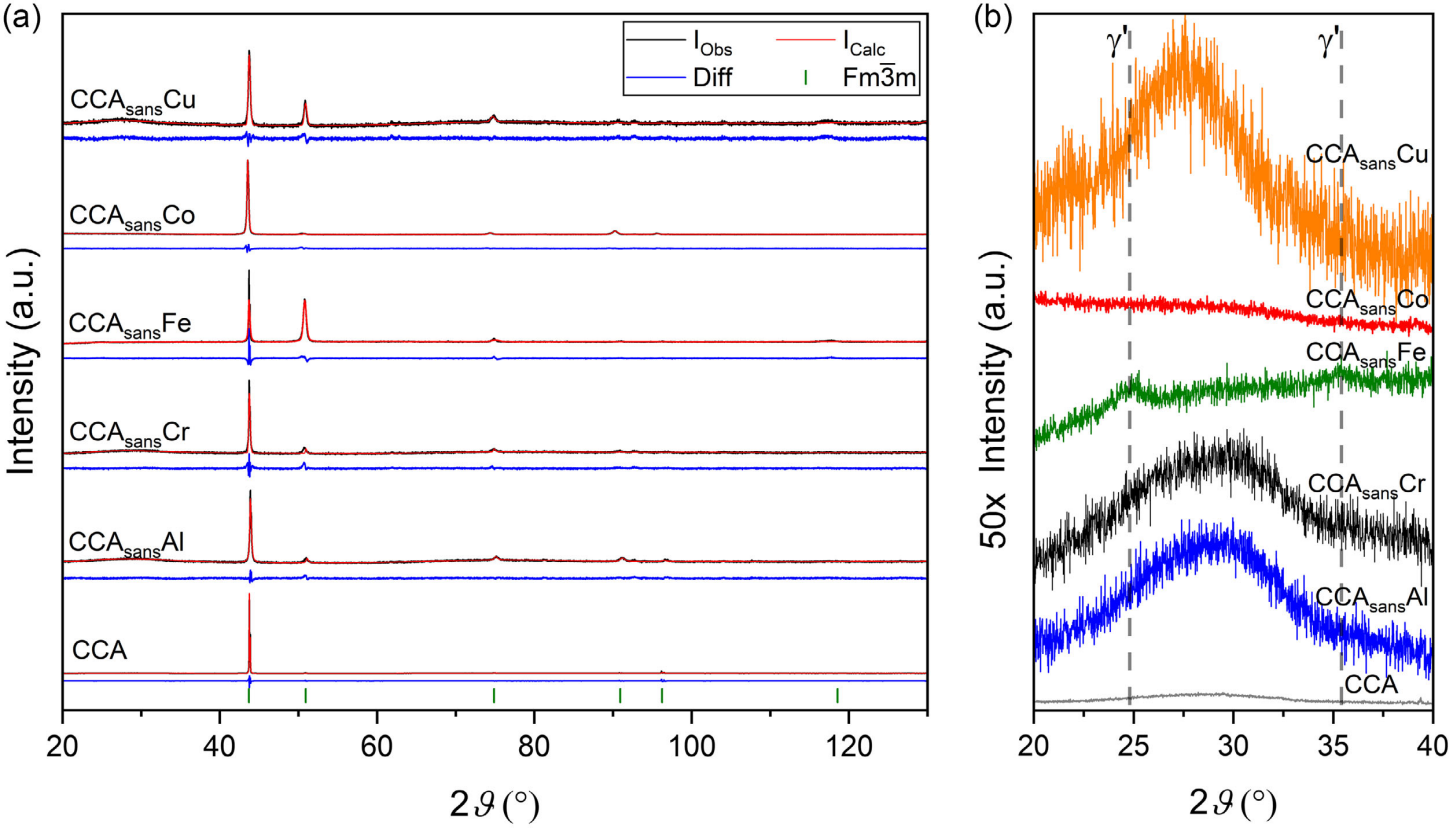
a) Normalized XRD patterns of CCA, CCA_sans_Al, CCA_sans_Cr, CCA_sans_Fe, CCA_sans_Co, and CCA_sans_Cu specimens as a function of the scattering angle 2ϑ (20° ≤ 2ϑ ≤ 130°), from bottom to top, respectively. Observed data (*I*_Obs_, black), calculated data (*I*_Calc_, red), and difference of the two (Diff, blue) are reported for each pattern. Reflection positions according to the space group *Fm* $\bar{3}$ *m*, used for fitting, are also shown at the bottom (green ticks). Peak width is due to the Cu Kα1/Kα2 doublet. b) 50× vertical magnification in the region 20° ≤ 2ϑ ≤ 40°, highlighting γ’ reflections at ≈2ϑ = 25°, 35°.

Conventional XRD was carried out on all the CCA_sans_ *X* (*X* = Ø, Al, Cr, Fe, Co, Cu) series, and patterns with observed, calculated X‐ray intensities and the difference of the two, are depicted in **Figure** **2**.

All alloys are of *Fm* $\bar{3}$ *m* structure, also known as A1 or γ (Figure S1a, Supporting Information). Lattice parameters do not vary much between the alloys, laying mostly between 3.580 Å and 3.590 Å apart from CCA_sans_Al (3.575 Å), the smallest unit cell, and CCA_sans_Co (3.604 Å), the largest one. Preferred orientations are also visible in the different reflection intensity ratios between the different alloys. At first glance, no unindexed peaks are visible in the diffraction patterns, from which it can be concluded that the volume fraction of possible secondary phases is negligible (less than few wt%) at a typical XRD scale (μm). When a magnification of 50× is applied to the low‐2ϑ region (20° ≤ 2ϑ ≤ 40°), small reflections belonging to a γ′ phase (*Pm* $\bar{3}$ *m* s.g., Figure S1b, Supporting Information) appear in CCA_sans_Fe. The broad peak appearing in CCA, CCA_sans_Al, CCA_sans_Cr, and CCA_sans_Cu is background, likely originating from the specimen holder.

#### 3.1.3 Hardness

**Figure 3:**
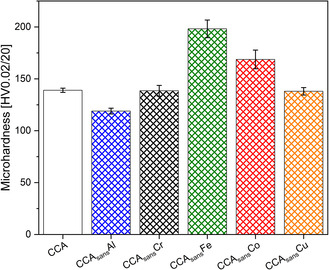
Intrinsic Vickers’ hardness (HV) measured at 0.2 N s^−1^ for the senary and the quinary alloys.

Significant differences were found among the HV values (cf. **Figure** **3**), highlighting how removing specific elements of the alloying composition may have an important effect on alloy strengthening. The highest HV values were found for CCA_sans_Fe and CCA_sans_Co, while CCA_sans_Al has the lowest hardness of the series.

#### 3.1.4 Scanning Electron Microscopy‐Energy‐Dispersive Spectroscopy and Electron Backscatter Diffraction

Table 1 reports the average composition obtained by EDS analysis on each of the studied samples. No significant variations in chemical composition are observed on several point analyses (cf. Table S1, Supporting Information) measured on different grains in each sample. Figure S2, Supporting Information, shows the layered EDS maps measured on each alloying element superimposed on the backscattered electron (BSE) image. EBSD inverse pole figure overlapped with grain boundaries maps are shown in Figure S3, Supporting Information. Both figures highlight the homogeneity at the micrometric scale of all the studied samples in terms of both lattice and chemical composition. The grain size ranges from several hundreds of microns for CCA and CCA_sans_Fe (smallest) and a few millimeters for CCA_sans_Cu and CCA_sans_Al (largest).

#### 3.1.5 Transmission Electron Microscopy

**Figure 4:**
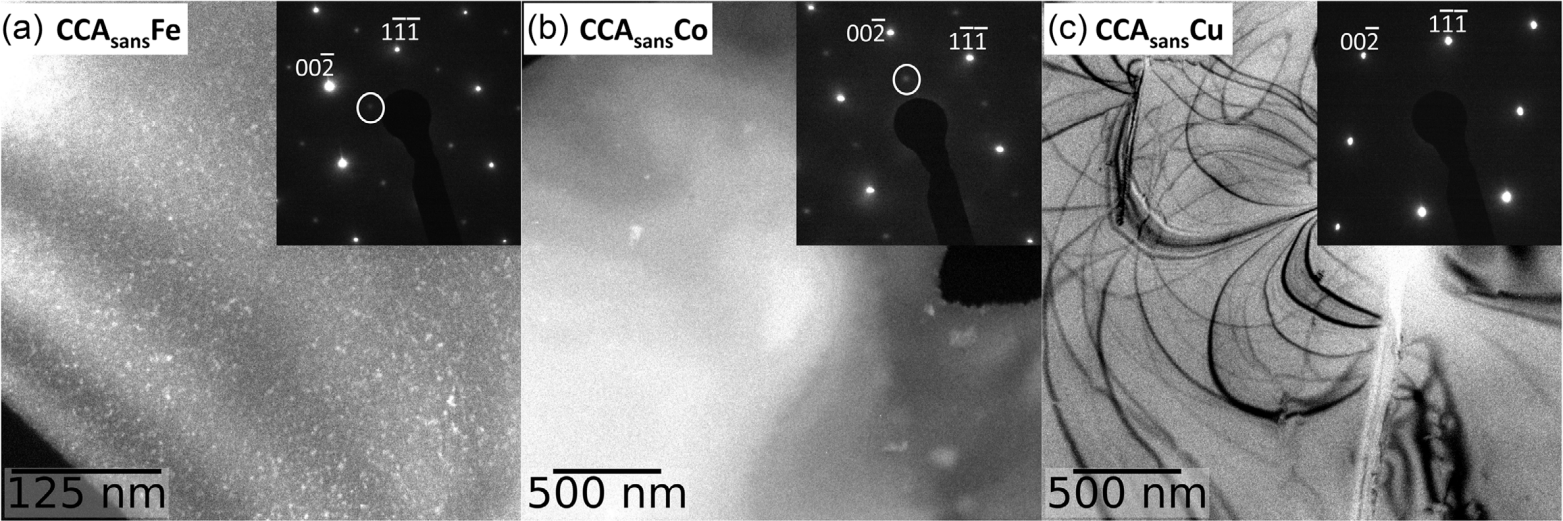
TEM observations of three selected samples: a) DF of CCA_sans_Fe recorded with the $00\bar{1}$ spot (highlighted in a white circle) in the [110] zone axis; b) DF of CCA_sans_Co recorded with the $00\bar{1}$ spot in the [110] zone axis; c) BF of CCA_sans_Cu recorded close to the [110] zone axis. The corresponding SADs are in the insets. Note the different magnifications.

**Figure** **4** shows TEM observations from three selected samples. Similar observations of the CCA have already been shown in a previous article.^[^ ^27^ ^]^ Alloys CCA_sans_Fe (Figure 4a) and CCA_sans_Co (Figure 4b) show a second phase next to γ in their SAD and in the corresponding DF image imaged with the $00\bar{1}$ spot. The additional phase is of L1_2_ structure, also known as γ′, and it has already been detected in the CCA alloy at different heat treatments. The γ′ particles in the CCA_sans_Fe are around 5 nm sized and widely spread, while the particles in CCA_sans_Co are 50–100 nm in size and rather scarce.

CCA_sans_Cu (Figure 4c) shows no additional L1_2_ (nor any other) spots in the SAD, not even via a plot profile unlike the CCA (see Figure 1 in ref. [27]). It can thus be considered completely homogeneous at all scales, which is consistent with other works on this alloy.^[^ 45, 46 ^]^ The corresponding BF image shows only diffraction lines, dislocations, some oxides, and a crack in the sample.

### 3.2 X‐ray Absorption Near‐Edge Structure, Extended X‐ray Absorption Fine Structure, and Simulations

#### 3.2.1 X‐ray Absorption Near‐Edge Structure

As the K‐edge XANES originates from a 1s–*n*p electron transition, it reflects the density of states distribution of the valence electrons’ p‐states of a probed element. By measuring an Al K‐edge XANES spectrum, one can access the empty states of the Al 3p band, which plays a key role in Al chemistry.

**Figure 5:**
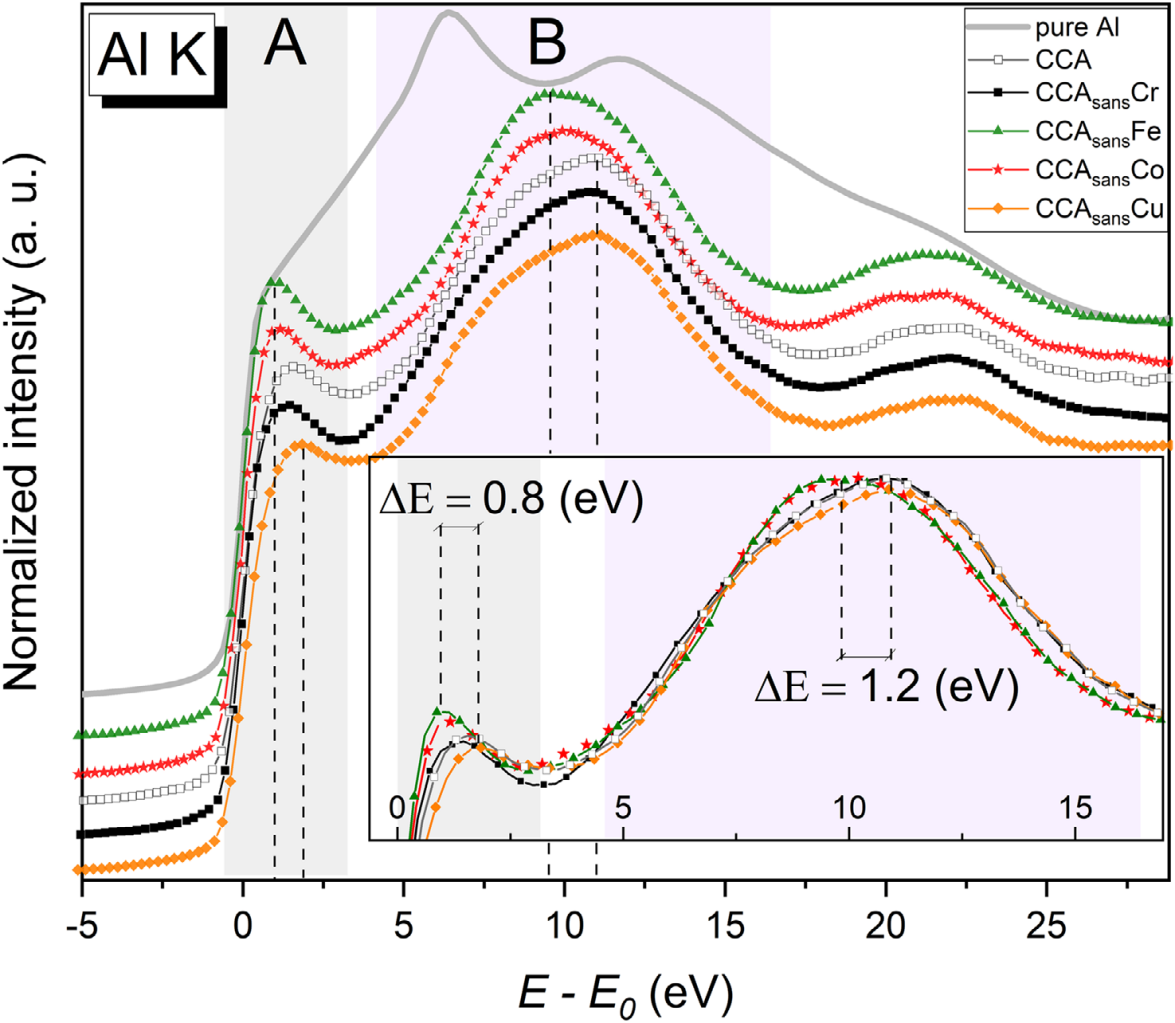
XANES region measured at Al K‐edge for all CCA_sans_ *X* (*X* = Ø, Cr, Fe, Co, Cu). The energy scale is adjusted by the inflection point E_0_ of the edge onset. The pre‐edge and the main line region of the alloys’ spectra are denoted as A and B, respectively. Dashed lines mark the intensity maximum energy position of the mentioned regions with extreme values. In the inset, a magnification of the edge region is presented.

The acquired normalized Al K‐edge spectra of the CCA, CCA_sans_ *X,* and pure Al are presented in **Figure** **5**. The photon energy scale of the spectra is transformed by adjusting the edge onset to zero energy to make it correspond the Fermi level of a specimen. Unlike the pure Al metal spectrum, which is characterized by a broad line with some weak presence of a double‐peak fine structure (5–15 eV), typical of fcc‐structured metals such as Ni or Cu, the alloy spectra appear to have a clear separation in two main regions: a pre‐edge region (denoted A in Figure 5) and a mainline region (B). According to the literature,^[^ ^47^ ^]^ the pre‐edge region can be attributed to empty Al 3p states intermixed with empty 3d TMs states, while the main line region (B) corresponds to empty states with pure p‐character. Given the localized nature of d‐electrons and, on the contrary, itinerant behavior of p‐electrons, the emerging pre‐edge region upon alloying points to increased localization of Al 3p states as a result of alloys formation, and introduction of directional character to metallic bonds.

Based on the energy position and intensity of the A and B regions’ intensity maxima, the alloys can be divided into two groups. The CCA_sans_Fe and CCA_sans_Co spectra appear to have a more pronounced pre‐edge feature, relative to the rest of the alloys. Moreover, the pre‐edge feature and the main line maximum of the CCA_sans_Fe and CCA_sans_Co spectra are shifted closer to the edge onset compared to other alloys. The most significant difference is between the CCA_sans_Fe and CCA_sans_Cu spectra: 0.8 and 1.2 eV for the pre‐edge feature and the main line positions, respectively. Thus, one can conclude a higher localization extent of Al 3p states when Fe or Co are removed from the CCA composition.

#### 3.2.2 Extended X‐ray Absorption Fine Structure

**Figure 6:**
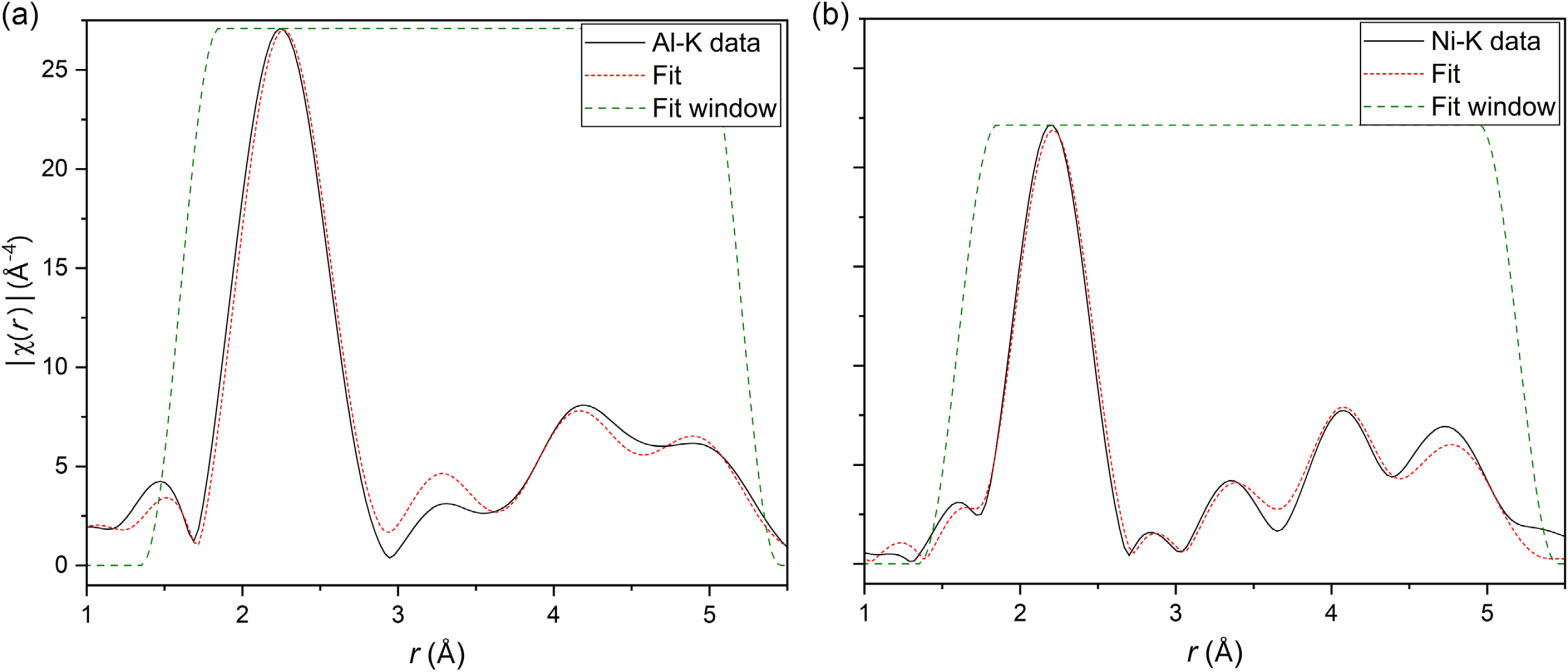
FT |χ(*r*)| in a simultaneous: a) Al K‐edge and b) Ni K‐edge fit (red dotted line) of the CCA data (solid black line) including 4 shells. Fitting windows, using a Hanning function, are shown in green.

The EXAFS fitting model used in the CCA and quinary suballoys is reported in detail elsewhere:^[^ 26, 27 ^]^ it consists of a simultaneous fitting of Al K‐edge and the transition metal K‐edge spectra with a binary model, i.e., using an average 3d metal *M* which accounts for Cr to Cu elements, and an Al atom. This follows the impossibility for EXAFS to discriminate nearest neighbors in the periodic table, as the electron number contrast of all 3d elements between Cr and Cu is too low. Similar arguments apply also to TEM studies.^[^ 48, 49 ^]^ Scattering paths are referred to as central atom‐M/Al (*C*‐M, *C*‐Al), corresponding to the average distance of each absorber *C* to the average transition metal M or to Al, respectively. Compared to previous work^[^ 26, 27 ^]^ where a simple 1^st^ shell approach was used, now an extended 4‐shell model was employed for fitting the data, allowing to obtain information on local distortions up to about 5.2 Å. Several fitting procedures were tested, e.g., assuming a 1^st^ shell single bond length parameter (*dR*) while the 2^nd^, 3^rd^, and 4^th^ shell bond lengths were constrained to a linear expansion. Finally, the fitting procedure giving best results was achieved by employing two parameters for the 1^st^ shell (σ^2^, *dR*), where σ^2^ is a Debye–Waller related factor, a specific *dR* for every other shell (one for *C*‐M and one for *C*‐Al), and a Debye model to account for disorder (dynamic and static) for the 2^nd^, 3^rd^, and 4^th^ shells with one free parameter—the Debye temperature—for the Al (*θ*_D‐Al_) and the 3d absorber (*θ*_D‐M_) paths. A fit example and a results table are presented in **Figure** **6** and **Table** **2**, respectively.

**Table 2:** Fit results example for the CCA at all edges. 1^st^ to 4^th^ shell distances d are reported together with the Debye temperature and Al FNNs. Associated result‐fitting uncertainties are also reported

Sample (edge)	*d* 1^st^ [Å]	Δ1^st^ [Å]	*d* 2^nd^ [Å]	Δ2^nd^ [Å]	*d* 3^rd^ [Å]	Δ3^rd^ [Å]	*d* 4^th^ [Å]	Δ4^th^ [Å]	Θ_D_ [K]	ΔΘ_D_ [K]	Al 1^st^ NN	ΔNN
CCA(Al)	2.541	0.007	3.582	0.034	4.408	0.022	5.134	0.027	397	25	0	0
CCA(Cr)	2.516	0.006	3.537	0.022	4.402	0.015	5.106	0.017	363	16	0	0
CCA(Fe)	2.517	0.005	3.604	0.023	4.409	0.014	5.091	0.016	318	12	1	0.4
CCA(Co)	2.518	0.005	3.601	0.017	4.412	0.011	5.095	0.013	323	9	1	0.3
CCA(Ni)	2.526	0.006	3.599	0.024	4.408	0.016	5.087	0.018	314	12	1.8	0.4
CCA(Cu)	2.542	0.006	3.597	0.022	4.424	0.015	5.100	0.017	324	13	1.6	0.4

**Figure 7:**
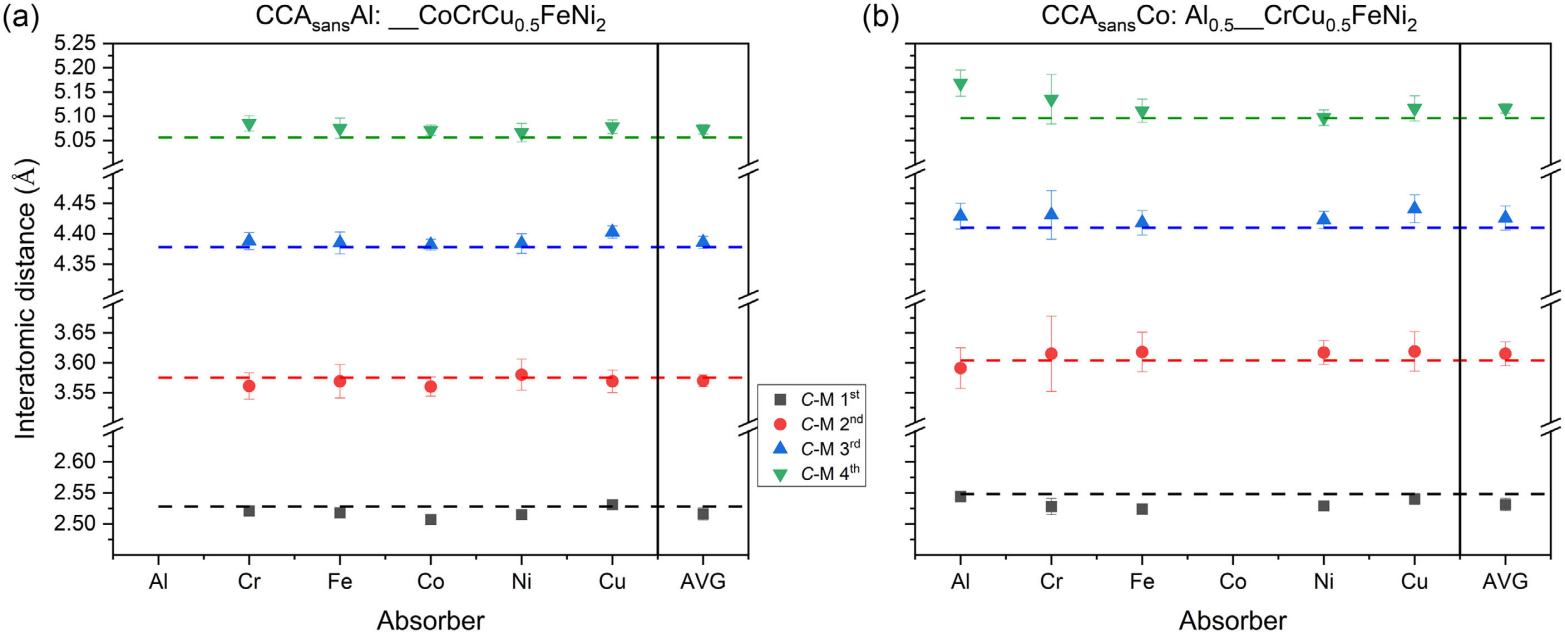
1^st^ to 4^th^ shell refined distances in CCA_sans_ *X* (*X*: Al (a), Co (b)) from XAS data, as a function of the absorber, or central atom C, increasing Z toward the right, together with the corresponding distances obtained by averaging all the EXAFS bond lengths according to the experimentally determined composition (AVG). Dashed lines are added representing the XRD 1^st^ to 4^th^ shell distances for comparison. Results in other CCA_sans_ *X* (*X*: Ø, Cr, Fe, Cu) can be found in Figure S4, Supporting Information.

**Figure** **7** and S4, Supporting Information, focus on the 1^st^ to 4^th^ shell bond distances of each shell retrieved by EXAFS fitting with respect to the long‐range (average) value obtained by conventional XRD (cf. Figure 2 and Table 2). Overall, the crystal structure of the investigated specimens shows relevant differences at different shell levels and by different specimens. Some, such as CCA_sans_Al, do not differ much locally (EXAFS) and on average (XRD), while others such as CCA_sans_Fe or CCA_sans_Co show non‐negligible differences up to the 4^th^ shell included. In Figure 7, two extremes are depicted (CCA_sans_Al, CCA_sans_Co) while the other EXAFS results are shown in Figure S4, Supporting Information.

#### 3.2.3 Simulations

**Figure 8:**
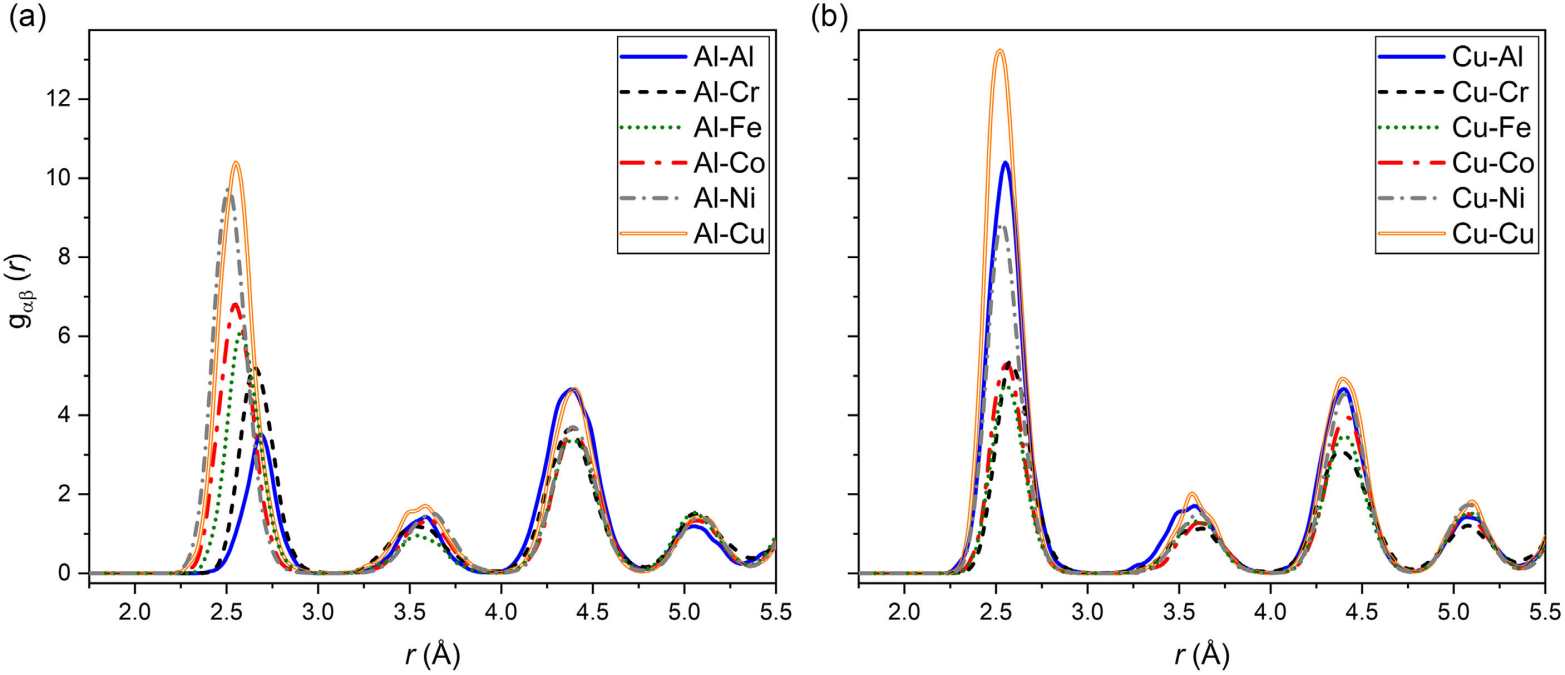
a,b) Simulated Al‐Y (a) and Cu‐Y (b) pair correlation functions as a function of the distance *r* (Å) in the mother compound CCA.

Pair correlation function *g*_αβ_(*r*) of the Al_8_Co_17_Cr_17_Cu_8_Fe_17_Ni_33_ CCA was evaluated for 11 independent structures at 300 K (cf. methods). Results of one run are presented in **Figure** **8** (Al–*Y*, Cu–*Y* pairs) and in Figure S5, Supporting Information (Co, Cr, Fe, Ni–*Y* pairs; *Y* = Al, Cr, Fe, Co, Ni, Cu). The reader is referred to Table S2, Supporting Information, for the calculated averages and standard deviations for the first coordination shell including all 11 runs. Notice the strong Al–Cu peak that reflects the relative unfavorability of Cu–*Y* bonds for each of the transition metal (TM) species, especially Cr, Fe, and Co (Figure 8b). The other notable peak is Al–Ni (Figure 8a), in agreement with their known strong interaction. Warren–Cowley (WC) order parameters provide an alternate measure of correlation. Table S3, Supporting Information, lists WC values for the first coordination shell. Notice that initial values of WC were obtained from a special quasi‐random structure that targeted vanishing WC parameters for three coordination shells, achieving values of 0.03 or less in magnitude in the first shell.

Species swap acceptance rates tell a similar story. The fraction of attempted swaps that are accepted reveals the substitution abilities of pairs of chemical species. The Supporting Information contains a complete list for each species pair (Table S4, Supporting Information). Note that Cu swaps primarily with Al and Ni, satisfying chemical intuition because Cu and Ni both share a filled d‐band, and Cu and Ni are both nearly free electron metals (cf. Bader charges analysis, Figure S6, Supporting Information).

## 4 Discussion

Results show very different elemental influences on the overall behavior on the CCA and its quinary subsystems. The most visible one is the change in homogeneity when Fe or Co are removed from the CCA. Both CCA_sans_Fe and CCA_sans_Co alloys form a second phase, γ′ precipitates, assumed to be the reason of the substantial increase in HV from all other alloys depicted in Figure 3. In CCA_sans_Fe, such precipitates are visible in the TEM (Figure 4a) and in the XRD pattern, denoting a low amount of at least 5 wt%. In the case of the CCA_sans_Co, γ′ phase is visible in TEM (Figure 4b) but not in XRD (Figure 2b), from which it can be assumed a rough upper limit of 5 wt%. Appearance of γ′ might have been prevented by choosing a different heat treatment, which was not possible within the frame of this study and allows thus for ongoing research. Though the composition of γ′ could not be retrieved in the TEM‐EDS, it can be assumed similar to previous studies:^[^ 29, 30 ^]^ an Al–Cu–Ni‐rich composition containing all alloying elements. The presence of γ′ precipitates could be at the origin of the higher energy localization and intensity of the pre‐edge peak observed at the Al K‐edge XANES (Figure 5) for CCA_sans_Co and CCA_sans_Fe, supporting the assumption of an Al‐rich γ′ phase.

**Figure 9:**
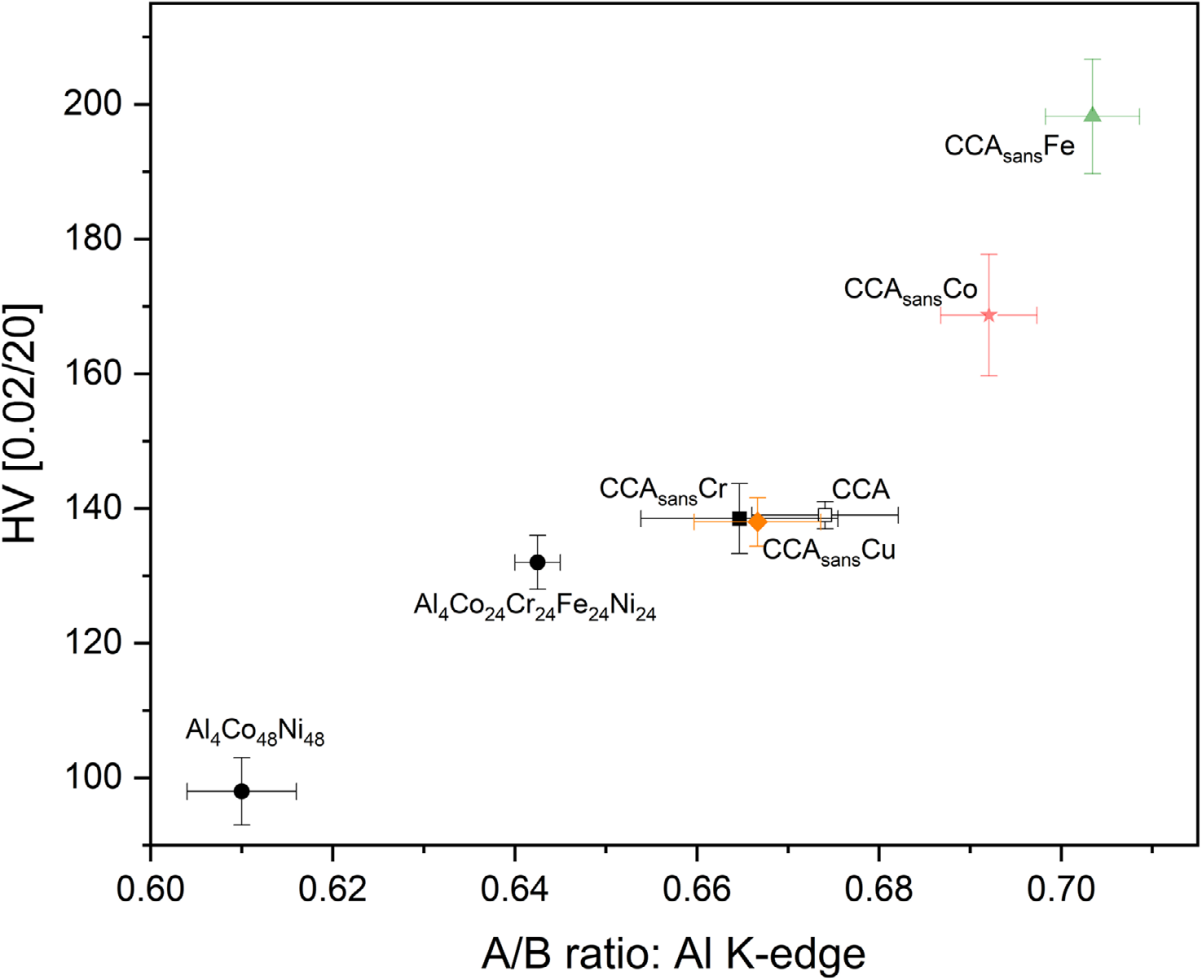
Linear correlation of HV and the A/B features maximum intensity ratio of Al K‐edge spectra within all Al–Ni‐containing alloys.

According to the performed spectra analysis of Figure 5, the pre‐edge feature (A) and the main line (B) of the alloy Al K‐edge spectra can be considered as indicators for the extent of Al 3p band localization/delocalization. In turn, a variation in localization of the Al valence states points to changes in bond directionality that can be crucial for mechanical properties.^[^ 50, 51, 52 ^]^ To make a tentative assessment of Al 3p band localization and Al–TMs bonds directionality, one can consider a maximum intensity ratio of the A to B features, high values of which imply an increased interaction of Al 3p and TMs 3d electronic states, and, hence, higher Al 3p band energy localization and Al–TMs bonds directionality and vice versa. **Figure** **9** shows the HV values as a function of the A/B ratio measured for the CCA and CCA_sans_ *X* alloys and additional Al–Ni‐containing alloys: Al_4_Co_48_Ni_48_, Al_4_Cr_24_Co_24_Fe_24_Ni_24_ (in at%) (cf. Figure S7, Supporting Information, for the additional alloys spectra).

**Figure 10:**
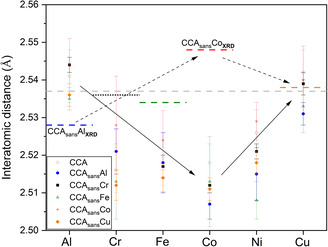
EXAFS 1^st^ shell results magnification together with XRD data. Lines are guided to the eye for the opposite trends of XRD (dashed) and XAS (full).

Negligible differences in measured EXAFS spectra and subsequent fit outcomes in γ′‐containing and γ′‐free specimens indicate the substantial local similarity and/or the relevant disproportion in quantity between the γ and the γ′. Still, the presence of γ′ in CCA_sans_Fe and CCA_sans_Co must be considered in the EXAFS discussion on the solid solution; in all subsequent figures, CCA_sans_Fe and CCA_sans_Co data points will be shown with a 50% transparency. **Figure** **10** highlights the 1^st^ shell comparison between EXAFS and XRD results. The *V*‐shaped behavior of EXAFS distances versus Z number, already seen in Ref. [26] for the mother compound CCA, is observed for the whole CCA_sans_ *X* family and guided by a full arrow.

It is interesting to observe the distribution of bond lengths for each absorbing element. Al, which has an overall good affinity with any other alloying element except itself (cf. Table S3, S4, Supporting Information, and Figure 8), shows the lowest spread in bond length distribution. The large *C*‐Al (*C*: central atom) bond length derives from the larger Al metallic radius (*r*_Al_ = 1.43 Å) although, as also shown in Ref. [26], a reduction by approximately 0.17 Å is observed when Al is surrounded by 3d elements.

Co–*M*, Fe–*M*, and Cu–*M* pairs also have limited variation in bond lengths. Fe and Co tend not to dislike bonding with any other alloying element except Cu being the two most compatible elements in the system. In contrast, Cu prefers Al and itself (cf. Figure 8) meaning that Cu on average interacts less and has a strong nearest neighbor preference, as shown previously for the CCA.^[^ 26, 27, 28 ^]^

The comparison between average interatomic distances, as derived from the unit‐cell parameters determined by XRD (Figure 2), and the local structural effects due to the removal of specific atoms, provides an interesting perspective. Al–*M* has the largest bonding distances (together with Cu–*M*) and a narrow distribution for the lowest mixing enthalpy with all elements (cf. Table S5, Supporting Information). The removal of Al from the system (CCA_sans_Al) decreases the unit cell size and therefore the average 1^st^ shell distances based on XRD data (blue‐dashed line) compared to the CCA (dashed gray line). Removal of Co (CCA_sans_Co) instead, increases the unit cell size, as Co–*M* are the shortest bond lengths, increasing also the 1^st^ shell XRD distances (red‐dashed line) compared to CCA (dashed gray line). In between Al–*M* and Co–*M*, with increasing *Z* number, such behavior can be interpolated naturally on both XRD and XAS level, i.e., the V‐shaped bond length distribution retrieved from EXAFS is, in first approximation, opposed by an inversed V shape of the unit cell distribution found by XRD. Cu–*M* distances are explained by the fact that Cu is the least interacting element in the alloy according to formation enthalpy (cf. Table S5, Supporting Information) and to the fact that in Cu–*M*, there is a non‐negligible number of Cu–Cu pairs. The preference for Cu–Cu pairing would also explain the large Cu–*M* bond lengths, as Cu has the largest 3d nominal radius, *r*_Cu_ = 1.28 Å. Finally, the Cr–*M* and the Ni–*M* ones have the most dispersed bond length distributions, implying that their role changes most when removing one (any) element from the mother compound CCA, possibly driven by specific interactions such as SRO.^[^ ^28^ ^]^

**Figure 11:**
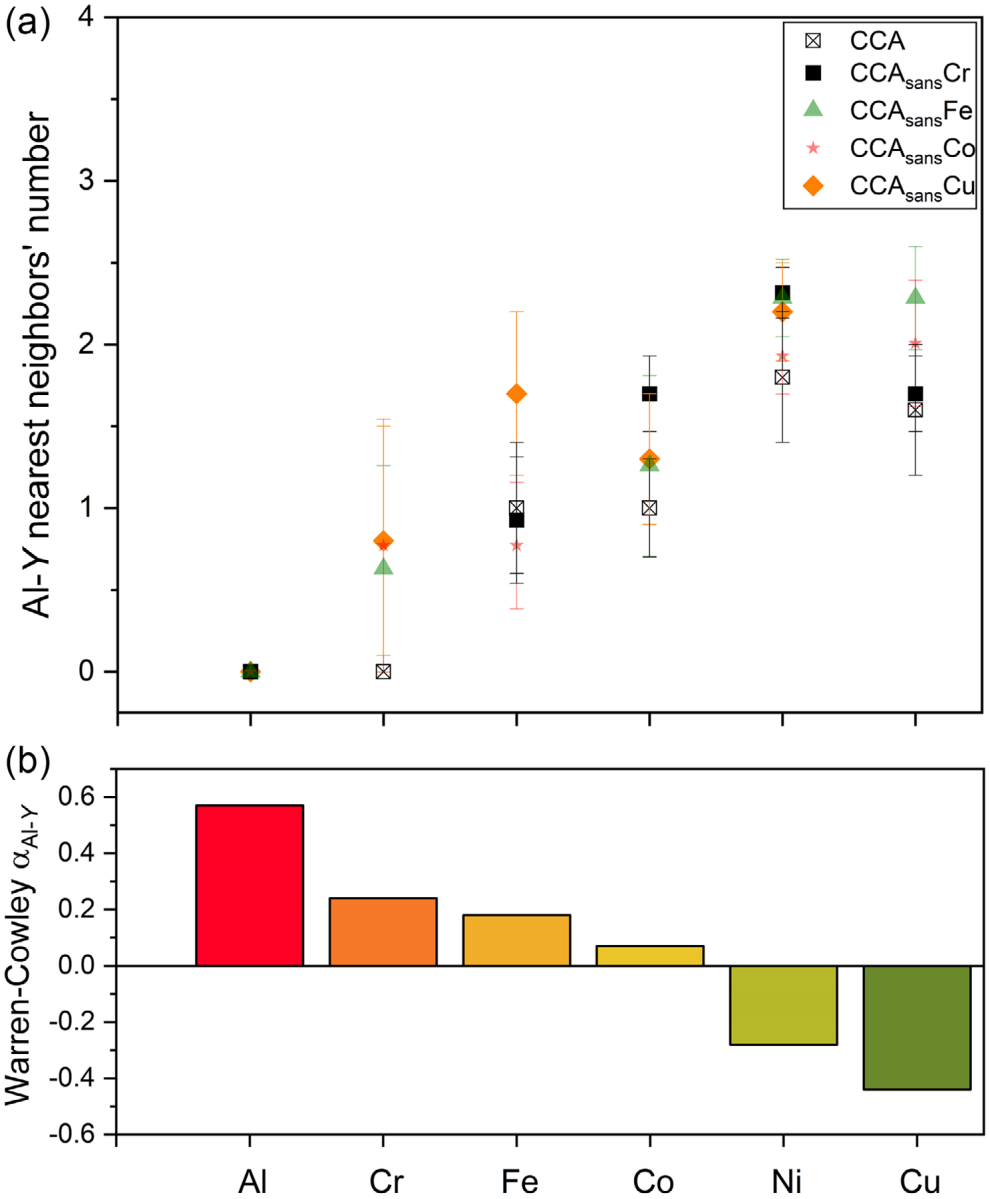
Chemical SRO of Al in the 1^st^ shell of each absorber Y. a) Al nearest neighbors’ number in CCA_sans_ *X* (*X* = Ø, Cr, Fe, Co, Cu) determined by EXAFS fits. b) MC/MD calculated WC parameters for Al‐Y pairs in the CCA (Table S3, Supporting Information); positive values: rejection (toward red color); negative values: preference (toward green color).

The effect of removing elements from the CCA is also evaluated from the chemical SRO point of view around Al, the only element with enough X‐ray scattering contrast to be separated from the other 3d alloying elements in the EXAFS data analysis. Results are depicted in **Figure** **11**, where the 1^st^ shell Al nearest neighbor number is established for each compound and each element.

The general trend shown for the mother compound CCA^[^ ^26^ ^]^ with Al–Ni and Al–Cu preferences and no Al–Al pairs is in first approximation confirmed for all alloys, both experimentally and by MC/MD calculations. Al–Cr pairs are also limited, though to a lesser extent than Al–Al. In CCA_sans_Cr, a rise in Al–Co preferred pairing comparable with Al–Cu is observed, while in CCA_sans_Cu by removing the most preferred Al–Cu (also the only nonmagnetic pair) and Cu–Cu pairs, there is a tendency of a more homogeneous distribution of Al around the other elements. A direct comparison between experimental SRO and simulation work can be carried out by reporting such results together in **Table** **3**, where also the weighted average of theoretical data according to composition, pair correlation intensities, and experimentally determined SRO is calculated.

**Table 3:** Experimental *C*‐Al averaged distance by EXAFS and differently weighed *C*‐Al distances calculated by hybrid MC/MD. Weight: stoichiometry, pair correlation intensities *g*_αβ_, and FNN established by EXAFS

Weight	AVG	Stoichiometry	*g*_αβ_	FNN
*C*‐Al	2.54(1)	2.57(1)	2.57(1)	2.54(1)

As shown in Table 3, different weights were used to determine the average *C*‐Al distance according to different SRO models, so that simulation and experiment can be exhaustively compared among each other. The experimental *C*‐Al distance determined by EXAFS, averaged (AVG), is 2.54(1) (Å). In the second column, a completely disordered model is assumed, meaning that the average distances calculated by MC/MD are weighted on the alloy composition and determined to be 2.57 Å. Next, a model assuming the weight as the pair correlation function *g*_αβ_ is carried out, obtaining a *C*‐Al distance of 2.57 Å, comparable to the fully disordered model. Finally, if weighting the MC/MD distances with the first nearest neighbor (FNN) number obtained by EXAFS (last column), the simulated average distance decreases within the EXAFS uncertainty, resulting in a *C*‐Al of 2.54 Å, showing how accounting experimentally established SRO is crucial to enhance calculation accuracy.

The bond lengths in Figure 10, showing a minimum at Co–*M*, do not follow precisely the atomic sizes Al > Cr > Fe > Co > Ni < Cu, with a minimum at Ni. This is confirmed by the simulated bond lengths when average separations of each element from the others are looked at: Al–*Y* = 2.57 Å; Cr–*Y* = 2.53 Å; Fe–*Y* = 2.52 Å; Co–*Y* = 2.51 Å; Ni–*Y* = 2.52 Å; Cu–*Y* = 2.55 Å. The distance increase with Z number from Co–*M* to Cu–*M* (Co–*M* < Ni–*M* < Cu–*M*) has to do with the progressive filling of the d‐orbitals. Note that Ni has the highest electronegativity of all elements present, so its d‐band is likely to be filled, meaning that it might begin to behave more free‐electron‐like than Co. This is corroborated by Bader charges analysis (cf. Figure S6, Supporting Information).

Despite EXAFS determining bond distances locally around a specific element, such distances become more and more “averaged” when increasing the distance from the absorber because of multiple‐scattering paths, ideally converging to the long‐range structure determined by XRD. This is one of the reasons why EXAFS fits were extended as much as possible, i.e., until the 4^th^ shell (≈5.2 Å), to experimentally establish until which distance distortions may subsist. Such modeling is the basis for a possible link between SRO, distortion, and physical properties, namely hardness, and allowed proposing an improvement of the frequently used atomic‐size mismatch parameter *δ*.

**Figure 12:**
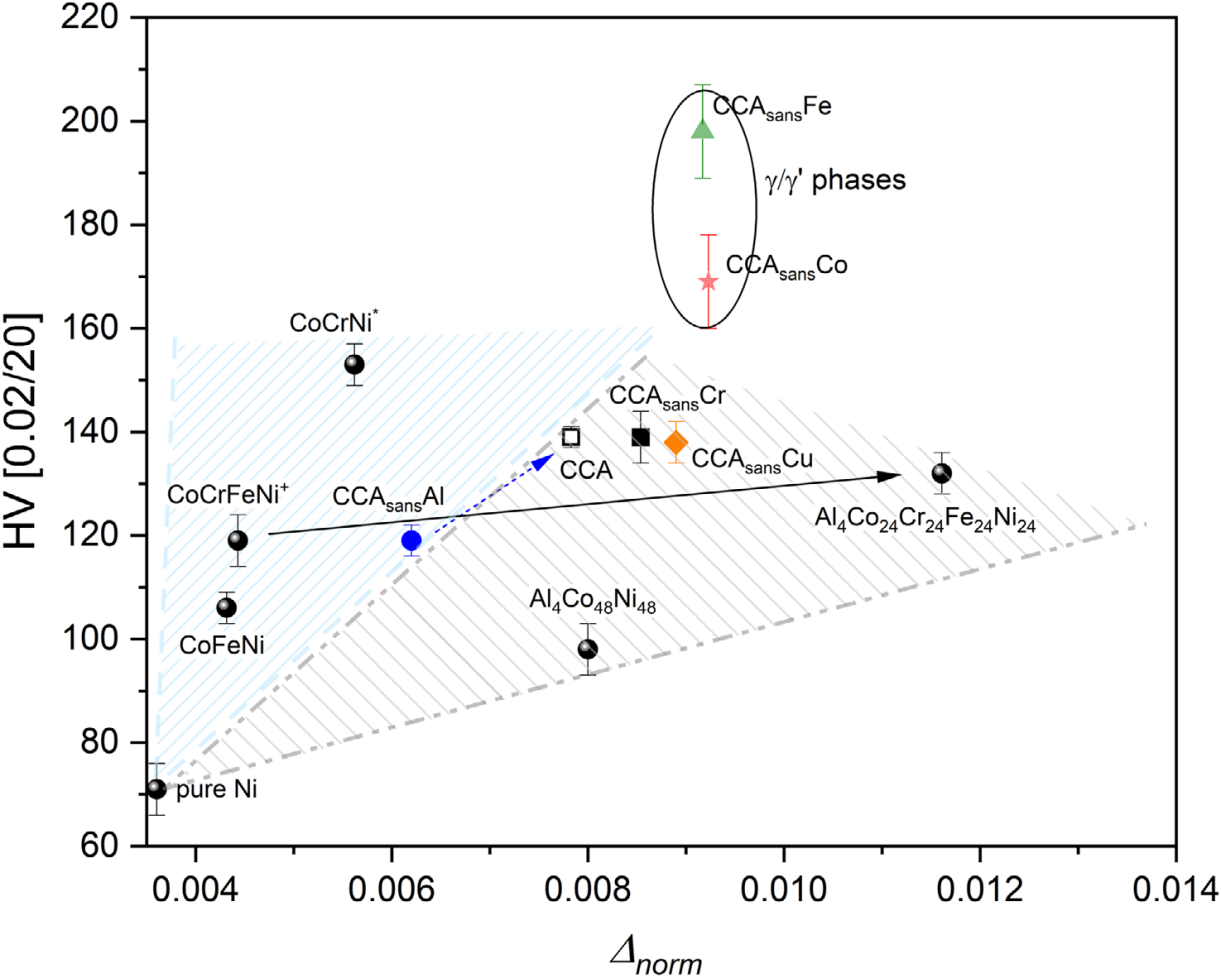
Vickers hardness (HV) as a function of $\Delta_{\text{norm}}$ (cf. formula above), for several Ni‐based fcc compounds. *CoCrNi EXAFS data was measured at *T* = 80 K, XRD and HV at room temperature. XRD data at 80 K were calculated from the known CoCrNi thermal expansion reported in ref. [55]. ^+^CoCrFeNi data^[^ ^56^ ^]^ were measured at KMC3, BESSYII.

The atomic radii in *δ* are often used as the tabulated values, not considering SRO and/or orbital interactions which finally results in an effective atomic radius generally different than the tabulated one. By considering experimentally measured effects in the local structure up to the 4^th^ shell and relating them to XRD distances, a total distortion value $\Delta_{\text{norm}}$ can be defined as:

(1) $$\Delta_{\text{norm}}=\sqrt{\sum_{i}{\left(\frac{{\text{AVG}}_{\left(\text{XAS}\right)i}-{\text{AVG}}_{\left(\text{XRD}\right)i}}{{\text{AVG}}_{\left(\text{XRD}\right)i}}\right)}^{2}}$$

where i represents the shell number and runs between 1 and 4, and norm stands for the normalization on AVG_XRD_ values at the denominator, making $\Delta_{\text{norm}}$ a normalized mean squared value between average shell distances (XRD) and local shell distances (XAS), summed up to 5.2 Å. $\Delta_{\text{norm}}$ represents then a fully experimental value which intrinsically considers charge transfer effects or chemical SRO accounted, e.g., in ref. [53] in a HfNbTiZr HEA. HV values have been plotted as a function of the obtained $\Delta_{\text{norm}}$ values in **Figure** **12**. For better statistics, pure Ni and several single‐phased fcc‐based alloys (Al_4_Co_48_Ni_48_, CoFeNi, CoCrNi, CoCrFeNi, Al_4_Co_24_Cr_24_Fe_24_Ni_24_), were added in the HV–$\Delta_{\text{norm}}$ plot. The fcc‐based alloys with three or four components have values of configurational entropies corresponding to medium entropy alloys (MEA) alloys except in the Al_4_Co_48_Ni_48_ case (S_config_ = 0.83 R, low entropy) and in the Al_4_Co_24_Cr_24_Fe_24_Ni_24_ case (S_config_ = 1.50 R, HEA).

Pure Ni is used as an ideal baseline for $\Delta_{\text{norm}}$ to interpret the determined distortional parameters, based on the assumption that pure elements show negligible distortions. Excluding the γ/γ′ region, biased by multiphasic specimens, two distinct regions appear (labeled with filled triangles) where the discriminant seems to be the presence of Al. The light blue (left) triangle delineates a region of lower distortions for specimens without Al where CoFeNi, CoCrNi, CoCrFeNi, and CCA_sans_Al lie, while higher distortions for the Al‐containing CCA, CCA_sans_Cr, CCA_sans_Cu, Al_4_Co_24_Cr_24_Fe_24_Ni_24_, and Al_4_Co_48_Ni_48_ are highlighted in the light gray (right) triangle. From this fact, one can infer that adding Al has an effect in increasing the overall distortions of the system. Adding Al seems also to slightly increase hardness together with the distortions, though this can be shown only for two depicted alloys: CCA_sans_Al → CCA and CoCrFeNi → Al_4_Co_24_Cr_24_Fe_24_Ni_24_, which move from bottom‐left to upper‐right (cf. arrows in Figure 12). Overall, considering the data altogether, a proportional correlation HV–$\Delta_{\text{norm}}$ is proposed, with different proportionality values as a function of the composition (with Al vs without Al); however, more data points are essential to validate and/or extend this correlation to different MEA/HEA systems and possibly isolate the role of more specific alloying elements.

Finally, a conclusion on the role of Co and Fe absence in the CCA is suggested. In previous studies, it has often been found that Co was the most “compatible” element with all the others, made visible by the smallest fluctuations in concentration in one alloy between a disordered phase (like A1/γ or A2) and its ordered companion (like L1_2_/γ′ or B2), both in fcc^[^ ^54^ ^]^ and bcc^[^ ^32^ ^]^ structured alloys. This element seems to serve like a kit holding everything together. Fe removal shows a similar drive to phase separation in the present work, i.e., Fe shows signs of “holding together” the alloy, similarly to Co. Such behavior of Fe is not reported in the literature, being an interesting turn, which has to be further investigated.

## 5 Conclusion

The systematic study carried out on the Al_8_Co_17_Cr_17_Cu_8_Fe_17_Ni_33_ and its quinary subsystems with one element removed, series labeled as CCA_sans_ *X* (*X* = Ø, Al, Cr, Co, Cu, Fe), demonstrated how specific element removal influences both atomic‐scale properties of the material but also macroscopic properties such as hardness. Experimentally determined atomic distortions and variations in the electronic structure were correlated with material performance, highlighting once more the correlation of some effects in HEA, i.e., “baseless” systems with no predominating elements, to the intrinsic nature of the solid solution.

It was found that the presence or absence of the elements Cu and Cr (≈8 and 17 at%, respectively) has the least influence neither on the local atomic structural deviations nor on the hardness. Removing Al reduces both hardness and the variation in bond lengths—as expected in an alloy consisting of only transition elements. Removing Co and Fe from the senary alloys implies a change in homogeneity, i.e., a formation of a second phase, concluding that rather than the quantity, it is the type of the element removed that drives specific behaviors. More generally, the contribution to microhardness values is defined by local distortions; however, such local distortions include also chemical short‐range ordering, and it is not possible to separate such contributions unequivocally with the techniques used in the present work. The relations hardness–distortions and hardness–orbitals were demonstrated acceptably valid, serving as a base for future work on the alloy family. As only Ni‐containing fcc multicomponent alloys were included, this leaves space for future investigations in symmetry‐dependent or alternative compositions.

## Conflict of Interest

The authors declare no conflict of interest.

## Supporting information

Supplementary Material

Supplementary Material

## Data Availability

The data that support the findings of this study are available from the corresponding author upon reasonable request.
