# Supplementary material for: How Atomic Bonding Plays the Hardness Behavior in the Al–Co–Cr–Cu–Fe–Ni High Entropy Family
Source: Small Sci. 2023 Dec 7;4(2):2300225. doi: 10.1002/smsc.202300225 (PMC11935079; doi:10.1002/smsc.202300225)
Supplement: Supplementary file 1 — Supplementary Material [file SMSC-4-2300225-s002.pdf]

EXAFS fits' details

Pure metal references

Al 2
Cr 3
Fe 4
Co 6
Ni 7
Cu 9

$CCA_{sans}X$

CCA 10
$CCA_{sans}Al$ 21
$CCA_{sans}Cr$ 28
$CCA_{sans}Fe$ 36
$CCA_{sans}Co$ 44
$CCA_{sans}Cu$ 52

Ternaries and quaternaries

$Al_4Co_{48}Ni_{48}$ 61
$Al_4Co_{24}Cr_{24}Fe_{24}Ni_{24}$ 64
CoCrNi 71
CoFeNi 75
CoCrFeNi 79

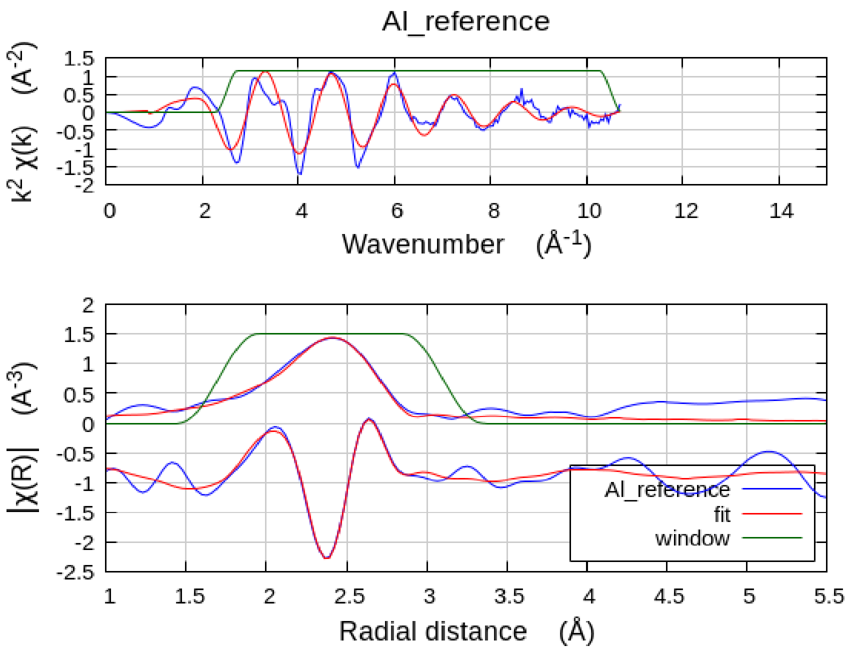

: name = Al_reference
: k-range = 2.5 - 10.5
: dk = 0.5
: k-window = Hanning
: k-weight = 1,2,3
: R-range = 1.7 - 3.1
: dR = 0.5
: R-window = Hanning
: fitting space = r
: background function = no
: phase correction = no
: background removal = E0: 1557.731462, Rbkg: 1.0, range: [0:10.769], clamps: 0/24, kw: 2
: user-supplied epsilon_k = 0
: epsilon_k by k-weight = 9.292e-04
: epsilon_r by k-weight = 1.255e-01
: R-factor by k-weight = 1 -> 0.00351, 2 -> 0.00580, 3 -> 0.01303

```
 name               N      S02    sigma^2   e0     delr     Reff     R
==========================================================================
==
[Al] Al1.1        12.000   0.652   0.01158   3.026 -0.02716  2.86340  2.83624
```

guess parameters:
  amp_Al = 0.65164997 # +/- 0.05389771 [0.65348]
  dEnot_Al = 3.02628132 # +/- 0.66078837 [2.71027]
  alpha = -0.00948616 # +/- 0.00353849 [-0.01018]
  ss00 = 0.01158154 # +/- 0.00155317 [0.01160]

Independent points : 6.9873047
Number of variables : 4
Chi-square : 241.8714852

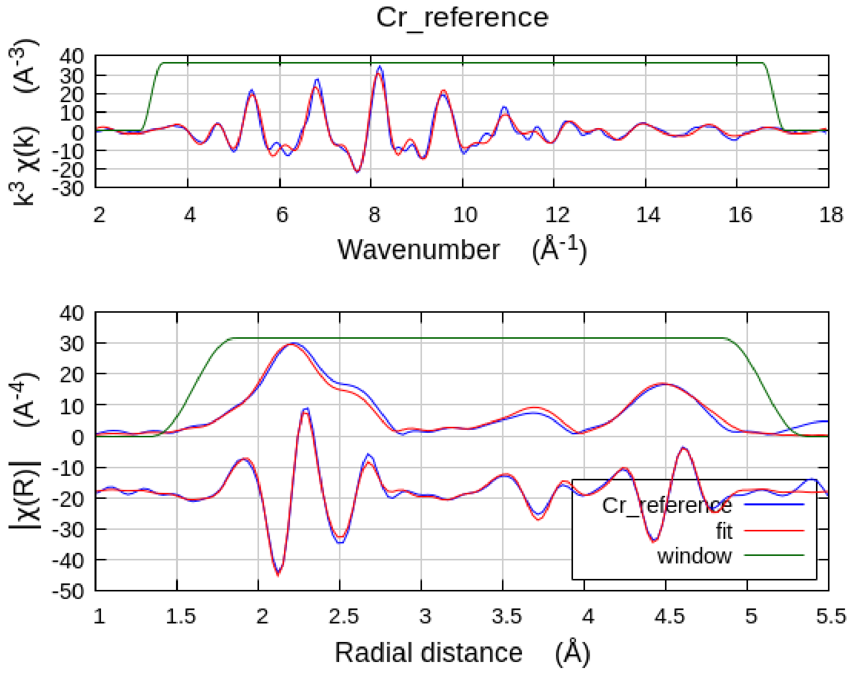

Reduced chi-square : 80.9664599
R-factor : 0.0074458
Number of data sets : 1
: name = Cr_reference
: k-range = 3.2 - 16.8
: dk = 0.5
: k-window = Hanning
: k-weight = 1,2,3
: R-range = 1.6 - 5.1
: dR = 0.5
: R-window = Hanning
: fitting space = r
: background function = no
: phase correction = no
: background removal = E0: 5990.25936755738, Rbkg: 1.0, range: [0:18.017], clamps: 0/24, kw: 2
: user-supplied epsilon_k = 0
: epsilon_k by k-weight = 5.765e-05
: epsilon_r by k-weight = 4.763e-02
: R-factor by k-weight = 1 -> 0.00993, 2 -> 0.01042, 3 -> 0.01396

name	N	S02	sigma^2	e0	delr	Reff	R
[Cr] Cr1.1	8.000	0.731	0.00526	3.727	-0.01170	2.49840	2.48670
[Cr] Cr1.2	6.000	0.731	0.00594	3.727	-0.01686	2.88490	2.86804
[Cr] Cr1.1 Cr1.1	24.000	0.731	0.00620	3.727	-0.01650	3.94090	3.92440
[Cr] Cr1.1 Cr1.2	48.000	0.731	0.00620	3.727	-0.01428	3.94090	3.92662
[Cr] Cr1.3	12.000	0.731	0.00678	3.727	-0.00267	4.07990	4.07723
[Cr] Cr1.1 Cr1.1	24.000	0.731	0.00678	3.727	-0.01650	4.53840	4.52190
[Cr] Cr1.1 Cr1.3	48.000	0.731	0.00678	3.727	-0.00719	4.53840	4.53121
[Cr] Cr1.4	24.000	0.731	0.00685	3.727	-0.01012	4.78410	4.77398
[Cr] Cr1.2 Cr1.2	24.000	0.731	0.00706	3.727	-0.03372	4.92490	4.89118
[Cr] Cr1.2 Cr1.3	48.000	0.731	0.00706	3.727	-0.00977	4.92490	4.91513

[Cr] Cr1.5	8.000	0.731	0.00688	3.727	0.01624	4.99690	5.01314
[Cr] Cr1.1 Cr1.1	8.000	0.731	0.00688	3.727	-0.02340	4.99690	4.97350
[Cr] Cr1.1 Cr1.5	16.000	0.731	0.00688	3.727	0.01624	4.99690	5.01314
[Cr] Cr1.1	8.000	0.731	0.00688	3.727	-0.02340	4.99690	4.97350
[Cr] Cr1.1 Cr1.5 Cr1.1	8.000	0.731	0.00688	3.727	0.01624	4.99690	5.01314
[Cr] Cr1.1 Cr1.2	48.000	0.731	0.00693	3.727	-0.01428	5.08380	5.06952
[Cr] Cr1.1 Cr1.4	48.000	0.731	0.00693	3.727	-0.01091	5.08380	5.07289
[Cr] Cr1.2 Cr1.4	48.000	0.731	0.00693	3.727	-0.01349	5.08380	5.07031
[Cr] Cr1.1	48.000	0.731	0.01462	3.727	-0.01428	5.38340	5.36912
[Cr] Cr1.1 Cr1.1 Cr1.1	48.000	0.731	0.01462	3.727	-0.01755	5.38340	5.36585
[Cr] Cr1.1 Cr1.1 Cr1.2	48.000	0.731	0.00651	3.727	-0.02013	5.38340	5.36327
[Cr] Cr1.1 Cr1.4 Cr1.2	48.000	0.731	0.00651	3.727	-0.01349	5.38340	5.36991
[Cr] Cr1.1	48.000	0.731	0.00868	3.727	-0.01428	5.38340	5.36912
[Cr] Cr1.1 Cr1.4 Cr1.1	48.000	0.731	0.00868	3.727	-0.01012	5.38340	5.37328

guess parameters:

amp	=	0.73107489	# +/-	0.04599551	[0.76090]
dEnot	=	3.72730754	# +/-	0.40152730	[3.85728]
thetaCr	=	461.57526067	# +/-	25.57088321	[436.48611]
ss1st	=	0.00525913	# +/-	0.00048937	[0.00549]
dR1st	=	-0.01169936	# +/-	0.00334932	[-0.01077]
dR2nd	=	-0.01685923	# +/-	0.00513113	[-0.01681]
dR3rd	=	-0.00267374	# +/-	0.00789305	[0.01]
dR4th	=	-0.01011976	# +/-	0.00885904	[0.01]
dR5th	=	0.01624143	# +/-	0.00859842	[0.01]

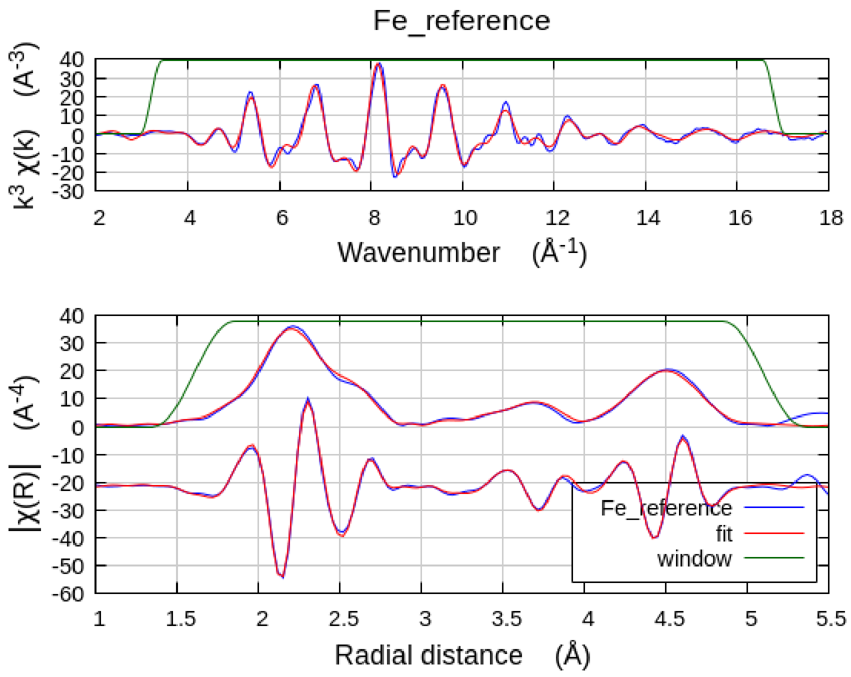

set parameters:
temperature = 300.00000000

Independent points : 29.9052734
Number of variables : 9
Chi-square : 49945.6742473

```
Reduced chi-square         : 2389.1423567
R-factor                   : 0.0114345
Number of data sets        : 1
: name                     = Fe_reference
: k-range                  = 3.2 - 16.8
: dk                       = 0.5
: k-window                 = Hanning
: k-weight                 = 1,2,3
: R-range                  = 1.6 - 5.1
: dR                       = 0.5
: R-window                 = Hanning
: fitting space            = r
: background function      = no
: phase correction         = no
: background removal       = E0: 7112.52831231642, Rbkg: 1.0, range: [0:18.025], clamps: 0/24, kw: 2
: user-supplied epsilon_k = 0
: epsilon_k by k-weight   = 1.127e-04
: epsilon_r by k-weight   = 9.311e-02
: R-factor by k-weight    = 1 -> 0.00378,  2 -> 0.00307,  3 -> 0.00491

  name                       N      S02     sigma^2   e0     delr     Reff     R
===============================================================================
========
[Fe] Fe1.1                 8.000   0.868   0.00563   6.139  0.01174  2.45840  2.47013
[Fe] Fe1.2                 6.000   0.868   0.00671   6.139  0.00528  2.83870  2.84398
[Fe] Fe1.1 Fe1.2          48.000   0.868   0.00696   6.139  0.00851  3.87780  3.88631
[Fe] Fe1.3                12.000   0.868   0.00788   6.139  0.03912  4.01460  4.05372
[Fe] Fe1.1 Fe1.3          48.000   0.868   0.00780   6.139  0.02543  4.46570  4.49113
[Fe] Fe1.4                24.000   0.868   0.00801   6.139  0.04383  4.70750  4.75133
[Fe] Fe1.5                 8.000   0.868   0.00805   6.139  0.04198  4.91680  4.95878
[Fe] Fe1.1 Fe1.5          16.000   0.868   0.00805   6.139  0.04198  4.91680  4.95878
[Fe] Fe1.1 Fe1.5 Fe1.1     8.000   0.868   0.00805   6.139  0.04198  4.91680  4.95878
[Fe] Fe1.1 Fe1.2          48.000   0.868   0.00810   6.139  0.00851  5.00230  5.01081
[Fe] Fe1.1 Fe1.4          48.000   0.868   0.00810   6.139  0.02778  5.00230  5.03008
[Fe] Fe1.2 Fe1.4          48.000   0.868   0.00810   6.139  0.02456  5.00230  5.02686
[Fe] Fe1.1 Fe1.1           8.000   0.868   0.00805   6.139  0.02347  4.91680  4.94027
[Fe] Fe1.1                 8.000   0.868   0.00805   6.139  0.02347  4.91680  4.94027
[Fe] Fe1.2 Fe1.3          48.000   0.868   0.00824   6.139  0.02220  4.84600  4.86820
[Fe] Fe1.1 Fe1.4          96.000   0.868   0.00842   6.139  0.02778  5.59030  5.61808
[Fe] Fe1.3 Fe1.4          96.000   0.868   0.00842   6.139  0.04147  5.59020  5.63168


guess parameters:
  amp            =   0.86798303    # +/-   0.04036455     [0.85190]
  dEnot          =   6.13919268    # +/-   0.26032841     [6.32116]
  dR1st          =   0.01173503    # +/-   0.00210761     [0.01205]
  thetaFe        = 408.53751371    # +/-  12.55146661     [412.60132]
  ss1st          =   0.00563249    # +/-   0.00034704     [0.00550]
  dR2nd          =   0.00528377    # +/-   0.00326838     [0.00686]
  dR3rd          =   0.03911792    # +/-   0.00506648     [0.04186]
  dR4th          =   0.04383200    # +/-   0.00730719     [0.04453]
  dR5th          =   0.04198291    # +/-   0.00710905     [0.04573]

set parameters:
  temperature    = 300.00000000
```

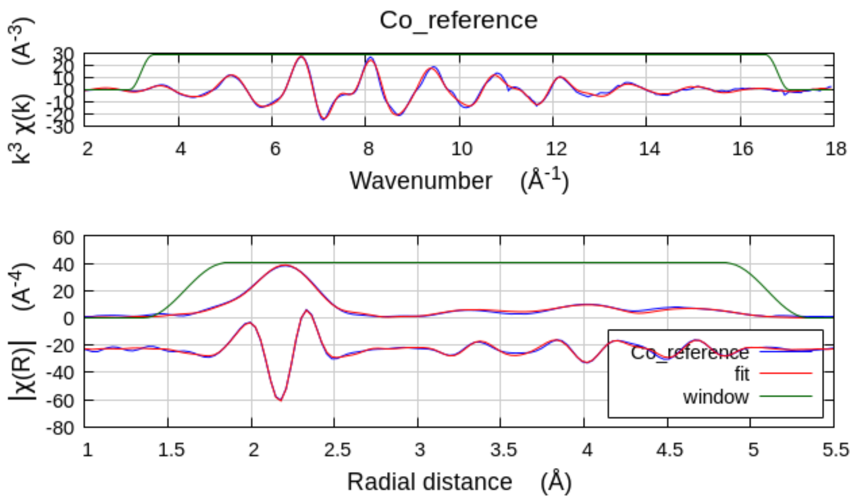

Independent points : 29.9052734
Number of variables : 9
Chi-square : 10724.0627297
Reduced chi-square : 512.9836145
R-factor : 0.0039197
Number of data sets : 1

: name = Co_reference
: k-range = 3.200 - 16.8
: dk = 0.5
: k-window = Hanning
: k-weight = 1,2,3
: R-range = 1.6 - 5.1
: dR = 0.5
: R-window = Hanning
: fitting space = r
: background function = no
: phase correction = no
: background removal = E0: 7710.84530731476, Rbkg: 1.0, range: [0.000:18.012], clamps: 0/24, kw: 2
: user-supplied epsilon_k = 0
: epsilon_k by k-weight = 1.600e-04
: epsilon_r by k-weight = 1.322e-01
: R-factor by k-weight = 1 -> 0.00559, 2 -> 0.00528, 3 -> 0.00700

name	N	S02	sigma^2	e0	delr	Reff	R
[Co] Co1.1	12.000	0.797	0.00655	8.311	-0.01273	2.50950	2.49677
[Co] Co1.3	6.000	0.797	0.00871	8.311	-0.00043	3.54890	3.54847
[Co] Co1.1 Co1.1	48.000	0.797	0.00792	8.311	-0.01273	3.76420	3.75147
[Co] Co1.1 Co1.2	24.000	0.797	0.00878	8.311	-0.00855	4.28400	4.27545
[Co] Co1.1 Co1.3	48.000	0.797	0.00878	8.311	-0.00658	4.28400	4.27742
[Co] Co1.5	18.000	0.797	0.00897	8.311	-0.00698	4.34770	4.34072
[Co] Co1.1 Co1.2	36.000	0.797	0.00901	8.311	-0.00855	4.68380	4.67525

[Co] Co1.1 Co1.5 72.000 0.797 0.00901 8.311 -0.00985 4.68380 4.67395
[Co] Co1.7 12.000 0.797 0.00904 8.311 -0.00474 4.80250 4.79776
[Co] Co1.1 Co1.1 12.000 0.797 0.00906 8.311 -0.02546 4.90950 4.88404
[Co] Co1.1 Co1.7 24.000 0.797 0.00906 8.311 -0.00873 4.90950 4.90077
[Co] Co1.8 6.000 0.797 0.00909 8.311 0.00191 5.02140 5.02331
[Co] Co1.2 Co1.8 12.000 0.797 0.00909 8.311 0.00191 5.02140 5.02331
[Co] Co1.2 Co1.8 Co1.2 6.000 0.797 0.00909 8.311 0.00191 5.02140 5.02331
[Co] Co1.1 12.000 0.797 0.02817 8.311 -0.02546 5.01900 4.99354
[Co] Co1.1 48.000 0.797 0.01759 8.311 -0.02546 5.01900 4.99354
[Co] Co1.1 Co1.2 Co1.1 48.000 0.797 0.01759 8.311 -0.01491 5.01900 5.00408
[Co] Co1.1 Co1.5 Co1.1 36.000 0.797 0.01153 8.311 -0.01622 5.01980 5.00358
[Co] Co1.2 Co1.2 6.000 0.797 0.00909 8.311 -0.00874 5.02140 5.01265
[Co] Co1.2 6.000 0.797 0.00909 8.311 -0.00874 5.02140 5.01265

guess parameters:
amp = 0.79652238 # +/- 0.02934981 [0.79652]
dEnot = 8.31132545 # +/- 0.38236282 [8.31130]
dR1st = -0.01272910 # +/- 0.00259912 [-0.01273]
thetaCo = 373.15821289 # +/- 13.33369413 [373.16068]
ss1st = 0.00654620 # +/- 0.00032667 [0.00655]
dR2nd = -0.00437228 # +/- 0.05119832 [-0.00436]
dR3rd = -0.00043306 # +/- 0.00873411 [-0.00043]
dR5th = -0.00697860 # +/- 0.00789693 [-0.00698]
dR7th = -0.00474053 # +/- 0.02984500 [-0.00475]
dR8th = 0.00191205 # +/- 0.02431800 [0.00191]

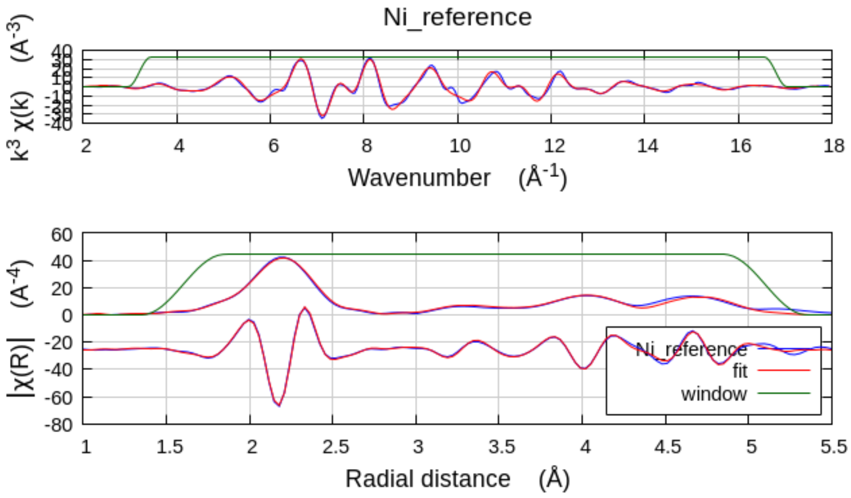

set parameters:
temperature = 300.00000000

Independent points : 29.9052734
Number of variables : 10
Chi-square : 11588.5931726
Reduced chi-square : 582.1870877
R-factor : 0.0059553
Number of data sets : 1

: name = Ni311_foil_01_1_r.dat
: k-range = 3.200 - 16.8

```
: dk                    = 0.5
: k-window              = Hanning
: k-weight              = 1,2,3
: R-range               = 1.6 - 5.1
: dR                    = 0.5
: R-window              = Hanning
: fitting space         = r
: background function   = no
: phase correction      = no
: background removal    = E0: 8333.5868093532, Rbkg: 1.0, range: [0:18.024], clamps: 0/24, kw: 2
: user-supplied epsilon_k = 0
: epsilon_k by k-weight  = 5.747e-05
: epsilon_r by k-weight  = 4.748e-02
: R-factor by k-weight   = 1 -> 0.00445,  2 -> 0.00456,  3 -> 0.00656

  name                      N      S02     sigma^2   e0     delr     Reff     R
================================================================================
========
[Ni] Ni1.1                12.000   0.842   0.00641   7.502 -0.00841  2.49440  2.48599
[Ni] Ni1.2                 6.000   0.842   0.00851   7.502 -0.00312  3.52760  3.52448
[Ni] Ni1.1 Ni1.1          48.000   0.842   0.00772   7.502 -0.01186  3.74160  3.72974
[Ni] Ni1.1 Ni1.1          24.000   0.842   0.00857   7.502 -0.01186  4.25820  4.24634
[Ni] Ni1.1 Ni1.2          48.000   0.842   0.00857   7.502 -0.00576  4.25820  4.25244
[Ni] Ni1.3                24.000   0.842   0.00877   7.502  0.00179  4.32040  4.32219
[Ni] Ni1.1 Ni1.1          48.000   0.842   0.00880   7.502 -0.01682  4.65460  4.63778
[Ni] Ni1.1 Ni1.3          96.000   0.842   0.00880   7.502 -0.00331  4.65460  4.65129
[Ni] Ni1.4                12.000   0.842   0.00888   7.502  0.00713  4.98880  4.99594
[Ni] Ni1.1 Ni1.1          12.000   0.842   0.00888   7.502 -0.01682  4.98880  4.97198
[Ni] Ni1.1 Ni1.4          24.000   0.842   0.00888   7.502  0.00713  4.98880  4.99594
[Ni] Ni1.1                12.000   0.842   0.00888   7.502 -0.01682  4.98880  4.97198
[Ni] Ni1.1 Ni1.4 Ni1.1    12.000   0.842   0.00888   7.502  0.00713  4.98880  4.99594

guess parameters:
  amp            =   0.84217748    # +/-   0.02797739     [0.86407]
  dEnot          =   7.50209399    # +/-   0.33598993     [7.62964]
  dR1st          =  -0.00841237    # +/-   0.00213846     [-0.00138]
  thetaNi        = 378.45318495    # +/-   7.99202112     [381.97821]
  ss1st          =   0.00641164    # +/-   0.00026859     [0.00300]
  dR2nd          =  -0.00311499    # +/-   0.00749683     [0.00100]
  dR3rd          =   0.00178635    # +/-   0.00477806     [0.00300]
  dR4th          =   0.00713531    # +/-   0.00515872     [0.00300]

set parameters:
  temperature    = 300.00000000
```

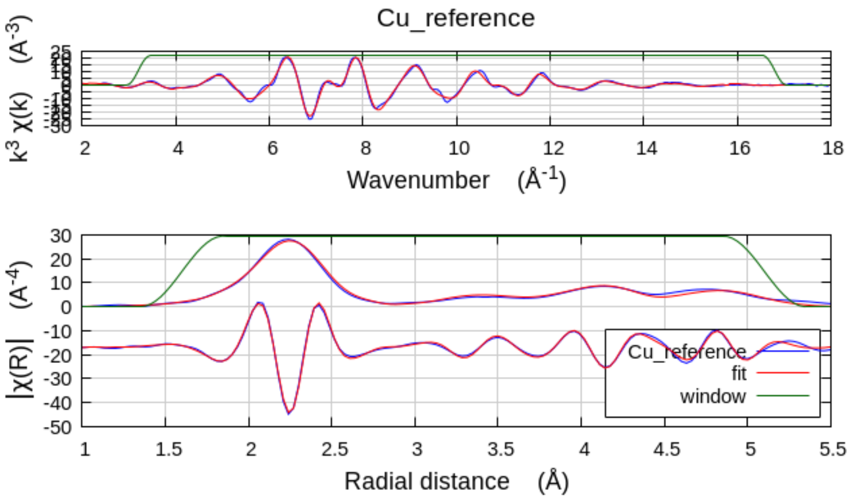

Independent points : 29.9052734
Number of variables : 8
Chi-square : 47423.9623907
Reduced chi-square : 2164.9564214
R-factor : 0.0051921
Number of data sets : 1

: name = Cu_reference
: k-range = 3.2 - 16.8
: dk = 0.5
: k-window = Hanning
: k-weight = 1,2,3
: R-range = 1.6 - 5.1
: dR = 0.5
: R-window = Hanning
: fitting space = r
: background function = no
: phase correction = no
: background removal = E0: 8983.5043, Rbkg: 1.0, range: [0:18], clamps: 0/24, kw: 2
: user-supplied epsilon_k = 0
: epsilon_k by k-weight = 5.615e-05
: epsilon_r by k-weight = 4.639e-02
: R-factor by k-weight = 1 -> 0.00426, 2 -> 0.00426, 3 -> 0.00631

name	N	S02	sigma^2	e0	delr	Reff	R
[Cu] Cu1.1	12.000	0.953	0.00917	3.929	-0.01398	2.55620	2.54222
[Cu] Cu1.2	6.000	0.953	0.01209	3.929	-0.00365	3.61500	3.61135
[Cu] Cu1.1 Cu1.1	48.000	0.953	0.01100	3.929	-0.01971	3.83430	3.81458
[Cu] Cu1.1 Cu1.1	24.000	0.953	0.01218	3.929	-0.01971	4.36370	4.34398
[Cu] Cu1.1 Cu1.2	48.000	0.953	0.01218	3.929	-0.00881	4.36370	4.35489
[Cu] Cu1.3	24.000	0.953	0.01246	3.929	0.00379	4.42750	4.43129
[Cu] Cu1.1 Cu1.1	48.000	0.953	0.01251	3.929	-0.02797	4.76990	4.74193
[Cu] Cu1.1 Cu1.3	96.000	0.953	0.01251	3.929	-0.00510	4.76990	4.76480

[Cu] Cu1.4 12.000 0.953 0.01263 3.929 0.01675 5.11240 5.12915
[Cu] Cu1.1 Cu1.1 12.000 0.953 0.01263 3.929 -0.02797 5.11240 5.08444
[Cu] Cu1.1 Cu1.4 24.000 0.953 0.01263 3.929 0.01675 5.11240 5.12915
[Cu] Cu1.1 12.000 0.953 0.01263 3.929 -0.02797 5.11240 5.08444
[Cu] Cu1.1 Cu1.4 Cu1.1 12.000 0.953 0.01263 3.929 0.01675 5.11240 5.12915

guess parameters:
amp = 0.95252248 # +/- 0.03425936 [0.95052]
dEnot = 3.92866708 # +/- 0.35426366 [3.90336]
dR1st = -0.01398254 # +/- 0.00234159 [-0.01417]
thetaCu = 302.37870805 # +/- 5.47340677 [302.47356]
ss1st = 0.00916675 # +/- 0.00031438 [0.00915]
dR2nd = -0.00364628 # +/- 0.00819347 [-0.00406]
dR3rd = 0.00378696 # +/- 0.00550927 [0.00367]
dR4th = 0.01675135 # +/- 0.00662693 [0.01632]

set parameters:
temperature = 300.00000000

Independent points : 29.9052734
Number of variables : 8
Chi-square : 29278.4195549
Reduced chi-square : 1336.5922885
R-factor : 0.0049408
Number of data sets : 1

CCA
Al-K; Cr-K

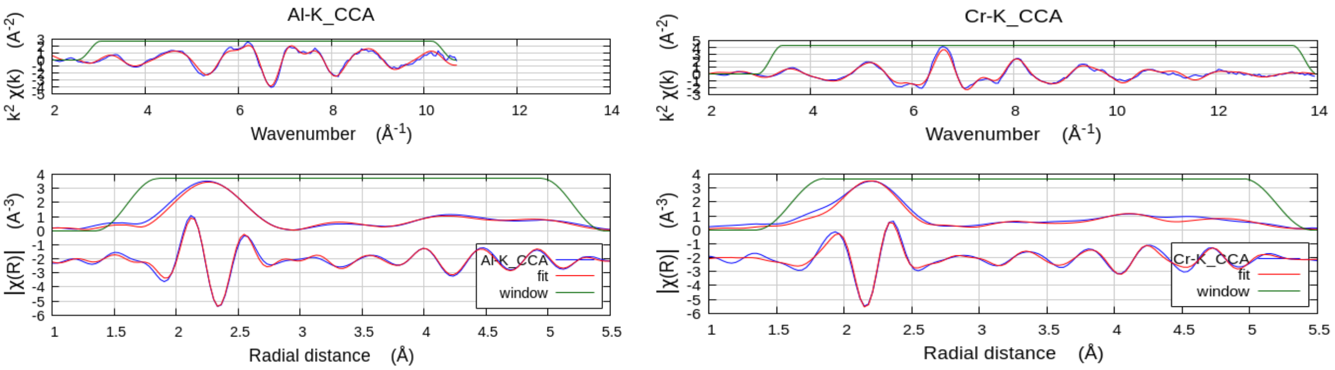

Independent points : 40.8945312
Number of variables : 15
Chi-square : 3582.1007677
Reduced chi-square : 138.3342580
R-factor : 0.0232515
Number of data sets : 2

guess parameters:
dEnot_Cr = 6.85008684 # +/- 0.87033055 [6.85017]
dEnot_Al = 6.92587707 # +/- 0.97883892 [6.92741]
thetaAl = 396.69041258 # +/- 25.26836552 [396.74145]

```
  thetaCr          = 363.38165920    # +/-  16.46710133     [363.38135]
  dRAl1st          =  -0.01109696    # +/-   0.00719917     [-0.01100]
  dRAl2nd          =  -0.02846873    # +/-   0.03481183     [-0.02909]
  dRAl3rd          =  -0.01332041    # +/-   0.02230001     [-0.01345]
  dRAl4th          =   0.02797429    # +/-   0.02727412     [0.02792]
  ssAlCo           =   0.00653844    # +/-   0.00050130     [0.00653]
  ssCrCo           =   0.00613758    # +/-   0.00036278     [0.00614]
  x                =  -0.00017218    # +/-   0.00679001     [-0.00017]
  dRCr1st          =  -0.03649688    # +/-   0.00574317     [-0.03650]
  dRCr2nd          =  -0.07273692    # +/-   0.02226374     [-0.07274]
  dRCr3rd          =  -0.01970954    # +/-   0.01472004     [-0.01971]
  dRCr4th          =   0.00075320    # +/-   0.01689350     [0.00075]

set parameters:
  amp_Cr           =   0.57000000
  amp_Al           =   0.66000000
  temperature      = 300.00000000

restrain parameters:
  res_x            =   0.17218281    # [:= 1000*penalty(x,0, 12)]

: name                     = Al-K_CCA
: k-range                  = 2.8 - 10.4
: dk                       = 0.5
: k-window                 = Hanning
: k-weight                 = 1,2,3
: R-range                  = 1.6 - 5.2
: dR                       = 0.5
: R-window                 = Hanning
: fitting space            = r
: background function      = no
: phase correction         = no
: background removal       = E0: 1557.237901, Rbkg: 1.0, range: [0:10.774], clamps: 0/24, kw: 2
: user-supplied epsilon_k = 0
: epsilon_k by k-weight   = 2.047e-03
: epsilon_r by k-weight   = 2.765e-01
: R-factor by k-weight    = 1 -> 0.02113,  2 -> 0.01673,  3 -> 0.01840

  name                                       N      S02     sigma^2   e0     delr     Reff     R
=================================================================================
[Al_absorber_Co_scatterer] Co1.1               12.000   0.660   0.00654   6.926 -0.01110  2.55270  2.54160
[Al_absorber_Co_scatterer] Co1.2                6.000   0.660   0.01260   6.926 -0.02847  3.61000  3.58153
[Al_absorber_Co_scatterer] Co1.1 Co1.1         48.000   0.660   0.01017   6.926 -0.01565  3.82900  3.81335
[Al_absorber_Co_scatterer] Co1.1 Co1.1         24.000   0.660   0.00989   6.926 -0.01565  4.35770  4.34205
[Al_absorber_Co_scatterer] Co1.1 Co1.2         48.000   0.660   0.01178   6.926 -0.01978  4.35770  4.33792
[Al_absorber_Co_scatterer] Co1.3               24.000   0.660   0.01294   6.926 -0.01332  4.42130  4.40798
[Al_absorber_Co_scatterer] Co1.1 Co1.1         48.000   0.660   0.00906   6.926 -0.02219  4.76330  4.74111
[Al_absorber_Co_scatterer] Co1.1 Co1.3         96.000   0.660   0.01254   6.926 -0.01221  4.76330  4.75109
[Al_absorber_Co_scatterer] Co1.4               12.000   0.660   0.01308   6.926  0.02797  5.10530  5.13327
[Al_absorber_Co_scatterer] Co1.1 Co1.1         12.000   0.660   0.00809   6.926 -0.02219  5.10530  5.08311
[Al_absorber_Co_scatterer] Co1.1 Co1.4         24.000   0.660   0.01308   6.926  0.02797  5.10530  5.13327
[Al_absorber_Co_scatterer] Co1.1               12.000   0.660   0.00809   6.926 -0.02219  5.10530  5.08311
[Al_absorber_Co_scatterer] Co1.1 Co1.4 Co1.1   12.000   0.660   0.01308   6.926  0.02797  5.10530  5.13327

: name                     = Cr-K_CCA
: k-range                  = 3.2 - 13.75
```

: dk = 0.5
: k-window = Hanning
: k-weight = 1,2,3
: R-range = 1.6 - 5.2
: dR = 0.5
: R-window = Hanning
: fitting space = r
: background function = no
: phase correction = no
: background removal = E0: 5989.6, Rbkg: 1.0, range: [0:14.015], clamps: 0/24, kw: 2
: user-supplied epsilon_k = 0
: epsilon_k by k-weight = 8.141e-04
: epsilon_r by k-weight = 2.783e-01
: R-factor by k-weight = 1 -> 0.01681, 2 -> 0.02412, 3 -> 0.04232

name	N	S02	sigma^2	e0	delr	Reff	R
[Cr_absorber_Co_scatterer] Co1.1	1.000	6.840	0.00614	6.850	-0.03650	2.55270	2.51620
[Cr_absorber_Co_scatterer] Co1.2	6.000	0.570	0.00978	6.850	-0.07274	3.61000	3.53726
[Cr_absorber_Co_scatterer] Co1.1 Co1.1	48.000	0.570	0.00870	6.850	-0.05146	3.82900	3.77754
[Cr_absorber_Co_scatterer] Co1.1 Co1.1	24.000	0.570	0.00948	6.850	-0.05146	4.35770	4.30624
[Cr_absorber_Co_scatterer] Co1.1 Co1.2	48.000	0.570	0.00973	6.850	-0.05462	4.35770	4.30308
[Cr_absorber_Co_scatterer] Co1.3	24.000	0.570	0.01007	6.850	-0.01971	4.42130	4.40159
[Cr_absorber_Co_scatterer] Co1.1 Co1.1	48.000	0.570	0.00960	6.850	-0.07299	4.76330	4.69031
[Cr_absorber_Co_scatterer] Co1.1 Co1.3	96.000	0.570	0.01005	6.850	-0.02810	4.76330	4.73520
[Cr_absorber_Co_scatterer] Co1.4	12.000	0.570	0.01020	6.850	0.00075	5.10530	5.10605
[Cr_absorber_Co_scatterer] Co1.1 Co1.1	12.000	0.570	0.00956	6.850	-0.07299	5.10530	5.03231
[Cr_absorber_Co_scatterer] Co1.1 Co1.4	24.000	0.570	0.01020	6.850	0.00075	5.10530	5.10605
[Cr_absorber_Co_scatterer] Co1.1	12.000	0.570	0.00956	6.850	-0.07299	5.10530	5.03231
[Cr_absorber_Co_scatterer] Co1.1 Co1.4 Co1.1	12.000	0.570	0.01020	6.850	0.00075	5.10530	5.10605
[Cr_absorber_Al_scatterer] Al1.1	1.000	-0.000	0.00654	6.850	-0.01110	2.55270	2.54160

Al-K; Fe-K

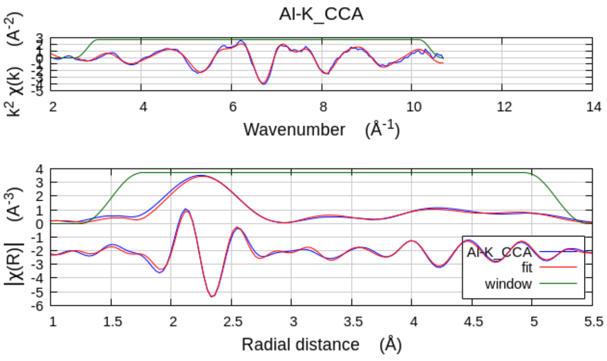

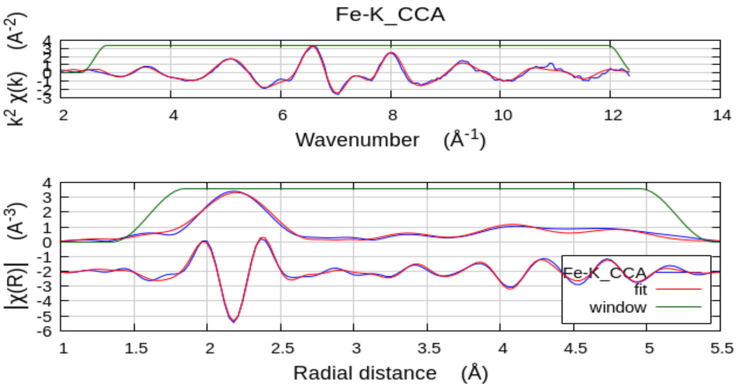

Independent points : 39.3320313
Number of variables : 15

Chi-square : 2632.3507061
Reduced chi-square : 108.1845851
R-factor : 0.0202555
Number of data sets : 2

guess parameters:
dEnot_Fe = 7.08330798 # +/- 0.71111123 [7.00367]
dEnot_Al = 6.94471096 # +/- 0.86311276 [6.95835]
thetaAl = 396.81328077 # +/- 22.38252359 [396.81185]
thetaFe = 317.58599001 # +/- 12.13397107 [310.64464]
ssAlCo = 0.00653039 # +/- 0.00044199 [0.00653]
ssCoFe = 0.00750125 # +/- 0.00060296 [0.00776]
dRFe1st = -0.03422681 # +/- 0.00535679 [-0.03483]
dRFe2nd = -0.00878740 # +/- 0.02311591 [-0.00625]
dRFe3rd = -0.00971142 # +/- 0.01414606 [-0.01000]
dRFe4th = -0.01064854 # +/- 0.01647379 [-0.01134]
dRAl1st = -0.01090807 # +/- 0.00637263 [-0.01079]
dRAl2nd = -0.02900720 # +/- 0.03072777 [-0.02890]
dRAl3rd = -0.01456760 # +/- 0.02012473 [-0.01438]
dRAl4th = 0.02752733 # +/- 0.02415982 [0.02766]
x = 0.98543377 # +/- 0.38163381 [1.19324]

set parameters:
amp_Fe = 0.75000000
amp_Al = 0.66000000
temperature = 300.00000000

: name = Al-K_CCA
: k-range = 2.8 - 10.4
: dk = 0.5
: k-window = Hanning
: k-weight = 1,2,3
: R-range = 1.5 - 5.2
: dR = 0.5
: R-window = Hanning
: fitting space = r
: background function = no
: phase correction = no
: background removal = E0: 1557.237901, Rbkg: 1.0, range: [0:10.774], clamps: 0/24, kw: 2
: user-supplied epsilon_k = 0
: epsilon_k by k-weight = 2.047e-03
: epsilon_r by k-weight = 2.765e-01
: R-factor by k-weight = 1 -> 0.02207, 2 -> 0.01735, 3 -> 0.01877

name	N	S02	sigma^2	e0	delr	Reff	R
[Al_absorber_Co_scatterer] Co1.1	12.000	0.660	0.00653	6.945	-0.01091	2.55270	2.54179
[Al_absorber_Co_scatterer] Co1.2	6.000	0.660	0.01259	6.945	-0.02901	3.61000	3.58099
[Al_absorber_Co_scatterer] Co1.1 Co1.1	48.000	0.660	0.01016	6.945	-0.01527	3.82900	3.81373
[Al_absorber_Co_scatterer] Co1.1 Co1.1	24.000	0.660	0.00989	6.945	-0.01527	4.35770	4.34243
[Al_absorber_Co_scatterer] Co1.1 Co1.2	48.000	0.660	0.01177	6.945	-0.01996	4.35770	4.33774
[Al_absorber_Co_scatterer] Co1.3	24.000	0.660	0.01293	6.945	-0.01457	4.42130	4.40673
[Al_absorber_Co_scatterer] Co1.1 Co1.1	48.000	0.660	0.00906	6.945	-0.01527	4.76330	4.74803
[Al_absorber_Co_scatterer] Co1.1 Co1.3	96.000	0.660	0.01253	6.945	-0.01274	4.76330	4.75056

```
[Al_absorber_Co_scatterer] Co1.4              12.000   0.660   0.01308   6.945  0.02753  5.10530  5.13283
[Al_absorber_Co_scatterer] Co1.1 Co1.1        12.000   0.660   0.00809   6.945 -0.02182  5.10530  5.08348
[Al_absorber_Co_scatterer] Co1.1 Co1.4        24.000   0.660   0.01308   6.945  0.02753  5.10530  5.13283
[Al_absorber_Co_scatterer] Co1.1              12.000   0.660   0.00809   6.945 -0.02182  5.10530  5.08348
[Al_absorber_Co_scatterer] Co1.1 Co1.4 Co1.1  12.000   0.660   0.01308   6.945  0.02753  5.10530  5.13283


: name                 = Fe-K_CCA
: k-range              = 2.6 - 12.2
: dk                   = 0.5
: k-window             = Hanning
: k-weight             = 1,2,3
: R-range              = 1.6 - 5.2
: dR                   = 0.5
: R-window             = Hanning
: fitting space        = r
: background function  = no
: phase correction     = no
: background removal   = E0: 7112.8068, Rbkg: 1.0, range: [0:12.396], clamps: 0/24, kw: 2
: user-supplied epsilon_k = 0
: epsilon_k by k-weight   = 1.279e-03
: epsilon_r by k-weight   = 2.855e-01
: R-factor by k-weight    = 1 -> 0.01474,  2 -> 0.01844,  3 -> 0.03018

  name                                          N       S02     sigma^2   e0     delr     Reff     R
=========================================================================================
[Fe_absorber_Al_scatterer] Al1.1               1.000   0.739   0.00653   7.083 -0.01091  2.55270  2.54179
[Fe_absorber_Co_scatterer] Co1.1               1.000   8.261   0.00750   7.083 -0.03423  2.55270  2.51847
[Fe_absorber_Co_scatterer] Co1.2               6.000   0.750   0.01217   7.083 -0.00879  3.61000  3.60121
[Fe_absorber_Co_scatterer] Co1.1 Co1.1        48.000   0.750   0.01094   7.083 -0.04826  3.82900  3.78074
[Fe_absorber_Co_scatterer] Co1.1 Co1.1        24.000   0.750   0.01205   7.083 -0.04826  4.35770  4.30944
[Fe_absorber_Co_scatterer] Co1.1 Co1.2        48.000   0.750   0.01218   7.083 -0.02151  4.35770  4.33619
[Fe_absorber_Co_scatterer] Co1.3              24.000   0.750   0.01255   7.083 -0.00971  4.42130  4.41159
[Fe_absorber_Co_scatterer] Co1.1 Co1.1        48.000   0.750   0.01232   7.083 -0.06845  4.76330  4.69485
[Fe_absorber_Co_scatterer] Co1.1 Co1.3        96.000   0.750   0.01256   7.083 -0.02197  4.76330  4.74133
[Fe_absorber_Co_scatterer] Co1.4              12.000   0.750   0.01272   7.083 -0.01065  5.10530  5.09465
[Fe_absorber_Co_scatterer] Co1.1 Co1.1        12.000   0.750   0.01238   7.083 -0.06845  5.10530  5.03685
[Fe_absorber_Co_scatterer] Co1.1 Co1.4        24.000   0.750   0.01272   7.083 -0.01065  5.10530  5.09465
[Fe_absorber_Co_scatterer] Co1.1              12.000   0.750   0.01238   7.083 -0.06845  5.10530  5.03685
[Fe_absorber_Co_scatterer] Co1.1 Co1.4 Co1.1  12.000   0.750   0.01272   7.083 -0.01065  5.10530  5.09465
```

Al-K; Co-K

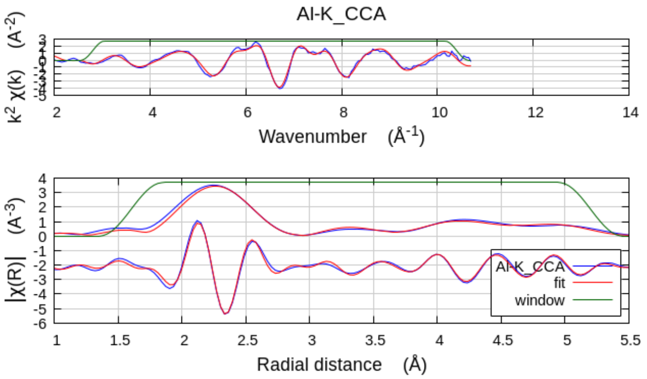

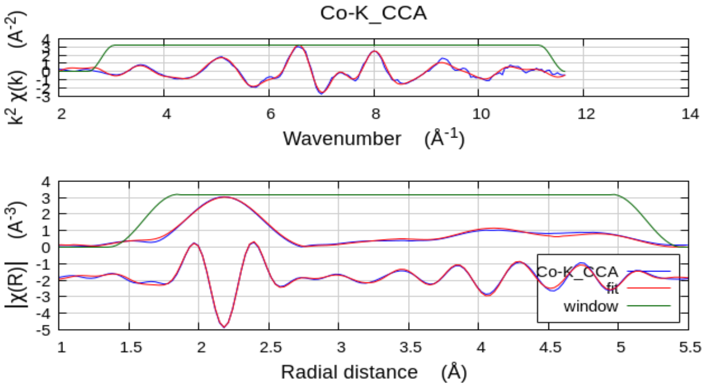

Independent points : 36.4765625
Number of variables : 15
Chi-square : 1738.3755699
Reduced chi-square : 80.9429149
R-factor : 0.0129286
Number of data sets : 2

guess parameters:
dEnot_Co = 7.84543441 # +/- 0.67546888 [7.80188]
dEnot_Al = 6.95036265 # +/- 0.74793731 [6.95056]
thetaAl = 396.69145660 # +/- 19.32677736 [396.68976]
thetaCo = 323.14113451 # +/- 9.94975098 [319.03011]
dRCo1st = -0.03792596 # +/- 0.00492002 [-0.03802]
dRCo2nd = -0.00476987 # +/- 0.01741598 [-0.00505]
dRCo3rd = -0.01397930 # +/- 0.01120391 [-0.01481]
dRCo4th = -0.00946280 # +/- 0.01293539 [-0.01059]
ssCoCo = 0.00837032 # +/- 0.00056569 [0.00858]
ssCoAl = 0.00654060 # +/- 0.00038363 [0.00654]
dRAl1st = -0.01088227 # +/- 0.00550047 [-0.01088]
dRAl2nd = -0.02827374 # +/- 0.02662839 [-0.02828]
dRAl3rd = -0.01302589 # +/- 0.01705824 [-0.01302]
dRAl4th = 0.02819471 # +/- 0.02086243 [0.02820]
x = 0.96057885 # +/- 0.34305702 [1.00131]

set parameters:
amp_Co = 0.78000000
amp_Al = 0.66000000
temperature = 300.00000000

: name = Al-K_CCA
: k-range = 2.8 - 10.4
: dk = 0.5
: k-window = Hanning
: k-weight = 1,2,3
: R-range = 1.6 - 5.2
: dR = 0.5
: R-window = Hanning
: fitting space = r
: background function = no

: phase correction = no
: background removal = E0: 1557.237901, Rbkg: 1.0, range: [0:10.774], clamps: 0/24, kw: 2
: user-supplied epsilon_k = 0
: epsilon_k by k-weight = 2.047e-03
: epsilon_r by k-weight = 2.765e-01
: R-factor by k-weight = 1 -> 0.02108, 2 -> 0.01677, 3 -> 0.01848

name	N	S02	sigma^2	e0	delr	Reff	R
[Al_absorber_Co_scatterer] Co1.1	12.000	0.660	0.00654	6.950	-0.01088	2.55270	2.54182
[Al_absorber_Co_scatterer] Co1.2	6.000	0.660	0.01260	6.950	-0.02827	3.61000	3.58173
[Al_absorber_Co_scatterer] Co1.1 Co1.1	48.000	0.660	0.01017	6.950	-0.01534	3.82900	3.81366
[Al_absorber_Co_scatterer] Co1.1 Co1.1	24.000	0.660	0.00989	6.950	-0.01534	4.35770	4.34236
[Al_absorber_Co_scatterer] Co1.1 Co1.2	48.000	0.660	0.01178	6.950	-0.01958	4.35770	4.33812
[Al_absorber_Co_scatterer] Co1.3	24.000	0.660	0.01294	6.950	-0.01303	4.42130	4.40827
[Al_absorber_Co_scatterer] Co1.1 Co1.1	48.000	0.660	0.00906	6.950	-0.02176	4.76330	4.74153
[Al_absorber_Co_scatterer] Co1.1 Co1.3	96.000	0.660	0.01254	6.950	-0.01195	4.76330	4.75135
[Al_absorber_Co_scatterer] Co1.4	12.000	0.660	0.01308	6.950	0.02820	5.10530	5.13349
[Al_absorber_Co_scatterer] Co1.1 Co1.1	12.000	0.660	0.00809	6.950	-0.02176	5.10530	5.08353
[Al_absorber_Co_scatterer] Co1.1 Co1.4	24.000	0.660	0.01308	6.950	0.02820	5.10530	5.13349
[Al_absorber_Co_scatterer] Co1.1	12.000	0.660	0.00809	6.950	-0.02176	5.10530	5.08353
[Al_absorber_Co_scatterer] Co1.1 Co1.4 Co1.1	12.000	0.660	0.01308	6.950	0.02820	5.10530	5.13349

: name = Co-K_CCA
: k-range = 2.8 - 11.4
: dk = 0.5
: k-window = Hanning
: k-weight = 1,2,3
: R-range = 1.6 - 5.2
: dR = 0.5
: R-window = Hanning
: fitting space = r
: background function = no
: phase correction = no
: background removal = E0: 7709.042937, Rbkg: 1.0, range: [0:11.72], clamps: 0/24, kw: 2
: user-supplied epsilon_k = 0
: epsilon_k by k-weight = 1.320e-03
: epsilon_r by k-weight = 2.401e-01
: R-factor by k-weight = 1 -> 0.00580, 2 -> 0.00614, 3 -> 0.00931

name	N	S02	sigma^2	e0	delr	Reff	R
[Co_absorber_Al_scatterer] Al1.1	1.000	0.749	0.00654	7.845	-0.01088	2.55270	2.54182
[Co_absorber_Co_scatterer] Co1.1	1.000	8.611	0.00837	7.845	-0.03793	2.55270	2.51477
[Co_absorber_Co_scatterer] Co1.2	6.000	0.780	0.01145	7.845	-0.00477	3.61000	3.60523
[Co_absorber_Co_scatterer] Co1.1 Co1.1	48.000	0.780	0.01038	7.845	-0.05348	3.82900	3.77552
[Co_absorber_Co_scatterer] Co1.1 Co1.1	24.000	0.780	0.01153	7.845	-0.05348	4.35770	4.30422
[Co_absorber_Co_scatterer] Co1.1 Co1.2	48.000	0.780	0.01153	7.845	-0.02135	4.35770	4.33635
[Co_absorber_Co_scatterer] Co1.3	24.000	0.780	0.01181	7.845	-0.01398	4.42130	4.40732
[Co_absorber_Co_scatterer] Co1.1 Co1.1	48.000	0.780	0.01185	7.845	-0.07585	4.76330	4.68745
[Co_absorber_Co_scatterer] Co1.1 Co1.3	96.000	0.780	0.01185	7.845	-0.02595	4.76330	4.73735
[Co_absorber_Co_scatterer] Co1.4	12.000	0.780	0.01197	7.845	-0.00946	5.10530	5.09584
[Co_absorber_Co_scatterer] Co1.1 Co1.1	12.000	0.780	0.01197	7.845	-0.07585	5.10530	5.02945
[Co_absorber_Co_scatterer] Co1.1 Co1.4	24.000	0.780	0.01197	7.845	-0.00946	5.10530	5.09584

[Co_absorber_Co_scatterer] Co1.1 12.000 0.780 0.01197 7.845 -0.07585 5.10530 5.02945
[Co_absorber_Co_scatterer] Co1.1 Co1.4 Co1.1 12.000 0.780 0.01197 7.845 -0.00946 5.10530 5.09584

Al-K; Ni-K

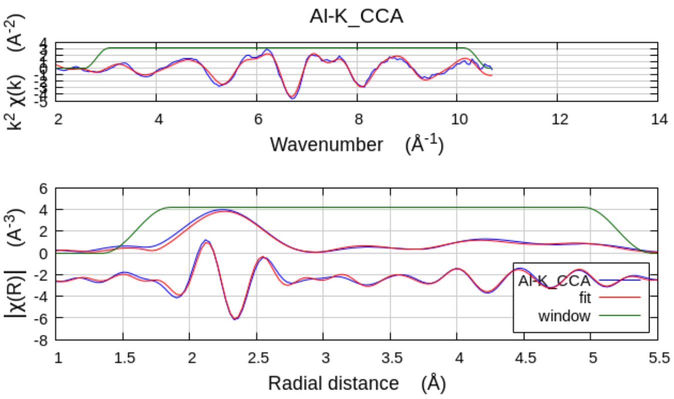

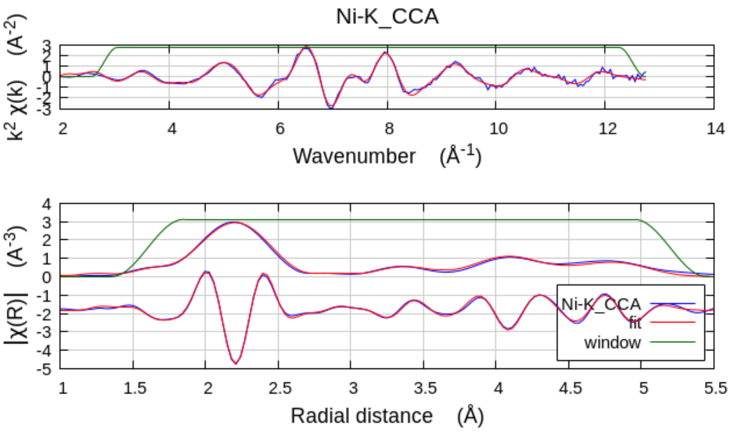

Independent points : 38.9687500
Number of variables : 15
Chi-square : 2234.7311059
Reduced chi-square : 93.2351961
R-factor : 0.0180368
Number of data sets : 2

guess parameters:
x = 1.80738472 # +/- 0.39148667 [2.03383]
dEnot_Ni = 6.21741545 # +/- 0.93963742 [6.21845]
dEnot_Al = 6.79955609 # +/- 0.85371553 [6.80783]
dAl1st = -0.01310168 # +/- 0.00600531 [-0.01303]
dAl2nd = -0.03027684 # +/- 0.02814877 [-0.03022]
dAl3rd = -0.01547172 # +/- 0.01809157 [-0.01537]
dAl4th = 0.02574863 # +/- 0.02184282 [0.02583]
ssNiAl = 0.00547834 # +/- 0.00040751 [0.00548]
ssNiCo = 0.00733449 # +/- 0.00066392 [0.00767]
thetaNi = 314.01783546 # +/- 12.38620372 [304.30042]
thetaAl = 423.00001611 # +/- 24.68306068 [423.00292]
dNi1st = -0.02654540 # +/- 0.00622441 [-0.02710]
dNi2nd = -0.01126914 # +/- 0.02394256 [-0.00829]

```
  dNi3rd          =  -0.01310555    # +/-   0.01597529     [-0.01178]
  dNi4th          =  -0.01868458    # +/-   0.01825426     [-0.01845]

set parameters:
  amp_Ni          =   0.77000000
  amp_Al          =   0.66000000
  temperature     = 300.00000000

: name                  = Al-K_CCA
: k-range               = 2.8 - 10.4
: dk                    = 0.5
: k-window              = Hanning
: k-weight              = 1,2,3
: R-range               = 1.6 - 5.2
: dR                    = 0.5
: R-window              = Hanning
: fitting space         = r
: background function   = no
: phase correction      = no
: background removal    = E0: 1557.237901, Rbkg: 1.0, range: [0:10.774], clamps: 0/24, kw: 2
: user-supplied epsilon_k = 0
: epsilon_k by k-weight   = 2.336e-03
: epsilon_r by k-weight   = 3.157e-01
: R-factor by k-weight    = 1 -> 0.03309,  2 -> 0.02729,  3 -> 0.02940

  name                                 N      S02     sigma^2   e0     delr     Reff     R
=======================================================================================
[Al_absorber_Co_scatterer] Co1.1               12.000   0.660   0.00548   6.800 -0.01310  2.55270  2.53960
[Al_absorber_Co_scatterer] Co1.2                6.000   0.660   0.01117   6.800 -0.03028  3.61000  3.57972
[Al_absorber_Co_scatterer] Co1.1 Co1.1         48.000   0.660   0.00902   6.800 -0.01834  3.82900  3.81066
[Al_absorber_Co_scatterer] Co1.1 Co1.1         24.000   0.660   0.00878   6.800 -0.01834  4.35770  4.33936
[Al_absorber_Co_scatterer] Co1.1 Co1.2         48.000   0.660   0.01045   6.800 -0.02169  4.35770  4.33601
[Al_absorber_Co_scatterer] Co1.3               24.000   0.660   0.01146   6.800 -0.01547  4.42130  4.40583
[Al_absorber_Co_scatterer] Co1.1 Co1.1         48.000   0.660   0.00803   6.800 -0.02620  4.76330  4.73710
[Al_absorber_Co_scatterer] Co1.1 Co1.3         96.000   0.660   0.01111   6.800 -0.01429  4.76330  4.74901
[Al_absorber_Co_scatterer] Co1.4               12.000   0.660   0.01159   6.800  0.02575  5.10530  5.13105
[Al_absorber_Co_scatterer] Co1.1 Co1.1         12.000   0.660   0.00717   6.800 -0.02620  5.10530  5.07910
[Al_absorber_Co_scatterer] Co1.1 Co1.4         24.000   0.660   0.01159   6.800  0.02575  5.10530  5.13105
[Al_absorber_Co_scatterer] Co1.1               12.000   0.660   0.00717   6.800 -0.02620  5.10530  5.07910
[Al_absorber_Co_scatterer] Co1.1 Co1.4 Co1.1   12.000   0.660   0.01159   6.800  0.02575  5.10530  5.13105

: name                  = Ni-K_CCA
: k-range               = 2.8 - 12.5
: dk                    = 0.5
: k-window              = Hanning
: k-weight              = 1,2,3
: R-range               = 1.6 - 5.2
: dR                    = 0.5
: R-window              = Hanning
: fitting space         = r
: background function   = no
: phase correction      = no
: background removal    = E0: 8333, Rbkg: 1.0, range: [0:12.817], clamps: 0/24, kw: 2
: user-supplied epsilon_k = 0
: epsilon_k by k-weight   = 1.459e-03
: epsilon_r by k-weight   = 3.640e-01
```

: R-factor by k-weight = 1 -> 0.00535, 2 -> 0.00530, 3 -> 0.00779

name	N	S02	sigma^2	e0	delr	Reff	R
[Ni_absorber_Co_scatterer] Co1.1	1.000	7.848	0.00733	6.217	-0.02655	2.55270	2.52615
[Ni_absorber_Co_scatterer] Co1.2	6.000	0.770	0.01212	6.217	-0.01127	3.61000	3.59873
[Ni_absorber_Co_scatterer] Co1.1 Co1.1	48.000	0.770	0.01096	6.217	-0.03716	3.82900	3.79184
[Ni_absorber_Co_scatterer] Co1.1 Co1.1	24.000	0.770	0.01217	6.217	-0.03716	4.35770	4.32054
[Ni_absorber_Co_scatterer] Co1.1 Co1.2	48.000	0.770	0.01218	6.217	-0.01891	4.35770	4.33879
[Ni_absorber_Co_scatterer] Co1.3	24.000	0.770	0.01251	6.217	-0.01311	4.42130	4.40819
[Ni_absorber_Co_scatterer] Co1.1 Co1.1	48.000	0.770	0.01252	6.217	-0.05309	4.76330	4.71021
[Ni_absorber_Co_scatterer] Co1.1 Co1.3	96.000	0.770	0.01254	6.217	-0.01983	4.76330	4.74347
[Ni_absorber_Co_scatterer] Co1.4	12.000	0.770	0.01267	6.217	-0.01868	5.10530	5.08662
[Ni_absorber_Co_scatterer] Co1.1 Co1.1	12.000	0.770	0.01265	6.217	-0.05309	5.10530	5.05221
[Ni_absorber_Co_scatterer] Co1.1 Co1.4	24.000	0.770	0.01267	6.217	-0.01868	5.10530	5.08662
[Ni_absorber_Co_scatterer] Co1.1	12.000	0.770	0.01265	6.217	-0.05309	5.10530	5.05221
[Ni_absorber_Co_scatterer] Co1.1 Co1.4 Co1.1	12.000	0.770	0.01267	6.217	-0.01868	5.10530	5.08662
[Ni_absorber_Al_scatterer] Al1.1	1.000	1.392	0.00548	6.217	-0.01310	2.55270	2.53960

Al-K; Cu-K

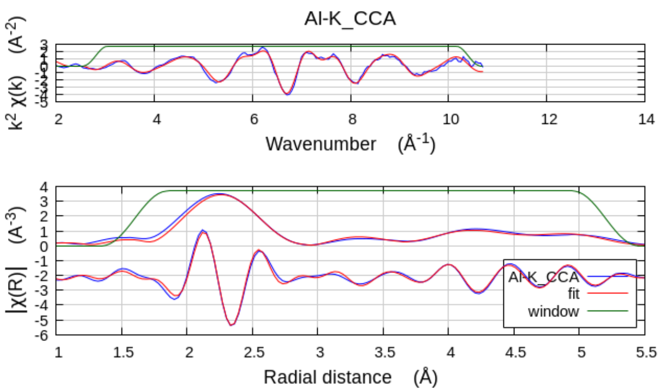

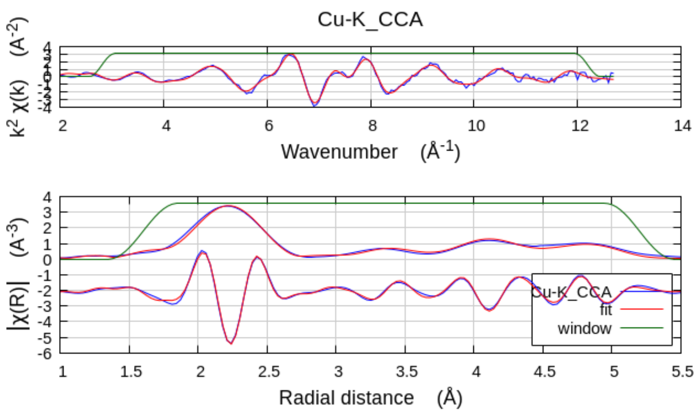

Independent points : 38.2890625
Number of variables : 15
Chi-square : 1628.8257089
Reduced chi-square : 69.9395138
R-factor : 0.0149936
Number of data sets : 2

guess parameters:

```
  dEnot_Al    =    6.91984573   # +/-   0.69477410    [6.92322]
  dEnot_Cu    =    7.38429019   # +/-   0.91436550    [7.38339]
  thetaAl     =  396.69322506   # +/-  17.96752709    [396.81045]
  thetaCu     =  324.83149520   # +/-  13.00601448    [324.83080]
  ssCuCo      =    0.00675092   # +/-   0.00060938    [0.00675]
  ssCuAl      =    0.00654060   # +/-   0.00035652    [0.00654]
  dRAl1st     =   -0.01114893   # +/-   0.00510647    [-0.01155]
  dRAl2nd     =   -0.02850182   # +/-   0.02475032    [-0.02829]
```

```
  dRAl3rd        =  -0.01339133    # +/-   0.01584791     [-0.01317]
  dRAl4th        =   0.02792113    # +/-   0.01938862     [0.02856]
  dRCu1st        =  -0.01035696    # +/-   0.00611100     [-0.01036]
  dRCu2nd        =  -0.01325493    # +/-   0.02236709     [-0.01327]
  dRCu3rd        =   0.00228766    # +/-   0.01515705     [0.00228]
  dRCu4th        =  -0.00526301    # +/-   0.01676160     [-0.00527]
  x              =   1.55339564    # +/-   0.42301977     [1.55306]

set parameters:
  amp_Al         =   0.66000000
  amp_Cu         =   0.79000000
  temperature    = 300.00000000

: name                   = Al-K_CCA
: k-range                = 2.8 - 10.4
: dk                     = 0.5
: k-window               = Hanning
: k-weight               = 1,2,3
: R-range                = 1.6 - 5.2
: dR                     = 0.5
: R-window               = Hanning
: fitting space          = r
: background function    = no
: phase correction       = no
: background removal     = E0: 1557.237901, Rbkg: 1.0, range: [0:10.774], clamps: 0/24, kw: 2
: user-supplied epsilon_k = 0
: epsilon_k by k-weight  = 2.047e-03
: epsilon_r by k-weight  = 2.765e-01
: R-factor by k-weight   = 1 -> 0.02115,  2 -> 0.01672,  3 -> 0.01836

  name                                    N      S02    sigma^2   e0     delr     Reff     R
===========================================================================================
[Al_absorber_Co_scatterer] Co1.1                12.000   0.660   0.00654   6.920 -0.01115  2.55270  2.54155
[Al_absorber_Co_scatterer] Co1.2                 6.000   0.660   0.01260   6.920 -0.02850  3.61000  3.58150
[Al_absorber_Co_scatterer] Co1.1 Co1.1          48.000   0.660   0.01017   6.920 -0.01572  3.82900  3.81328
[Al_absorber_Co_scatterer] Co1.1 Co1.1          24.000   0.660   0.00989   6.920 -0.01572  4.35770  4.34198
[Al_absorber_Co_scatterer] Co1.1 Co1.2          48.000   0.660   0.01178   6.920 -0.01982  4.35770  4.33788
[Al_absorber_Co_scatterer] Co1.3                24.000   0.660   0.01294   6.920 -0.01339  4.42130  4.40791
[Al_absorber_Co_scatterer] Co1.1 Co1.1          48.000   0.660   0.00906   6.920 -0.02230  4.76330  4.74100
[Al_absorber_Co_scatterer] Co1.1 Co1.3          96.000   0.660   0.01254   6.920 -0.01227  4.76330  4.75103
[Al_absorber_Co_scatterer] Co1.4                12.000   0.660   0.01308   6.920  0.02792  5.10530  5.13322
[Al_absorber_Co_scatterer] Co1.1 Co1.1          12.000   0.660   0.00809   6.920 -0.02230  5.10530  5.08300
[Al_absorber_Co_scatterer] Co1.1 Co1.4          24.000   0.660   0.01308   6.920  0.02792  5.10530  5.13322
[Al_absorber_Co_scatterer] Co1.1                12.000   0.660   0.00809   6.920 -0.02230  5.10530  5.08300
[Al_absorber_Co_scatterer] Co1.1 Co1.4 Co1.1    12.000   0.660   0.01308   6.920  0.02792  5.10530  5.13322


: name                   = Cu-K_CCA
: k-range                = 2.8 - 12.2
: dk                     = 0.5
: k-window               = Hanning
: k-weight               = 1,2,3
: R-range                = 1.6 - 5.2
: dR                     = 0.5
: R-window               = Hanning
: fitting space          = r
```

: background function   = no
: phase correction   = no
: background removal   = E0: 8979.351385, Rbkg: 1.0, range: [0:12.783], clamps: 0/24, kw: 2
: user-supplied epsilon_k = 0
: epsilon_k by k-weight   = 1.962e-03
: epsilon_r by k-weight   = 4.828e-01
: R-factor by k-weight   = 1 -> 0.00743, 2 -> 0.00980, 3 -> 0.01650

name	N	S02	sigma^2	e0	delr	Reff	R
[Cu_absorber_Co_scatterer] Co1.1	1.000	8.253	0.00675	7.384	-0.01036	2.55270	2.54234
[Cu_absorber_Co_scatterer] Co1.2	6.000	0.790	0.01092	7.384	-0.01325	3.61000	3.59674
[Cu_absorber_Co_scatterer] Co1.1 Co1.1	48.000	0.790	0.01003	7.384	-0.01460	3.82900	3.81440
[Cu_absorber_Co_scatterer] Co1.1 Co1.1	24.000	0.790	0.01124	7.384	-0.01460	4.35770	4.34310
[Cu_absorber_Co_scatterer] Co1.1 Co1.2	48.000	0.790	0.01108	7.384	-0.01181	4.35770	4.34589
[Cu_absorber_Co_scatterer] Co1.3	24.000	0.790	0.01127	7.384	0.00229	4.42130	4.42359
[Cu_absorber_Co_scatterer] Co1.1 Co1.1	48.000	0.790	0.01165	7.384	-0.02071	4.76330	4.74259
[Cu_absorber_Co_scatterer] Co1.1 Co1.3	96.000	0.790	0.01135	7.384	-0.00404	4.76330	4.75927
[Cu_absorber_Co_scatterer] Co1.4	12.000	0.790	0.01142	7.384	-0.00526	5.10530	5.10004
[Cu_absorber_Co_scatterer] Co1.1 Co1.1	12.000	0.790	0.01185	7.384	-0.02071	5.10530	5.08459
[Cu_absorber_Co_scatterer] Co1.1 Co1.4	24.000	0.790	0.01142	7.384	-0.00526	5.10530	5.10004
[Cu_absorber_Co_scatterer] Co1.1	12.000	0.790	0.01185	7.384	-0.02071	5.10530	5.08459
[Cu_absorber_Co_scatterer] Co1.1 Co1.4 Co1.1	12.000	0.790	0.01142	7.384	-0.00526	5.10530	5.10004
[Cu_absorber_Al_scatterer] Al1.1	1.000	1.227	0.00654	7.384	-0.01115	2.55270	2.54155

CCA$_{sans}$Al

Cr-K

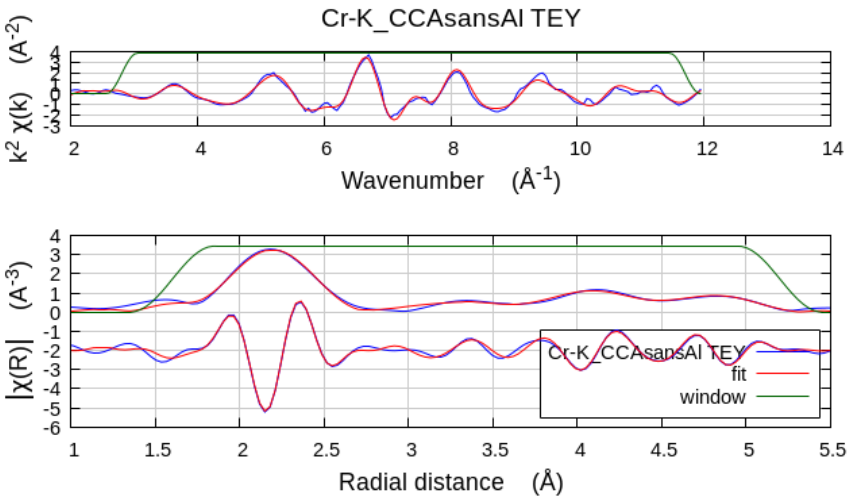

Independent points   : 20.0507813
Number of variables   : 8
Chi-square   : 1666.0508982
Reduced chi-square   : 138.2525219
R-factor   : 0.0152062
Number of data sets   : 1

guess parameters:

```
  amp_Cr          =   0.56961874    # +/-   0.04832130     [0.56962]
  dEnot_Cr        =   7.81218579    # +/-   0.72561857     [7.81206]
  ss0_CrGrey      =   0.00642893    # +/-   0.00075077     [0.00643]
  dR1st           =  -0.03119621    # +/-   0.00507450     [-0.03120]
  dR2nd           =  -0.04856271    # +/-   0.01971151     [-0.04857]
  dR3rd           =  -0.03343394    # +/-   0.01247616     [-0.03344]
  dR4th           =  -0.02070580    # +/-   0.01391319     [-0.02071]
  thetaCr         = 364.28925057    # +/-  20.02072651     [364.28661]

set parameters:
  temperature     = 300.00000000

: name                 = Cr-K_CCAsansAl TEY
: k-range              = 2.8 - 11.7
: dk                   = 0.5
: k-window             = Hanning
: k-weight             = 1,2,3
: R-range              = 1.6 - 5.2
: dR                   = 0.5
: R-window             = Hanning
: fitting space        = r
: background function  = no
: phase correction     = no
: background removal   = E0: 5990.26737660909, Rbkg: 1.0, range: [0:15.019], clamps: 0/24, kw: 2
: user-supplied epsilon_k = 0
: epsilon_k by k-weight   = 8.282e-04
: epsilon_r by k-weight   = 1.647e-01
: R-factor by k-weight    = 1 -> 0.01048,  2 -> 0.01332,  3 -> 0.02182

  name                                      N      S02    sigma^2   e0     delr     Reff     R
===============================================================================
[Cr_absorber_Co_scatterer] Co1.1               12.000   0.570   0.00643   7.812 -0.03120  2.55270  2.52150
[Cr_absorber_Co_scatterer] Co1.2                6.000   0.570   0.00973   7.812 -0.04856  3.61000  3.56144
[Cr_absorber_Co_scatterer] Co1.1 Co1.1         48.000   0.570   0.00866   7.812 -0.04399  3.82900  3.78501
[Cr_absorber_Co_scatterer] Co1.1 Co1.1         24.000   0.570   0.00944   7.812 -0.04399  4.35770  4.31371
[Cr_absorber_Co_scatterer] Co1.1 Co1.2         48.000   0.570   0.00968   7.812 -0.03988  4.35770  4.31782
[Cr_absorber_Co_scatterer] Co1.3               24.000   0.570   0.01002   7.812 -0.03343  4.42130  4.38787
[Cr_absorber_Co_scatterer] Co1.1 Co1.1         48.000   0.570   0.00956   7.812 -0.06239  4.76330  4.70091
[Cr_absorber_Co_scatterer] Co1.1 Co1.3         96.000   0.570   0.01000   7.812 -0.03232  4.76330  4.73099
[Cr_absorber_Co_scatterer] Co1.4               12.000   0.570   0.01015   7.812 -0.02071  5.10530  5.08459
[Cr_absorber_Co_scatterer] Co1.1 Co1.1         12.000   0.570   0.00951   7.812 -0.06239  5.10530  5.04291
[Cr_absorber_Co_scatterer] Co1.1 Co1.4         24.000   0.570   0.01015   7.812 -0.02071  5.10530  5.08459
[Cr_absorber_Co_scatterer] Co1.1               12.000   0.570   0.00951   7.812 -0.06239  5.10530  5.04291
[Cr_absorber_Co_scatterer] Co1.1 Co1.4 Co1.1   12.000   0.570   0.01015   7.812 -0.02071  5.10530  5.08459
```

Fe-K

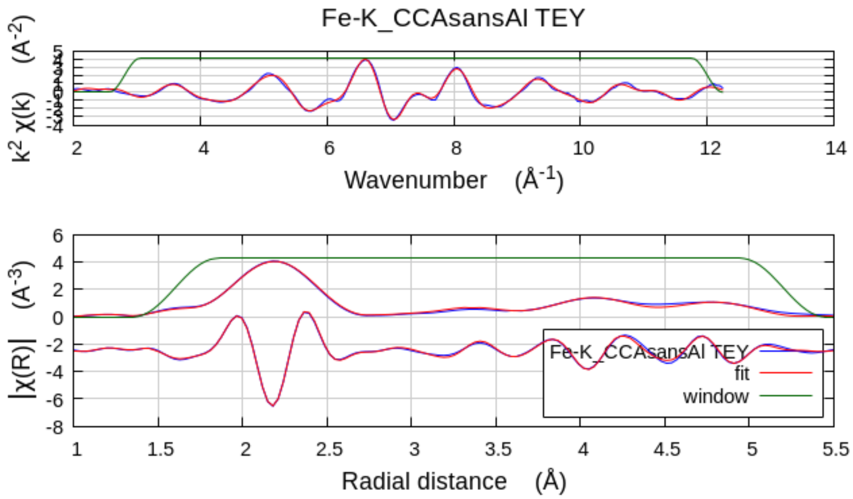

Independent points : 20.7304688
Number of variables : 8
Chi-square : 4314.5350339
Reduced chi-square : 338.9140745
R-factor : 0.0052889
Number of data sets : 1

guess parameters:
amp_Fe = 0.74889066 # +/- 0.03565545 [0.58435]
dEnot_Fe = 7.35776386 # +/- 0.39117987 [7.45879]
ss0_FeGrey = 0.00700609 # +/- 0.00044646 [0.00626]
dR1st = -0.03491907 # +/- 0.00291731 [-0.03542]
dR2nd = -0.04121670 # +/- 0.01154815 [-0.04283]
dR3rd = -0.03662497 # +/- 0.00714011 [-0.03747]
dR4th = -0.03053855 # +/- 0.00809683 [-0.03508]
thetaFe = 349.16346977 # +/- 10.52783855 [370.47980]

set parameters:
temperature = 300.00000000

: name = Fe-K_CCAsansAl TEY
: k-range = 2.8 - 12
: dk = 0.5
: k-window = Hanning
: k-weight = 1,2,3
: R-range = 1.6 - 5.2
: dR = 0.5
: R-window = Hanning
: fitting space = r
: background function = no
: phase correction = no
: background removal = E0: 7113.01287878116, Rbkg: 1.0, range: [0:12.338217430407], clamps: 0/24, kw: 2
: user-supplied epsilon_k = 0
: epsilon_k by k-weight = 5.147e-04
: epsilon_r by k-weight = 1.117e-01
: R-factor by k-weight = 1 -> 0.00515, 2 -> 0.00474, 3 -> 0.00598

name	N	S02	sigma^2	e0	delr	Reff	R
[Fe_absorber_Co_scatterer] Co1.1	12.000	0.749	0.00701	7.358	-0.03492	2.55270	2.51778
[Fe_absorber_Co_scatterer] Co1.2	6.000	0.749	0.01015	7.358	-0.04122	3.61000	3.56878
[Fe_absorber_Co_scatterer] Co1.1 Co1.1	48.000	0.749	0.00914	7.358	-0.04924	3.82900	3.77976
[Fe_absorber_Co_scatterer] Co1.1 Co1.1	24.000	0.749	0.01006	7.358	-0.04924	4.35770	4.30846
[Fe_absorber_Co_scatterer] Co1.1 Co1.2	48.000	0.749	0.01017	7.358	-0.03807	4.35770	4.31963
[Fe_absorber_Co_scatterer] Co1.3	24.000	0.749	0.01047	7.358	-0.03662	4.42130	4.38467
[Fe_absorber_Co_scatterer] Co1.1 Co1.1	48.000	0.749	0.01028	7.358	-0.06984	4.76330	4.69346
[Fe_absorber_Co_scatterer] Co1.1 Co1.3	96.000	0.749	0.01048	7.358	-0.03577	4.76330	4.72753
[Fe_absorber_Co_scatterer] Co1.4	12.000	0.749	0.01060	7.358	-0.03054	5.10530	5.07476
[Fe_absorber_Co_scatterer] Co1.1 Co1.1	12.000	0.749	0.01031	7.358	-0.06984	5.10530	5.03546
[Fe_absorber_Co_scatterer] Co1.1 Co1.4	24.000	0.749	0.01060	7.358	-0.03054	5.10530	5.07476
[Fe_absorber_Co_scatterer] Co1.1	12.000	0.749	0.01031	7.358	-0.06984	5.10530	5.03546
[Fe_absorber_Co_scatterer] Co1.1 Co1.4 Co1.1	12.000	0.749	0.01060	7.358	-0.03054	5.10530	5.07476

Co-K

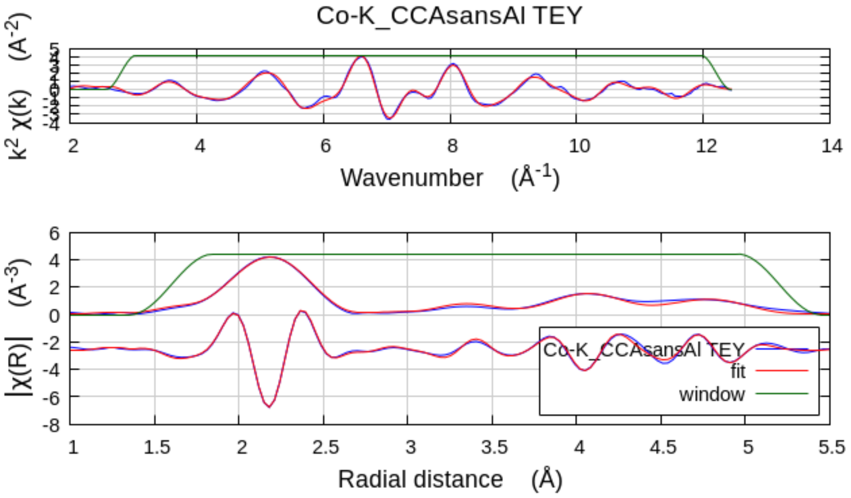

Independent points : 21.1835938
Number of variables : 8
Chi-square : 6820.6730198
Reduced chi-square : 517.3606794
R-factor : 0.0076576
Number of data sets : 1

guess parameters:
amp_Co = 0.77665745 # +/- 0.04170229 [0.77666]
dEnot_Co = 7.44529754 # +/- 0.44086633 [7.44529]
dR1st = -0.04544467 # +/- 0.00337222 [-0.04544]
dR2nd = -0.04991606 # +/- 0.01286767 [-0.04991]

```
  dR3rd            =  -0.03929737    # +/-   0.00792083     [-0.03930]
  dR4th            =  -0.03379011    # +/-   0.00909889     [-0.03379]
  thetaCo          = 349.90909915    # +/-  12.12034612     [349.90915]
  ss0_CoGrey       =   0.00716845    # +/-   0.00051409     [0.00717]

set parameters:
  temperature      = 300.00000000

: name                    = Co-K_CCAsansAl TEY
: k-range                 = 2.8 - 12.2
: dk                      = 0.5
: k-window                = Hanning
: k-weight                = 1,2,3
: R-range                 = 1.6 - 5.2
: dR                      = 0.5
: R-window                = Hanning
: fitting space           = r
: background function     = no
: phase correction        = no
: background removal      = E0: 7710.86380749016, Rbkg: 1.0, range: [0.000:12.338217430407], clamps: 0/24, kw: 2
: user-supplied epsilon_k = 0
: epsilon_k by k-weight   = 4.715e-04
: epsilon_r by k-weight   = 1.082e-01
: R-factor by k-weight    = 1 -> 0.00663,  2 -> 0.00684,  3 -> 0.00950
```

name	N	S02	sigma^2	e0	delr	Reff	R
[Co_absorber_Co_scatterer] Co1.1	12.000	0.777	0.00717	7.445	-0.04544	2.55270	2.50726
[Co_absorber_Co_scatterer] Co1.2	6.000	0.777	0.00984	7.445	-0.04992	3.61000	3.56008
[Co_absorber_Co_scatterer] Co1.1 Co1.1	48.000	0.777	0.00893	7.445	-0.06408	3.82900	3.76492
[Co_absorber_Co_scatterer] Co1.1 Co1.1	24.000	0.777	0.00991	7.445	-0.06408	4.35770	4.29362
[Co_absorber_Co_scatterer] Co1.1 Co1.2	48.000	0.777	0.00991	7.445	-0.04768	4.35770	4.31002
[Co_absorber_Co_scatterer] Co1.3	24.000	0.777	0.01014	7.445	-0.03930	4.42130	4.38200
[Co_absorber_Co_scatterer] Co1.1 Co1.1	48.000	0.777	0.01018	7.445	-0.09089	4.76330	4.67241
[Co_absorber_Co_scatterer] Co1.1 Co1.3	96.000	0.777	0.01018	7.445	-0.04237	4.76330	4.72093
[Co_absorber_Co_scatterer] Co1.4	12.000	0.777	0.01027	7.445	-0.03379	5.10530	5.07151
[Co_absorber_Co_scatterer] Co1.1 Co1.1	12.000	0.777	0.01027	7.445	-0.09089	5.10530	5.01441
[Co_absorber_Co_scatterer] Co1.1 Co1.4	24.000	0.777	0.01027	7.445	-0.03379	5.10530	5.07151
[Co_absorber_Co_scatterer] Co1.1	12.000	0.777	0.01027	7.445	-0.09089	5.10530	5.01441
[Co_absorber_Co_scatterer] Co1.1 Co1.4 Co1.1	12.000	0.777	0.01027	7.445	-0.03379	5.10530	5.07151

Ni-K

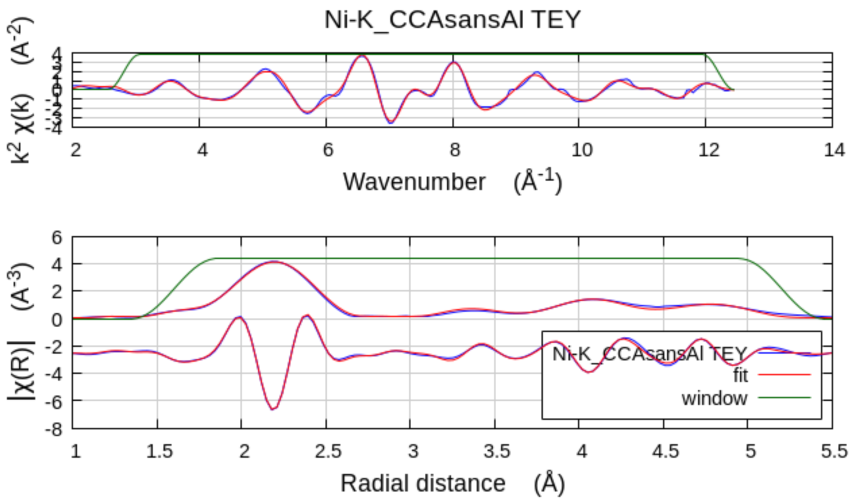

Independent points : 21.1835938
Number of variables : 8
Chi-square : 1646.7204916
Reduced chi-square : 124.9067988
R-factor : 0.0054515
Number of data sets : 1

guess parameters:
amp_Ni = 0.77337638 # +/- 0.03584924 [0.77295]
dEnot_Ni = 6.94182571 # +/- 0.38107324 [6.94130]
dR1st = -0.03813906 # +/- 0.00292007 [-0.03815]
dR2nd = -0.03027530 # +/- 0.01161816 [-0.03032]
dR3rd = -0.03686387 # +/- 0.00703263 [-0.03688]
dR4th = -0.03898673 # +/- 0.00807202 [-0.03900]
thetaNi = 343.13934584 # +/- 10.14652841 [343.22285]
ss0_NiGrey = 0.00718123 # +/- 0.00044282 [0.00717]

set parameters:
temperature = 300.00000000

: name = Ni-K_CCAsansAl TEY
: k-range = 2.8 - 12.2
: dk = 0.5
: k-window = Hanning
: k-weight = 1,2,3
: R-range = 1.6 - 5.2
: dR = 0.5
: R-window = Hanning
: fitting space = r
: background function = no
: phase correction = no
: background removal = E0: 8333.45814688946, Rbkg: 1.0, range: [0.000:12.338217430407], clamps: 0/24, kw: 2
: user-supplied epsilon_k = 0
: epsilon_k by k-weight = 8.211e-04
: epsilon_r by k-weight = 1.885e-01
: R-factor by k-weight = 1 -> 0.00521, 2 -> 0.00493, 3 -> 0.00621

name	N	S02	sigma^2	e0	delr	Reff	R
[Ni_absorber_Co_scatterer] Co1.1	12.000	0.773	0.00718	6.942	-0.03814	2.55270	2.51456
[Ni_absorber_Co_scatterer] Co1.2	6.000	0.773	0.01022	6.942	-0.03027	3.61000	3.57972
[Ni_absorber_Co_scatterer] Co1.1 Co1.1	48.000	0.773	0.00926	6.942	-0.05378	3.82900	3.77522
[Ni_absorber_Co_scatterer] Co1.1 Co1.1	24.000	0.773	0.01028	6.942	-0.05378	4.35770	4.30392
[Ni_absorber_Co_scatterer] Co1.1 Co1.2	48.000	0.773	0.01029	6.942	-0.03421	4.35770	4.32349
[Ni_absorber_Co_scatterer] Co1.3	24.000	0.773	0.01055	6.942	-0.03686	4.42130	4.38444
[Ni_absorber_Co_scatterer] Co1.1 Co1.1	48.000	0.773	0.01056	6.942	-0.07628	4.76330	4.68702
[Ni_absorber_Co_scatterer] Co1.1 Co1.3	96.000	0.773	0.01058	6.942	-0.03750	4.76330	4.72580
[Ni_absorber_Co_scatterer] Co1.4	12.000	0.773	0.01068	6.942	-0.03899	5.10530	5.06631
[Ni_absorber_Co_scatterer] Co1.1 Co1.1	12.000	0.773	0.01066	6.942	-0.07628	5.10530	5.02902
[Ni_absorber_Co_scatterer] Co1.1 Co1.4	24.000	0.773	0.01068	6.942	-0.03899	5.10530	5.06631
[Ni_absorber_Co_scatterer] Co1.1	12.000	0.773	0.01066	6.942	-0.07628	5.10530	5.02902
[Ni_absorber_Co_scatterer] Co1.1 Co1.4 Co1.1	12.000	0.773	0.01068	6.942	-0.03899	5.10530	5.06631

Cu-K

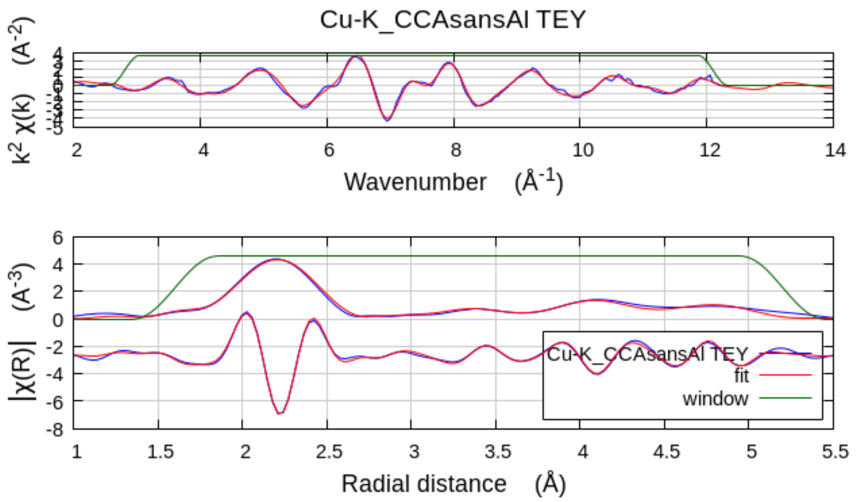

Independent points : 21.1835938
Number of variables : 8
Chi-square : 758.3219650
Reduced chi-square : 57.5201253
R-factor : 0.0084834
Number of data sets : 1

guess parameters:

```
amp_Cu       =   0.78679719   # +/-   0.04713173    [0.78680]
dEnot_Cu     =   6.79146643   # +/-   0.51719117    [6.79145]
ss0_CuGrey   =   0.00667973   # +/-   0.00053270    [0.00668]
dR1st        =  -0.02169705   # +/-   0.00367570    [-0.02170]
dR2nd        =  -0.04110870   # +/-   0.01521452    [-0.04111]
dR3rd        =  -0.01833010   # +/-   0.00983722    [-0.01833]
dR4th        =  -0.02655606   # +/-   0.01079273    [-0.02656]
thetaCu      = 332.58645544   # +/-  12.50729287    [332.58600]
```

set parameters:
  temperature = 300.00000000

: name = Cu-CCA-Al_TEY.dat
: k-range = 2.8 - 12.2
: dk = 0.5
: k-window = Hanning
: k-weight = 1,2,3
: R-range = 1.6 - 5.2
: dR = 0.5
: R-window = Hanning
: fitting space = r
: background function = no
: phase correction = no
: background removal = E0: 8983.2662897816, Rbkg: 1.0, range: [0:12.2], clamps: 0/24, kw: 2
: user-supplied epsilon_k = 0
: epsilon_k by k-weight = 3.762e-04
: epsilon_r by k-weight = 3.108e-01
: R-factor by k-weight = 1 -> 0.00958, 2 -> 0.00731, 3 -> 0.00856

name	N	S02	sigma^2	e0	delr	Reff	R
[Cu_absorber_Co_scatterer] Co1.1	12.000	0.787	0.00668	6.791	-0.02170	2.55270	2.53100
[Cu_absorber_Co_scatterer] Co1.2	6.000	0.787	0.01044	6.791	-0.04111	3.61000	3.56889
[Cu_absorber_Co_scatterer] Co1.1 Co1.1	48.000	0.787	0.00959	6.791	-0.03059	3.82900	3.79841
[Cu_absorber_Co_scatterer] Co1.1 Co1.1	24.000	0.787	0.01075	6.791	-0.03059	4.35770	4.32711
[Cu_absorber_Co_scatterer] Co1.1 Co1.2	48.000	0.787	0.01059	6.791	-0.03140	4.35770	4.32630
[Cu_absorber_Co_scatterer] Co1.3	24.000	0.787	0.01077	6.791	-0.01833	4.42130	4.40297
[Cu_absorber_Co_scatterer] Co1.1 Co1.1	48.000	0.787	0.01114	6.791	-0.02170	4.76330	4.74160
[Cu_absorber_Co_scatterer] Co1.1 Co1.3	96.000	0.787	0.01085	6.791	-0.02001	4.76330	4.74329
[Cu_absorber_Co_scatterer] Co1.4	12.000	0.787	0.01091	6.791	-0.02656	5.10530	5.07874
[Cu_absorber_Co_scatterer] Co1.1 Co1.1	12.000	0.787	0.01132	6.791	-0.04339	5.10530	5.06191
[Cu_absorber_Co_scatterer] Co1.1 Co1.4	24.000	0.787	0.01091	6.791	-0.02656	5.10530	5.07874
[Cu_absorber_Co_scatterer] Co1.1	12.000	0.787	0.01132	6.791	-0.04339	5.10530	5.06191
[Cu_absorber_Co_scatterer] Co1.1 Co1.4 Co1.1	12.000	0.787	0.01091	6.791	-0.02656	5.10530	5.07874

CCA$_{sans}$Cr

Al-K; Fe-K

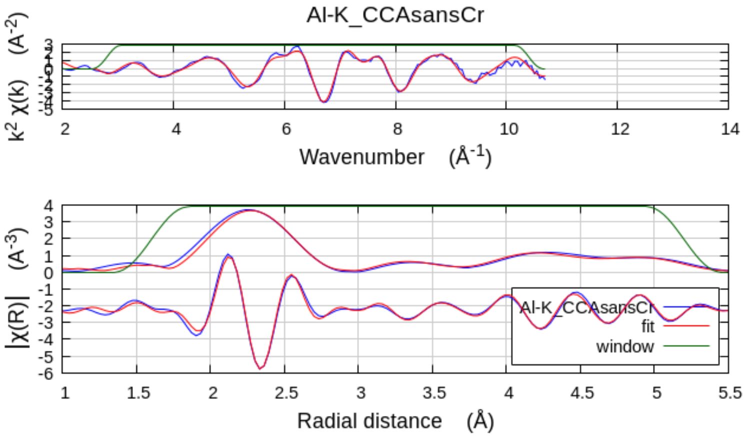

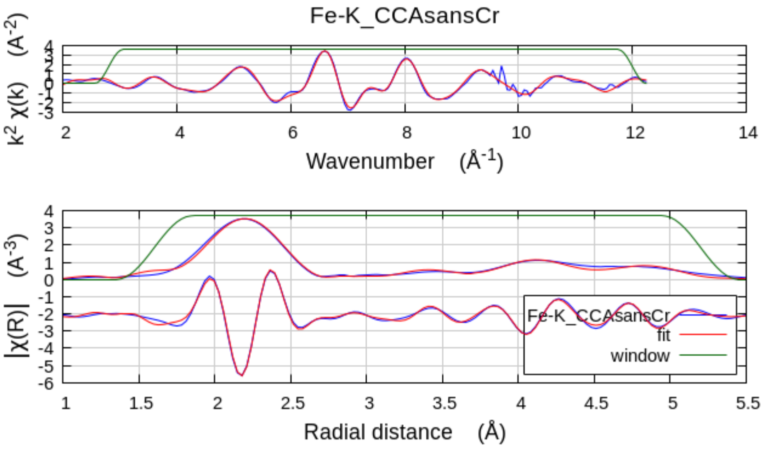

Independent points : 37.8359375
Number of variables : 15
Chi-square : 905.2306761
Reduced chi-square : 39.6406180
R-factor : 0.0129101
Number of data sets : 2

guess parameters:
dEnot_Fe = 8.28638246 # +/- 0.95490810 [8.28638]
dEnot_Al = 7.77943528 # +/- 0.57966583 [7.77944]
dRFe1st = -0.03526916 # +/- 0.00659919 [-0.03527]
dR1st = -0.00886579 # +/- 0.00429105 [-0.00887]
ssFeCo = 0.00656225 # +/- 0.00072350 [0.00656]
ssAlCo = 0.00593768 # +/- 0.00030490 [0.00594]
x = 1.24874300 # +/- 0.46943571 [1.5]
dRAl2nd = -0.01735156 # +/- 0.02099846 [-0.01735]
dRAl3rd = 0.00216151 # +/- 0.01322042 [0.00216]
dRAl4th = 0.02403366 # +/- 0.01597080 [0.02403]
thetaAl = 411.09300917 # +/- 16.39772289 [411.09301]
thetaFe = 315.39840652 # +/- 16.05225951 [315.39841]
dRFe2nd = -0.01737404 # +/- 0.03044243 [-0.01737]
dRFe3rd = -0.01434945 # +/- 0.01899996 [-0.01435]
dRFe4th = -0.00594922 # +/- 0.02244998 [-0.00595]

set parameters:
amp_Fe = 0.75000000
amp_Al = 0.66000000
temperature = 300.00000000

: name = Al-K_CCAsansCr
: k-range = 2.8 - 10.4
: dk = 0.5
: k-window = Hanning
: k-weight = 1,2,3
: R-range = 1.6 - 5.2
: dR = 0.5
: R-window = Hanning
: fitting space = r
: background function = no

```
: phase correction        = no
: background removal      = E0: 1557.228694, Rbkg: 1.0, range: [0:10.775], clamps: 0/24, kw: 2
: user-supplied epsilon_k = 0
: epsilon_k by k-weight   = 2.669e-03
: epsilon_r by k-weight   = 3.606e-01
: R-factor by k-weight    = 1 -> 0.01544,  2 -> 0.01377,  3 -> 0.01702

  name                                   N      S02     sigma^2   e0     delr     Reff     R
===============================================================================
[Al_absorber_Co_scatterer] Co1.1              12.000   0.660   0.00594   7.779 -0.00887  2.55270  2.54383
[Al_absorber_Co_scatterer] Co1.2               6.000   0.660   0.01179   7.779 -0.01735  3.61000  3.59265
[Al_absorber_Co_scatterer] Co1.1 Co1.1         48.000   0.660   0.00951   7.779 -0.01250  3.82900  3.81650
[Al_absorber_Co_scatterer] Co1.1 Co1.1         24.000   0.660   0.00925   7.779 -0.01250  4.35770  4.34520
[Al_absorber_Co_scatterer] Co1.1 Co1.2         48.000   0.660   0.01102   7.779 -0.01311  4.35770  4.34459
[Al_absorber_Co_scatterer] Co1.3              24.000   0.660   0.01209   7.779  0.00216  4.42130  4.42346
[Al_absorber_Co_scatterer] Co1.1 Co1.1         48.000   0.660   0.00847   7.779 -0.01773  4.76330  4.74557
[Al_absorber_Co_scatterer] Co1.1 Co1.3         96.000   0.660   0.01173   7.779 -0.00335  4.76330  4.75995
[Al_absorber_Co_scatterer] Co1.4              12.000   0.660   0.01223   7.779  0.02403  5.10530  5.12933
[Al_absorber_Co_scatterer] Co1.1 Co1.1         12.000   0.660   0.00756   7.779 -0.01773  5.10530  5.08757
[Al_absorber_Co_scatterer] Co1.1 Co1.4         24.000   0.660   0.01223   7.779  0.02403  5.10530  5.12933
[Al_absorber_Co_scatterer] Co1.1              12.000   0.660   0.00756   7.779 -0.01773  5.10530  5.08757
[Al_absorber_Co_scatterer] Co1.1 Co1.4 Co1.1     12.000   0.660   0.01223   7.779  0.02403  5.10530  5.12933

: name                   = Fe-K_CCAsansCr
: k-range                = 2.8 - 12
: dk                     = 0.5
: k-window               = Hanning
: k-weight               = 1,2,3
: R-range                = 1.6 - 5.2
: dR                     = 0.5
: R-window               = Hanning
: fitting space          = r
: background function    = no
: phase correction        = no
: background removal      = E0: 7112.54755530147, Rbkg: 1.0, range: [0:12.319], clamps: 0/24, kw: 2
: user-supplied epsilon_k = 0
: epsilon_k by k-weight   = 2.819e-03
: epsilon_r by k-weight   = 6.114e-01
: R-factor by k-weight    = 1 -> 0.00729,  2 -> 0.00917,  3 -> 0.01478

  name                                   N      S02     sigma^2   e0     delr     Reff     R
===============================================================================
[Fe_absorber_Co_scatterer] Co1.1               1.000   8.063   0.00656   8.286 -0.03527  2.55270  2.51743
[Fe_absorber_Al_scatterer] Al1.1              1.000   0.937   0.00594   8.286 -0.00887  2.55270  2.54383
[Fe_absorber_Co_scatterer] Co1.2               6.000   0.750   0.01233   8.286 -0.01737  3.61000  3.59263
[Fe_absorber_Co_scatterer] Co1.1 Co1.1         48.000   0.750   0.01108   8.286 -0.04973  3.82900  3.77927
[Fe_absorber_Co_scatterer] Co1.1 Co1.1         24.000   0.750   0.01221   8.286 -0.04973  4.35770  4.30797
[Fe_absorber_Co_scatterer] Co1.1 Co1.2         48.000   0.750   0.01234   8.286 -0.02632  4.35770  4.33138
[Fe_absorber_Co_scatterer] Co1.3              24.000   0.750   0.01272   8.286 -0.01435  4.42130  4.40695
[Fe_absorber_Co_scatterer] Co1.1 Co1.1         48.000   0.750   0.01249   8.286 -0.07054  4.76330  4.69276
[Fe_absorber_Co_scatterer] Co1.1 Co1.3         96.000   0.750   0.01273   8.286 -0.02481  4.76330  4.73849
[Fe_absorber_Co_scatterer] Co1.4              12.000   0.750   0.01289   8.286 -0.00595  5.10530  5.09935
[Fe_absorber_Co_scatterer] Co1.1 Co1.1         12.000   0.750   0.01254   8.286 -0.07054  5.10530  5.03476
[Fe_absorber_Co_scatterer] Co1.1 Co1.4         24.000   0.750   0.01289   8.286 -0.00595  5.10530  5.09935
[Fe_absorber_Co_scatterer] Co1.1              12.000   0.750   0.01254   8.286 -0.07054  5.10530  5.03476
[Fe_absorber_Co_scatterer] Co1.1 Co1.4 Co1.1     12.000   0.750   0.01289   8.286 -0.00595  5.10530  5.09935
```

Al-K; Co-K

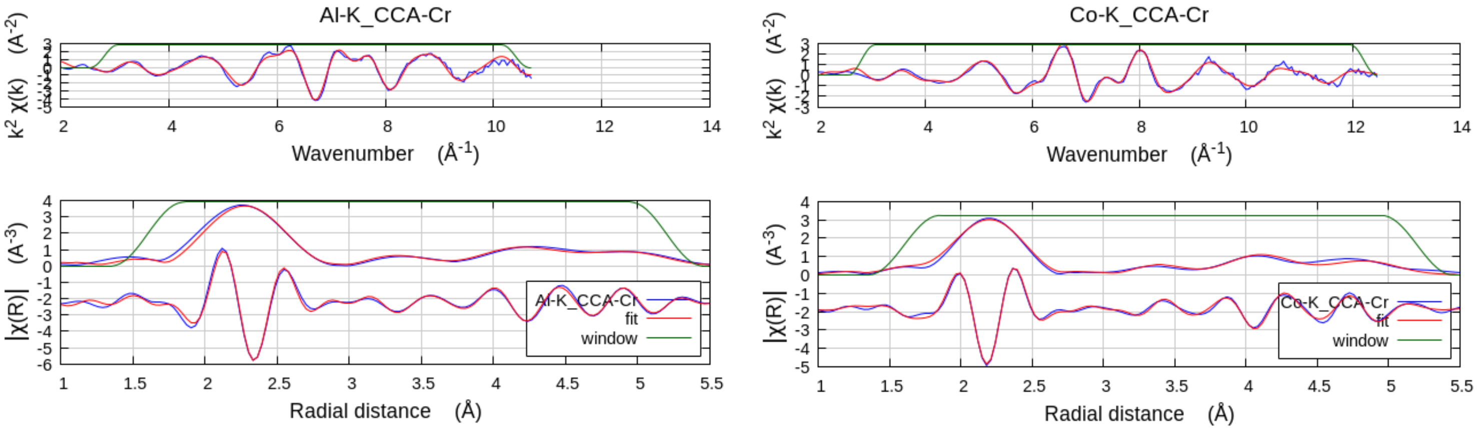

Independent points : 38.2890625
Number of variables : 15
Chi-square : 1444.1019506
Reduced chi-square : 62.0077322
R-factor : 0.0158785
Number of data sets : 2

guess parameters:
dEnot_Co = 7.85242586 # +/- 0.73807243 [7.81211]
dEnot_Al = 7.85609079 # +/- 0.71889875 [7.86601]
dR1st = -0.00818353 # +/- 0.00530608 [-0.00810]
dRCo1st = -0.04068317 # +/- 0.00466839 [-0.04092]
ssAlCo = 0.00594086 # +/- 0.00038169 [0.00594]
ssCoCo = 0.00667874 # +/- 0.00052387 [0.00677]
x = 2.23223951 # +/- 0.30700947 [2]
dRAl2nd = -0.01675129 # +/- 0.02625500 [-0.01667]
dRAl3rd = 0.00309222 # +/- 0.01651823 [0.00321]
dRAl4th = 0.02468434 # +/- 0.01996073 [0.02477]
thetaAl = 411.10738019 # +/- 20.50320401 [411.10919]
thetaCo = 307.73683779 # +/- 10.37167655 [305.01230]
dRCo2nd = -0.01111120 # +/- 0.02059766 [-0.01050]
dRCo3rd = -0.01221863 # +/- 0.01301860 [-0.01217]
dRCo4th = -0.01518755 # +/- 0.01545143 [-0.01554]

set parameters:
amp_Co = 0.78000000
amp_Al = 0.66000000
temperature = 300.00000000
: name = Al-K_CCA-Cr
: k-range = 2.8 - 10.4
: dk = 0.5
: k-window = Hanning
: k-weight = 1,2,3
: R-range = 1.6 - 5.2
: dR = 0.5
: R-window = Hanning
: fitting space = r

```
: background function     = no
: phase correction        = no
: background removal      = E0: 1557.228694, Rbkg: 1.0, range: [0:10.775], clamps: 0/24, kw: 2
: user-supplied epsilon_k = 0
: epsilon_k by k-weight   = 2.669e-03
: epsilon_r by k-weight   = 3.606e-01
: R-factor by k-weight    = 1 -> 0.01531,  2 -> 0.01391,  3 -> 0.01729

  name                                  N      S02     sigma^2   e0     delr     Reff     R
==================================================================================
[Al_absorber_Co_scatterer] Co1.1              12.000   0.660   0.00594   7.856 -0.00818  2.55270  2.54452
[Al_absorber_Co_scatterer] Co1.2               6.000   0.660   0.01179   7.856 -0.01675  3.61000  3.59325
[Al_absorber_Co_scatterer] Co1.1 Co1.1         48.000   0.660   0.00951   7.856 -0.01154  3.82900  3.81746
[Al_absorber_Co_scatterer] Co1.1 Co1.1         24.000   0.660   0.00925   7.856 -0.01154  4.35770  4.34616
[Al_absorber_Co_scatterer] Co1.1 Co1.2         48.000   0.660   0.01102   7.856 -0.01247  4.35770  4.34523
[Al_absorber_Co_scatterer] Co1.3              24.000   0.660   0.01209   7.856  0.00309  4.42130  4.42439
[Al_absorber_Co_scatterer] Co1.1 Co1.1         48.000   0.660   0.00847   7.856 -0.01637  4.76330  4.74693
[Al_absorber_Co_scatterer] Co1.1 Co1.3         96.000   0.660   0.01173   7.856 -0.00255  4.76330  4.76075
[Al_absorber_Co_scatterer] Co1.4              12.000   0.660   0.01223   7.856  0.02468  5.10530  5.12998
[Al_absorber_Co_scatterer] Co1.1 Co1.1         12.000   0.660   0.00756   7.856 -0.01637  5.10530  5.08893
[Al_absorber_Co_scatterer] Co1.1 Co1.4         24.000   0.660   0.01223   7.856  0.02468  5.10530  5.12998
[Al_absorber_Co_scatterer] Co1.1              12.000   0.660   0.00756   7.856 -0.01637  5.10530  5.08893
[Al_absorber_Co_scatterer] Co1.1 Co1.4 Co1.1     12.000   0.660   0.01223   7.856  0.02468  5.10530  5.12998


: name                    = Co-K_CCA-Cr
: k-range                 = 2.8 - 12.2
: dk                      = 0.5
: k-window                = Hanning
: k-weight                = 1,2,3
: R-range                 = 1.6 - 5.2
: dR                      = 0.5
: R-window                = Hanning
: fitting space           = r
: background function     = no
: phase correction        = no
: background removal      = E0: 7710.80502503065, Rbkg: 1.0, range: [0:12.506], clamps: 0/24, kw: 2
: user-supplied epsilon_k = 0
: epsilon_k by k-weight   = 1.591e-03
: epsilon_r by k-weight   = 3.652e-01
: R-factor by k-weight    = 1 -> 0.01488,  2 -> 0.01361,  3 -> 0.02027

  name                                  N      S02     sigma^2   e0     delr     Reff     R
==================================================================================
[Co_absorber_Co_scatterer] Co1.1               1.000   7.619   0.00668   7.852 -0.04068  2.55270  2.51202
[Co_absorber_Al_scatterer] Al1.1               1.000   1.741   0.00594   7.852 -0.00818  2.55270  2.54452
[Co_absorber_Co_scatterer] Co1.2               6.000   0.780   0.01258   7.852 -0.01111  3.61000  3.59889
[Co_absorber_Co_scatterer] Co1.1 Co1.1         48.000   0.780   0.01140   7.852 -0.05736  3.82900  3.77164
[Co_absorber_Co_scatterer] Co1.1 Co1.1         24.000   0.780   0.01266   7.852 -0.05736  4.35770  4.30034
[Co_absorber_Co_scatterer] Co1.1 Co1.2         48.000   0.780   0.01266   7.852 -0.02590  4.35770  4.33180
[Co_absorber_Co_scatterer] Co1.3              24.000   0.780   0.01298   7.852 -0.01222  4.42130  4.40908
[Co_absorber_Co_scatterer] Co1.1 Co1.1         48.000   0.780   0.01302   7.852 -0.08137  4.76330  4.68193
[Co_absorber_Co_scatterer] Co1.1 Co1.3         96.000   0.780   0.01302   7.852 -0.01020  4.76330  4.75310
[Co_absorber_Co_scatterer] Co1.4              12.000   0.780   0.01315   7.852 -0.01519  5.10530  5.09011
[Co_absorber_Co_scatterer] Co1.1 Co1.1         12.000   0.780   0.01315   7.852 -0.08137  5.10530  5.02393
[Co_absorber_Co_scatterer] Co1.1 Co1.4         24.000   0.780   0.01315   7.852 -0.01519  5.10530  5.09011
```

[Co_absorber_Co_scatterer] Co1.1 12.000 0.780 0.01315 7.852 -0.08137 5.10530 5.02393
[Co_absorber_Co_scatterer] Co1.1 Co1.4 Co1.1 12.000 0.780 0.01315 7.852 -0.01519 5.10530 5.09011

Al-K; Ni-K

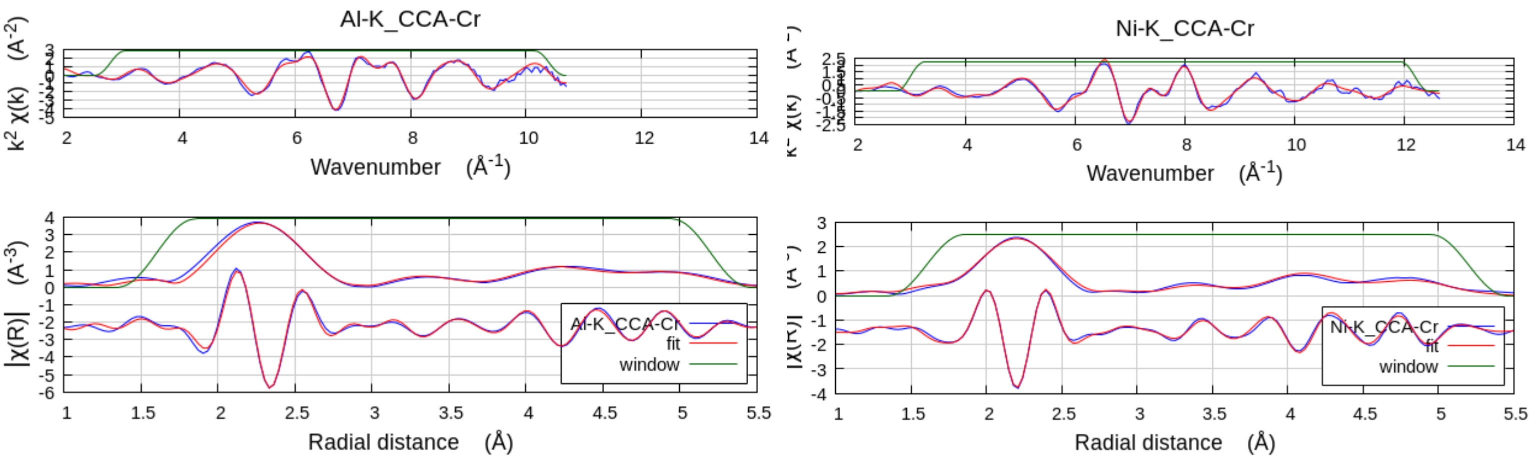

Independent points : 37.8359375
Number of variables : 15
Chi-square : 1551.3629097
Reduced chi-square : 67.9351531
R-factor : 0.0138449
Number of data sets : 2

guess parameters:
  dEnot_Ni = 6.92367883 # +/- 0.83309513 [6.92841]
  dEnot_Al = 7.80564878 # +/- 0.74944678 [7.83664]
  dRNi1st = -0.03138876 # +/- 0.00442063 [-0.03174]
  dR1st = -0.00872826 # +/- 0.00548560 [-0.00845]
  ssNiCo = 0.00720386 # +/- 0.00043503 [0.00756]
  ssAlCo = 0.00594529 # +/- 0.00039938 [0.00595]
  x = 2.97230885 # +/- 0.23130282 [3]
  dRAl2nd = -0.01695190 # +/- 0.02779133 [-0.01672]
  dRAl3rd = 0.00072995 # +/- 0.01661343 [0.00114]
  dRAl4th = 0.02249120 # +/- 0.02117662 [0.02278]
  thetaAl = 408.85344595 # +/- 21.40296367 [408.89146]
  thetaNi = 294.18365291 # +/- 7.14154682 [285.64851]
  dRNi2nd = -0.01080995 # +/- 0.01703992 [-0.00851]
  dRNi3rd = -0.01425979 # +/- 0.01253642 [-0.01241]
  dRNi4th = -0.02445593 # +/- 0.01452268 [-0.02471]

set parameters:
  amp_Ni = 0.77000000
  amp_Al = 0.66000000
  temperature = 300.00000000

: name = Al-K_CCA-Cr
: k-range = 2.8 - 10.4
: dk = 0.5
: k-window = Hanning
: k-weight = 1,2,3

: R-range = 1.6 - 5.2
: dR = 0.5
: R-window = Hanning
: fitting space = r
: background function = no
: phase correction = no
: background removal = E0: 1557.228694, Rbkg: 1.0, range: [0:10.775], clamps: 0/24, kw: 2
: user-supplied epsilon_k = 0
: epsilon_k by k-weight = 2.669e-03
: epsilon_r by k-weight = 3.606e-01
: R-factor by k-weight = 1 -> 0.01513, 2 -> 0.01336, 3 -> 0.01634

name	N	S02	sigma^2	e0	delr	Reff	R
[Al_absorber_Co_scatterer] Co1.1	12.000	0.660	0.00594	7.806	-0.00873	2.55270	2.54397
[Al_absorber_Co_scatterer] Co1.2	6.000	0.660	0.01191	7.806	-0.01695	3.61000	3.59305
[Al_absorber_Co_scatterer] Co1.1 Co1.1	48.000	0.660	0.00961	7.806	-0.01231	3.82900	3.81669
[Al_absorber_Co_scatterer] Co1.1 Co1.1	24.000	0.660	0.00935	7.806	-0.01231	4.35770	4.34539
[Al_absorber_Co_scatterer] Co1.1 Co1.2	48.000	0.660	0.01113	7.806	-0.01284	4.35770	4.34486
[Al_absorber_Co_scatterer] Co1.3	24.000	0.660	0.01222	7.806	0.00073	4.42130	4.42203
[Al_absorber_Co_scatterer] Co1.1 Co1.1	48.000	0.660	0.00856	7.806	-0.01746	4.76330	4.74584
[Al_absorber_Co_scatterer] Co1.1 Co1.3	96.000	0.660	0.01185	7.806	-0.01746	4.76330	4.74584
[Al_absorber_Co_scatterer] Co1.4	12.000	0.660	0.01236	7.806	0.02249	5.10530	5.12779
[Al_absorber_Co_scatterer] Co1.1 Co1.1	12.000	0.660	0.00764	7.806	-0.01746	5.10530	5.08784
[Al_absorber_Co_scatterer] Co1.1 Co1.4	24.000	0.660	0.01236	7.806	0.02249	5.10530	5.12779
[Al_absorber_Co_scatterer] Co1.1	12.000	0.660	0.00764	7.806	-0.01746	5.10530	5.08784
[Al_absorber_Co_scatterer] Co1.1 Co1.4 Co1.1	12.000	0.660	0.01236	7.806	0.02249	5.10530	5.12779

: name = Ni-K_CCA-Cr
: k-range = 3.000 - 12.2
: dk = 0.5
: k-window = Hanning
: k-weight = 1,2,3
: R-range = 1.6 - 5.2
: dR = 0.5
: R-window = Hanning
: fitting space = r
: background function = no
: phase correction = no
: background removal = E0: 8333.4981, Rbkg: 1.0, range: [0:12.724], clamps: 0/24, kw: 2
: user-supplied epsilon_k = 0
: epsilon_k by k-weight = 1.009e-03
: epsilon_r by k-weight = 2.450e-01
: R-factor by k-weight = 1 -> 0.01211, 2 -> 0.01082, 3 -> 0.01531

name	N	S02	sigma^2	e0	delr	Reff	R
[Ni_absorber_Al_scatterer] Al1.1	1.000	2.289	0.00594	6.924	-0.00873	2.55270	2.54397
[Ni_absorber_Co_scatterer] Co1.1	1.000	6.951	0.00720	6.924	-0.03139	2.55270	2.52131
[Ni_absorber_Co_scatterer] Co1.2	6.000	0.770	0.01374	6.924	-0.01081	3.61000	3.59919
[Ni_absorber_Co_scatterer] Co1.1 Co1.1	48.000	0.770	0.01242	6.924	-0.04426	3.82900	3.78474
[Ni_absorber_Co_scatterer] Co1.1 Co1.1	24.000	0.770	0.01380	6.924	-0.04426	4.35770	4.31344
[Ni_absorber_Co_scatterer] Co1.1 Co1.2	48.000	0.770	0.01381	6.924	-0.02110	4.35770	4.33660
[Ni_absorber_Co_scatterer] Co1.3	24.000	0.770	0.01419	6.924	-0.01426	4.42130	4.40704
[Ni_absorber_Co_scatterer] Co1.1 Co1.1	48.000	0.770	0.01420	6.924	-0.06278	4.76330	4.70052

[Ni_absorber_Co_scatterer] Co1.1 Co1.3 96.000 0.770 0.01422 6.924 -0.02282 4.76330 4.74048
[Ni_absorber_Co_scatterer] Co1.4 12.000 0.770 0.01438 6.924 -0.02446 5.10530 5.08084
[Ni_absorber_Co_scatterer] Co1.1 Co1.1 12.000 0.770 0.01435 6.924 -0.06278 5.10530 5.04252
[Ni_absorber_Co_scatterer] Co1.1 Co1.4 24.000 0.770 0.01438 6.924 -0.02446 5.10530 5.08084
[Ni_absorber_Co_scatterer] Co1.1 12.000 0.770 0.01435 6.924 -0.06278 5.10530 5.04252
[Ni_absorber_Co_scatterer] Co1.1 Co1.4 Co1.1 12.000 0.770 0.01438 6.924 -0.02446 5.10530 5.08084

Al-K; Cu-K

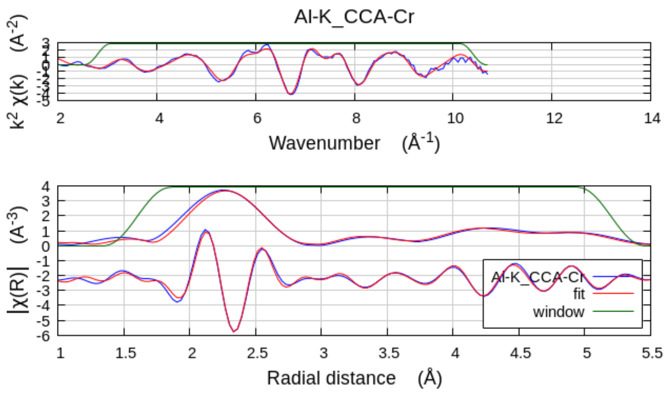

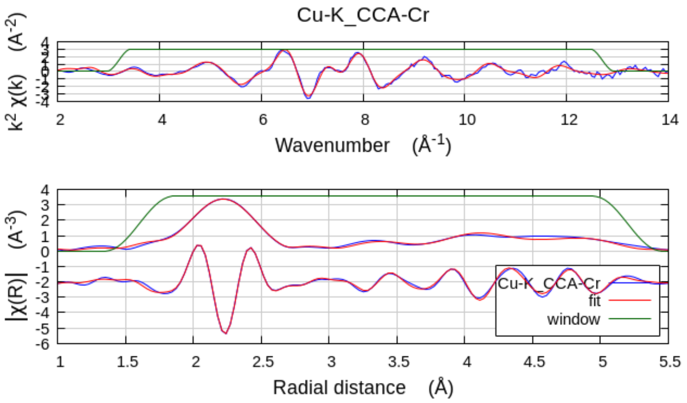

Independent points : 38.5156250
Number of variables : 15
Chi-square : 1085.6284285
Reduced chi-square : 46.1662588
R-factor : 0.0126044
Number of data sets : 2

guess parameters:
dEnot_Cu = 7.68114261 # +/- 0.94207111 [8.11302]
dEnot_Al = 7.78108301 # +/- 0.63089670 [7.83145]
dRCu1st = -0.01420102 # +/- 0.00515142 [-0.01396]
dR1st = -0.00895078 # +/- 0.00465496 [-0.00850]
ssCuCo = 0.00596794 # +/- 0.00045839 [0.00668]
ssAlCo = 0.00593537 # +/- 0.00032890 [0.00594]
x = 2.24458876 # +/- 0.31876929 [3]
dRCu2nd = -0.00600577 # +/- 0.01856195 [0.00397]
dRCu3rd = 0.00934846 # +/- 0.01370533 [0.01708]
dRCu4th = -0.00226311 # +/- 0.01531216 [0.00402]
thetaCu = 320.16616817 # +/- 9.96024038 [299.62409]
thetaAl = 408.82294567 # +/- 17.64177910 [408.88424]
dRAl2nd = -0.01712211 # +/- 0.02293646 [-0.01673]
dRAl3rd = 0.00039907 # +/- 0.01378586 [0.00107]
dRAl4th = 0.02226328 # +/- 0.01750224 [0.02272]

set parameters:
amp_Cu = 0.79000000
amp_Al = 0.66000000
temperature = 300.00000000

: name = Al-K_CCA-Cr
: k-range = 2.8 - 10.4

: dk = 0.5
: k-window = Hanning
: k-weight = 1,2,3
: R-range = 1.6 - 5.2
: dR = 0.5
: R-window = Hanning
: fitting space = r
: background function = no
: phase correction = no
: background removal = E0: 1557.228694, Rbkg: 1.0, range: [0:10.775], clamps: 0/24, kw: 2
: user-supplied epsilon_k = 0
: epsilon_k by k-weight = 2.669e-03
: epsilon_r by k-weight = 3.606e-01
: R-factor by k-weight = 1 -> 0.01513, 2 -> 0.01332, 3 -> 0.01634

name	N	S02	sigma^2	e0	delr	Reff	R
[Al_absorber_Co_scatterer] Co1.1	12.000	0.660	0.00594	7.781	-0.00895	2.55270	2.54375
[Al_absorber_Co_scatterer] Co1.2	6.000	0.660	0.01191	7.781	-0.01712	3.61000	3.59288
[Al_absorber_Co_scatterer] Co1.1 Co1.1	48.000	0.660	0.00961	7.781	-0.01262	3.82900	3.81638
[Al_absorber_Co_scatterer] Co1.1 Co1.1	24.000	0.660	0.00935	7.781	-0.01262	4.35770	4.34508
[Al_absorber_Co_scatterer] Co1.1 Co1.2	48.000	0.660	0.01113	7.781	-0.01304	4.35770	4.34466
[Al_absorber_Co_scatterer] Co1.3	24.000	0.660	0.01222	7.781	0.00040	4.42130	4.42170
[Al_absorber_Co_scatterer] Co1.1 Co1.1	48.000	0.660	0.00856	7.781	-0.01790	4.76330	4.74540
[Al_absorber_Co_scatterer] Co1.1 Co1.3	96.000	0.660	0.01185	7.781	-0.01790	4.76330	4.74540
[Al_absorber_Co_scatterer] Co1.4	12.000	0.660	0.01236	7.781	0.02226	5.10530	5.12756
[Al_absorber_Co_scatterer] Co1.1 Co1.1	12.000	0.660	0.00764	7.781	-0.01790	5.10530	5.08740
[Al_absorber_Co_scatterer] Co1.1 Co1.4	24.000	0.660	0.01236	7.781	0.02226	5.10530	5.12756
[Al_absorber_Co_scatterer] Co1.1	12.000	0.660	0.00764	7.781	-0.01790	5.10530	5.08740
[Al_absorber_Co_scatterer] Co1.1 Co1.4 Co1.1	12.000	0.660	0.01236	7.781	0.02226	5.10530	5.12756

: name = Cu-K_CCA-Cr
: k-range = 3.2 - 12.7
: dk = 0.5
: k-window = Hanning
: k-weight = 1,2,3
: R-range = 1.6 - 5.2
: dR = 0.5
: R-window = Hanning
: fitting space = r
: background function = no
: phase correction = no
: background removal = E0: 8983.44926587957, Rbkg: 1.0, range: [0:14.985], clamps: 0/24, kw: 2
: user-supplied epsilon_k = 0
: epsilon_k by k-weight = 1.838e-03
: epsilon_r by k-weight = 7.913e-01
: R-factor by k-weight = 1 -> 0.00693, 2 -> 0.00883, 3 -> 0.01508

name	N	S02	sigma^2	e0	delr	Reff	R
[Cu_absorber_Co_scatterer] Co1.1	1.000	7.707	0.00597	7.681	-0.01420	2.55270	2.53850
[Cu_absorber_Al_scatterer] Al1.1	1.000	1.773	0.00594	7.681	-0.00895	2.55270	2.54375
[Cu_absorber_Co_scatterer] Co1.2	6.000	0.790	0.01123	7.681	-0.00601	3.61000	3.60399
[Cu_absorber_Co_scatterer] Co1.1 Co1.1	48.000	0.790	0.01031	7.681	-0.02002	3.82900	3.80898
[Cu_absorber_Co_scatterer] Co1.1 Co1.1	24.000	0.790	0.01156	7.681	-0.02002	4.35770	4.33768
[Cu_absorber_Co_scatterer] Co1.1 Co1.2	48.000	0.790	0.01139	7.681	-0.01010	4.35770	4.34760

[Cu_absorber_Co_scatterer] Co1.3 24.000 0.790 0.01159 7.681 0.00935 4.42130 4.43065
[Cu_absorber_Co_scatterer] Co1.1 Co1.1 48.000 0.790 0.01198 7.681 -0.02840 4.76330 4.73490
[Cu_absorber_Co_scatterer] Co1.1 Co1.3 96.000 0.790 0.01167 7.681 -0.00243 4.76330 4.76087
[Cu_absorber_Co_scatterer] Co1.4 12.000 0.790 0.01174 7.681 -0.00226 5.10530 5.10304
[Cu_absorber_Co_scatterer] Co1.1 Co1.1 12.000 0.790 0.01218 7.681 -0.02840 5.10530 5.07690
[Cu_absorber_Co_scatterer] Co1.1 Co1.4 24.000 0.790 0.01174 7.681 -0.00226 5.10530 5.10304
[Cu_absorber_Co_scatterer] Co1.1 12.000 0.790 0.01218 7.681 -0.02840 5.10530 5.07690
[Cu_absorber_Co_scatterer] Co1.1 Co1.4 Co1.1 12.000 0.790 0.01174 7.681 -0.00226 5.10530 5.10304

CCA$_{sans}$Fe

Al-K; Cr-K

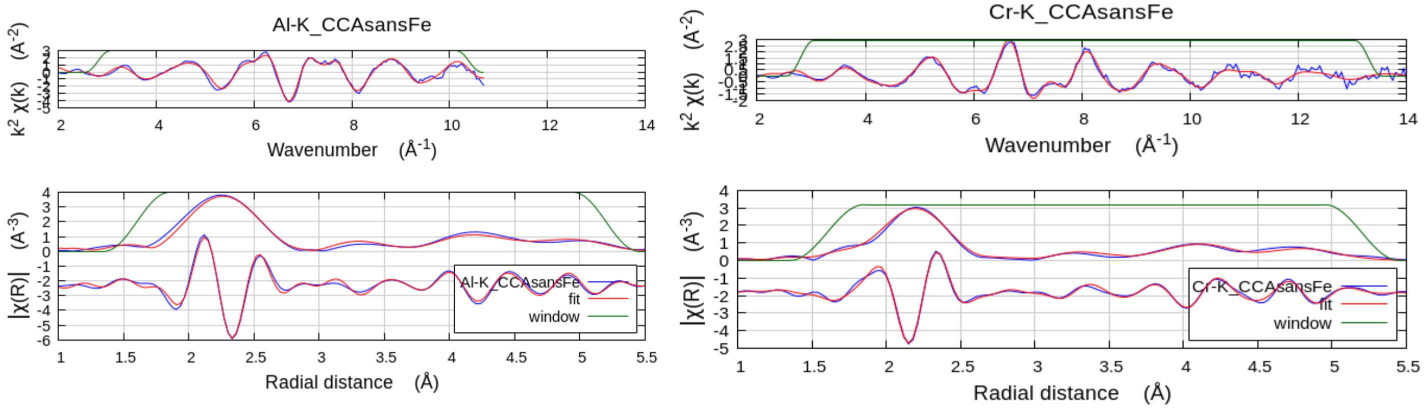

Independent points : 40.7812500
Number of variables : 15
Chi-square : 3824.1584708
Reduced chi-square : 148.3309952
R-factor : 0.0203612
Number of data sets : 2

guess parameters:
dEnot_Cr = 7.87236094 # +/- 1.48578950 [8.09411]
dEnot_Al = 7.32401649 # +/- 0.68002825 [7.32394]
dR1st = -0.01769800 # +/- 0.00478541 [-0.01770]
dRCr1st = -0.03974773 # +/- 0.01088887 [-0.03805]
ssCrAl = 0.00586520 # +/- 0.00032762 [0.00586]
ssCrCo = 0.00670811 # +/- 0.00115528 [0.00700]
x = 0.83699141 # +/- 0.82354838 [0.70657]
dRAl2nd = -0.03663860 # +/- 0.02410324 [-0.03663]
dRAl3rd = -0.01114095 # +/- 0.01515861 [-0.01114]
dRAl4th = 0.03894681 # +/- 0.01910664 [0.03895]
thetaCr = 331.57936413 # +/- 28.68740102 [331.62921]
thetaAl = 404.71818725 # +/- 19.16652140 [404.71934]
dRCr2nd = -0.03645076 # +/- 0.04876300 [-0.03655]
dRCr3rd = -0.02592734 # +/- 0.03023695 [-0.02355]
dRCr4th = -0.00621868 # +/- 0.03607696 [-0.00320]

set parameters:
amp_Cr = 0.57000000

```
  amp_Al           =   0.66000000
  temperature       = 300.00000000

: name                  = Al-K_CCAsansFe
: k-range               = 2.8 - 10.4
: dk                    = 0.5
: k-window              = Hanning
: k-weight              = 1,2,3
: R-range               = 1.6 - 5.2
: dR                    = 0.5
: R-window              = Hanning
: fitting space         = r
: background function     = no
: phase correction        = no
: background removal      = E0: 1557.235315, Rbkg: 1.0, range: [0:10.775], clamps: 0/24, kw: 2
: user-supplied epsilon_k = 0
: epsilon_k by k-weight   = 1.654e-03
: epsilon_r by k-weight   = 2.234e-01
: R-factor by k-weight    = 1 -> 0.02751,  2 -> 0.02064,  3 -> 0.02109

  name                                  N      S02    sigma^2   e0    delr    Reff    R
===============================================================================
[Al_absorber_Co_scatterer] Co1.1              12.000   0.660   0.00587   7.324 -0.01770  2.55270  2.53500
[Al_absorber_Co_scatterer] Co1.2               6.000   0.660   0.01214   7.324 -0.03664  3.61000  3.57336
[Al_absorber_Co_scatterer] Co1.1 Co1.1         48.000   0.660   0.00979   7.324 -0.01770  3.82900  3.81130
[Al_absorber_Co_scatterer] Co1.1 Co1.2         48.000   0.660   0.01135   7.324 -0.02717  4.35770  4.33053
[Al_absorber_Co_scatterer] Co1.3              24.000   0.660   0.01246   7.324 -0.01114  4.42130  4.41016
[Al_absorber_Co_scatterer] Co1.1 Co1.1         48.000   0.660   0.00873   7.324 -0.03540  4.76330  4.72790
[Al_absorber_Co_scatterer] Co1.1 Co1.3         96.000   0.660   0.01208   7.324 -0.01442  4.76330  4.74888
[Al_absorber_Co_scatterer] Co1.4              12.000   0.660   0.01260   7.324  0.03895  5.10530  5.14425
[Al_absorber_Co_scatterer] Co1.1 Co1.1         12.000   0.660   0.00779   7.324 -0.03540  5.10530  5.06990
[Al_absorber_Co_scatterer] Co1.1 Co1.4         24.000   0.660   0.01260   7.324  0.03895  5.10530  5.14425
[Al_absorber_Co_scatterer] Co1.1              12.000   0.660   0.00779   7.324 -0.03540  5.10530  5.06990
[Al_absorber_Co_scatterer] Co1.1 Co1.4 Co1.1     12.000   0.660   0.01260   7.324  0.03895  5.10530  5.14425


: name                  = Cr-K_CCAsansFe
: k-range               = 2.8 - 13.3
: dk                    = 0.5
: k-window              = Hanning
: k-weight              = 1,2,3
: R-range               = 1.6 - 5.2
: dR                    = 0.5
: R-window              = Hanning
: fitting space         = r
: background function     = no
: phase correction        = no
: background removal      = E0: 5990.22476878199, Rbkg: 1.0, range: [0:15.02], clamps: 0/24, kw: 2
: user-supplied epsilon_k = 0
: epsilon_k by k-weight   = 2.007e-03
: epsilon_r by k-weight   = 8.742e-01
: R-factor by k-weight    = 1 -> 0.01008,  2 -> 0.01461,  3 -> 0.02823

  name                                  N      S02    sigma^2   e0    delr    Reff    R
===============================================================================
[Cr_absorber_Al_scatterer] Al1.1               1.000   0.477   0.00587   7.872 -0.01770  2.55270  2.53500
```

[Cr_absorber_Co_scatterer] Co1.1 1.000 6.363 0.00671 7.872 -0.03975 2.55270 2.51295
[Cr_absorber_Co_scatterer] Co1.2 6.000 0.570 0.01164 7.872 -0.03645 3.61000 3.57355
[Cr_absorber_Co_scatterer] Co1.1 Co1.1 48.000 0.570 0.01035 7.872 -0.05604 3.82900 3.77296
[Cr_absorber_Co_scatterer] Co1.1 Co1.2 48.000 0.570 0.01157 7.872 -0.03810 4.35770 4.31960
[Cr_absorber_Co_scatterer] Co1.3 24.000 0.570 0.01200 7.872 -0.02593 4.42130 4.39537
[Cr_absorber_Co_scatterer] Co1.1 Co1.1 48.000 0.570 0.01144 7.872 -0.07949 4.76330 4.68381
[Cr_absorber_Co_scatterer] Co1.1 Co1.3 96.000 0.570 0.01197 7.872 -0.03284 4.76330 4.73046
[Cr_absorber_Co_scatterer] Co1.4 12.000 0.570 0.01216 7.872 -0.00622 5.10530 5.09908
[Cr_absorber_Co_scatterer] Co1.1 Co1.1 12.000 0.570 0.01139 7.872 -0.07949 5.10530 5.02581
[Cr_absorber_Co_scatterer] Co1.1 Co1.4 24.000 0.570 0.01216 7.872 -0.00622 5.10530 5.09908
[Cr_absorber_Co_scatterer] Co1.1 12.000 0.570 0.01139 7.872 -0.07949 5.10530 5.02581
[Cr_absorber_Co_scatterer] Co1.1 Co1.4 Co1.1 12.000 0.570 0.01216 7.872 -0.00622 5.10530 5.09908

Al-K; Co-K

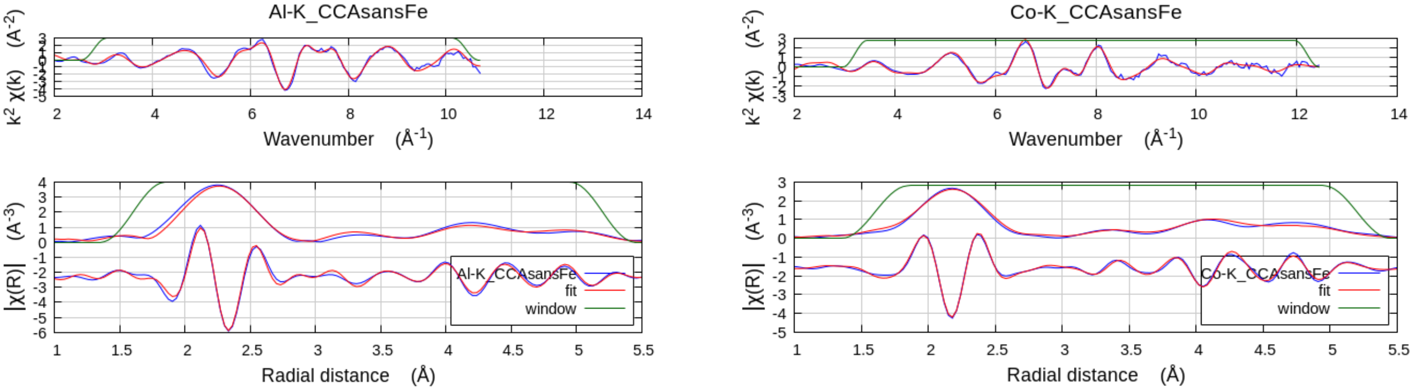

Independent points : 37.3828125
Number of variables : 15
Chi-square : 3809.8307787
Reduced chi-square : 170.2123350
R-factor : 0.0186854
Number of data sets : 2

guess parameters:
dEnot_Al = 7.32931721 # +/- 0.72828530 [7.33121]
dEnot_Co = 8.15635370 # +/- 1.90777721 [8.03811]
dR1st = -0.01765336 # +/- 0.00512494 [-0.01764]
dRCo1st = -0.04512315 # +/- 0.01236774 [-0.04572]
ssCoAl = 0.00586605 # +/- 0.00035100 [0.00587]
ssCoCo = 0.00878397 # +/- 0.00136711 [0.00880]
x = 1.58883053 # +/- 0.71948443 [1.57394]
thetaAl = 404.71541590 # +/- 20.53120665 [404.71475]
thetaCo = 297.84391432 # +/- 19.79719446 [297.80199]
dRCo2nd = -0.00502780 # +/- 0.04610654 [-0.00589]
dRCo3rd = -0.02897473 # +/- 0.03000921 [-0.03011]
dRCo4th = -0.02053972 # +/- 0.03697877 [-0.02193]
dRAl2nd = -0.03660667 # +/- 0.02582004 [-0.03659]
dRAl3rd = -0.01107725 # +/- 0.01623850 [-0.01105]
dRAl4th = 0.03900491 # +/- 0.02046777 [0.03903]

```
set parameters:
  amp_Al          =   0.66000000
  amp_Co          =   0.78000000
  temperature     = 300.00000000

: name                   = Al-K_CCAsansFe
: k-range                = 2.8 - 10.4
: dk                     = 0.5
: k-window               = Hanning
: k-weight               = 1,2,3
: R-range                = 1.6 - 5.2
: dR                     = 0.5
: R-window               = Hanning
: fitting space          = r
: background function    = no
: phase correction       = no
: background removal     = E0: 1557.235315, Rbkg: 1.0, range: [0:10.775], clamps: 0/24, kw: 2
: user-supplied epsilon_k = 0
: epsilon_k by k-weight  = 1.654e-03
: epsilon_r by k-weight  = 2.234e-01
: R-factor by k-weight   = 1 -> 0.02749,  2 -> 0.02065,  3 -> 0.02111

  name                                       N      S02    sigma^2   e0     delr     Reff     R
=========================================================================================
[Al_absorber_Co_scatterer] Co1.1             12.000   0.660   0.00587   7.329 -0.01765  2.55270  2.53505
[Al_absorber_Co_scatterer] Co1.2              6.000   0.660   0.01214   7.329 -0.03661  3.61000  3.57339
[Al_absorber_Co_scatterer] Co1.1 Co1.1       48.000   0.660   0.00979   7.329 -0.01765  3.82900  3.81135
[Al_absorber_Co_scatterer] Co1.1 Co1.2       48.000   0.660   0.01135   7.329 -0.02713  4.35770  4.33057
[Al_absorber_Co_scatterer] Co1.3             24.000   0.660   0.01246   7.329 -0.01108  4.42130  4.41022
[Al_absorber_Co_scatterer] Co1.1 Co1.1       48.000   0.660   0.00873   7.329 -0.03531  4.76330  4.72799
[Al_absorber_Co_scatterer] Co1.1 Co1.3       96.000   0.660   0.01208   7.329 -0.01436  4.76330  4.74894
[Al_absorber_Co_scatterer] Co1.4             12.000   0.660   0.01260   7.329  0.03900  5.10530  5.14431
[Al_absorber_Co_scatterer] Co1.1 Co1.1       12.000   0.660   0.00779   7.329 -0.03531  5.10530  5.06999
[Al_absorber_Co_scatterer] Co1.1 Co1.4       24.000   0.660   0.01260   7.329  0.03900  5.10530  5.14431
[Al_absorber_Co_scatterer] Co1.1             12.000   0.660   0.00779   7.329 -0.03531  5.10530  5.06999
[Al_absorber_Co_scatterer] Co1.1 Co1.4 Co1.1     12.000   0.660   0.01260   7.329  0.03900  5.10530  5.14431

: name                   = Co-K_CCAsansFe
: k-range                = 3.2 - 12.2
: dk                     = 0.5
: k-window               = Hanning
: k-weight               = 1,2,3
: R-range                = 1.6 - 5.2
: dR                     = 0.5
: R-window               = Hanning
: fitting space          = r
: background function    = no
: phase correction       = no
: background removal     = E0: 7710.8089040442, Rbkg: 1.0, range: [0:12.506], clamps: 0/24, kw: 2
: user-supplied epsilon_k = 0
: epsilon_k by k-weight  = 1.753e-03
: epsilon_r by k-weight  = 4.025e-01
: R-factor by k-weight   = 1 -> 0.00938,  2 -> 0.01223,  3 -> 0.02124

  name                                       N      S02    sigma^2   e0     delr     Reff     R
```

===
[Co_absorber_Co_scatterer] Co1.1 1.000 8.121 0.00878 8.156 -0.04512 2.55270 2.50758
[Co_absorber_Al_scatterer] Al1.1 1.000 1.239 0.00587 8.156 -0.01765 2.55270 2.53505
[Co_absorber_Co_scatterer] Co1.2 6.000 0.780 0.01339 8.156 -0.00503 3.61000 3.60497
[Co_absorber_Co_scatterer] Co1.1 Co1.1 48.000 0.780 0.01213 8.156 -0.04512 3.82900 3.78388
[Co_absorber_Co_scatterer] Co1.1 Co1.2 48.000 0.780 0.01348 8.156 -0.02508 4.35770 4.33263
[Co_absorber_Co_scatterer] Co1.3 24.000 0.780 0.01383 8.156 -0.02898 4.42130 4.39232
[Co_absorber_Co_scatterer] Co1.1 Co1.1 48.000 0.780 0.01387 8.156 -0.09025 4.76330 4.67305
[Co_absorber_Co_scatterer] Co1.1 Co1.3 96.000 0.780 0.01387 8.156 -0.09025 4.76330 4.67305
[Co_absorber_Co_scatterer] Co1.4 12.000 0.780 0.01401 8.156 -0.02054 5.10530 5.08476
[Co_absorber_Co_scatterer] Co1.1 Co1.1 12.000 0.780 0.01401 8.156 -0.09025 5.10530 5.01505
[Co_absorber_Co_scatterer] Co1.1 Co1.4 24.000 0.780 0.01401 8.156 -0.02054 5.10530 5.08476
[Co_absorber_Co_scatterer] Co1.1 12.000 0.780 0.01401 8.156 -0.09025 5.10530 5.01505
[Co_absorber_Co_scatterer] Co1.1 Co1.4 Co1.1 12.000 0.780 0.01401 8.156 -0.02054 5.10530 5.08476

Al-K; Ni-K

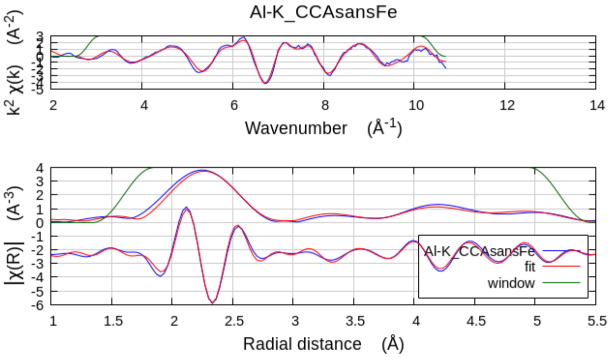

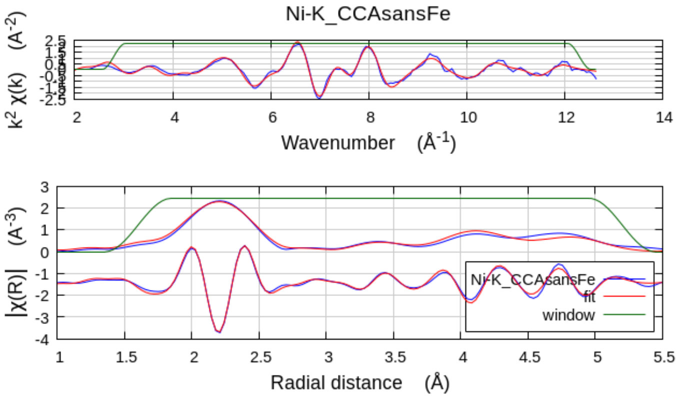

Independent points : 38.5156250
Number of variables : 15
Chi-square : 5687.1809125
Reduced chi-square : 241.8468959
R-factor : 0.0197602
Number of data sets : 2

guess parameters:
dEnot_Ni = 7.15385989 # +/- 0.91436825 [7.15392]
dEnot_Al = 7.50777132 # +/- 0.86367933 [7.50775]
dR1st = -0.01634529 # +/- 0.00601591 [-0.01635]
dRNi1st = -0.03308137 # +/- 0.00536756 [-0.03308]
ssNiAl = 0.00587019 # +/- 0.00041887 [0.00587]
ssNiCo = 0.00749268 # +/- 0.00058263 [0.00749]
x = 2.88458834 # +/- 0.31232726 [2.88458]
dRAl2nd = -0.02818504 # +/- 0.03091505 [-0.02818]
dRAl3rd = -0.00963983 # +/- 0.01947513 [-0.00964]
dRAl4th = 0.04065843 # +/- 0.02438165 [0.04066]
thetaAl = 404.67796640 # +/- 24.32197601 [404.67814]
thetaNi = 298.16429144 # +/- 9.52788394 [298.16455]
dRNi2nd = -0.00023632 # +/- 0.02168082 [-0.00024]
dRNi3rd = -0.01233826 # +/- 0.01488952 [-0.01234]
dRNi4th = -0.02616071 # +/- 0.01707162 [-0.02616]

set parameters:
amp_Ni = 0.77000000
amp_Al = 0.66000000
temperature = 300.00000000

: name = Al-K_CCAsansFe
: k-range = 2.8 - 10.4
: dk = 0.5
: k-window = Hanning
: k-weight = 1,2,3
: R-range = 1.6 - 5.2
: dR = 0.5
: R-window = Hanning
: fitting space = r
: background function = no
: phase correction = no
: background removal = E0: 1557.235315, Rbkg: 1.0, range: [0:10.775], clamps: 0/24, kw: 2
: user-supplied epsilon_k = 0
: epsilon_k by k-weight = 1.654e-03
: epsilon_r by k-weight = 2.234e-01
: R-factor by k-weight = 1 -> 0.02641, 2 -> 0.02005, 3 -> 0.02093

name	N	S02	sigma^2	e0	delr	Reff	R
[Al_absorber_Co_scatterer] Co1.1	12.000	0.660	0.00587	7.508	-0.01634	2.55270	2.53636
[Al_absorber_Co_scatterer] Co1.2	6.000	0.660	0.01214	7.508	-0.02819	3.61000	3.58181
[Al_absorber_Co_scatterer] Co1.1 Co1.1	48.000	0.660	0.00980	7.508	-0.02305	3.82900	3.80595
[Al_absorber_Co_scatterer] Co1.1 Co1.1	24.000	0.660	0.00953	7.508	-0.02305	4.35770	4.33465
[Al_absorber_Co_scatterer] Co1.1 Co1.2	48.000	0.660	0.01135	7.508	-0.02227	4.35770	4.33544
[Al_absorber_Co_scatterer] Co1.3	24.000	0.660	0.01246	7.508	-0.00964	4.42130	4.41166
[Al_absorber_Co_scatterer] Co1.1 Co1.1	48.000	0.660	0.00873	7.508	-0.03269	4.76330	4.73061
[Al_absorber_Co_scatterer] Co1.1 Co1.3	96.000	0.660	0.01208	7.508	-0.01299	4.76330	4.75031
[Al_absorber_Co_scatterer] Co1.4	12.000	0.660	0.01260	7.508	0.04066	5.10530	5.14596
[Al_absorber_Co_scatterer] Co1.1 Co1.1	12.000	0.660	0.00779	7.508	-0.03269	5.10530	5.07261
[Al_absorber_Co_scatterer] Co1.1 Co1.4	24.000	0.660	0.01260	7.508	0.04066	5.10530	5.14596
[Al_absorber_Co_scatterer] Co1.1	12.000	0.660	0.00779	7.508	-0.03269	5.10530	5.07261
[Al_absorber_Co_scatterer] Co1.1 Co1.4 Co1.1	12.000	0.660	0.01260	7.508	0.04066	5.10530	5.14596

: name = Ni-K_CCAsansFe
: k-range = 2.8 - 12.3
: dk = 0.5
: k-window = Hanning
: k-weight = 1,2,3
: R-range = 1.6 - 5.2
: dR = 0.5
: R-window = Hanning
: fitting space = r
: background function = no
: phase correction = no
: background removal = E0: 8333.55651446316, Rbkg: 1.0, range: [0:12.719], clamps: 0/24, kw: 2
: user-supplied epsilon_k = 0
: epsilon_k by k-weight = 6.302e-04
: epsilon_r by k-weight = 1.530e-01
: R-factor by k-weight = 1 -> 0.02015, 2 -> 0.01445, 3 -> 0.01657

name	N	S02	sigma^2	e0	delr	Reff	R
[Ni_absorber_Co_scatterer] Co1.1	1.000	7.019	0.00749	7.154	-0.03308	2.55270	2.51962
[Ni_absorber_Al_scatterer] Al1.1	1.000	2.221	0.00587	7.154	-0.01634	2.55270	2.53636
[Ni_absorber_Co_scatterer] Co1.2	6.000	0.770	0.01338	7.154	-0.00024	3.61000	3.60976
[Ni_absorber_Co_scatterer] Co1.1 Co1.1	48.000	0.770	0.01210	7.154	-0.04664	3.82900	3.78236
[Ni_absorber_Co_scatterer] Co1.1 Co1.1	24.000	0.770	0.01344	7.154	-0.04664	4.35770	4.31106
[Ni_absorber_Co_scatterer] Co1.1 Co1.2	48.000	0.770	0.01346	7.154	-0.01666	4.35770	4.34104
[Ni_absorber_Co_scatterer] Co1.3	24.000	0.770	0.01382	7.154	-0.01234	4.42130	4.40896
[Ni_absorber_Co_scatterer] Co1.1 Co1.1	48.000	0.770	0.01384	7.154	-0.06616	4.76330	4.69714
[Ni_absorber_Co_scatterer] Co1.1 Co1.3	96.000	0.770	0.01386	7.154	-0.02271	4.76330	4.74059
[Ni_absorber_Co_scatterer] Co1.4	12.000	0.770	0.01401	7.154	-0.02616	5.10530	5.07914
[Ni_absorber_Co_scatterer] Co1.1 Co1.1	12.000	0.770	0.01398	7.154	-0.06616	5.10530	5.03914
[Ni_absorber_Co_scatterer] Co1.1 Co1.4	24.000	0.770	0.01401	7.154	-0.02616	5.10530	5.07914
[Ni_absorber_Co_scatterer] Co1.1	12.000	0.770	0.01398	7.154	-0.06616	5.10530	5.03914
[Ni_absorber_Co_scatterer] Co1.1 Co1.4 Co1.1	12.000	0.770	0.01401	7.154	-0.02616	5.10530	5.07914

Al-K; Cu-K

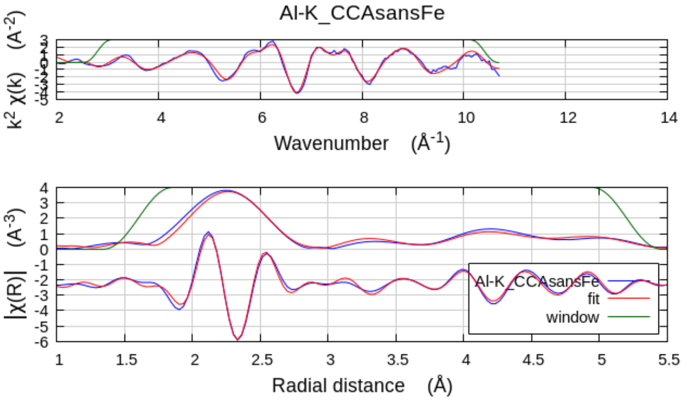

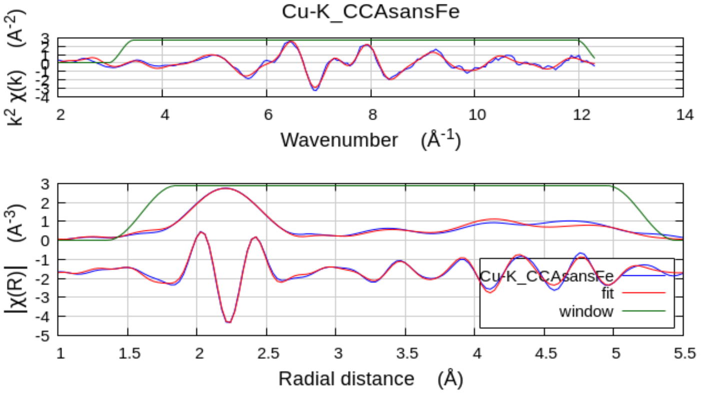

Independent points : 37.3828125
Number of variables : 15
Chi-square : 4904.7756804
Reduced chi-square : 219.1313393
R-factor : 0.0204198
Number of data sets : 2

guess parameters:

dEnot_Al	= 7.46234418	# +/-	0.81349342	[7.46238]
dEnot_Cu	= 7.98333598	# +/-	1.51598875	[7.98309]
dRCu1st	= -0.01946422	# +/-	0.00777212	[-0.01947]
dR1st	= -0.01656422	# +/-	0.00573285	[-0.01656]
ssAlCo	= 0.00587046	# +/-	0.00039879	[0.00587]
ssCuCo	= 0.00653404	# +/-	0.00071718	[0.00653]
x	= 2.86544089	# +/-	0.44954632	[2.9]
thetaAl	= 404.64976242	# +/-	23.28562189	[404.64509]
thetaCu	= 314.55409701	# +/-	13.35286349	[314.55370]
dRAl2nd	= -0.03568307	# +/-	0.02928807	[-0.03571]
dRAl3rd	= -0.00947188	# +/-	0.01838446	[-0.00947]
dRAl4th	= 0.04046849	# +/-	0.02317947	[0.04047]
dRCu2nd	= 0.00162304	# +/-	0.02702488	[0.00162]
dRCu3rd	= 0.00985876	# +/-	0.02042882	[0.00986]

```
  dRCu4th           =  -0.00136414    # +/-   0.02348230     [-0.00137]

set parameters:
  amp_Al            =   0.66000000
  amp_Cu            =   0.79000000
  temperature       = 300.00000000

: name                    = Al-K_CCAsansFe
: k-range                 = 2.8 - 10.4
: dk                      = 0.5
: k-window                = Hanning
: k-weight                = 1,2,3
: R-range                 = 1.6 - 5.2
: dR                      = 0.5
: R-window                = Hanning
: fitting space           = r
: background function     = no
: phase correction        = no
: background removal      = E0: 1557.235315, Rbkg: 1.0, range: [0:10.775], clamps: 0/24, kw: 2
: user-supplied epsilon_k = 0
: epsilon_k by k-weight   = 1.654e-03
: epsilon_r by k-weight   = 2.234e-01
: R-factor by k-weight    = 1 -> 0.02705,  2 -> 0.02095,  3 -> 0.02174

  name                                 N      S02     sigma^2   e0     delr     Reff     R
=====================================================================================
[Al_absorber_Co_scatterer] Co1.1              12.000   0.660   0.00587   7.462 -0.01656  2.55270  2.53614
[Al_absorber_Co_scatterer] Co1.2               6.000   0.660   0.01214   7.462 -0.03568  3.61000  3.57432
[Al_absorber_Co_scatterer] Co1.1 Co1.1        48.000   0.660   0.00980   7.462 -0.01656  3.82900  3.81244
[Al_absorber_Co_scatterer] Co1.1 Co1.2        48.000   0.660   0.01135   7.462 -0.02612  4.35770  4.33158
[Al_absorber_Co_scatterer] Co1.3              24.000   0.660   0.01246   7.462 -0.00947  4.42130  4.41183
[Al_absorber_Co_scatterer] Co1.1 Co1.1        48.000   0.660   0.00873   7.462 -0.03313  4.76330  4.73017
[Al_absorber_Co_scatterer] Co1.1 Co1.3        96.000   0.660   0.01208   7.462 -0.01302  4.76330  4.75028
[Al_absorber_Co_scatterer] Co1.4              12.000   0.660   0.01260   7.462  0.04047  5.10530  5.14577
[Al_absorber_Co_scatterer] Co1.1 Co1.1        12.000   0.660   0.00779   7.462 -0.03313  5.10530  5.07217
[Al_absorber_Co_scatterer] Co1.1 Co1.4        24.000   0.660   0.01260   7.462  0.04047  5.10530  5.14577
[Al_absorber_Co_scatterer] Co1.1              12.000   0.660   0.00779   7.462 -0.03313  5.10530  5.07217
[Al_absorber_Co_scatterer] Co1.1 Co1.4 Co1.1  12.000   0.660   0.01260   7.462  0.04047  5.10530  5.14577

: name                    = Cu-K_CCAsansFe
: k-range                 = 3.200 - 12.2
: dk                      = 0.5
: k-window                = Hanning
: k-weight                = 1,2,3
: R-range                 = 1.6 - 5.2
: dR                      = 0.5
: R-window                = Hanning
: fitting space           = r
: background function     = no
: phase correction        = no
: background removal      = E0: 8983.21654194799, Rbkg: 1.0, range: [0:12.384], clamps: 0/24, kw: 2
: user-supplied epsilon_k = 0
: epsilon_k by k-weight   = 1.109e-03
: epsilon_r by k-weight   = 2.441e-01
: R-factor by k-weight    = 1 -> 0.01685,  2 -> 0.01541,  3 -> 0.02057
```

name	N	S02	sigma^2	e0	delr	Reff	R
[Cu_absorber_Co_scatterer] Co1.1	1.000	7.216	0.00653	7.983	-0.01946	2.55270	2.53324
[Cu_absorber_Al_scatterer] Al1.1	1.000	2.264	0.00587	7.983	-0.01656	2.55270	2.53614
[Cu_absorber_Co_scatterer] Co1.2	6.000	0.790	0.01162	7.983	0.00162	3.61000	3.61162
[Cu_absorber_Co_scatterer] Co1.1 Co1.1	48.000	0.790	0.01066	7.983	-0.01946	3.82900	3.80954
[Cu_absorber_Co_scatterer] Co1.1 Co1.2	48.000	0.790	0.01178	7.983	-0.00892	4.35770	4.34878
[Cu_absorber_Co_scatterer] Co1.3	24.000	0.790	0.01199	7.983	0.00986	4.42130	4.43116
[Cu_absorber_Co_scatterer] Co1.1 Co1.1	48.000	0.790	0.01239	7.983	-0.03893	4.76330	4.72437
[Cu_absorber_Co_scatterer] Co1.1 Co1.3	96.000	0.790	0.01207	7.983	-0.00480	4.76330	4.75850
[Cu_absorber_Co_scatterer] Co1.4	12.000	0.790	0.01215	7.983	-0.00136	5.10530	5.10394
[Cu_absorber_Co_scatterer] Co1.1 Co1.1	12.000	0.790	0.01261	7.983	-0.03893	5.10530	5.06637
[Cu_absorber_Co_scatterer] Co1.1 Co1.4	24.000	0.790	0.01215	7.983	-0.00136	5.10530	5.10394
[Cu_absorber_Co_scatterer] Co1.1	12.000	0.790	0.01261	7.983	-0.03893	5.10530	5.06637
[Cu_absorber_Co_scatterer] Co1.1 Co1.4 Co1.1	12.000	0.790	0.01215	7.983	-0.00136	5.10530	5.10394

CCA$_{sans}$Co

Al-K; Cr-K

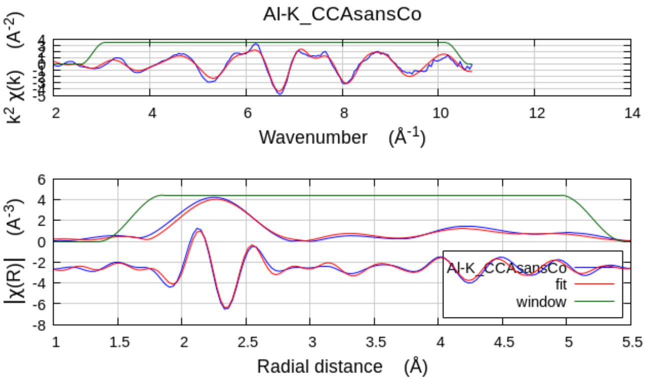

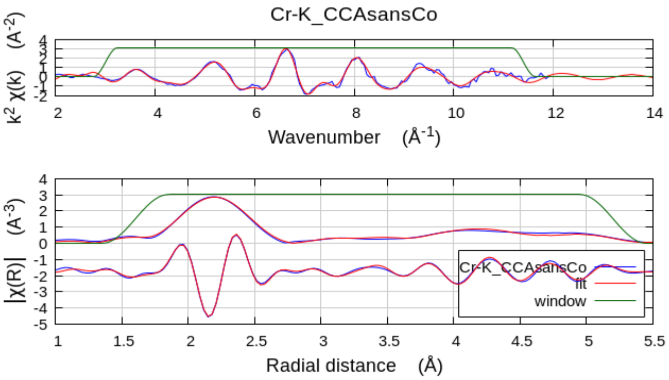

Independent points : 36.0234375
Number of variables : 15
Chi-square : 4246.4408062
Reduced chi-square : 201.9860361
R-factor : 0.0274565
Number of data sets : 2

guess parameters:
dEnot_Cr = 8.68312670 # +/- 1.92650582 [8.68140]
dEnot_Al = 6.80991611 # +/- 1.07113053 [6.80983]
dR1st = -0.00832506 # +/- 0.00759791 [-0.00833]
x = 0.75669994 # +/- 1.13223031 [0.75629]
dRCr1st = -0.02490012 # +/- 0.01318633 [-0.02491]
dRCr2nd = 0.00535949 # +/- 0.06258406 [0.00531]
dRCr3rd = 0.00945096 # +/- 0.04014225 [0.00942]
dRCr4th = 0.02941752 # +/- 0.05054937 [0.02939]
dRAl2nd = -0.03201155 # +/- 0.03619485 [-0.03202]
dRAl3rd = 0.00171100 # +/- 0.02272947 [0.00171]
dRAl4th = 0.03142103 # +/- 0.04948123 [0.03139]
thetaCr = 271.93991300 # +/- 21.27790685 [271.93720]
thetaAl = 432.59070739 # +/- 46.54398828 [432.57125]
ssCrAl = 0.00496351 # +/- 0.00051478 [0.00496]
ssCrCo = 0.00642560 # +/- 0.00153066 [0.00643]

```
set parameters:
  amp_Cr           =   0.57000000
  amp_Al           =   0.66000000
  temperature      = 300.00000000

: name                  = Al-K_CCAsansCo
: k-range               = 2.8 - 10.4
: dk                    = 0.5
: k-window              = Hanning
: k-weight              = 1,2,3
: R-range               = 1.6 - 5.2
: dR                    = 0.5
: R-window              = Hanning
: fitting space         = r
: background function   = no
: phase correction      = no
: background removal    = E0: 1557.481523, Rbkg: 1.0, range: [0:10.772], clamps: 0/24, kw: 2
: user-supplied epsilon_k = 0
: epsilon_k by k-weight   = 2.710e-03
: epsilon_r by k-weight   = 3.662e-01
: R-factor by k-weight    = 1 -> 0.05091,  2 -> 0.04307,  3 -> 0.04593

  name                                N      S02     sigma^2   e0     delr     Reff     R
=====================================================================================
[Al_absorber_Co_scatterer] Co1.1               12.000   0.660   0.00496   6.810 -0.00833  2.55270  2.54438
[Al_absorber_Co_scatterer] Co1.2                6.000   0.660   0.01072   6.810 -0.03201  3.61000  3.57799
[Al_absorber_Co_scatterer] Co1.1 Co1.1         48.000   0.660   0.00866   6.810 -0.01174  3.82900  3.81726
[Al_absorber_Co_scatterer] Co1.1 Co1.2         48.000   0.660   0.01002   6.810 -0.02017  4.35770  4.33753
[Al_absorber_Co_scatterer] Co1.3               24.000   0.660   0.01099   6.810  0.00171  4.42130  4.42301
[Al_absorber_Co_scatterer] Co1.1 Co1.1         48.000   0.660   0.00770   6.810 -0.01665  4.76330  4.74665
[Al_absorber_Co_scatterer] Co1.1 Co1.3         96.000   0.660   0.01066   6.810 -0.00331  4.76330  4.75999
[Al_absorber_Co_scatterer] Co1.4               12.000   0.660   0.01111   6.810  0.03142  5.10530  5.13672
[Al_absorber_Co_scatterer] Co1.1 Co1.1         12.000   0.660   0.00687   6.810 -0.01665  5.10530  5.08865
[Al_absorber_Co_scatterer] Co1.1 Co1.4         24.000   0.660   0.01111   6.810  0.01155  5.10530  5.11685
[Al_absorber_Co_scatterer] Co1.1               12.000   0.660   0.00687   6.810 -0.01665  5.10530  5.08865
[Al_absorber_Co_scatterer] Co1.1 Co1.4 Co1.1   12.000   0.660   0.01111   6.810  0.03142  5.10530  5.13672

: name                  = Cr-K_CCAsansCo
: k-range               = 3 - 11.4
: dk                    = 0.5
: k-window              = Hanning
: k-weight              = 1,2,3
: R-range               = 1.6 - 5.2
: dR                    = 0.5
: R-window              = Hanning
: fitting space         = r
: background function   = no
: phase correction      = no
: background removal    = E0: 5989.73739912488, Rbkg: 1.0, range: [0:11.8], clamps: 0/24, kw: 2
: user-supplied epsilon_k = 0
: epsilon_k by k-weight   = 1.006e-03
: epsilon_r by k-weight   = 4.382e-01
: R-factor by k-weight    = 1 -> 0.00709,  2 -> 0.00730,  3 -> 0.01043

  name                                N      S02     sigma^2   e0     delr     Reff     R
```

```
==================================================================================
[Cr_absorber_Al_scatterer] Al1.1              1.000   0.431   0.00496   8.683 -0.00833  2.55270  2.54438
[Cr_absorber_Co_scatterer] Co1.1              1.000   6.409   0.00643   8.683 -0.02490  2.55270  2.52780
[Cr_absorber_Co_scatterer] Co1.2              6.000   1.000   0.01705   8.683  0.00536  3.61000  3.61536
[Cr_absorber_Co_scatterer] Co1.1 Co1.1        48.000   1.000   0.01513   8.683 -0.03511  3.82900  3.79389
[Cr_absorber_Co_scatterer] Co1.1 Co1.1        24.000   1.000   0.01651   8.683 -0.03511  4.35770  4.32259
[Cr_absorber_Co_scatterer] Co1.1 Co1.2        48.000   1.000   0.01694   8.683 -0.00977  4.35770  4.34793
[Cr_absorber_Co_scatterer] Co1.3              24.000   1.000   0.01761   8.683  0.00945  4.42130  4.43075
[Cr_absorber_Co_scatterer] Co1.1 Co1.1        48.000   1.000   0.01676   8.683 -0.04980  4.76330  4.71350
[Cr_absorber_Co_scatterer] Co1.1 Co1.3        96.000   1.000   0.01755   8.683 -0.00773  4.76330  4.75558
[Cr_absorber_Co_scatterer] Co1.4              12.000   1.000   0.01785   8.683  0.02942  5.10530  5.13472
[Cr_absorber_Co_scatterer] Co1.1 Co1.1        12.000   1.000   0.01673   8.683 -0.04980  5.10530  5.05550
[Cr_absorber_Co_scatterer] Co1.1 Co1.4        24.000   1.000   0.01785   8.683  0.02942  5.10530  5.13472
[Cr_absorber_Co_scatterer] Co1.1              12.000   1.000   0.01673   8.683 -0.04980  5.10530  5.05550
[Cr_absorber_Co_scatterer] Co1.1 Co1.4 Co1.1   12.000   1.000   0.01785   8.683  0.02942  5.10530  5.13472
```

Al-K; Fe-K

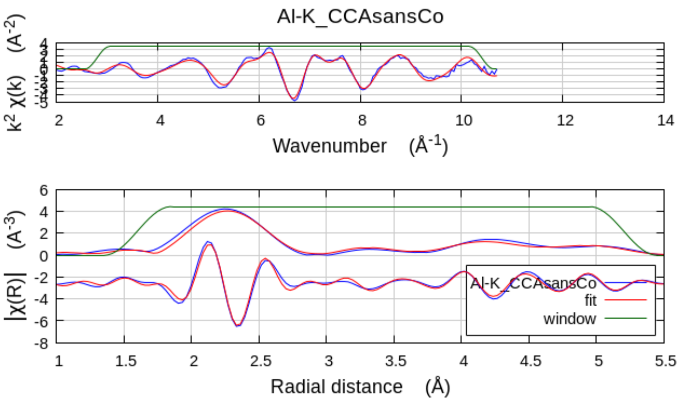

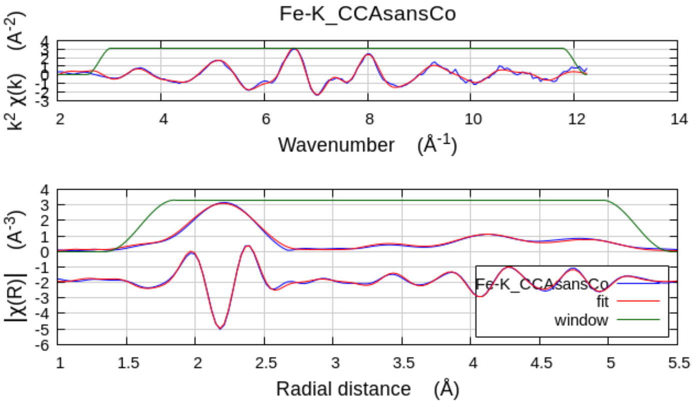


```
Independent points        : 37.8359375
Number of variables        : 15
Chi-square               : 3619.9534546
Reduced chi-square         : 158.5200281
R-factor                : 0.0222036
Number of data sets        : 2

guess parameters:
  dEnot_Fe        =   8.02921277   # +/-   1.06368210    [8.02921]
  dEnot_Al        =   7.06211909   # +/-   0.98168191    [7.06207]
  dR1st         =  -0.00913935   # +/-   0.00676618    [-0.00914]
  dRAl2nd         =  -0.02009206   # +/-   0.03290693    [-0.02006]
  dRAl3rd         =   0.00619569   # +/-   0.02089242    [0.00620]
  dRAl4th         =   0.06140489   # +/-   0.02594954    [0.06140]
  x             =   0.96807508   # +/-   0.52936222    [0.96808]
  dRFe1st         =  -0.02866158   # +/-   0.00798826    [-0.02866]
  dRFe2nd         =   0.00842674   # +/-   0.03315406    [0.00843]
  dRFe3rd         =  -0.00293730   # +/-   0.02046899    [-0.00294]
  dRFe4th         =   0.00582591   # +/-   0.02405858    [0.00583]
  ssAlCo         =   0.00495440   # +/-   0.00045465    [0.00495]
  thetaFe        = 313.94127411   # +/-  16.92664394    [313.94130]
  thetaAl        = 429.26665925   # +/-  30.91994231    [429.27212]
  ssFeCo         =   0.00794555   # +/-   0.00091702    [0.00795]
```

```
set parameters:
  amp_Fe          =   0.75000000
  amp_Al          =   0.66000000
  temperature      = 300.00000000

: name                  = Al-K_CCAsansCo
: k-range               = 2.8 - 10.4
: dk                    = 0.5
: k-window              = Hanning
: k-weight              = 1,2,3
: R-range               = 1.6 - 5.2
: dR                    = 0.5
: R-window              = Hanning
: fitting space         = r
: background function   = no
: phase correction      = no
: background removal    = E0: 1557.481523, Rbkg: 1.0, range: [0:10.772], clamps: 0/24, kw: 2
: user-supplied epsilon_k = 0
: epsilon_k by k-weight   = 2.710e-03
: epsilon_r by k-weight   = 3.662e-01
: R-factor by k-weight    = 1 -> 0.04309,  2 -> 0.03211,  3 -> 0.03286

  name                                  N      S02     sigma^2   e0     delr     Reff     R
=============================================================================================
[Al_absorber_Co_scatterer] Co1.1               12.000   0.660   0.00495   7.062 -0.00914  2.55270  2.54356
[Al_absorber_Co_scatterer] Co1.2                6.000   0.660   0.01087   7.062 -0.02009  3.61000  3.58991
[Al_absorber_Co_scatterer] Co1.1 Co1.1         48.000   0.660   0.00878   7.062 -0.00914  3.82900  3.81986
[Al_absorber_Co_scatterer] Co1.1 Co1.1         24.000   0.660   0.00854   7.062 -0.00914  4.35770  4.34856
[Al_absorber_Co_scatterer] Co1.1 Co1.2         48.000   0.660   0.01017   7.062 -0.01462  4.35770  4.34308
[Al_absorber_Co_scatterer] Co1.3               24.000   0.660   0.01115   7.062  0.00620  4.42130  4.42750
[Al_absorber_Co_scatterer] Co1.1 Co1.1         48.000   0.660   0.00782   7.062 -0.01828  4.76330  4.74502
[Al_absorber_Co_scatterer] Co1.1 Co1.3         96.000   0.660   0.01081   7.062 -0.00147  4.76330  4.76183
[Al_absorber_Co_scatterer] Co1.4               12.000   0.660   0.01127   7.062  0.06141  5.10530  5.16670
[Al_absorber_Co_scatterer] Co1.1 Co1.1         12.000   0.660   0.00697   7.062 -0.01828  5.10530  5.08702
[Al_absorber_Co_scatterer] Co1.1 Co1.4         24.000   0.660   0.01127   7.062  0.06141  5.10530  5.16670
[Al_absorber_Co_scatterer] Co1.1               12.000   0.660   0.00697   7.062 -0.01828  5.10530  5.08702
[Al_absorber_Co_scatterer] Co1.1 Co1.4 Co1.1   12.000   0.660   0.01127   7.062  0.06141  5.10530  5.16670

: name                  = Fe-K_CCAsansCo
: k-range               = 2.8 - 12
: dk                    = 0.5
: k-window              = Hanning
: k-weight              = 1,2,3
: R-range               = 1.6 - 5.2
: dR                    = 0.5
: R-window              = Hanning
: fitting space         = r
: background function   = no
: phase correction      = no
: background removal    = E0: 7112.54484153962, Rbkg: 1.0, range: [0.000:12.319], clamps: 0/24, kw: 2
: user-supplied epsilon_k = 0
: epsilon_k by k-weight   = 1.530e-03
: epsilon_r by k-weight   = 3.320e-01
: R-factor by k-weight    = 1 -> 0.00667,  2 -> 0.00707,  3 -> 0.01144
```

name	N	S02	sigma^2	e0	delr	Reff	R
[Fe_absorber_Al_scatterer] Al1.1	1.000	0.726	0.00495	8.029	-0.00914	2.55270	2.54356
[Fe_absorber_Co_scatterer] Co1.1	1.000	8.274	0.00795	8.029	-0.02866	2.55270	2.52404
[Fe_absorber_Co_scatterer] Co1.2	6.000	0.750	0.01244	8.029	0.00843	3.61000	3.61843
[Fe_absorber_Co_scatterer] Co1.1 Co1.1	48.000	0.750	0.01118	8.029	-0.04041	3.82900	3.78859
[Fe_absorber_Co_scatterer] Co1.1 Co1.1	24.000	0.750	0.01232	8.029	-0.04041	4.35770	4.31729
[Fe_absorber_Co_scatterer] Co1.1 Co1.2	48.000	0.750	0.01245	8.029	-0.01012	4.35770	4.34758
[Fe_absorber_Co_scatterer] Co1.3	24.000	0.750	0.01284	8.029	-0.00294	4.42130	4.41836
[Fe_absorber_Co_scatterer] Co1.1 Co1.1	48.000	0.750	0.01260	8.029	-0.05732	4.76330	4.70598
[Fe_absorber_Co_scatterer] Co1.1 Co1.3	96.000	0.750	0.01285	8.029	-0.01580	4.76330	4.74750
[Fe_absorber_Co_scatterer] Co1.4	12.000	0.750	0.01301	8.029	0.00583	5.10530	5.11113
[Fe_absorber_Co_scatterer] Co1.1 Co1.1	12.000	0.750	0.01266	8.029	-0.05732	5.10530	5.04798
[Fe_absorber_Co_scatterer] Co1.1 Co1.4	24.000	0.750	0.01301	8.029	0.00583	5.10530	5.11113
[Fe_absorber_Co_scatterer] Co1.1	12.000	0.750	0.01266	8.029	-0.05732	5.10530	5.04798
[Fe_absorber_Co_scatterer] Co1.1 Co1.4 Co1.1	12.000	0.750	0.01301	8.029	0.00583	5.10530	5.11113

Al-K; Ni-K

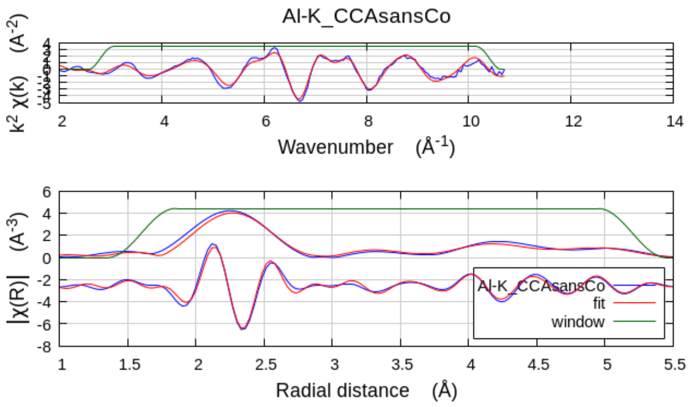

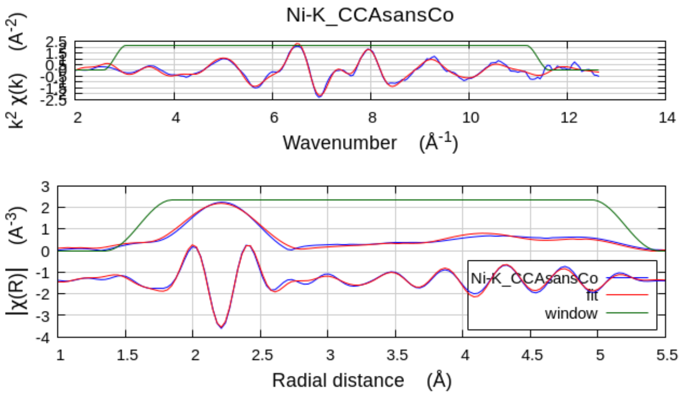

Independent points : 36.4765625

Number of variables : 15
Chi-square : 7156.0446976
Reduced chi-square : 333.2025178
R-factor : 0.0262210
Number of data sets : 2

guess parameters:

dEnot_Ni	= 7.02565320	# +/-	0.80130299	[7.02563]
dEnot_Al	= 7.18436111	# +/-	1.35964964	[7.18430]
dR1st	= -0.00808613	# +/-	0.00923193	[-0.00809]
dRAl2nd	= -0.01938289	# +/-	0.04756763	[-0.01938]
dRAl3rd	= 0.00772202	# +/-	0.02997848	[0.00772]
dRAl4th	= 0.06284380	# +/-	0.03733012	[0.06284]
x	= 2.53720409	# +/-	0.27131301	[2.53722]
dRNi1st	= -0.02377531	# +/-	0.00503077	[-0.02378]
dRNi2nd	= 0.00732078	# +/-	0.02051957	[0.00732]
dRNi3rd	= 0.00144109	# +/-	0.01404467	[0.00144]
dRNi4th	= -0.00790457	# +/-	0.01620906	[-0.00790]
thetaAl	= 429.33675101	# +/-	44.83603050	[429.33671]
thetaNi	= 286.99212704	# +/-	8.31628439	[286.99212]
ssNiAl	= 0.00499282	# +/-	0.00066211	[0.00499]
ssNiCo	= 0.00791718	# +/-	0.00055551	[0.00792]

```
set parameters:
  amp_Ni            =   0.77000000
  amp_Al            =   0.66000000
  temperature       = 300.00000000

: name                   = Al-K_CCAsansCo
: k-range                = 2.8 - 10.4
: dk                     = 0.5
: k-window               = Hanning
: k-weight               = 1,2,3
: R-range                = 1.6 - 5.2
: dR                     = 0.5
: R-window               = Hanning
: fitting space          = r
: background function    = no
: phase correction       = no
: background removal     = E0: 1557.481523, Rbkg: 1.0, range: [0:10.772], clamps: 0/24, kw: 2
: user-supplied epsilon_k = 0
: epsilon_k by k-weight  = 2.710e-03
: epsilon_r by k-weight  = 3.662e-01
: R-factor by k-weight   = 1 -> 0.04299,  2 -> 0.03237,  3 -> 0.03302

  name                                        N      S02     sigma^2   e0     delr     Reff     R
=======================================================================================================
[Al_absorber_Co_scatterer] Co1.1               12.000   0.660   0.00499   7.184 -0.00809  2.55270  2.54461
[Al_absorber_Co_scatterer] Co1.2                6.000   0.660   0.01087   7.184 -0.01938  3.61000  3.59062
[Al_absorber_Co_scatterer] Co1.1 Co1.1         48.000   0.660   0.00878   7.184 -0.00809  3.82900  3.82091
[Al_absorber_Co_scatterer] Co1.1 Co1.1         24.000   0.660   0.00854   7.184 -0.00809  4.35770  4.34961
[Al_absorber_Co_scatterer] Co1.1 Co1.2         48.000   0.660   0.01016   7.184 -0.01374  4.35770  4.34397
[Al_absorber_Co_scatterer] Co1.3               24.000   0.660   0.01115   7.184  0.00772  4.42130  4.42902
[Al_absorber_Co_scatterer] Co1.1 Co1.1         48.000   0.660   0.00781   7.184 -0.01617  4.76330  4.74713
[Al_absorber_Co_scatterer] Co1.1 Co1.3         96.000   0.660   0.01081   7.184 -0.00018  4.76330  4.76312
[Al_absorber_Co_scatterer] Co1.4               12.000   0.660   0.01127   7.184  0.06284  5.10530  5.16814
[Al_absorber_Co_scatterer] Co1.1 Co1.1         12.000   0.660   0.00697   7.184 -0.01617  5.10530  5.08913
[Al_absorber_Co_scatterer] Co1.1 Co1.4         24.000   0.660   0.01127   7.184  0.06284  5.10530  5.16814
[Al_absorber_Co_scatterer] Co1.1               12.000   0.660   0.00697   7.184 -0.01617  5.10530  5.08913
[Al_absorber_Co_scatterer] Co1.1 Co1.4 Co1.1   12.000   0.660   0.01127   7.184  0.06284  5.10530  5.16814

: name                   = Ni-K_CCAsansCo
: k-range                = 2.8 - 11.4
: dk                     = 0.5
: k-window               = Hanning
: k-weight               = 1,2,3
: R-range                = 1.6 - 5.2
: dR                     = 0.5
: R-window               = Hanning
: fitting space          = r
: background function    = no
: phase correction       = no
: background removal     = E0: 8333.4981, Rbkg: 1.0, range: [0.000:12.724], clamps: 0/24, kw: 2
: user-supplied epsilon_k = 0
: epsilon_k by k-weight  = 7.060e-04
: epsilon_r by k-weight  = 1.714e-01
: R-factor by k-weight   = 1 -> 0.01676,  2 -> 0.01409,  3 -> 0.01809
```

name	N	S02	sigma^2	e0	delr	Reff	R
[Ni_absorber_Al_scatterer] Al1.1	1.000	1.954	0.00499	7.026	-0.00809	2.55270	2.54461
[Ni_abs_Co_scatterer] Co1.1	1.000	7.286	0.00792	7.026	-0.02378	2.55270	2.52893
[Ni_abs_Co_scatterer] Co1.2	6.000	0.770	0.01441	7.026	0.00732	3.61000	3.61732
[Ni_abs_Co_scatterer] Co1.1 Co1.1	48.000	0.770	0.01303	7.026	-0.03352	3.82900	3.79548
[Ni_abs_Co_scatterer] Co1.1 Co1.1	24.000	0.770	0.01447	7.026	-0.03352	4.35770	4.32418
[Ni_abs_Co_scatterer] Co1.1 Co1.2	48.000	0.770	0.01448	7.026	-0.00823	4.35770	4.34947
[Ni_abs_Co_scatterer] Co1.3	24.000	0.770	0.01489	7.026	0.00144	4.42130	4.42274
[Ni_abs_Co_scatterer] Co1.1 Co1.1	48.000	0.770	0.01490	7.026	-0.04755	4.76330	4.71575
[Ni_abs_Co_scatterer] Co1.1 Co1.3	96.000	0.770	0.01492	7.026	-0.01117	4.76330	4.75213
[Ni_abs_Co_scatterer] Co1.4	12.000	0.770	0.01509	7.026	-0.00791	5.10530	5.09739
[Ni_abs_Co_scatterer] Co1.1 Co1.1	12.000	0.770	0.01506	7.026	-0.04755	5.10530	5.05775
[Ni_abs_Co_scatterer] Co1.1 Co1.4	24.000	0.770	0.01509	7.026	-0.00791	5.10530	5.09739
[Ni_abs_Co_scatterer] Co1.1	12.000	0.770	0.01506	7.026	-0.04755	5.10530	5.05775
[Ni_abs_Co_scatterer] Co1.1 Co1.4 Co1.1	12.000	0.770	0.01509	7.026	-0.00791	5.10530	5.09739

Al-K; Cu-K

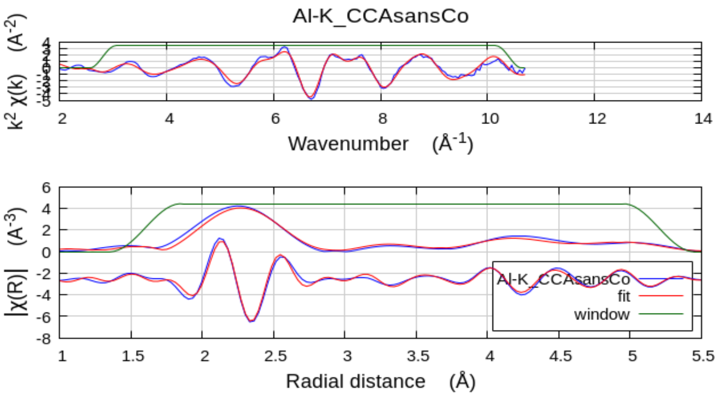

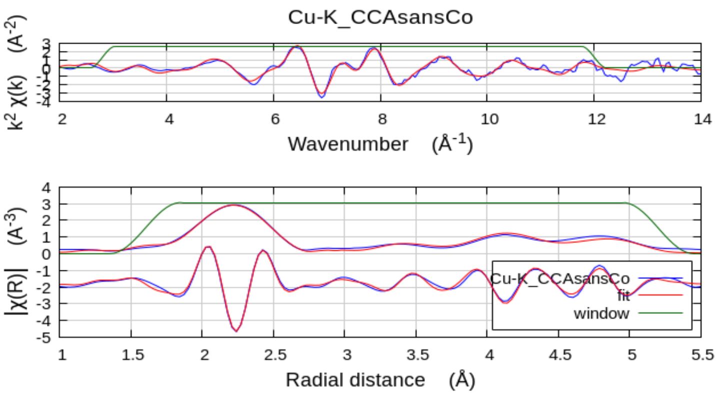

Independent points : 37.8359375
Number of variables : 15
Chi-square : 4032.5418513
Reduced chi-square : 176.5875323
R-factor : 0.0271723
Number of data sets : 2

guess parameters:
dEnot_Cu = 7.21555456 # +/- 1.53871946 [7.21531]
dR1st = -0.00812935 # +/- 0.00703951 [-0.00813]
ssCuAl = 0.00495505 # +/- 0.00048025 [0.00496]
dRCu1st = -0.01257433 # +/- 0.00895672 [-0.01258]
dRCu2nd = 0.00860880 # +/- 0.03333180 [0.00861]
dRCu3rd = 0.01968693 # +/- 0.02389549 [0.01968]
dRCu4th = 0.01028796 # +/- 0.02638364 [0.01028]
x = 2.62664585 # +/- 0.54874978 [2.62658]
dEnot_Al = 7.18434200 # +/- 1.02159488 [7.18440]
thetaCu = 320.19846024 # +/- 18.06673686 [320.19832]
thetaAl = 429.45013923 # +/- 32.67599595 [429.44965]
dR2nd = -0.01930973 # +/- 0.03468598 [-0.01931]
dR3rd = 0.00793030 # +/- 0.02190040 [0.00793]
dR4th = 0.06284620 # +/- 0.02730931 [0.06285]

ssCuCo = 0.00619790 # +/- 0.00090313 [0.00620]

set parameters:
amp_Cu = 0.79000000
amp_Al = 0.66000000
temperature = 300.00000000

: name = Al-K_CCAsansCo
: k-range = 2.8 - 10.4
: dk = 0.5
: k-window = Hanning
: k-weight = 1,2,3
: R-range = 1.6 - 5.2
: dR = 0.5
: R-window = Hanning
: fitting space = r
: background function = no
: phase correction = no
: background removal = E0: 1557.481523, Rbkg: 1.0, range: [0:10.772], clamps: 0/24, kw: 2
: user-supplied epsilon_k = 0
: epsilon_k by k-weight = 2.710e-03
: epsilon_r by k-weight = 3.662e-01
: R-factor by k-weight = 1 -> 0.04273, 2 -> 0.03237, 3 -> 0.03350

name	N	S02	sigma^2	e0	delr	Reff	R
[Al_absorber_Co_scatterer] Co1.1	12.000	0.660	0.00496	7.184	-0.00813	2.55270	2.54457
[Al_absorber_Co_scatterer] Co1.2	6.000	0.660	0.01086	7.184	-0.01931	3.61000	3.59069
[Al_absorber_Co_scatterer] Co1.1 Co1.1	48.000	0.660	0.00877	7.184	-0.00813	3.82900	3.82087
[Al_absorber_Co_scatterer] Co1.1 Co1.1	24.000	0.660	0.00853	7.184	-0.01626	4.35770	4.34144
[Al_absorber_Co_scatterer] Co1.1 Co1.2	48.000	0.660	0.01016	7.184	-0.01372	4.35770	4.34398
[Al_absorber_Co_scatterer] Co1.3	24.000	0.660	0.01114	7.184	0.00793	4.42130	4.42923
[Al_absorber_Co_scatterer] Co1.1 Co1.1	48.000	0.660	0.00781	7.184	-0.01626	4.76330	4.74704
[Al_absorber_Co_scatterer] Co1.1 Co1.3	96.000	0.660	0.01081	7.184	-0.00010	4.76330	4.76320
[Al_absorber_Co_scatterer] Co1.4	12.000	0.660	0.01126	7.184	0.06285	5.10530	5.16815
[Al_absorber_Co_scatterer] Co1.1 Co1.1	12.000	0.660	0.00696	7.184	-0.01626	5.10530	5.08904
[Al_absorber_Co_scatterer] Co1.1 Co1.4	24.000	0.660	0.01126	7.184	0.06285	5.10530	5.16815
[Al_absorber_Co_scatterer] Co1.1	12.000	0.660	0.00696	7.184	-0.01626	5.10530	5.08904
[Al_absorber_Co_scatterer] Co1.1 Co1.4 Co1.1	12.000	0.660	0.01126	7.184	0.06285	5.10530	5.16815

: name = Cu-K_CCAsansCo
: k-range = 2.8 - 12
: dk = 0.5
: k-window = Hanning
: k-weight = 1,2,3
: R-range = 1.6 - 5.2
: dR = 0.5
: R-window = Hanning
: fitting space = r
: background function = no
: phase correction = no
: background removal = E0: 8983.44336329588, Rbkg: 1.0, range: [0:14.4], clamps: 0/24, kw: 2
: user-supplied epsilon_k = 0
: epsilon_k by k-weight = 1.471e-03
: epsilon_r by k-weight = 6.332e-01

: R-factor by k-weight = 1 -> 0.02254, 2 -> 0.01587, 3 -> 0.01602

name	N	S02	sigma^2	e0	delr	Reff	R
[Cu_absorber_Al_scatterer] Al1.1	1.000	2.075	0.00496	7.216	-0.00813	2.55270	2.54457
[Cu_absorber_Co_scatterer] Co1.1	1.000	7.405	0.00620	7.216	-0.01257	2.55270	2.54013
[Cu_absorber_Co_scatterer] Co1.2	6.000	0.790	0.01123	7.216	0.00861	3.61000	3.61861
[Cu_absorber_Co_scatterer] Co1.1 Co1.1	48.000	0.790	0.01030	7.216	-0.01773	3.82900	3.81127
[Cu_absorber_Co_scatterer] Co1.1 Co1.1	24.000	0.790	0.01155	7.216	-0.01773	4.35770	4.33997
[Cu_absorber_Co_scatterer] Co1.1 Co1.2	48.000	0.790	0.01138	7.216	-0.00198	4.35770	4.35572
[Cu_absorber_Co_scatterer] Co1.3	24.000	0.790	0.01159	7.216	0.01969	4.42130	4.44099
[Cu_absorber_Co_scatterer] Co1.1 Co1.1	48.000	0.790	0.01198	7.216	-0.02515	4.76330	4.73815
[Cu_absorber_Co_scatterer] Co1.1 Co1.3	96.000	0.790	0.01167	7.216	0.00356	4.76330	4.76686
[Cu_absorber_Co_scatterer] Co1.4	12.000	0.790	0.01174	7.216	0.01029	5.10530	5.11559
[Cu_absorber_Co_scatterer] Co1.1 Co1.1	12.000	0.790	0.01218	7.216	-0.02515	5.10530	5.08015
[Cu_absorber_Co_scatterer] Co1.1 Co1.4	24.000	0.790	0.01174	7.216	0.01029	5.10530	5.11559
[Cu_absorber_Co_scatterer] Co1.1	12.000	0.790	0.01218	7.216	-0.02515	5.10530	5.08015
[Cu_absorber_Co_scatterer] Co1.1 Co1.4 Co1.1	12.000	0.790	0.01174	7.216	0.01029	5.10530	5.11559

CCA$_{sans}$Cu

Al-K; Cr-K

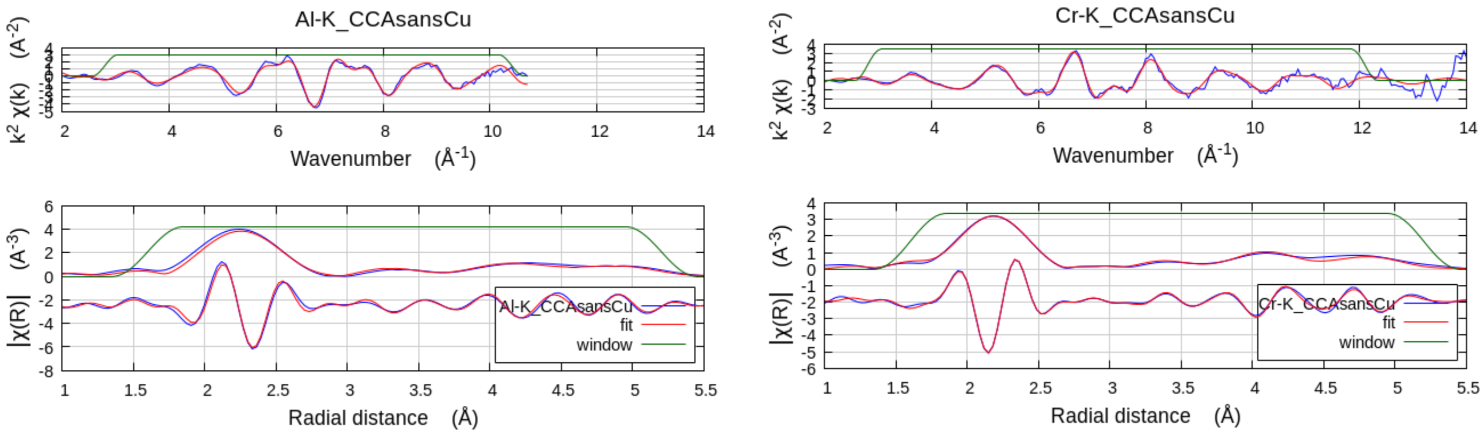

Independent points : 38.0625000
Number of variables : 15
Chi-square : 2218.4151222
Reduced chi-square : 96.1914416
R-factor : 0.0183623
Number of data sets : 2

guess parameters:
dEnot_Cr = 7.75363178 # +/- 1.25256512 [8.12369]
dEnot_Al = 6.37710043 # +/- 0.84946525 [6.37924]
dR1st = -0.01641908 # +/- 0.00592329 [-0.01640]
dRCr1st = -0.04246896 # +/- 0.00835842 [-0.04239]
ssCrCo = 0.00580358 # +/- 0.00088680 [0.00590]
ssAlCo = 0.00548448 # +/- 0.00039276 [0.00548]
x = 0.82666102 # +/- 0.68387187 [0.87040]
dRAl2nd = -0.04791590 # +/- 0.02777734 [-0.04790]

```
  dRAl3rd         =  -0.03527607    # +/-   0.01811076     [-0.03525]
  dRAl4th         =   0.00575518    # +/-   0.02157513     [0.00577]
  thetaAl         = 416.50792891    # +/-  23.44980465     [416.50817]
  thetaCr         = 344.12016833    # +/-  24.37211471     [342.04966]
  dRCr2nd         =  -0.01930263    # +/-   0.03834643     [-0.01905]
  dRCr3rd         =  -0.02105602    # +/-   0.02362669     [-0.02007]
  dRCr4th         =  -0.00249961    # +/-   0.01944415     [-0.00289]

set parameters:
  amp_Cr          =   0.57000000
  amp_Al          =   0.66000000
  temperature     = 300.00000000


: name                 = Al-K_CCAsansCu
: k-range              = 2.8 - 10.4
: dk                   = 0.5
: k-window             = Hanning
: k-weight             = 1,2,3
: R-range              = 1.6 - 5.2
: dR                   = 0.5
: R-window             = Hanning
: fitting space        = r
: background function  = no
: phase correction     = no
: background removal   = E0: 1556.971205, Rbkg: 1.0, range: [0:10.777], clamps: 0/24, kw: 2
: user-supplied epsilon_k = 0
: epsilon_k by k-weight   = 2.168e-03
: epsilon_r by k-weight   = 2.929e-01
: R-factor by k-weight    = 1 -> 0.03272,  2 -> 0.02585,  3 -> 0.02693

  name                                   N      S02     sigma^2   e0     delr     Reff     R
=======================================================================================
[Al_absorber_Co_scatterer] Co1.1              12.000   0.660   0.00548   6.377 -0.01642  2.55270  2.53628
[Al_absorber_Co_scatterer] Co1.2               6.000   0.660   0.01150   6.377 -0.04792  3.61000  3.56208
[Al_absorber_Co_scatterer] Co1.1 Co1.1        48.000   0.660   0.00929   6.377 -0.02315  3.82900  3.80585
[Al_absorber_Co_scatterer] Co1.1 Co1.1        24.000   0.660   0.00903   6.377 -0.02315  4.35770  4.33455
[Al_absorber_Co_scatterer] Co1.1 Co1.2        48.000   0.660   0.01075   6.377 -0.03217  4.35770  4.32553
[Al_absorber_Co_scatterer] Co1.3              24.000   0.660   0.01180   6.377 -0.03528  4.42130  4.38602
[Al_absorber_Co_scatterer] Co1.1 Co1.1        48.000   0.660   0.00827   6.377 -0.03284  4.76330  4.73046
[Al_absorber_Co_scatterer] Co1.1 Co1.3        96.000   0.660   0.01144   6.377 -0.02585  4.76330  4.73745
[Al_absorber_Co_scatterer] Co1.4              12.000   0.660   0.01193   6.377  0.00576  5.10530  5.11106
[Al_absorber_Co_scatterer] Co1.1 Co1.1        12.000   0.660   0.00738   6.377 -0.03284  5.10530  5.07246
[Al_absorber_Co_scatterer] Co1.1 Co1.4        24.000   0.660   0.01193   6.377  0.00576  5.10530  5.11106
[Al_absorber_Co_scatterer] Co1.1              12.000   0.660   0.00738   6.377 -0.03284  5.10530  5.07246
[Al_absorber_Co_scatterer] Co1.1 Co1.4 Co1.1  12.000   0.660   0.01193   6.377  0.00576  5.10530  5.11106


: name                 = Cr-K_CCAsansCu
: k-range              = 2.8 - 12.1
: dk                   = 0.5
: k-window             = Hanning
: k-weight             = 1,2,3
: R-range              = 1.6 - 5.2
: dR                   = 0.5
: R-window             = Hanning
```

: fitting space = r
: background function = no
: phase correction = no
: background removal = E0: 5990.22637056538, Rbkg: 1.0, range: [0:15.02], clamps: 0/24, kw: 2
: user-supplied epsilon_k = 0
: epsilon_k by k-weight = 2.236e-03
: epsilon_r by k-weight = 9.739e-01
: R-factor by k-weight = 1 -> 0.00616, 2 -> 0.00750, 3 -> 0.01101

name	N	S02	sigma^2	e0	delr	Reff	R
[Cr_absorber_Al_scatterer] Al1.1	1.000	0.471	0.00548	7.754	-0.01642	2.55270	2.53628
[Cr_absorber_Co_scatterer] Co1.1	1.000	6.369	0.00580	7.754	-0.04247	2.55270	2.51023
[Cr_absorber_Co_scatterer] Co1.2	6.000	0.570	0.01084	7.754	-0.01930	3.61000	3.59070
[Cr_absorber_Co_scatterer] Co1.1 Co1.1	48.000	0.570	0.00965	7.754	-0.05988	3.82900	3.76912
[Cr_absorber_Co_scatterer] Co1.1 Co1.1	24.000	0.570	0.01051	7.754	-0.05988	4.35770	4.29782
[Cr_absorber_Co_scatterer] Co1.1 Co1.2	48.000	0.570	0.01078	7.754	-0.03088	4.35770	4.32682
[Cr_absorber_Co_scatterer] Co1.3	24.000	0.570	0.01117	7.754	-0.02106	4.42130	4.40024
[Cr_absorber_Co_scatterer] Co1.1 Co1.1	48.000	0.570	0.01065	7.754	-0.08494	4.76330	4.67836
[Cr_absorber_Co_scatterer] Co1.1 Co1.3	96.000	0.570	0.01115	7.754	-0.03176	4.76330	4.73154
[Cr_absorber_Co_scatterer] Co1.4	12.000	0.570	0.01132	7.754	0.00000	5.10530	5.10530
[Cr_absorber_Co_scatterer] Co1.1 Co1.1	12.000	0.570	0.01061	7.754	-0.08494	5.10530	5.02036
[Cr_absorber_Co_scatterer] Co1.1 Co1.4	24.000	0.570	0.01132	7.754	-0.00250	5.10530	5.10280
[Cr_absorber_Co_scatterer] Co1.1	12.000	0.570	0.01061	7.754	-0.08494	5.10530	5.02036
[Cr_absorber_Co_scatterer] Co1.1 Co1.4 Co1.1	12.000	0.570	0.01132	7.754	-0.00250	5.10530	5.10280

Al-K; Fe-K

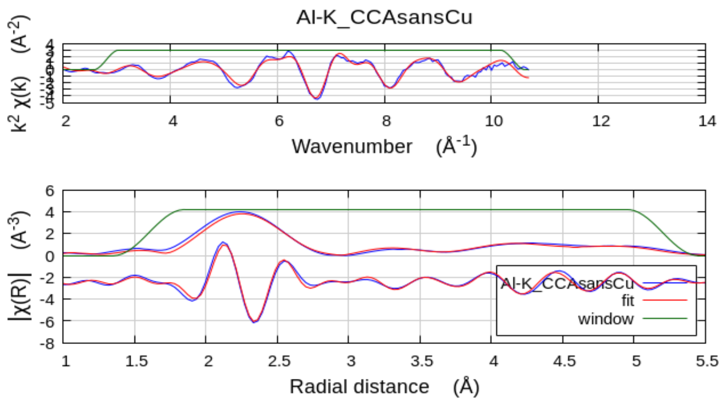

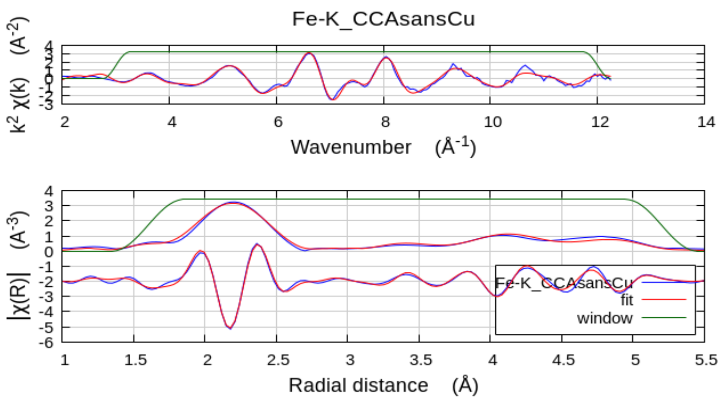

Independent points : 37.3828125
Number of variables : 15
Chi-square : 3041.7718496
Reduced chi-square : 135.8976603
R-factor : 0.0257311
Number of data sets : 2

guess parameters:
dEnot_Fe = 8.30419360 # +/- 1.17744895 [7.83416]
dEnot_Al = 6.33655356 # +/- 1.01149765 [6.34314]
dRFe1st = -0.03600458 # +/- 0.00757566 [-0.03956]
dR1st = -0.01534077 # +/- 0.00708835 [-0.01527]

```
  ssFeCo           =   0.00681963    # +/-   0.00079462     [0.00700]
  ssAlCo           =   0.00549550    # +/-   0.00046819     [0.00550]
  x                =   1.65098262    # +/-   0.48738378     [2.19628]
  dRAl2nd          =  -0.04943138    # +/-   0.03281641     [-0.04954]
  dRAl3rd          =  -0.03690619    # +/-   0.02099116     [-0.03642]
  dRAl4th          =  -0.00776934    # +/-   0.03619952     [-0.00769]
  thetaAl          = 419.11054920    # +/-  35.41807038     [417.67343]
  thetaFe          = 320.45478768    # +/-  20.56336629     [306.86288]
  dRFe2nd          =  -0.00900663    # +/-   0.03052849     [-0.00639]
  dRFe3rd          =  -0.01752254    # +/-   0.01933374     [-0.01868]
  dRFe4th          =  -0.02570422    # +/-   0.03310756     [-0.02991]

set parameters:
  amp_Fe           =   0.75000000
  amp_Al           =   0.66000000
  temperature      = 300.00000000

: name                    = Al-K_CCAsansCu
: k-range                 = 2.8 - 10.4
: dk                      = 0.5
: k-window                = Hanning
: k-weight                = 1,2,3
: R-range                 = 1.6 - 5.2
: dR                      = 0.5
: R-window                = Hanning
: fitting space           = r
: background function     = no
: phase correction        = no
: background removal      = E0: 1556.971205, Rbkg: 1.0, range: [0:10.777], clamps: 0/24, kw: 2
: user-supplied epsilon_k = 0
: epsilon_k by k-weight   = 2.168e-03
: epsilon_r by k-weight   = 2.929e-01
: R-factor by k-weight    = 1 -> 0.03449,  2 -> 0.02806,  3 -> 0.02934

  name                                 N       S02     sigma^2   e0     delr     Reff     R
=======================================================================================
[Al_absorber_Co_scatterer] Co1.1               12.000   0.660   0.00549   6.337 -0.01534  2.55270  2.53736
[Al_absorber_Co_scatterer] Co1.2                6.000   0.660   0.01137   6.337 -0.04943  3.61000  3.56057
[Al_absorber_Co_scatterer] Co1.1 Co1.1         48.000   0.660   0.00918   6.337 -0.02163  3.82900  3.80737
[Al_absorber_Co_scatterer] Co1.1 Co1.1         24.000   0.660   0.00893   6.337 -0.02163  4.35770  4.33607
[Al_absorber_Co_scatterer] Co1.1 Co1.2         48.000   0.660   0.01063   6.337 -0.03239  4.35770  4.32531
[Al_absorber_Co_scatterer] Co1.3               24.000   0.660   0.01166   6.337 -0.03691  4.42130  4.38439
[Al_absorber_Co_scatterer] Co1.1 Co1.1         48.000   0.660   0.00817   6.337 -0.03068  4.76330  4.73262
[Al_absorber_Co_scatterer] Co1.1 Co1.3         96.000   0.660   0.01131   6.337 -0.02612  4.76330  4.73718
[Al_absorber_Co_scatterer] Co1.4               12.000   0.660   0.01179   6.337 -0.00777  5.10530  5.09753
[Al_absorber_Co_scatterer] Co1.1 Co1.1         12.000   0.660   0.00729   6.337 -0.03068  5.10530  5.07462
[Al_absorber_Co_scatterer] Co1.1 Co1.4         24.000   0.660   0.01179   6.337 -0.01155  5.10530  5.09374
[Al_absorber_Co_scatterer] Co1.1               12.000   0.660   0.00729   6.337 -0.03068  5.10530  5.07462
[Al_absorber_Co_scatterer] Co1.1 Co1.4 Co1.1   12.000   0.660   0.01179   6.337 -0.00777  5.10530  5.09753


: name                    = Fe-K_CCAsansCu
: k-range                 = 3 - 12
: dk                      = 0.5
: k-window                = Hanning
: k-weight                = 1,2,3
```

: R-range = 1.6 - 5.2
: dR = 0.5
: R-window = Hanning
: fitting space = r
: background function = no
: phase correction = no
: background removal = E0: 7112.52510516437, Rbkg: 1.0, range: [0:12.32], clamps: 0/24, kw: 2
: user-supplied epsilon_k = 0
: epsilon_k by k-weight = 1.621e-03
: epsilon_r by k-weight = 3.516e-01
: R-factor by k-weight = 1 -> 0.01387, 2 -> 0.01951, 3 -> 0.02912

name	N	S02	sigma^2	e0	delr	Reff	R
[Fe_absorber_Co_scatterer] Co1.1	1.000	7.762	0.00682	8.304	-0.03601	2.55270	2.51670
[Fe_absorber_Al_scatterer] Al1.1	1.000	1.238	0.00549	8.304	-0.01534	2.55270	2.53736
[Fe_absorber_Co_scatterer] Co1.2	6.000	0.750	0.01196	8.304	-0.00901	3.61000	3.60099
[Fe_absorber_Co_scatterer] Co1.1 Co1.1	48.000	0.750	0.01075	8.304	-0.05077	3.82900	3.77823
[Fe_absorber_Co_scatterer] Co1.1 Co1.1	24.000	0.750	0.01184	8.304	-0.05077	4.35770	4.30693
[Fe_absorber_Co_scatterer] Co1.1 Co1.2	48.000	0.750	0.01197	8.304	-0.02251	4.35770	4.33519
[Fe_absorber_Co_scatterer] Co1.3	24.000	0.750	0.01234	8.304	-0.01752	4.42130	4.40378
[Fe_absorber_Co_scatterer] Co1.1 Co1.1	48.000	0.750	0.01211	8.304	-0.07201	4.76330	4.69129
[Fe_absorber_Co_scatterer] Co1.1 Co1.3	96.000	0.750	0.01235	8.304	-0.02676	4.76330	4.73654
[Fe_absorber_Co_scatterer] Co1.4	12.000	0.750	0.01250	8.304	-0.02570	5.10530	5.07960
[Fe_absorber_Co_scatterer] Co1.1 Co1.1	12.000	0.750	0.01217	8.304	-0.07201	5.10530	5.03329
[Fe_absorber_Co_scatterer] Co1.1 Co1.4	24.000	0.750	0.01250	8.304	-0.03085	5.10530	5.07445
[Fe_absorber_Co_scatterer] Co1.1	12.000	0.750	0.01217	8.304	-0.07201	5.10530	5.03329
[Fe_absorber_Co_scatterer] Co1.1 Co1.4 Co1.1	12.000	0.750	0.01250	8.304	-0.02570	5.10530	5.07960

Al-K; Co-K

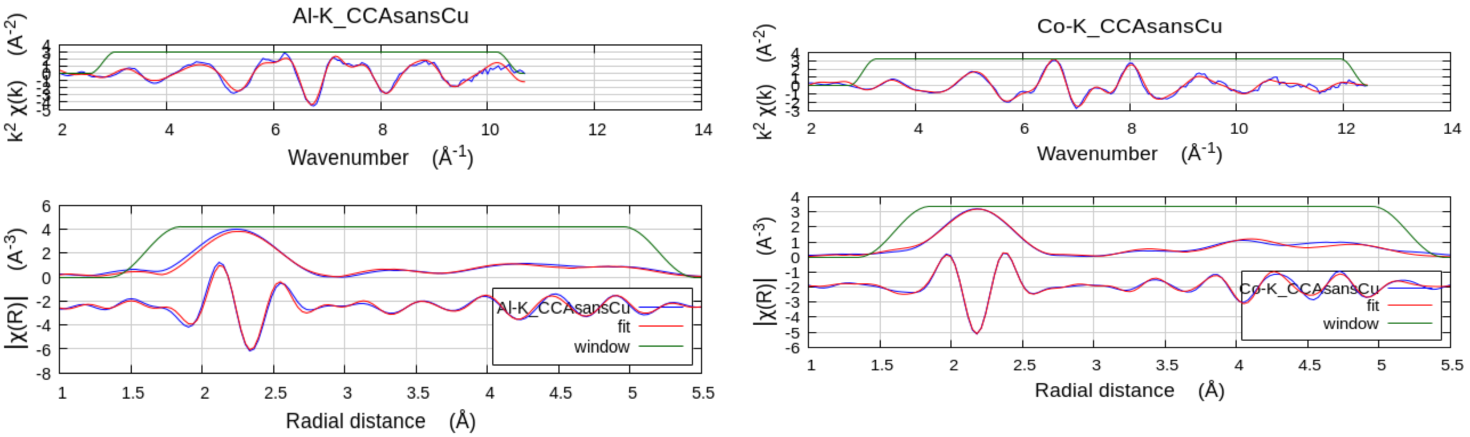

Independent points : 37.8359375
Number of variables : 15
Chi-square : 2798.9257938
Reduced chi-square : 122.5667128
R-factor : 0.0222998
Number of data sets : 2

guess parameters:

```
  dEnot_Co          =   7.71383597    # +/-   0.93250024     [8.08841]
  dEnot_Al          =   6.41524229    # +/-   0.95747237     [6.17436]
  dRCo1st           =  -0.04368481    # +/-   0.00640327     [-0.04149]
  dR1st             =  -0.01609956    # +/-   0.00667595     [-0.01704]
  ssCoCo            =   0.00775542    # +/-   0.00072162     [0.00765]
  ssAlCo            =   0.00548488    # +/-   0.00044349     [0.00592]
  x                 =   1.25975365    # +/-   0.41892342     [1.29]
  dRCo2nd           =  -0.00368173    # +/-   0.02392266     [0.00455]
  dRCo3rd           =  -0.02521696    # +/-   0.01524563     [-0.01877]
  dRCo4th           =  -0.01909283    # +/-   0.01804764     [-0.01211]
  thetaAl           = 416.51564039    # +/-  26.46952005     [403.58968]
  thetaCo           = 320.12147582    # +/-  13.19870928     [323.10240]
  dRAl2nd           =  -0.04768109    # +/-   0.03135260     [-0.05063]
  dRAl3rd           =  -0.03476354    # +/-   0.02044256     [-0.03602]
  dRAl4th           =   0.00609800    # +/-   0.02435070     [0.00624]

set parameters:
  amp_Co            =   0.78000000
  amp_Al            =   0.66000000
  temperature       = 300.00000000

: name                     = Al-K_CCAsansCu
: k-range                  = 2.8 - 10.4
: dk                       = 0.5
: k-window                 = Hanning
: k-weight                 = 1,2,3
: R-range                  = 1.6 - 5.2
: dR                       = 0.5
: R-window                 = Hanning
: fitting space            = r
: background function      = no
: phase correction         = no
: background removal       = E0: 1556.971205, Rbkg: 1.0, range: [0:10.777], clamps: 0/24, kw: 2
: user-supplied epsilon_k = 0
: epsilon_k by k-weight    = 2.168e-03
: epsilon_r by k-weight    = 2.929e-01
: R-factor by k-weight     = 1 -> 0.03265,  2 -> 0.02590,  3 -> 0.02703

  name                                    N      S02     sigma^2   e0     delr     Reff     R
=======================================================================================
[Al_absorber_Co_scatterer] Co1.1                12.000   0.660   0.00549   6.415 -0.01610  2.55270  2.53660
[Al_absorber_Co_scatterer] Co1.2                 6.000   0.660   0.01150   6.415 -0.04768  3.61000  3.56232
[Al_absorber_Co_scatterer] Co1.1 Co1.1          48.000   0.660   0.00928   6.415 -0.02270  3.82900  3.80630
[Al_absorber_Co_scatterer] Co1.1 Co1.1          24.000   0.660   0.00903   6.415 -0.02270  4.35770  4.33500
[Al_absorber_Co_scatterer] Co1.1 Co1.2          48.000   0.660   0.01075   6.415 -0.03189  4.35770  4.32581
[Al_absorber_Co_scatterer] Co1.3                24.000   0.660   0.01180   6.415 -0.03476  4.42130  4.38654
[Al_absorber_Co_scatterer] Co1.1 Co1.1          48.000   0.660   0.00827   6.415 -0.03220  4.76330  4.73110
[Al_absorber_Co_scatterer] Co1.1 Co1.3          96.000   0.660   0.01144   6.415 -0.02543  4.76330  4.73787
[Al_absorber_Co_scatterer] Co1.4                12.000   0.660   0.01193   6.415  0.00610  5.10530  5.11140
[Al_absorber_Co_scatterer] Co1.1 Co1.1          12.000   0.660   0.00738   6.415 -0.03220  5.10530  5.07310
[Al_absorber_Co_scatterer] Co1.1 Co1.4          24.000   0.660   0.01193   6.415  0.00610  5.10530  5.11140
[Al_absorber_Co_scatterer] Co1.1                12.000   0.660   0.00738   6.415 -0.03220  5.10530  5.07310
[Al_absorber_Co_scatterer] Co1.1 Co1.4 Co1.1    12.000   0.660   0.01193   6.415  0.00610  5.10530  5.11140

: name                     = Co-K_CCAsansCu
: k-range                  = 3 - 12.2
```

: dk = 0.5
: k-window = Hanning
: k-weight = 1,2,3
: R-range = 1.6 - 5.2
: dR = 0.5
: R-window = Hanning
: fitting space = r
: background function = no
: phase correction = no
: background removal = E0: 7710.80025086635, Rbkg: 1.0, range: [0:12.506], clamps: 0/24, kw: 2
: user-supplied epsilon_k = 0
: epsilon_k by k-weight = 1.396e-03
: epsilon_r by k-weight = 3.206e-01
: R-factor by k-weight = 1 -> 0.00754, 2 -> 0.01363, 3 -> 0.02705

name	N	S02	sigma^2	e0	delr	Reff	R
[Co_absorber_Co_scatterer] Co1.1	1.000	8.377	0.00775	7.714	-0.04369	2.55270	2.50902
[Co_absorber_Al_scatterer] Al1.1	1.000	0.983	0.00549	7.714	-0.01610	2.55270	2.53660
[Co_absorber_Co_scatterer] Co1.2	6.000	0.780	0.01166	7.714	-0.00368	3.61000	3.60632
[Co_absorber_Co_scatterer] Co1.1 Co1.1	48.000	0.780	0.01057	7.714	-0.06160	3.82900	3.76740
[Co_absorber_Co_scatterer] Co1.1 Co1.1	24.000	0.780	0.01174	7.714	-0.06160	4.35770	4.29610
[Co_absorber_Co_scatterer] Co1.1 Co1.2	48.000	0.780	0.01174	7.714	-0.02368	4.35770	4.33402
[Co_absorber_Co_scatterer] Co1.3	24.000	0.780	0.01203	7.714	-0.02522	4.42130	4.39608
[Co_absorber_Co_scatterer] Co1.1 Co1.1	48.000	0.780	0.01207	7.714	-0.08737	4.76330	4.67593
[Co_absorber_Co_scatterer] Co1.1 Co1.3	96.000	0.780	0.01207	7.714	-0.03445	4.76330	4.72885
[Co_absorber_Co_scatterer] Co1.4	12.000	0.780	0.01219	7.714	-0.01909	5.10530	5.08621
[Co_absorber_Co_scatterer] Co1.1 Co1.1	12.000	0.780	0.01219	7.714	-0.08737	5.10530	5.01793
[Co_absorber_Co_scatterer] Co1.1 Co1.4	24.000	0.780	0.01219	7.714	-0.01909	5.10530	5.08621
[Co_absorber_Co_scatterer] Co1.1	12.000	0.780	0.01219	7.714	-0.08737	5.10530	5.01793
[Co_absorber_Co_scatterer] Co1.1 Co1.4 Co1.1	12.000	0.780	0.01219	7.714	-0.01909	5.10530	5.08621

Al-K; Ni-K

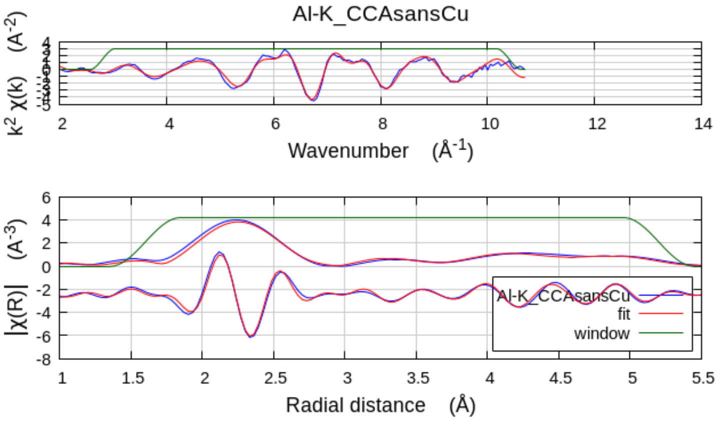

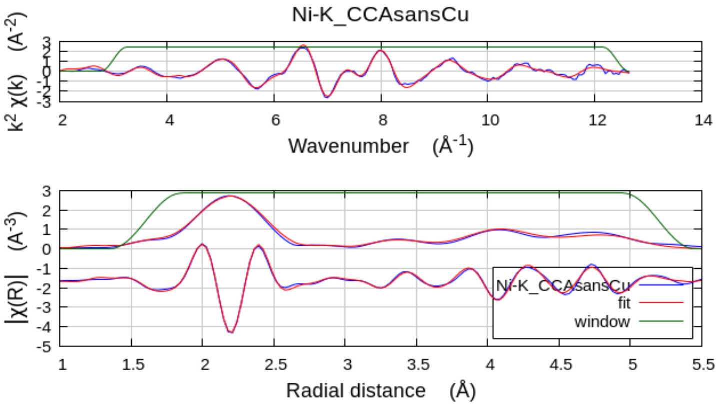

Independent points : 38.2890625
Number of variables : 15
Chi-square : 2754.2338059
Reduced chi-square : 118.2629746
R-factor : 0.0198004
Number of data sets : 2

```
guess parameters:
  dEnot_Ni         =   6.50414795    # +/-   0.93096065     [6.52003]
  dEnot_Al         =   6.37048994    # +/-   0.93534440     [6.36914]
  dRNi1st          =  -0.03528851    # +/-   0.00556689     [-0.03514]
  dR1st            =  -0.01647069    # +/-   0.00650730     [-0.01648]
  ssAlCo           =   0.00549546    # +/-   0.00043603     [0.00549]
  ssNiCo           =   0.00746576    # +/-   0.00057495     [0.00748]
  x                =   2.19557046    # +/-   0.32060398     [2.19024]
  thetaNi          = 305.87097204    # +/-   9.88815691     [306.01669]
  thetaAl          = 416.50318038    # +/-  26.00095417     [416.50331]
  dRAl2nd          =  -0.04797173    # +/-   0.03078941     [-0.04798]
  dRAl3rd          =  -0.03535162    # +/-   0.02003928     [-0.03537]
  dRAl4th          =   0.00570024    # +/-   0.02389864     [0.00569]
  dRNi2nd          =  -0.01033553    # +/-   0.02083593     [-0.01010]
  dRNi3rd          =  -0.02468236    # +/-   0.01434397     [-0.02457]
  dRNi4th          =  -0.03385049    # +/-   0.01675028     [-0.03381]

set parameters:
  amp_Ni           =   0.77000000
  amp_Al           =   0.66000000
  temperature      = 300.00000000

: name                     = Al-K_CCAsansCu
: k-range                  = 2.8 - 10.4
: dk                       = 0.5
: k-window                 = Hanning
: k-weight                 = 1,2,3
: R-range                  = 1.6 - 5.2
: dR                       = 0.5
: R-window                 = Hanning
: fitting space            = r
: background function      = no
: phase correction         = no
: background removal       = E0: 1556.971205, Rbkg: 1.0, range: [0:10.777], clamps: 0/24, kw: 2
: user-supplied epsilon_k = 0
: epsilon_k by k-weight   = 2.168e-03
: epsilon_r by k-weight   = 2.929e-01
: R-factor by k-weight    = 1 -> 0.03281,  2 -> 0.02583,  3 -> 0.02677

  name                                       N      S02     sigma^2   e0     delr     Reff     R
=======================================================================================================
[Al_absorber_Co_scatterer] Co1.1               12.000   0.660   0.00549   6.370 -0.01647  2.55270  2.53623
[Al_absorber_Co_scatterer] Co1.2                6.000   0.660   0.01150   6.370 -0.04797  3.61000  3.56203
[Al_absorber_Co_scatterer] Co1.1 Co1.1         48.000   0.660   0.00929   6.370 -0.02322  3.82900  3.80578
[Al_absorber_Co_scatterer] Co1.1 Co1.1         24.000   0.660   0.00903   6.370 -0.02322  4.35770  4.33448
[Al_absorber_Co_scatterer] Co1.1 Co1.2         48.000   0.660   0.01075   6.370 -0.03222  4.35770  4.32548
[Al_absorber_Co_scatterer] Co1.3               24.000   0.660   0.01180   6.370 -0.03535  4.42130  4.38595
[Al_absorber_Co_scatterer] Co1.1 Co1.1         48.000   0.660   0.00827   6.370 -0.03294  4.76330  4.73036
[Al_absorber_Co_scatterer] Co1.1 Co1.3         96.000   0.660   0.01144   6.370 -0.02591  4.76330  4.73739
[Al_absorber_Co_scatterer] Co1.4               12.000   0.660   0.01193   6.370  0.00570  5.10530  5.11100
[Al_absorber_Co_scatterer] Co1.1 Co1.1         12.000   0.660   0.00738   6.370 -0.03294  5.10530  5.07236
[Al_absorber_Co_scatterer] Co1.1 Co1.4         24.000   0.660   0.01193   6.370  0.00570  5.10530  5.11100
[Al_absorber_Co_scatterer] Co1.1               12.000   0.660   0.00738   6.370 -0.03294  5.10530  5.07236
[Al_absorber_Co_scatterer] Co1.1 Co1.4 Co1.1   12.000   0.660   0.01193   6.370  0.00570  5.10530  5.11100
```

```
: name                    = Ni-K_CCAsansCu
: k-range                 = 3.000 - 12.4
: dk                      = 0.5
: k-window                = Hanning
: k-weight                = 1,2,3
: R-range                 = 1.6 - 5.2
: dR                      = 0.5
: R-window                = Hanning
: fitting space           = r
: background function     = no
: phase correction        = no
: background removal      = E0: 8333.4981, Rbkg: 1.0, range: [0:12.724], clamps: 0/24, kw: 2
: user-supplied epsilon_k = 0
: epsilon_k by k-weight   = 1.075e-03
: epsilon_r by k-weight   = 2.609e-01
: R-factor by k-weight    = 1 -> 0.00760,  2 -> 0.00939,  3 -> 0.01641

  name                                        N      S02    sigma^2   e0     delr     Reff     R
======================================================================================================
[Ni_absorber_Co_scatterer] Co1.1               1.000   7.549   0.00747   6.504 -0.03529  2.55270  2.51741
[Ni_absorber_Al_scatterer] Al1.1               1.000   1.691   0.00549   6.504 -0.01647  2.55270  2.53623
[Ni_absorber_Co_scatterer] Co1.2               6.000   0.770   0.01274   6.504 -0.01034  3.61000  3.59966
[Ni_absorber_Co_scatterer] Co1.1 Co1.1        48.000   0.770   0.01153   6.504 -0.04976  3.82900  3.77924
[Ni_absorber_Co_scatterer] Co1.1 Co1.1        24.000   0.770   0.01280   6.504 -0.04976  4.35770  4.30794
[Ni_absorber_Co_scatterer] Co1.1 Co1.2        48.000   0.770   0.01281   6.504 -0.02281  4.35770  4.33489
[Ni_absorber_Co_scatterer] Co1.3              24.000   0.770   0.01316   6.504 -0.02468  4.42130  4.39662
[Ni_absorber_Co_scatterer] Co1.1 Co1.1        48.000   0.770   0.01317   6.504 -0.07058  4.76330  4.69272
[Ni_absorber_Co_scatterer] Co1.1 Co1.3        96.000   0.770   0.01319   6.504 -0.02999  4.76330  4.73332
[Ni_absorber_Co_scatterer] Co1.4              12.000   0.770   0.01333   6.504 -0.03385  5.10530  5.07145
[Ni_absorber_Co_scatterer] Co1.1 Co1.1        12.000   0.770   0.01331   6.504 -0.07058  5.10530  5.03472
[Ni_absorber_Co_scatterer] Co1.1 Co1.4        24.000   0.770   0.01333   6.504 -0.03385  5.10530  5.07145
[Ni_absorber_Co_scatterer] Co1.1              12.000   0.770   0.01331   6.504 -0.07058  5.10530  5.03472
[Ni_absorber_Co_scatterer] Co1.1 Co1.4 Co1.1  12.000   0.770   0.01333   6.504 -0.03385  5.10530  5.07145
```

Ternaries and quaternaries

$Al_4Co_{48}Ni_{48}$

Al-K; Co-K

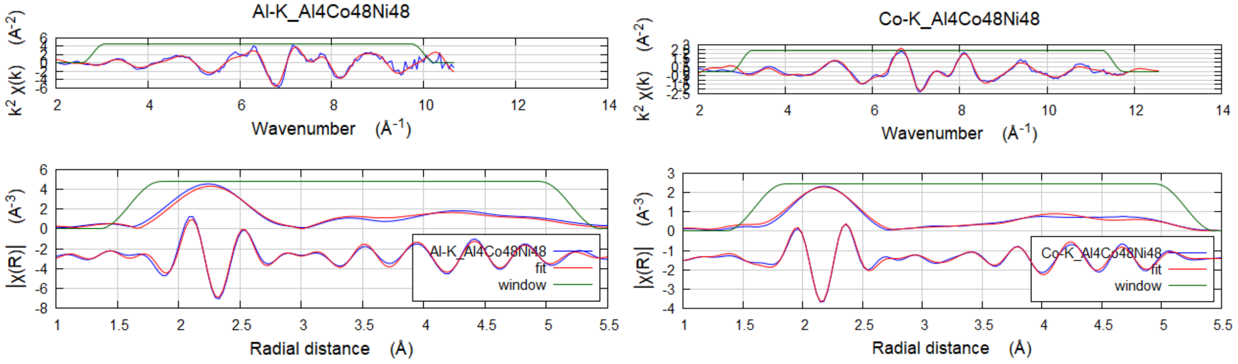


```
Independent points          : 35.3437500
Number of variables         : 15
Chi-square                  : 1300.6754904
Reduced chi-square          : 63.9348935
R-factor                    : 0.0189750
Number of data sets         : 2


guess parameters:
  dEnotCo          =   8.98904410    # +/-    0.79206696     [8.99478]
  dEnotAl          =   8.89807895    # +/-    1.76784961     [8.83225]
  dR1st            =   0.02581396    # +/-    0.01183029     [0.02554]
  ss1st            =   0.00400635    # +/-    0.00094184     [0.00402]
  ssCo1st          =   0.00752891    # +/-    0.00048816     [0.00753]
  dRAl2nd          =  -0.00781837    # +/-    0.03986392     [-0.00793]
  dRAl3rd          =   0.04019160    # +/-    0.02896200     [0.03949]
  dRAl4th          =   0.06232749    # +/-    0.03027162     [0.06149]
  dRCo1st          =  -0.00723781    # +/-    0.00455241     [-0.00727]
  dRCo2nd          =   0.03131735    # +/-    0.01698862     [0.03136]
  dRCo3rd          =   0.03058671    # +/-    0.01250725     [0.03068]
  dRCo4th          =   0.02699263    # +/-    0.01414490     [0.02709]
  thetaAl          = 631.57799652    # +/- 127.06749186     [630.94637]
  thetaCo          = 290.84907412    # +/-    6.73291177     [290.85189]
  x                =   2.66297323    # +/-    0.23277247     [2.66300]

set parameters:
  ampCo            =   0.78000000
  ampAl            =   0.66000000
  temperature      = 300.00000000


: name                      = Al-K_Al4Co48Ni48
: k-range                   = 2.8 - 10
: dk                        = 0.5
: k-window                  = Hanning
: k-weight                  = 1,2,3
: R-range                   = 1.6 - 5.2
: dR                        = 0.5
: R-window                  = Hanning
: fitting space             = r
: background function       = no
: phase correction          = no
: background removal        = E0: 1556.648074, Rbkg: 1.0, range: [0:10.729], clamps: 0/24, kw: 2
: user-supplied epsilon_k = 0
: epsilon_k by k-weight     = 9.643e-003
: epsilon_r by k-weight     = 1.282e+000
: R-factor by k-weight      = 1 -> 0.02445,  2 -> 0.01919,  3 -> 0.01710
```

name	N	S02	sigma^2	e0	delr	Reff	R
[feff_Al_abs_Co_scatt] Co1.1	12.000	0.660	0.00401	8.898	0.02581	2.50320	2.52901

[feff_Al_abs_Co_scatt] Co1.2	6.000	0.660	0.00541	8.898	-0.00782	3.54000	3.53218
[feff_Al_abs_Co_scatt] Co1.1 Co1.1	48.000	0.660	0.00441	8.898	0.03640	3.75470	3.79110
[feff_Al_abs_Co_scatt] Co1.1 Co1.1	24.000	0.660	0.00427	8.898	0.03640	4.27320	4.30960
[feff_Al_abs_Co_scatt] Co1.1 Co1.2	48.000	0.660	0.00508	8.898	0.00900	4.27320	4.28220
[feff_Al_abs_Co_scatt] Co1.3	24.000	0.660	0.00552	8.898	0.04019	4.33560	4.37579
[feff_Al_abs_Co_scatt] Co1.1 Co1.1	48.000	0.660	0.00389	8.898	0.05163	4.67100	4.72263
[feff_Al_abs_Co_scatt] Co1.1 Co1.3	96.000	0.660	0.00537	8.898	0.03300	4.67100	4.70400
[feff_Al_abs_Co_scatt] Co1.4	12.000	0.660	0.00556	8.898	0.06233	5.00630	5.06863
[feff_Al_abs_Co_scatt] Co1.1 Co1.4	24.000	0.660	0.00556	8.898	0.06233	5.00630	5.06863
[feff_Al_abs_Co_scatt] Co1.1 Co1.4 Co1.1	12.000	0.660	0.00556	8.898	0.06233	5.00630	5.06863

```
: name                    = Co-K_Al4Co48Ni48
: k-range                 = 3.000 - 11.5
: dk                      = 0.5
: k-window                = Hanning
: k-weight                = 1,2,3
: R-range                 = 1.6 - 5.2
: dR                      = 0.5
: R-window                = Hanning
: fitting space           = r
: background function     = no
: phase correction        = no
: background removal      = E0: 7709, Rbkg: 1.0, range: [0.000:11.75], clamps: 0/24, kw: 2
: user-supplied epsilon_k = 0
: epsilon_k by k-weight   = 8.595e-004
: epsilon_r by k-weight   = 2.029e-001
: R-factor by k-weight    = 1 -> 0.01713,  2 -> 0.01529,  3 -> 0.02068
```

name	N	S02	sigma^2	e0	delr	Reff	R
[feff_Co_abs_Al_scatt] Al1.1	1.000	2.077	0.00401	8.989	0.02581	2.50320	2.52901
[feff_Co_abs_Co_scatt] Co1.1	1.000	7.283	0.00753	8.989	-0.00724	2.50320	2.49596
[feff_Co_abs_Co_scatt] Co1.2	6.000	0.780	0.01402	8.989	0.03132	3.54000	3.57132
[feff_Co_abs_Co_scatt] Co1.1 Co1.1	48.000	0.780	0.01269	8.989	-0.01021	3.75470	3.74450
[feff_Co_abs_Co_scatt] Co1.1 Co1.1	24.000	0.780	0.01410	8.989	-0.01021	4.27320	4.26300
[feff_Co_abs_Co_scatt] Co1.1 Co1.2	48.000	0.780	0.01410	8.989	0.01204	4.27320	4.28524
[feff_Co_abs_Co_scatt] Co1.3	24.000	0.780	0.01448	8.989	0.03059	4.33560	4.36619
[feff_Co_abs_Co_scatt] Co1.1 Co1.1	48.000	0.780	0.01452	8.989	-0.01448	4.67100	4.65652
[feff_Co_abs_Co_scatt] Co1.1 Co1.3	96.000	0.780	0.01452	8.989	0.01167	4.67100	4.68267
[feff_Co_abs_Co_scatt] Co1.4	12.000	0.780	0.01467	8.989	0.02699	5.00630	5.03329
[feff_Co_abs_Co_scatt] Co1.1 Co1.1	12.000	0.780	0.01467	8.989	-0.01448	5.00630	4.99182
[feff_Co_abs_Co_scatt] Co1.1 Co1.4	24.000	0.780	0.01467	8.989	0.02699	5.00630	5.03329
[feff_Co_abs_Co_scatt] Co1.1	12.000	0.780	0.01467	8.989	-0.01448	5.00630	4.99182
[feff_Co_abs_Co_scatt] Co1.1 Co1.4 Co1.1	12.000	0.780	0.01467	8.989	0.02699	5.00630	5.03329

Al-K; Ni-K

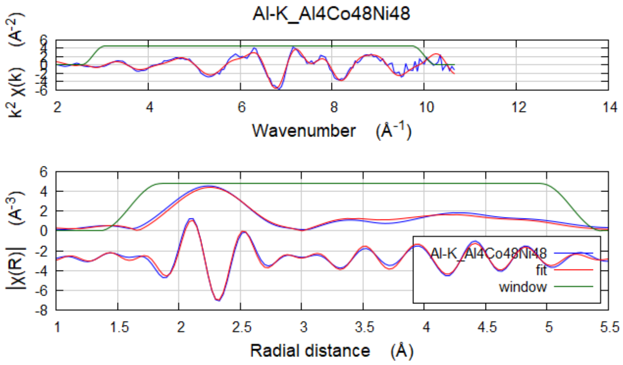

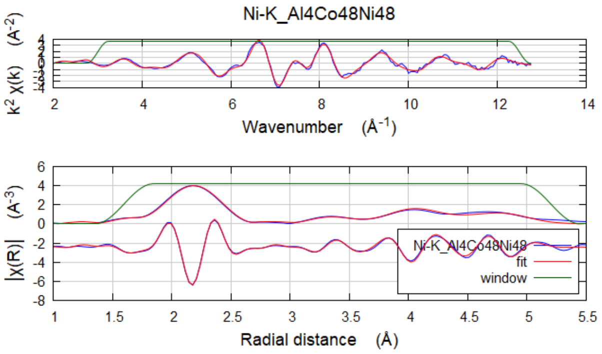


```
Independent points         : 37.6093750
Number of variables        : 15
Chi-square                 : 630.9161812
Reduced chi-square         : 27.9050695
R-factor                   : 0.0137968
Number of data sets        : 2
```

```
guess parameters:
  dEnotNi          =   8.17024669    # +/-   0.47771370     [8.63484]
  dEnotAl          =   8.57546765    # +/-   1.25584849     [8.29077]
  dR1st            =   0.02297734    # +/-   0.00863504     [0.02067]
  ss1st            =   0.00382639    # +/-   0.00060918     [0.00384]
  ssNi1st          =   0.00543853    # +/-   0.00031478     [0.00544]
  dRAl2nd          =  -0.01063132    # +/-   0.02645753     [-0.01235]
  dRAl3rd          =   0.03652219    # +/-   0.01980250     [0.03326]
  dRAl4th          =   0.05904339    # +/-   0.02059908     [0.05586]
  dRNi1st          =  -0.00351589    # +/-   0.00286487     [-0.00126]
  dRNi2nd          =   0.02484007    # +/-   0.01085495     [0.03028]
  dRNi3rd          =   0.02741686    # +/-   0.00800848     [0.03619]
  dRNi4th          =   0.01902817    # +/-   0.00844911     [0.02709]
  thetaAl          = 631.08996866    # +/-  83.61705032     [630.17865]
  thetaNi          = 323.94544723    # +/-   5.83164044     [323.27053]
  x                =   1.58289993    # +/-   0.21988659     [1.59545]

set parameters:
  ampNi            =   0.77000000
  ampAl            =   0.66000000
  temperature      = 300.00000000
: name                     = Al-K_Al4Co48Ni48
: k-range                  = 2.8 - 10
: dk                       = 0.5
: k-window                 = Hanning
: k-weight                 = 1,2,3
: R-range                  = 1.6 - 5.2
: dR                       = 0.5
: R-window                 = Hanning
: fitting space            = r
: background function      = no
: phase correction         = no
: background removal       = E0: 1556.648074, Rbkg: 1.0, range: [0:10.729], clamps: 0/24, kw: 2
: user-supplied epsilon_k = 0
: epsilon_k by k-weight    = 9.643e-003
: epsilon_r by k-weight    = 1.282e+000
: R-factor by k-weight     = 1 -> 0.02342,  2 -> 0.01867,  3 -> 0.01761
```

name	N	S02	sigma^2	e0	delr	Reff	R
[feff_Al_abs_Co_scatt] Co1.1	12.000	0.660	0.00383	8.575	0.02298	2.50320	2.52618
[feff_Al_abs_Co_scatt] Co1.2	6.000	0.660	0.00542	8.575	-0.01063	3.54000	3.52937
[feff_Al_abs_Co_scatt] Co1.1 Co1.1	48.000	0.660	0.00441	8.575	0.03240	3.75470	3.78710
[feff_Al_abs_Co_scatt] Co1.1 Co1.1	24.000	0.660	0.00428	8.575	0.03240	4.27320	4.30560
[feff_Al_abs_Co_scatt] Co1.1 Co1.2	48.000	0.660	0.00509	8.575	0.00617	4.27320	4.27937
[feff_Al_abs_Co_scatt] Co1.3	24.000	0.660	0.00553	8.575	0.03652	4.33560	4.37212
[feff_Al_abs_Co_scatt] Co1.1 Co1.1	48.000	0.660	0.00389	8.575	0.04596	4.67100	4.71696
[feff_Al_abs_Co_scatt] Co1.1 Co1.3	96.000	0.660	0.00537	8.575	0.02975	4.67100	4.70075
[feff_Al_abs_Co_scatt] Co1.4	12.000	0.660	0.00557	8.575	0.05904	5.00630	5.06534
[feff_Al_abs_Co_scatt] Co1.1 Co1.4	24.000	0.660	0.00557	8.575	0.05904	5.00630	5.06534
[feff_Al_abs_Co_scatt] Co1.1 Co1.4 Co1.1	12.000	0.660	0.00557	8.575	0.05904	5.00630	5.06534

```
: name                     = Ni-K_Al4Co48Ni48
: k-range                  = 3.000 - 12.5
: dk                       = 0.5
: k-window                 = Hanning
: k-weight                 = 1,2,3
: R-range                  = 1.6 - 5.2
: dR                       = 0.5
: R-window                 = Hanning
: fitting space            = r
: background function      = no
: phase correction         = no
: background removal       = E0: 8331.846473, Rbkg: 1.0, range: [0:12.8], clamps: 0/24, kw: 2
: user-supplied epsilon_k = 0
: epsilon_k by k-weight    = 1.605e-003
: epsilon_r by k-weight    = 4.004e-001
: R-factor by k-weight     = 1 -> 0.00699,  2 -> 0.00628,  3 -> 0.00981
```

name	N	S02	sigma^2	e0	delr	Reff	R
[feff_Ni_abs_Al_scatt] Al1.1	1.000	1.219	0.00383	8.170	0.02298	2.50320	2.52618
[feff_Ni_abs_Co_scatt] Co1.1	1.000	8.021	0.00544	8.170	-0.00352	2.50320	2.49968

```
[feff_Ni_abs_Co_scatt] Co1.2                     6.000    1.000    0.01141    8.170   0.02484  3.54000   3.56484
[feff_Ni_abs_Co_scatt] Co1.1 Co1.1              48.000    1.000    0.01032    8.170  -0.00496  3.75470   3.74974
[feff_Ni_abs_Co_scatt] Co1.1 Co1.1              24.000    1.000    0.01146    8.170  -0.00496  4.27320   4.26824
[feff_Ni_abs_Co_scatt] Co1.1 Co1.2              48.000    1.000    0.01147    8.170   0.01066  4.27320   4.28386
[feff_Ni_abs_Co_scatt] Co1.3                    24.000    1.000    0.01178    8.170   0.02742  4.33560   4.36302
[feff_Ni_abs_Co_scatt] Co1.1 Co1.1              48.000    1.000    0.01179    8.170  -0.00703  4.67100   4.66397
[feff_Ni_abs_Co_scatt] Co1.1 Co1.3              96.000    1.000    0.01181    8.170   0.02742  4.67100   4.69842
[feff_Ni_abs_Co_scatt] Co1.4                    12.000    1.000    0.01193    8.170   0.01903  5.00630   5.02533
[feff_Ni_abs_Co_scatt] Co1.1 Co1.1              12.000    1.000    0.01191    8.170  -0.00703  5.00630   4.99927
[feff_Ni_abs_Co_scatt] Co1.1 Co1.4              24.000    1.000    0.01193    8.170   0.01903  5.00630   5.02533
[feff_Ni_abs_Co_scatt] Co1.1                    12.000    1.000    0.01191    8.170  -0.00703  5.00630   4.99927
[feff_Ni_abs_Co_scatt] Co1.1 Co1.4 Co1.1        12.000    1.000    0.01193    8.170   0.01903  5.00630   5.02533
```

$Al_4Co_{24}Cr_{24}Fe_{24}Ni_{24}$

Al-K; Cr-K

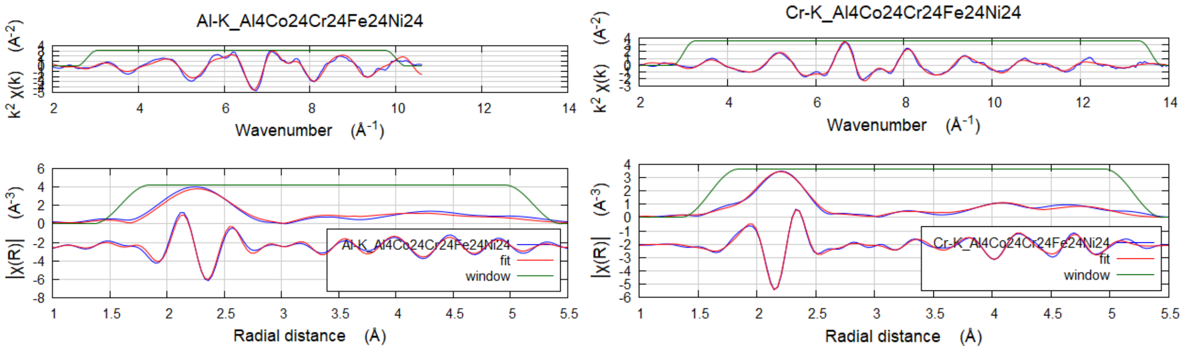


```
Independent points          : 40.1015625
Number of variables         : 15
Chi-square                  : 5801.2746868
Reduced chi-square          : 231.1120946
R-factor                    : 0.0235157
Number of data sets         : 2
```

```
guess parameters:
  dEnotAl            =   6.86542067    # +/-    0.98237118     [6.70727]
  dEnotCr            =   8.35659987    # +/-    0.94256144     [8.47203]
  dR1st              =   0.04528558    # +/-    0.00681319     [0.04454]
  ss1st              =   0.00499392    # +/-    0.00044333     [0.00506]
  ssCr1st            =   0.00644611    # +/-    0.00071532     [0.00686]
  dRAl2nd            =   0.03088184    # +/-    0.02290825     [0.03419]
  dRAl3rd            =   0.05538752    # +/-    0.01694365     [0.05227]
  dRAl4th            =   0.10117633    # +/-    0.01814892     [0.09741]
  dRCr1st            =   0.01604203    # +/-    0.00664536     [0.01697]
  dRCr2nd            =   0.03496745    # +/-    0.02806048     [0.03767]
  dRCr3rd            =   0.04924387    # +/-    0.01765187     [0.05325]
  dRCr4th            =   0.07979006    # +/-    0.01958785     [0.08285]
  thetaAl            = 500.12397720    # +/-   36.72764847     [500.90353]
  thetaCr            = 351.77753137    # +/-   17.93287070     [350.72381]
  x                  =   0.03875135    # +/-    0.54309913     [-0.15543]

set parameters:
  ampAl              =   0.66000000
  ampCr              =   0.57000000
  temperature        = 300.00000000


: name                      = Al-K_Al4Co24Cr24Fe24Ni24
```

```
: k-range                  = 2.8 - 10
: dk                       = 0.5
: k-window                 = Hanning
: k-weight                 = 1,2,3
: R-range                  = 1.6 - 5.2
: dR                       = 0.5
: R-window                 = Hanning
: fitting space            = r
: background function      = no
: phase correction         = no
: background removal       = E0: 1555.512908, Rbkg: 1.0, range: [0:10.642], clamps: 0/24, kw: 2
: user-supplied epsilon_k = 0
: epsilon_k by k-weight    = 1.428e-003
: epsilon_r by k-weight    = 1.867e-001
: R-factor by k-weight     = 1 -> 0.03839,  2 -> 0.02885,  3 -> 0.02583
```

name	N	S02	sigma^2	e0	delr	Reff	R
[feff_Al_abs_Co_scatt] Co1.1	12.000	0.660	0.00499	6.865	0.04529	2.50320	2.54849
[feff_Al_abs_Co_scatt] Co1.2	6.000	0.660	0.00820	6.865	0.03088	3.54000	3.57088
[feff_Al_abs_Co_scatt] Co1.1 Co1.1	48.000	0.660	0.00664	6.865	0.06385	3.75470	3.81855
[feff_Al_abs_Co_scatt] Co1.1 Co1.1	24.000	0.660	0.00645	6.865	0.06385	4.27320	4.33705
[feff_Al_abs_Co_scatt] Co1.1 Co1.2	48.000	0.660	0.00768	6.865	0.03808	4.27320	4.31128
[feff_Al_abs_Co_scatt] Co1.3	24.000	0.660	0.00840	6.865	0.05539	4.33560	4.39099
[feff_Al_abs_Co_scatt] Co1.1 Co1.1	48.000	0.660	0.00589	6.865	0.09057	4.67100	4.76157
[feff_Al_abs_Co_scatt] Co1.1 Co1.3	96.000	0.660	0.00815	6.865	0.05034	4.67100	4.72134
[feff_Al_abs_Co_scatt] Co1.4	12.000	0.660	0.00848	6.865	0.10118	5.00630	5.10748
[feff_Al_abs_Co_scatt] Co1.1 Co1.4	24.000	0.660	0.00848	6.865	0.10118	5.00630	5.10748
[feff_Al_abs_Co_scatt] Co1.1 Co1.4 Co1.1	12.000	0.660	0.00848	6.865	0.10118	5.00630	5.10748

```
: name                     = Cr-K_Al4Co24Cr24Fe24Ni24
: k-range                  = 3.000 - 13.6
: dk                       = 0.5
: k-window                 = Hanning
: k-weight                 = 1,2,3
: R-range                  = 1.6 - 5.2
: dR                       = 0.5
: R-window                 = Hanning
: fitting space            = r
: background function      = no
: phase correction         = no
: background removal       = E0: 5989.423294, Rbkg: 1.0, range: [0.000:14.018], clamps: 0/24, kw:
2
: user-supplied epsilon_k = 0
: epsilon_k by k-weight    = 7.512e-004
: epsilon_r by k-weight    = 2.568e-001
: R-factor by k-weight     = 1 -> 0.00710,  2 -> 0.01318,  3 -> 0.02775
```

name	N	S02	sigma^2	e0	delr	Reff	R
[feff_Cr_abs_Al_scatt] Al1.1	1.000	0.022	0.00499	8.357	0.04529	2.50320	2.54849
[feff_Cr_abs_Co_scatt] Co1.1	1.000	6.818	0.00645	8.357	0.01604	2.50320	2.51924
[feff_Cr_abs_Co_scatt] Co1.2	6.000	0.570	0.01039	8.357	0.03497	3.54000	3.57497
[feff_Cr_abs_Co_scatt] Co1.1 Co1.1	48.000	0.570	0.00924	8.357	0.02262	3.75470	3.77732
[feff_Cr_abs_Co_scatt] Co1.1 Co1.1	24.000	0.570	0.01007	8.357	0.02262	4.27320	4.29582
[feff_Cr_abs_Co_scatt] Co1.1 Co1.2	48.000	0.570	0.01034	8.357	0.02551	4.27320	4.29871
[feff_Cr_abs_Co_scatt] Co1.3	24.000	0.570	0.01071	8.357	0.04924	4.33560	4.38484
[feff_Cr_abs_Co_scatt] Co1.1 Co1.1	48.000	0.570	0.01021	8.357	0.03208	4.67100	4.70308
[feff_Cr_abs_Co_scatt] Co1.1 Co1.3	96.000	0.570	0.01069	8.357	0.03264	4.67100	4.70364
[feff_Cr_abs_Co_scatt] Co1.4	12.000	0.570	0.01085	8.357	0.07979	5.00630	5.08609
[feff_Cr_abs_Co_scatt] Co1.1 Co1.1	12.000	0.570	0.01017	8.357	0.03208	5.00630	5.03838
[feff_Cr_abs_Co_scatt] Co1.1 Co1.4	24.000	0.570	0.01085	8.357	0.07979	5.00630	5.08609
[feff_Cr_abs_Co_scatt] Co1.1	12.000	0.570	0.01017	8.357	0.03208	5.00630	5.03838
[feff_Cr_abs_Co_scatt] Co1.1 Co1.4 Co1.1	12.000	0.570	0.01085	8.357	0.07979	5.00630	5.08609

Al-K; Fe-K

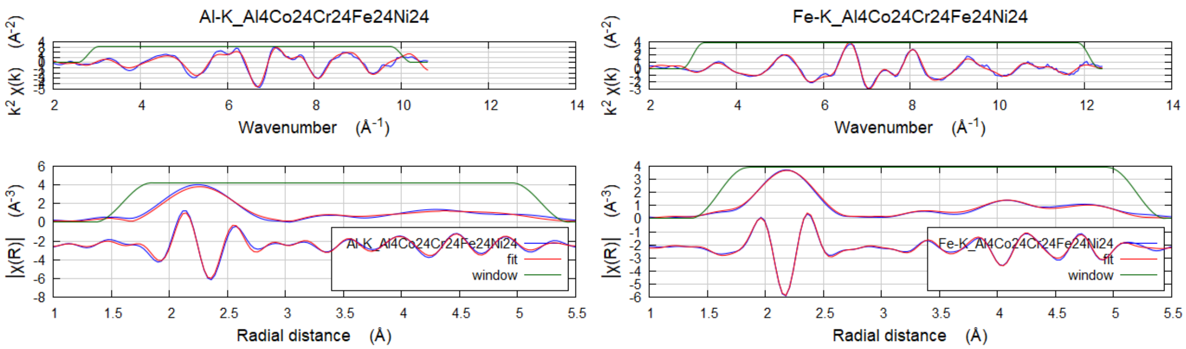


```
Independent points           : 36.7031250
Number of variables          : 15
Chi-square                   : 4346.1837956
Reduced chi-square           : 200.2561288
R-factor                     : 0.0154213
Number of data sets          : 2


guess parameters:
  dEnotFe           =   8.17136087    # +/-    0.87114696     [8.17136]
  dEnotAl           =   6.44007559    # +/-    0.93096142     [6.44004]
  dR1st             =   0.04237447    # +/-    0.00640008     [0.04237]
  ssFe1st           =   0.00760953    # +/-    0.00054672     [0.00761]
  dRAl2nd           =   0.02942313    # +/-    0.02630675     [0.02942]
  dRAl3rd           =   0.04916105    # +/-    0.01899593     [0.04916]
  dRAl4th           =   0.09064962    # +/-    0.02032512     [0.09065]
  dRFe1st           =   0.01134536    # +/-    0.00612898     [0.01135]
  dRFe2nd           =   0.04420860    # +/-    0.02242646     [0.04421]
  dRFe3rd           =   0.04963301    # +/-    0.01446747     [0.04963]
  dRFe4th           =   0.07167563    # +/-    0.01590498     [0.07168]
  x                 =   0.62727988    # +/-    0.75556605     [0.62728]
  thetaAl           = 443.00205570    # +/-   25.96171196     [443.00259]
  thetaFe           = 343.03585308    # +/-   13.63582756     [343.03593]
  ss1st             =   0.00502172    # +/-    0.00041320     [0.00502]

set parameters:
  ampFe             =   0.75000000
  ampAl             =   0.66000000
  temperature       = 300.00000000

: name                        = Al-K_Al4Co24Cr24Fe24Ni24
: k-range                     = 2.8 - 10
: dk                          = 0.5
: k-window                    = Hanning
: k-weight                    = 1,2,3
: R-range                     = 1.6 - 5.2
: dR                          = 0.5
: R-window                    = Hanning
: fitting space               = r
: background function         = no
: phase correction            = no
: background removal          = E0: 1555.512908, Rbkg: 1.0, range: [0:10.642], clamps: 0/24, kw: 2
: user-supplied epsilon_k = 0
: epsilon_k by k-weight       = 1.428e-003
: epsilon_r by k-weight       = 1.867e-001
: R-factor by k-weight        = 1 -> 0.03456,   2 -> 0.02271,   3 -> 0.01900
```

name	N	S02	sigma^2	e0	delr	Reff	R
[feff_Al_abs_Co_scatt] Co1.1	12.000	0.660	0.00502	6.440	0.04237	2.50320	2.54557
[feff_Al_abs_Co_scatt] Co1.2	6.000	0.660	0.01025	6.440	0.02942	3.54000	3.56942
[feff_Al_abs_Co_scatt] Co1.1 Co1.1	48.000	0.660	0.00828	6.440	0.05975	3.75470	3.81445
[feff_Al_abs_Co_scatt] Co1.1 Co1.1	24.000	0.660	0.00805	6.440	0.05975	4.27320	4.33295
[feff_Al_abs_Co_scatt] Co1.1 Co1.2	48.000	0.660	0.00959	6.440	0.03590	4.27320	4.30910

[feff_Al_abs_Co_scatt] Co1.3	24.000	0.660	0.01051	6.440	0.04916	4.33560	4.38476
[feff_Al_abs_Co_scatt] Co1.1 Co1.1	48.000	0.660	0.00737	6.440	0.08475	4.67100	4.75575
[feff_Al_abs_Co_scatt] Co1.1 Co1.3	96.000	0.660	0.01019	6.440	0.04577	4.67100	4.71677
[feff_Al_abs_Co_scatt] Co1.4	12.000	0.660	0.01062	6.440	0.09065	5.00630	5.09695
[feff_Al_abs_Co_scatt] Co1.1 Co1.4	24.000	0.660	0.01062	6.440	0.09065	5.00630	5.09695
[feff_Al_abs_Co_scatt] Co1.1 Co1.4 Co1.1	12.000	0.660	0.01062	6.440	0.09065	5.00630	5.09695
[feff_Al_abs_Co_scatt] Co1.1	12.000	0.660	0.00657	6.440	0.08475	5.00630	5.09105
[feff_Al_abs_Co_scatt] Co1.1 Co1.1	12.000	0.660	0.00657	6.440	0.08475	5.00630	5.09105

```
: name                     = Fe-K_Al4Co24Cr24Fe24Ni24
: k-range                  = 3.000 - 12.1
: dk                       = 0.5
: k-window                 = Hanning
: k-weight                 = 1,2,3
: R-range                  = 1.6 - 5.2
: dR                       = 0.5
: R-window                 = Hanning
: fitting space            = r
: background function      = no
: phase correction         = no
: background removal       = E0: 7112.428717, Rbkg: 1.0, range: [0:12.462], clamps: 0/24, kw: 2
: user-supplied epsilon_k = 0
: epsilon_k by k-weight    = 1.062e-003
: epsilon_r by k-weight    = 2.405e-001
: R-factor by k-weight     = 1 -> 0.00416,  2 -> 0.00475,  3 -> 0.00734
```

name	N	S02	sigma^2	e0	delr	Reff	R
[feff_Fe_abs_Al_scatt] Al1.1	1.000	0.470	0.00502	8.171	0.04237	2.50320	2.54557
[feff_Fe_abs_Co_scatt] Co1.1	1.000	9.000	0.00761	8.171	0.01134	2.50320	2.51455
[feff_Fe_abs_Co_scatt] Co1.2	6.000	0.750	0.01050	8.171	0.04421	3.54000	3.58421
[feff_Fe_abs_Co_scatt] Co1.1 Co1.1	48.000	0.750	0.00944	8.171	0.01600	3.75470	3.77070
[feff_Fe_abs_Co_scatt] Co1.1 Co1.1	24.000	0.750	0.01040	8.171	0.01600	4.27320	4.28920
[feff_Fe_abs_Co_scatt] Co1.1 Co1.2	48.000	0.750	0.01051	8.171	0.02778	4.27320	4.30098
[feff_Fe_abs_Co_scatt] Co1.3	24.000	0.750	0.01083	8.171	0.04963	4.33560	4.38523
[feff_Fe_abs_Co_scatt] Co1.1 Co1.1	48.000	0.750	0.01063	8.171	0.02269	4.67100	4.69369
[feff_Fe_abs_Co_scatt] Co1.1 Co1.3	96.000	0.750	0.01083	8.171	0.03049	4.67100	4.70149
[feff_Fe_abs_Co_scatt] Co1.4	12.000	0.750	0.01097	8.171	0.07168	5.00630	5.07798
[feff_Fe_abs_Co_scatt] Co1.1 Co1.1	12.000	0.750	0.01067	8.171	0.02269	5.00630	5.02899
[feff_Fe_abs_Co_scatt] Co1.1 Co1.4	24.000	0.750	0.01097	8.171	0.07168	5.00630	5.07798
[feff_Fe_abs_Co_scatt] Co1.1	12.000	0.750	0.01067	8.171	0.02269	5.00630	5.02899
[feff_Fe_abs_Co_scatt] Co1.1 Co1.4 Co1.1	12.000	0.750	0.01097	8.171	0.07168	5.00630	5.07798

Al-K; Co-K

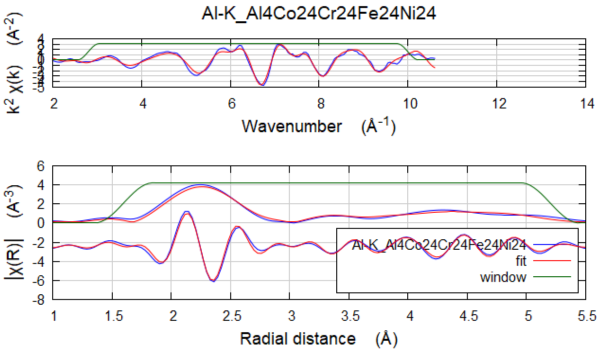

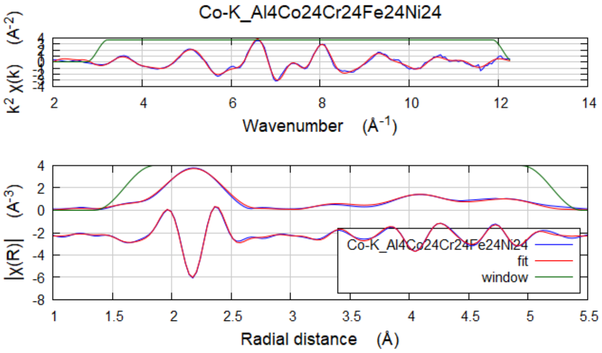


```
Independent points           : 36.7031250
Number of variables          : 15
Chi-square                   : 4176.1885884
Reduced chi-square           : 192.4233763
R-factor                     : 0.0159531
Number of data sets          : 2
```

<u>guess parameters:</u>

```
  dEnotCo          =    8.55568015    # +/-    1.02168216     [8.59204]
  dEnotAl          =    6.43719746    # +/-    0.91275610     [6.43693]
  dR1st            =    0.04235087    # +/-    0.00627513     [0.04235]
```

```
  ss1st             =   0.00502195    # +/-   0.00040504     [0.00502]
  ssCo1st           =   0.00767504    # +/-   0.00086333     [0.00768]
  dRAl2nd           =   0.02940985    # +/-   0.02578717     [0.02941]
  dRAl3rd           =   0.04912272    # +/-   0.01862173     [0.04912]
  dRAl4th           =   0.09062599    # +/-   0.01992424     [0.09063]
  dRCo1st           =   0.00756564    # +/-   0.00740650     [0.00777]
  dRCo2nd           =   0.05332495    # +/-   0.02751902     [0.05353]
  dRCo3rd           =   0.05209125    # +/-   0.01730348     [0.05241]
  dRCo4th           =   0.07308288    # +/-   0.01932862     [0.07381]
  thetaAl           = 443.00285998    # +/-  25.44856685     [442.99931]
  thetaCo           = 335.88567852    # +/-  16.36469025     [335.83875]
  x                 =   0.41010297    # +/-   0.55315011     [0.40675]

set parameters:
  ampCo             =   0.78000000
  ampAl             =   0.66000000
  temperature       = 300.00000000


: name                      = Al-K_Al4Co24Cr24Fe24Ni24
: k-range                   = 2.8 - 10
: dk                        = 0.5
: k-window                  = Hanning
: k-weight                  = 1,2,3
: R-range                   = 1.6 - 5.2
: dR                        = 0.5
: R-window                  = Hanning
: fitting space             = r
: background function       = no
: phase correction          = no
: background removal        = E0: 1555.512908, Rbkg: 1.0, range: [0:10.642], clamps: 0/24, kw: 2
: user-supplied epsilon_k = 0
: epsilon_k by k-weight     = 1.428e-003
: epsilon_r by k-weight     = 1.867e-001
: R-factor by k-weight      = 1 -> 0.03457,  2 -> 0.02271,  3 -> 0.01900
```

name	N	S02	sigma^2	e0	delr	Reff	R
[feff_Al_abs_Co_scatt] Co1.1	12.000	0.660	0.00502	6.437	0.04235	2.50320	2.54555
[feff_Al_abs_Co_scatt] Co1.2	6.000	0.660	0.01025	6.437	0.02941	3.54000	3.56941
[feff_Al_abs_Co_scatt] Co1.1 Co1.1	48.000	0.660	0.00828	6.437	0.05971	3.75470	3.81442
[feff_Al_abs_Co_scatt] Co1.1 Co1.1	24.000	0.660	0.00805	6.437	0.05971	4.27320	4.33291
[feff_Al_abs_Co_scatt] Co1.1 Co1.2	48.000	0.660	0.00959	6.437	0.03588	4.27320	4.30908
[feff_Al_abs_Co_scatt] Co1.3	24.000	0.660	0.01051	6.437	0.04912	4.33560	4.38472
[feff_Al_abs_Co_scatt] Co1.1 Co1.1	48.000	0.660	0.00737	6.437	0.08470	4.67100	4.75570
[feff_Al_abs_Co_scatt] Co1.1 Co1.3	96.000	0.660	0.01019	6.437	0.04574	4.67100	4.71674
[feff_Al_abs_Co_scatt] Co1.4	12.000	0.660	0.01062	6.437	0.09063	5.00630	5.09693
[feff_Al_abs_Co_scatt] Co1.1 Co1.1	12.000	0.660	0.00657	6.437	0.08470	5.00630	5.09100
[feff_Al_abs_Co_scatt] Co1.1 Co1.4	24.000	0.660	0.01062	6.437	0.09063	5.00630	5.09693
[feff_Al_abs_Co_scatt] Co1.1	12.000	0.660	0.00657	6.437	0.08470	5.00630	5.09100
[feff_Al_abs_Co_scatt] Co1.1 Co1.4 Co1.1	12.000	0.660	0.01062	6.437	0.09063	5.00630	5.09693

```
: name                      = Co-K_Al4Co24Cr24Fe24Ni24
: k-range                   = 3.000 - 12.1
: dk                        = 0.5
: k-window                  = Hanning
: k-weight                  = 1,2,3
: R-range                   = 1.6 - 5.2
: dR                        = 0.5
: R-window                  = Hanning
: fitting space             = r
: background function       = no
: phase correction          = no
: background removal        = E0: 7708.383341, Rbkg: 1.0, range: [0:12.3], clamps: 0/24, kw: 2
: user-supplied epsilon_k = 0
: epsilon_k by k-weight     = 1.379e-003
: epsilon_r by k-weight     = 2.992e-001
: R-factor by k-weight      = 1 -> 0.00414,  2 -> 0.00585,  3 -> 0.00947
```

name	N	S02	sigma^2	e0	delr	Reff	R
[feff_Co_abs_Al_scatt] Al1.1	1.000	0.320	0.00502	8.556	0.04235	2.50320	2.54555

[feff_Co_abs_Co_scatt]	Co1.1	1.000	9.040	0.00768	8.556	0.00757	2.50320	2.51077
[feff_Co_abs_Co_scatt]	Co1.2	6.000	0.780	0.01063	8.556	0.05332	3.54000	3.59333
[feff_Co_abs_Co_scatt]	Co1.1 Co1.1	48.000	0.780	0.00964	8.556	0.01067	3.75470	3.76537
[feff_Co_abs_Co_scatt]	Co1.1 Co1.1	24.000	0.780	0.01070	8.556	0.01067	4.27320	4.28387
[feff_Co_abs_Co_scatt]	Co1.1 Co1.2	48.000	0.780	0.01070	8.556	0.03045	4.27320	4.30365
[feff_Co_abs_Co_scatt]	Co1.3	24.000	0.780	0.01097	8.556	0.05209	4.33560	4.38769
[feff_Co_abs_Co_scatt]	Co1.1 Co1.1	48.000	0.780	0.01100	8.556	0.01513	4.67100	4.68613
[feff_Co_abs_Co_scatt]	Co1.1 Co1.3	96.000	0.780	0.01100	8.556	0.02983	4.67100	4.70083
[feff_Co_abs_Co_scatt]	Co1.4	12.000	0.780	0.01111	8.556	0.07308	5.00630	5.07938
[feff_Co_abs_Co_scatt]	Co1.1 Co1.1	12.000	0.780	0.01111	8.556	0.01513	5.00630	5.02143
[feff_Co_abs_Co_scatt]	Co1.1 Co1.4	24.000	0.780	0.01111	8.556	0.07308	5.00630	5.07938
[feff_Co_abs_Co_scatt]	Co1.1	12.000	0.780	0.01111	8.556	0.01513	5.00630	5.02143
[feff_Co_abs_Co_scatt]	Co1.1 Co1.4 Co1.1	12.000	0.780	0.01111	8.556	0.07308	5.00630	5.07938

Al-K; Ni-K

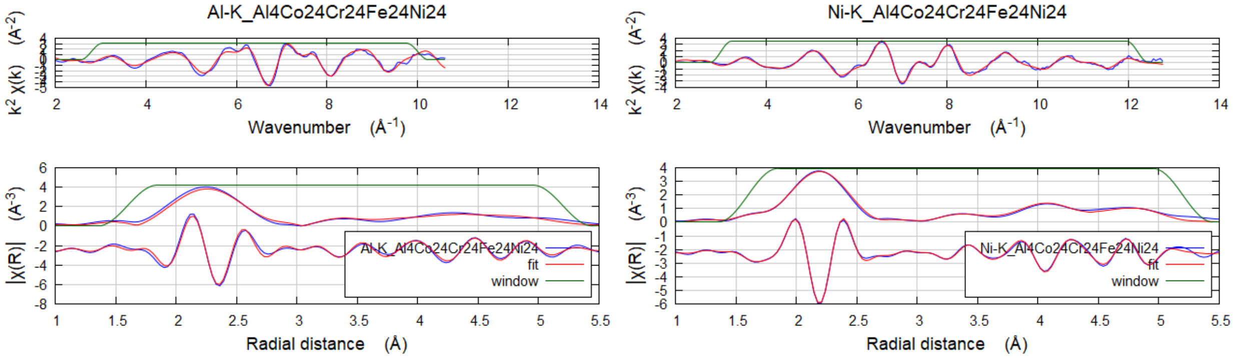


```
Independent points          : 36.9296875
Number of variables         : 15
Chi-square                  : 4347.3112013
Reduced chi-square          : 198.2386298
R-factor                    : 0.0147449
Number of data sets         : 2
```

```
guess parameters:
  dEnotNi          =   6.97223098    # +/-    0.76514947     [6.97223]
  dEnotAl          =   6.45777886    # +/-    0.89515327     [6.45789]
  dR1st            =   0.04272513    # +/-    0.00622222     [0.04273]
  ss1st            =   0.00502918    # +/-    0.00041146     [0.00503]
  ssNi1st          =   0.00702332    # +/-    0.00057431     [0.00702]
  dRAl2nd          =   0.01624995    # +/-    0.02174390     [0.01625]
  dRAl3rd          =   0.04981515    # +/-    0.01869991     [0.04982]
  dRAl4th          =   0.09045675    # +/-    0.01999900     [0.09046]
  dRNi1st          =   0.01434528    # +/-    0.00517487     [0.01435]
  dRNi2nd          =   0.05089958    # +/-    0.01906716     [0.05090]
  dRNi3rd          =   0.04501350    # +/-    0.01227174     [0.04501]
  dRNi4th          =   0.05963190    # +/-    0.01339235     [0.05963]
  thetaNi          = 342.15452554    # +/-   11.78620062     [342.15453]
  thetaAl          = 442.98967878    # +/-   25.76685934     [442.98761]
  x                =   0.67186411    # +/-    0.39939904     [0.67186]

set parameters:
  ampNi            =   0.77000000
  ampAl            =   0.66000000
  temperature      = 300.00000000
```

```
: name                    = Al-K_Al4Co24Cr24Fe24Ni24
: k-range                 = 2.8 - 10
: dk                      = 0.5
: k-window                = Hanning
: k-weight                = 1,2,3
: R-range                 = 1.6 - 5.2
```

```
: dR                        = 0.5
: R-window                  = Hanning
: fitting space             = r
: background function       = no
: phase correction          = no
: background removal        = E0: 1555.512908, Rbkg: 1.0, range: [0:10.642], clamps: 0/24, kw: 2
: user-supplied epsilon_k = 0
: epsilon_k by k-weight     = 1.428e-003
: epsilon_r by k-weight     = 1.867e-001
: R-factor by k-weight      = 1 -> 0.03161,  2 -> 0.02209,  3 -> 0.01907

  name                                              N       S02     sigma^2   e0     delr     Reff      R
==============================================================================================================
[feff_Al_abs_Co_scatt] Co1.1                    12.000   0.660   0.00503   6.458  0.04272  2.50320  2.54593
[feff_Al_abs_Co_scatt] Co1.2                     6.000   0.660   0.01025   6.458  0.01625  3.54000  3.55625
[feff_Al_abs_Co_scatt] Co1.1 Co1.1              48.000   0.660   0.00828   6.458  0.02291  3.75470  3.77761
[feff_Al_abs_Co_scatt] Co1.1 Co1.1              24.000   0.660   0.00805   6.458  0.02291  4.27320  4.29611
[feff_Al_abs_Co_scatt] Co1.1 Co1.2              48.000   0.660   0.00959   6.458  0.02949  4.27320  4.30269
[feff_Al_abs_Co_scatt] Co1.3                    24.000   0.660   0.01051   6.458  0.04981  4.33560  4.38542
[feff_Al_abs_Co_scatt] Co1.1 Co1.1              48.000   0.660   0.00737   6.458  0.08545  4.67100  4.75645
[feff_Al_abs_Co_scatt] Co1.1 Co1.3              96.000   0.660   0.01019   6.458  0.04627  4.67100  4.71727
[feff_Al_abs_Co_scatt] Co1.4                    12.000   0.660   0.01063   6.458  0.09046  5.00630  5.09676
[feff_Al_abs_Co_scatt] Co1.1 Co1.1              12.000   0.660   0.00657   6.458  0.08545  5.00630  5.09175
[feff_Al_abs_Co_scatt] Co1.1 Co1.4              24.000   0.660   0.01063   6.458  0.09046  5.00630  5.09676
[feff_Al_abs_Co_scatt] Co1.1                    12.000   0.660   0.00657   6.458  0.08545  5.00630  5.09175
[feff_Al_abs_Co_scatt] Co1.1 Co1.4 Co1.1        12.000   0.660   0.01063   6.458  0.09046  5.00630  5.09676


: name                      = Ni-K_Al4Co24Cr24Fe24Ni24
: k-range                   = 3.000 - 12.2
: dk                        = 0.5
: k-window                  = Hanning
: k-weight                  = 1,2,3
: R-range                   = 1.6 - 5.2
: dR                        = 0.5
: R-window                  = Hanning
: fitting space             = r
: background function       = no
: phase correction          = no
: background removal        = E0: 8331.977408, Rbkg: 1.0, range: [0:12.816], clamps: 0/24, kw: 2
: user-supplied epsilon_k = 0
: epsilon_k by k-weight     = 9.436e-004
: epsilon_r by k-weight     = 2.355e-001
: R-factor by k-weight      = 1 -> 0.00481,  2 -> 0.00440,  3 -> 0.00650

  name                                              N       S02     sigma^2   e0     delr     Reff      R
==============================================================================================================
[feff_Ni_abs_Al_scatt] Al1.1                     1.000   0.517   0.00503   6.972  0.04272  2.50320  2.54593
[feff_Ni_abs_Co_scatt] Co1.1                     1.000   8.723   0.00702   6.972  0.01435  2.50320  2.51755
[feff_Ni_abs_Co_scatt] Co1.2                     6.000   0.770   0.01028   6.972  0.05090  3.54000  3.59090
[feff_Ni_abs_Co_scatt] Co1.1 Co1.1              48.000   0.770   0.00930   6.972  0.02023  3.75470  3.77493
[feff_Ni_abs_Co_scatt] Co1.1 Co1.1              24.000   0.770   0.01033   6.972  0.02023  4.27320  4.29343
[feff_Ni_abs_Co_scatt] Co1.1 Co1.2              48.000   0.770   0.01034   6.972  0.03262  4.27320  4.30582
[feff_Ni_abs_Co_scatt] Co1.3                    24.000   0.770   0.01061   6.972  0.04501  4.33560  4.38061
[feff_Ni_abs_Co_scatt] Co1.1 Co1.1              48.000   0.770   0.01062   6.972  0.02869  4.67100  4.69969
[feff_Ni_abs_Co_scatt] Co1.1 Co1.3              96.000   0.770   0.01064   6.972  0.02968  4.67100  4.70068
[feff_Ni_abs_Co_scatt] Co1.4                    12.000   0.770   0.01074   6.972  0.05963  5.00630  5.06593
[feff_Ni_abs_Co_scatt] Co1.1 Co1.1              12.000   0.770   0.01072   6.972  0.02869  5.00630  5.03499
[feff_Ni_abs_Co_scatt] Co1.1 Co1.4              24.000   0.770   0.01074   6.972  0.05963  5.00630  5.06593
[feff_Ni_abs_Co_scatt] Co1.1                    12.000   0.770   0.01072   6.972  0.02869  5.00630  5.03499
[feff_Ni_abs_Co_scatt] Co1.1 Co1.4 Co1.1        12.000   0.770   0.01074   6.972  0.05963  5.00630  5.06593
```

CoCrNi

Cr-K

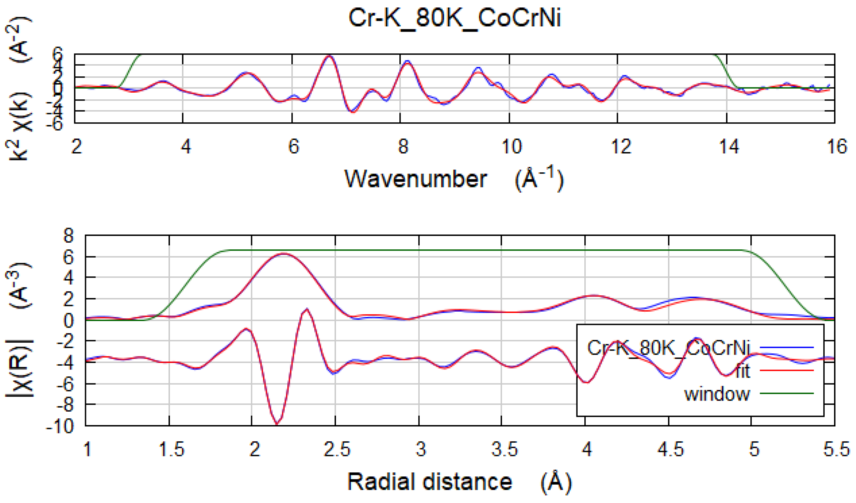


```
Independent points           : 24.8085937
Number of variables          : 8
Chi-square                   : 2968.5821656
Reduced chi-square           : 176.6109771
R-factor                     : 0.0086776
Number of data sets          : 1


guess parameters:
  ampCr              =    0.68933069    # +/-    0.03142816     [0.69000]
  dEnot              =    6.82855736    # +/-    0.45669408     [6.81093]
  dR1st              =    0.00051121    # +/-    0.00281229     [0.00042]
  dR2nd              =    0.00073522    # +/-    0.01124419     [0.00066]
  dR3rd              =    0.01919663    # +/-    0.00678466     [0.01902]
  dR4th              =    0.03284262    # +/-    0.00715805     [0.03270]
  thetaCr            =  460.62524754    # +/-   18.27501730     [460.39176]
  ss1st              =    0.00362833    # +/-    0.00034043     [0.00363]

set parameters:
  temperature        =  300.00000000


: name                     = Cr-K_80K_CoCrNi
: k-range                  = 3.000 - 14
: dk                       = 0.5
: k-window                 = Hanning
: k-weight                 = 1,2,3
: R-range                  = 1.6 - 5.2
: dR                       = 0.5
: R-window                 = Hanning
: fitting space            = r
: background function      = no
: phase correction         = no
: background removal       = E0: 5992.4982, Rbkg: 1.0, range: [0:15.974], clamps: 0/24, kw: 2
: user-supplied epsilon_k = 0
: epsilon_k by k-weight    = 8.552e-004
: epsilon_r by k-weight    = 4.622e-001
: R-factor by k-weight     = 1 -> 0.00698,  2 -> 0.00797,  3 -> 0.01108

  name                                          N       S02     sigma^2   e0      delr      Reff      R
```

==

[feff_Cr_abs_Co_scatt]	Co1.1	12.000	0.689	0.00363	6.829	0.00051	2.50320	2.50371
[feff_Cr_abs_Co_scatt]	Co1.2	6.000	0.689	0.00627	6.829	0.00073	3.54000	3.54074
[feff_Cr_abs_Co_scatt]	Co1.1 Co1.1	48.000	0.689	0.00560	6.829	0.00072	3.75470	3.75542
[feff_Cr_abs_Co_scatt]	Co1.1 Co1.1	24.000	0.689	0.00609	6.829	0.00072	4.27320	4.27392
[feff_Cr_abs_Co_scatt]	Co1.1 Co1.2	48.000	0.689	0.00625	6.829	0.00062	4.27320	4.27382
[feff_Cr_abs_Co_scatt]	Co1.3	24.000	0.689	0.00645	6.829	0.01920	4.33560	4.35480
[feff_Cr_abs_Co_scatt]	Co1.1 Co1.1	48.000	0.689	0.00615	6.829	0.00102	4.67100	4.67202
[feff_Cr_abs_Co_scatt]	Co1.1 Co1.3	96.000	0.689	0.00644	6.829	0.00985	4.67100	4.68085
[feff_Cr_abs_Co_scatt]	Co1.4	12.000	0.689	0.00652	6.829	0.03284	5.00630	5.03914
[feff_Cr_abs_Co_scatt]	Co1.1 Co1.1	12.000	0.689	0.00611	6.829	0.00102	5.00630	5.00732
[feff_Cr_abs_Co_scatt]	Co1.1 Co1.4	24.000	0.689	0.00652	6.829	0.03284	5.00630	5.03914
[feff_Cr_abs_Co_scatt]	Co1.1	12.000	0.689	0.00611	6.829	0.00102	5.00630	5.00732
[feff_Cr_abs_Co_scatt]	Co1.1 Co1.4 Co1.1	12.000	0.689	0.00652	6.829	0.03284	5.00630	5.03914

Co-K

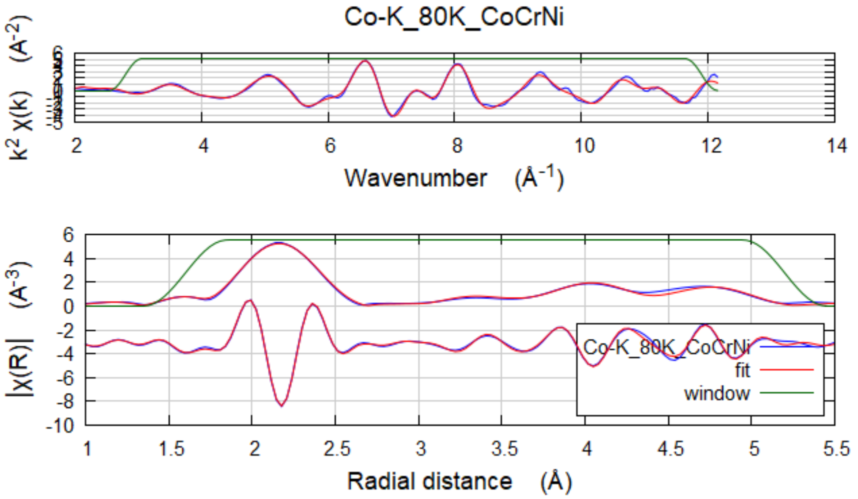


```
Independent points          : 20.5039063
Number of variables         : 8
Chi-square                  : 3039.1824283
Reduced chi-square          : 243.0586384
R-factor                    : 0.0067012
Number of data sets         : 1
```

guess parameters:

```
  ampCo              =    0.69258359    # +/-    0.03597058     [0.68537]
  dEnot              =    6.28647352    # +/-    0.47845986     [6.25052]
  dR1st              =   -0.00273949    # +/-    0.00305772     [-0.00317]
  dR2nd              =    0.02603389    # +/-    0.01179584     [0.02449]
  dR3rd              =    0.03040896    # +/-    0.00710616     [0.02903]
  dR4th              =    0.04231748    # +/-    0.00764394     [0.04129]
  thetaCo            =  424.06635577    # +/-   17.69350449     [429.17634]
  ss1st              =    0.00434989    # +/-    0.00042740     [0.00423]
```

set parameters:

```
  temperature        = 300.00000000
```

```
: name                    = Co-K_80K_CoCrNi
: k-range                 = 2.8 - 11.9
: dk                      = 0.5
: k-window                = Hanning
: k-weight                = 1,2,3
: R-range                 = 1.6 - 5.2
: dR                      = 0.5
: R-window                = Hanning
: fitting space           = r
```

```
: background function      = no
: phase correction         = no
: background removal       = E0: 7708.5019, Rbkg: 1.0, range: [0:12.209], clamps: 0/24, kw: 2
: user-supplied epsilon_k = 0
: epsilon_k by k-weight    = 1.019e-003
: epsilon_r by k-weight    = 2.148e-001
: R-factor by k-weight     = 1 -> 0.00503,  2 -> 0.00597,  3 -> 0.00910
```

name	N	S02	sigma^2	e0	delr	Reff	R
[feff_Co_abs_Co_scatt] Co1.1	12.000	0.693	0.00435	6.286	-0.00274	2.50320	2.50046
[feff_Co_abs_Co_scatt] Co1.2	6.000	0.693	0.00685	6.286	0.02603	3.54000	3.56603
[feff_Co_abs_Co_scatt] Co1.1 Co1.1	48.000	0.693	0.00623	6.286	-0.00386	3.75470	3.75084
[feff_Co_abs_Co_scatt] Co1.1 Co1.1	24.000	0.693	0.00690	6.286	-0.00386	4.27320	4.26934
[feff_Co_abs_Co_scatt] Co1.1 Co1.2	48.000	0.693	0.00690	6.286	0.01165	4.27320	4.28485
[feff_Co_abs_Co_scatt] Co1.3	24.000	0.693	0.00705	6.286	0.03041	4.33560	4.36601
[feff_Co_abs_Co_scatt] Co1.1 Co1.1	48.000	0.693	0.00708	6.286	-0.00548	4.67100	4.66552
[feff_Co_abs_Co_scatt] Co1.1 Co1.3	96.000	0.693	0.00708	6.286	0.01384	4.67100	4.68484
[feff_Co_abs_Co_scatt] Co1.4	12.000	0.693	0.00713	6.286	0.04232	5.00630	5.04862
[feff_Co_abs_Co_scatt] Co1.1 Co1.1	12.000	0.693	0.00713	6.286	-0.00548	5.00630	5.00082
[feff_Co_abs_Co_scatt] Co1.1 Co1.4	24.000	0.693	0.00713	6.286	0.04232	5.00630	5.04862
[feff_Co_abs_Co_scatt] Co1.1	12.000	0.693	0.00713	6.286	-0.00548	5.00630	5.00082
[feff_Co_abs_Co_scatt] Co1.1 Co1.4 Co1.1	12.000	0.693	0.00713	6.286	0.04232	5.00630	5.04862

Ni-K

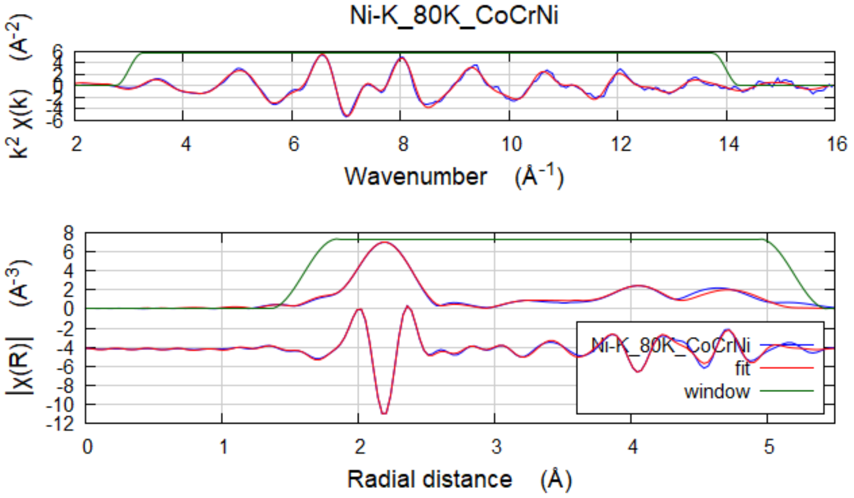


```
Independent points          : 24.8085937
Number of variables         : 8
Chi-square                  : 1187.7748937
Reduced chi-square          : 70.6647392
R-factor                    : 0.0098835
Number of data sets         : 1
```

<u>guess parameters:</u>

```
  ampNi             =   0.81035074    # +/-   0.03818342     [0.80786]
  dEnot             =   5.85048713    # +/-   0.46535095     [5.83799]
  dR1st             =   0.00372744    # +/-   0.00285570     [0.00364]
  dR2nd             =   0.02313171    # +/-   0.01185587     [0.02304]
  dR3rd             =   0.02730327    # +/-   0.00701436     [0.02718]
  dR4th             =   0.03489270    # +/-   0.00747622     [0.03451]
  thetaNi           = 434.34487998    # +/-  17.22720283     [435.31934]
  ss1st             =   0.00377614    # +/-   0.00035282     [0.00376]
```

<u>set parameters:</u>

```
  temperature       = 300.00000000
```

```
: name                      = Ni-K_80K_CoCrNi
: k-range                   = 3.000 - 14
: dk                        = 0.5
: k-window                  = Hanning
: k-weight                  = 1,2,3
: R-range                   = 1.6 - 5.2
: dR                        = 0.5
: R-window                  = Hanning
: fitting space             = r
: background function       = no
: phase correction          = no
: background removal        = E0: 8329.9992, Rbkg: 1.0, range: [0:16.028], clamps: 0/24, kw: 2
: user-supplied epsilon_k = 0
: epsilon_k by k-weight     = 1.333e-003
: epsilon_r by k-weight     = 7.281e-001
: R-factor by k-weight      = 1 -> 0.00642,  2 -> 0.00874,  3 -> 0.01449
```

name	N	S02	sigma^2	e0	delr	Reff	R
[feff_Ni_abs_Co_scatt] Co1.1	12.000	0.810	0.00378	5.850	0.00373	2.50320	2.50693
[feff_Ni_abs_Co_scatt] Co1.2	6.000	0.810	0.00656	5.850	0.02313	3.54000	3.56313
[feff_Ni_abs_Co_scatt] Co1.1 Co1.1	48.000	0.810	0.00596	5.850	0.00526	3.75470	3.75996
[feff_Ni_abs_Co_scatt] Co1.1 Co1.1	24.000	0.810	0.00660	5.850	0.00526	4.27320	4.27846
[feff_Ni_abs_Co_scatt] Co1.1 Co1.2	48.000	0.810	0.00661	5.850	0.01343	4.27320	4.28663
[feff_Ni_abs_Co_scatt] Co1.3	24.000	0.810	0.00675	5.850	0.02730	4.33560	4.36290
[feff_Ni_abs_Co_scatt] Co1.1 Co1.1	48.000	0.810	0.00677	5.850	0.00745	4.67100	4.67846
[feff_Ni_abs_Co_scatt] Co1.1 Co1.3	96.000	0.810	0.00678	5.850	0.01551	4.67100	4.68652
[feff_Ni_abs_Co_scatt] Co1.4	12.000	0.810	0.00683	5.850	0.03489	5.00630	5.04119
[feff_Ni_abs_Co_scatt] Co1.1 Co1.1	12.000	0.810	0.00681	5.850	0.00745	5.00630	5.01376
[feff_Ni_abs_Co_scatt] Co1.1 Co1.4	24.000	0.810	0.00683	5.850	0.03489	5.00630	5.04119
[feff_Ni_abs_Co_scatt] Co1.1	12.000	0.810	0.00681	5.850	0.00745	5.00630	5.01376
[feff_Ni_abs_Co_scatt] Co1.1 Co1.4 Co1.1	12.000	0.810	0.00683	5.850	0.03489	5.00630	5.04119

CoFeNi

Fe-K

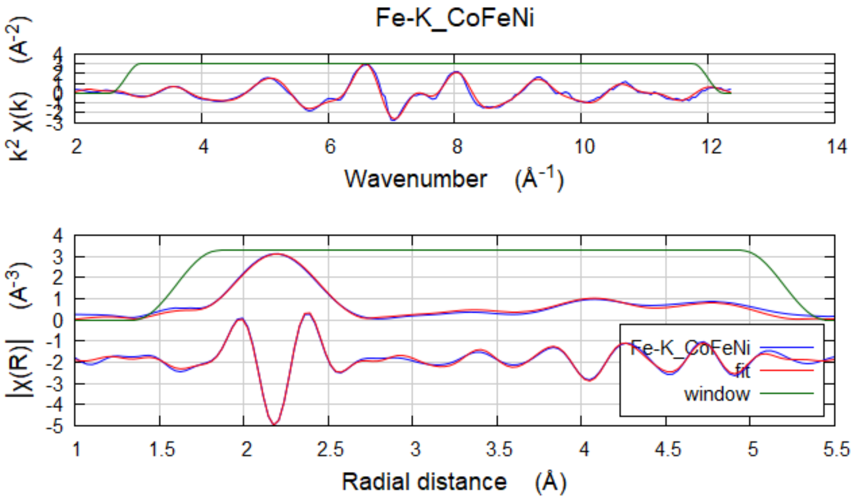


```
Independent points          : 20.7304688
Number of variables         : 8
Chi-square                  : 853.3625993
Reduced chi-square          : 67.0330854
R-factor                    : 0.0080112
Number of data sets         : 1
```

```
guess parameters:
  ampFe              =   0.51629723    # +/-    0.03088602      [0.52544]
  dEnot              =   7.93970858    # +/-    0.53188942      [7.86399]
  dR1st              =   0.02263359    # +/-    0.00353858      [0.02097]
  ss1st              =   0.00606168    # +/-    0.00050642      [0.00631]
  dR2nd              =   0.02038556    # +/-    0.01496408      [0.00766]
  dR3rd              =   0.04781665    # +/-    0.00946485      [0.04496]
  dR4th              =   0.06271705    # +/-    0.01011189      [0.06102]
  thetaFe            = 353.67465229    # +/-   13.30596645      [344.64998]

set parameters:
  temperature        = 300.00000000
```

```
: name                     = Fe-K_CoFeNi
: k-range                  = 2.8 - 12
: dk                       = 0.5
: k-window                 = Hanning
: k-weight                 = 1,2,3
: R-range                  = 1.6 - 5.2
: dR                       = 0.5
: R-window                 = Hanning
: fitting space            = r
: background function      = no
: phase correction         = no
: background removal       = E0: 7112.8068, Rbkg: 1.0, range: [0:12.396], clamps: 0/24, kw: 2
: user-supplied epsilon_k = 0
: epsilon_k by k-weight    = 7.514e-004
: epsilon_r by k-weight    = 1.677e-001
: R-factor by k-weight     = 1 -> 0.00762,  2 -> 0.00769,  3 -> 0.00873
```

name	N	S02	sigma^2	e0	delr	Reff	R
[feff_Fe_abs_Co_scatt] Co1.1	12.000	0.516	0.00606	7.940	0.02263	2.50320	2.52583

[feff_Fe_abs_Co_scatt]	Co1.2	6.000	0.516	0.00990	7.940	0.02039	3.54000	3.56039
[feff_Fe_abs_Co_scatt]	Co1.1 Co1.1	48.000	0.516	0.00891	7.940	0.03191	3.75470	3.78661
[feff_Fe_abs_Co_scatt]	Co1.1 Co1.1	24.000	0.516	0.00981	7.940	0.03191	4.27320	4.30511
[feff_Fe_abs_Co_scatt]	Co1.1 Co1.2	48.000	0.516	0.00992	7.940	0.02151	4.27320	4.29471
[feff_Fe_abs_Co_scatt]	Co1.3	24.000	0.516	0.01021	7.940	0.04782	4.33560	4.38342
[feff_Fe_abs_Co_scatt]	Co1.1 Co1.1	48.000	0.516	0.01002	7.940	0.04527	4.67100	4.71627
[feff_Fe_abs_Co_scatt]	Co1.1 Co1.3	96.000	0.516	0.01022	7.940	0.03522	4.67100	4.70622
[feff_Fe_abs_Co_scatt]	Co1.4	12.000	0.516	0.01034	7.940	0.06272	5.00630	5.06902
[feff_Fe_abs_Co_scatt]	Co1.1 Co1.1	12.000	0.516	0.01006	7.940	0.04527	5.00630	5.05157
[feff_Fe_abs_Co_scatt]	Co1.1 Co1.4	24.000	0.516	0.01034	7.940	0.06272	5.00630	5.06902
[feff_Fe_abs_Co_scatt]	Co1.1	12.000	0.516	0.01006	7.940	0.04527	5.00630	5.05157
[feff_Fe_abs_Co_scatt]	Co1.1 Co1.4 Co1.1	12.000	0.516	0.01034	7.940	0.06272	5.00630	5.06902

Fe-K

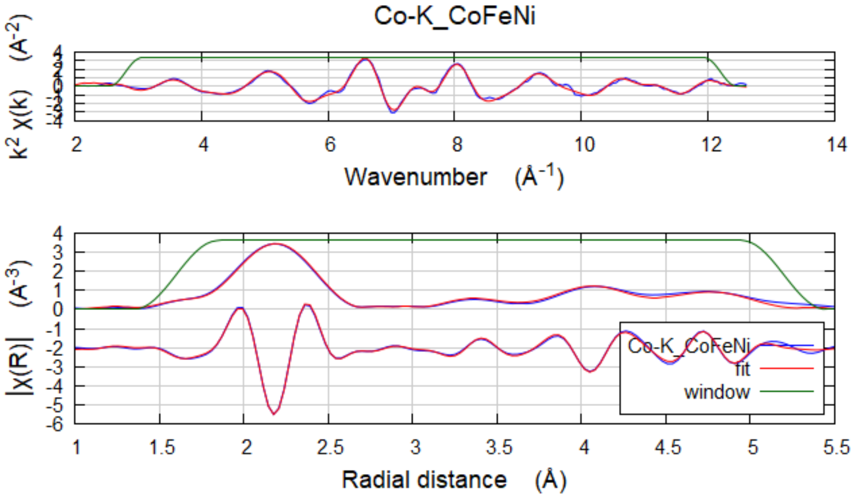


```
Independent points          : 21.1835938
Number of variables         : 8
Chi-square                  : 821.6806626
Reduced chi-square          : 62.3259999
R-factor                    : 0.0041935
Number of data sets         : 1


guess parameters:
  ampCo              =   0.58603989    # +/-   0.02445726     [0.58999]
  dEnot              =   8.04961401    # +/-   0.36795037     [7.97204]
  dR1st              =   0.01018864    # +/-   0.00254673     [0.00975]
  ss1st              =   0.00648304    # +/-   0.00036372     [0.00652]
  dR2nd              =   0.03651350    # +/-   0.01054435     [0.03605]
  dR3rd              =   0.05001801    # +/-   0.00645051     [0.04952]
  dR4th              =   0.06071131    # +/-   0.00699122     [0.05994]
  thetaCo            = 352.72244638    # +/-   9.21249086     [351.79289]

set parameters:
  temperature        = 300.00000000


: name                    = Co-K_CoFeNi
: k-range                 = 2.8 - 12.2
: dk                      = 0.5
: k-window                = Hanning
: k-weight                = 1,2,3
: R-range                 = 1.6 - 5.2
: dR                      = 0.5
: R-window                = Hanning
: fitting space           = r
: background function     = no
: phase correction        = no
```

```
: background removal       = E0: 7709.072719, Rbkg: 1.0, range: [0:13], clamps: 0/24, kw: 2
: user-supplied epsilon_k = 0
: epsilon_k by k-weight    = 6.188e-004
: epsilon_r by k-weight    = 1.482e-001
: R-factor by k-weight     = 1 -> 0.00478,  2 -> 0.00368,  3 -> 0.00413
```

name	N	S02	sigma^2	e0	delr	Reff	R
[feff_Co_abs_Co_scatt] Co1.1	12.000	0.586	0.00648	8.050	0.01019	2.50320	2.51339
[feff_Co_abs_Co_scatt] Co1.2	6.000	0.586	0.00968	8.050	0.03651	3.54000	3.57651
[feff_Co_abs_Co_scatt] Co1.1 Co1.1	48.000	0.586	0.00878	8.050	0.01437	3.75470	3.76907
[feff_Co_abs_Co_scatt] Co1.1 Co1.1	24.000	0.586	0.00975	8.050	0.01437	4.27320	4.28757
[feff_Co_abs_Co_scatt] Co1.1 Co1.2	48.000	0.586	0.00975	8.050	0.02335	4.27320	4.29655
[feff_Co_abs_Co_scatt] Co1.3	24.000	0.586	0.00999	8.050	0.05002	4.33560	4.38562
[feff_Co_abs_Co_scatt] Co1.1 Co1.1	48.000	0.586	0.01002	8.050	0.02038	4.67100	4.69138
[feff_Co_abs_Co_scatt] Co1.1 Co1.3	96.000	0.586	0.01002	8.050	0.03010	4.67100	4.70110
[feff_Co_abs_Co_scatt] Co1.4	12.000	0.586	0.01011	8.050	0.06071	5.00630	5.06701
[feff_Co_abs_Co_scatt] Co1.1 Co1.1	12.000	0.586	0.01011	8.050	0.02038	5.00630	5.02668
[feff_Co_abs_Co_scatt] Co1.1 Co1.4	24.000	0.586	0.01011	8.050	0.06071	5.00630	5.06701
[feff_Co_abs_Co_scatt] Co1.1	12.000	0.586	0.01011	8.050	0.02038	5.00630	5.02668
[feff_Co_abs_Co_scatt] Co1.1 Co1.4 Co1.1	12.000	0.586	0.01011	8.050	0.06071	5.00630	5.06701

Ni-K

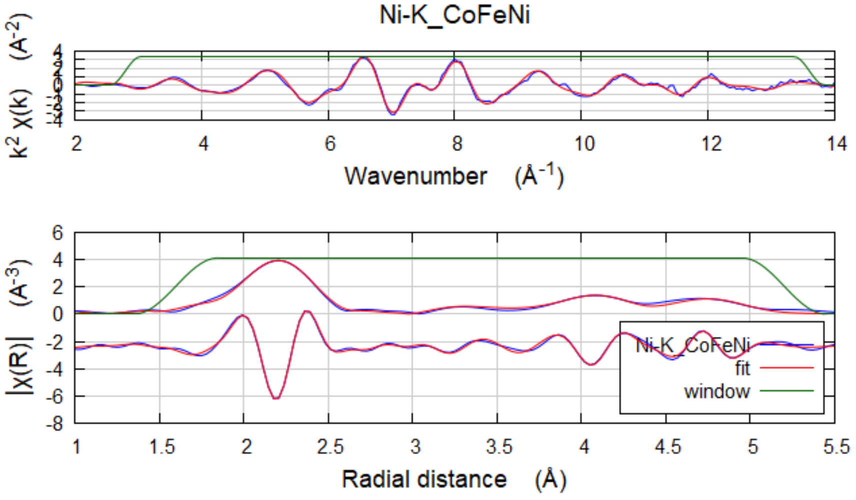


```
Independent points          : 24.3554688
Number of variables         : 8
Chi-square                  : 1010.1712350
Reduced chi-square          : 61.7635147
R-factor                    : 0.0125342
Number of data sets         : 1
```

```
guess parameters:
  amp              =    0.58681959    # +/-    0.03252766     [0.59244]
  dEnot            =    7.24582952    # +/-    0.49404380     [7.13534]
  dR1st            =    0.01178836    # +/-    0.00345471     [0.01109]
  ss1st            =    0.00557001    # +/-    0.00046878     [0.00564]
  dR2nd            =    0.03869288    # +/-    0.01394783     [0.03837]
  dR3rd            =    0.07388831    # +/-    0.01653738     [0.07346]
  dR4th            =    0.04921176    # +/-    0.00904003     [0.04800]
  thetaNi          =  384.03525980    # +/-   15.23785884     [382.58788]

set parameters:
  temperature      =  300.00000000
```

```
: name                     = Ni-K_CoFeNi
: k-range                  = 2.8 - 13.6
: dk                       = 0.5
```

```
: k-window                 = Hanning
: k-weight                 = 1,2,3
: R-range                  = 1.6 - 5.2
: dR                       = 0.5
: R-window                 = Hanning
: fitting space            = r
: background function      = no
: phase correction         = no
: background removal       = E0: 8331.2242, Rbkg: 1.0, range: [0:14.966], clamps: 0/24, kw: 2
: user-supplied epsilon_k = 0
: epsilon_k by k-weight    = 8.577e-004
: epsilon_r by k-weight    = 3.736e-001
: R-factor by k-weight     = 1 -> 0.00829,  2 -> 0.01073,  3 -> 0.01858
```

name	N	S02	sigma^2	e0	delr	Reff	R
[feff_Ni_abs_Co_scatt] Co1.1	12.000	0.587	0.00557	7.246	0.01179	2.50320	2.51499
[feff_Ni_abs_Co_scatt] Co1.2	6.000	0.587	0.00826	7.246	0.03869	3.54000	3.57869
[feff_Ni_abs_Co_scatt] Co1.1 Co1.1	48.000	0.587	0.00749	7.246	0.01662	3.75470	3.77132
[feff_Ni_abs_Co_scatt] Co1.1 Co1.1	24.000	0.587	0.00830	7.246	0.01662	4.27320	4.28982
[feff_Ni_abs_Co_scatt] Co1.1 Co1.2	48.000	0.587	0.00831	7.246	0.02524	4.27320	4.29844
[feff_Ni_abs_Co_scatt] Co1.3	24.000	0.587	0.00851	7.246	0.04284	4.33560	4.37844
[feff_Ni_abs_Co_scatt] Co1.1 Co1.1	48.000	0.587	0.00853	7.246	0.02358	4.67100	4.69458
[feff_Ni_abs_Co_scatt] Co1.1 Co1.3	96.000	0.587	0.00854	7.246	0.04284	4.67100	4.71384
[feff_Ni_abs_Co_scatt] Co1.4	12.000	0.587	0.00861	7.246	0.04921	5.00630	5.05551
[feff_Ni_abs_Co_scatt] Co1.1 Co1.1	12.000	0.587	0.00860	7.246	0.02358	5.00630	5.02988
[feff_Ni_abs_Co_scatt] Co1.1 Co1.4	24.000	0.587	0.00861	7.246	0.04921	5.00630	5.05551
[feff_Ni_abs_Co_scatt] Co1.1	12.000	0.587	0.00860	7.246	0.02358	5.00630	5.02988
[feff_Ni_abs_Co_scatt] Co1.1 Co1.4 Co1.1	12.000	0.587	0.00861	7.246	0.04921	5.00630	5.05551

CoCrFeNi

Data are contained in **Anomalous behavior of thermophysical properties of CrFeNi, CoCrNi, and CoCrFeNi medium-and high-entropy alloys,** October 2023, B-MRS conference (DOI: 10.13140/RG.2.2.30552.47361) and in the Supporting Information of: The impact of chemical short-range order on the thermophysical properties of medium- and high-entropy alloys, A. F. Andreoli *et al. unpublished)*
