# Supplementary material for: How Atomic Bonding Plays the Hardness Behavior in the Al–Co–Cr–Cu–Fe–Ni High Entropy Family
Source: Small Sci. 2023 Dec 7;4(2):2300225. doi: 10.1002/smsc.202300225 (PMC11935079; doi:10.1002/smsc.202300225)
Supplement: Supplementary file 2 — Supplementary Material [file SMSC-4-2300225-s001.pdf]

How Atomic Bonding Plays the Hardness Behavior in the Al–Co–Cr–Cu–Fe–Ni High Entropy Family

A. Fantin[1,2*], G. O. Lepore[3], M. Widom[4], S. Kasatikov[2], A. M. Manzoni[1]

[1]Federal Institute of Materials Research and Testing (BAM), 12205 Berlin, Germany

[2]Helmholtz-Zentrum Berlin, 14109 Berlin, Germany

[3]Università degli Studi di Firenze, 50121 Firenze, Italy

[4]Carnegie Mellon University, Pittsburgh PA, 15217, U.S.A.

*corresponding author: andrea.fantin@bam.de

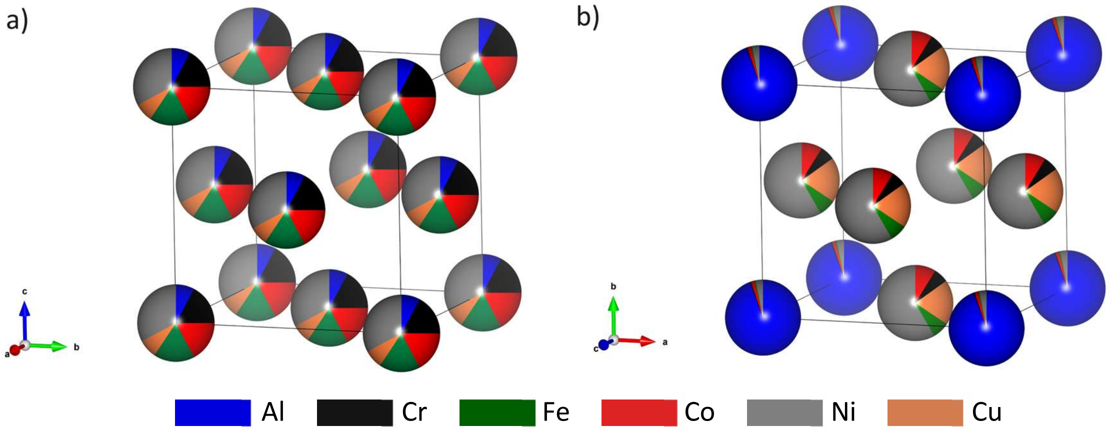

Figure S1. Schematic representation of a) A1/γ (disordered, $Fm\bar{3}m$) and b) $L1_2$/γ' (ordered, $Pm\bar{3}m$) crystal structures, with elemental ratios as in Daoud, Manzoni [1] and partial occupancy based on stoichiometry.

Table S1. Chemical composition EDS results measured on at least 20-point analysis in different grains on all specimens.

**Sample name/ atoms %**		**CCA**	**$CCA_{sans}Al$**	**$CCA_{sans}Cr$**	**$CCA_{sans}Fe$**	**$CCA_{sans}Co$**	**$CCA_{sans}Cu$**
**Al**	**Max**<br>**Min**	7.53<br>6.86		9.54<br>8.84	9.46<br>8.64	9.54<br>8.84	7.58<br>6.63
	**Average**<br>**Std. Dev.**	7.20<br>0.15		9.20<br>0.19	9.04<br>0.20	9.20<br>0.18	7.17<br>0.24
**Cr**	**Max**<br>**Min**	16.63<br>15.64	18.53<br>17.7		20.13<br>19.47	20.13<br>19.48	18.79<br>17.91
	**Average**<br>**Std. Dev.**	16.19<br>0.21	18.12<br>0.22		19.82<br>0.21	19.84<br>0.21	18.27<br>0.22
**Fe**	**Max**<br>**Min**	17.78<br>16.31	19.66<br>18.64	20.26<br>19.11		20.55<br>19.53	19.12<br>18.04
	**Average**<br>**Std. Dev.**	16.98<br>0.30	19.06<br>0.36	19.72<br>0.28		19.98<br>0.24	18.69<br>0.26
**Co**	**Max**<br>**Min**	17.08<br>15.80	20.39<br>19.27	20.70<br>19.27	21.02<br>19.61		20.75<br>19.46
	**Average**<br>**Std. Dev.**	16.42<br>0.31	19.82<br>0.31	19.99<br>0.30	20.42<br>0.32		20.07<br>0.33
**Ni**	**Max**<br>**Min**	35.36<br>33.61	35.60<br>34.36	38.74<br>37.64	39.40<br>38.33	39.33<br>38.09	36.31<br>34.93
	**Average**<br>**Std. Dev.**	34.75<br>0.45	35.04<br>0.33	38.15<br>0.31	38.91<br>0.27	38.80<br>0.32	35.81<br>0.37
**Cu**	**Max**<br>**Min**	9.17<br>7.8	8.46<br>7.53	13.81<br>12.25	12.23<br>11.36	12.57<br>11.66	
	**Average**<br>**Std. Dev.**	8.46<br>0.31	7.96<br>0.25	12.94<br>0.38	11.82<br>0.23	12.16<br>0.23	

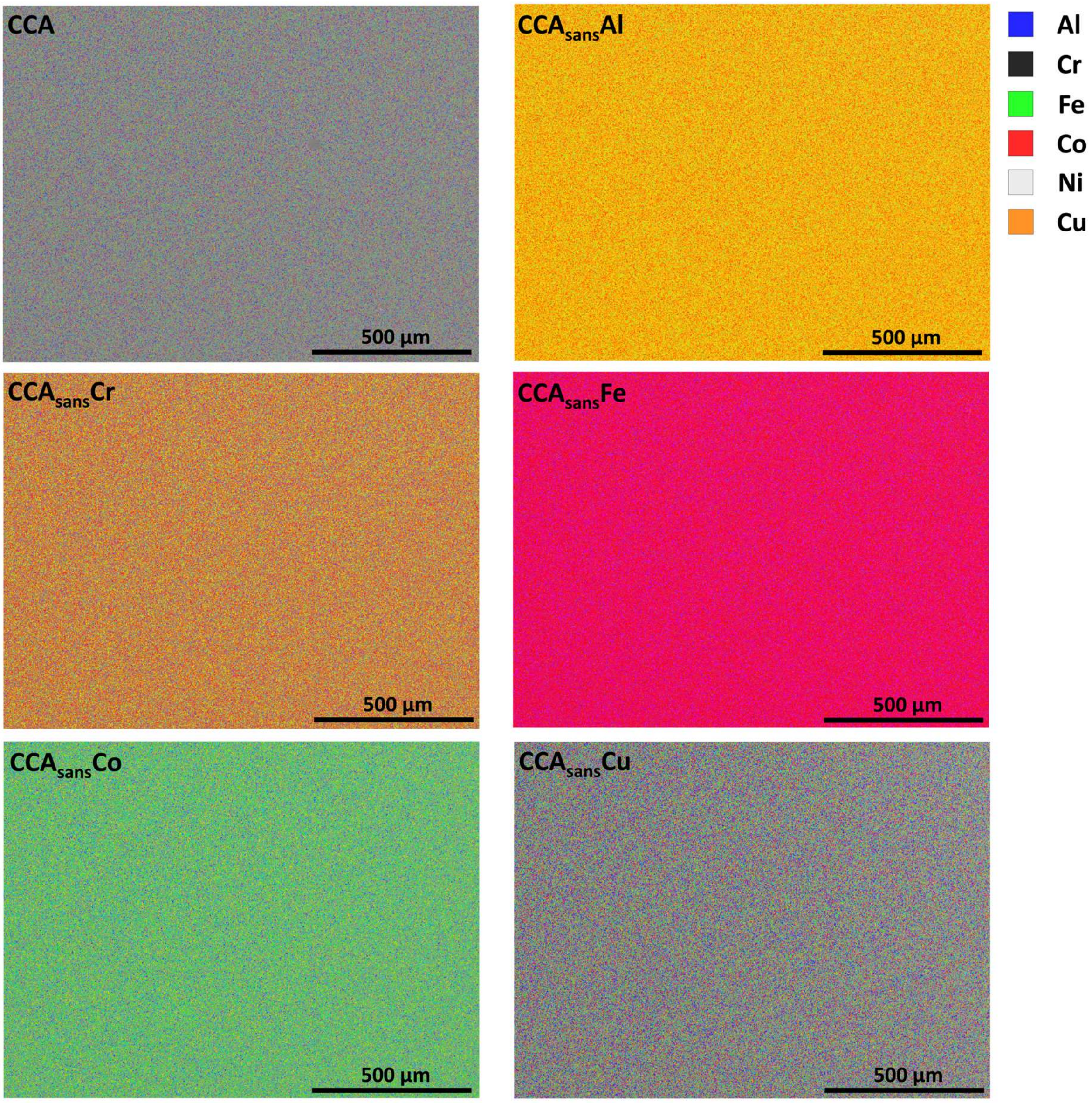

Figure S2. EDS maps formed by layering of each element emission map superimposed on the BSE image for all studied specimens.

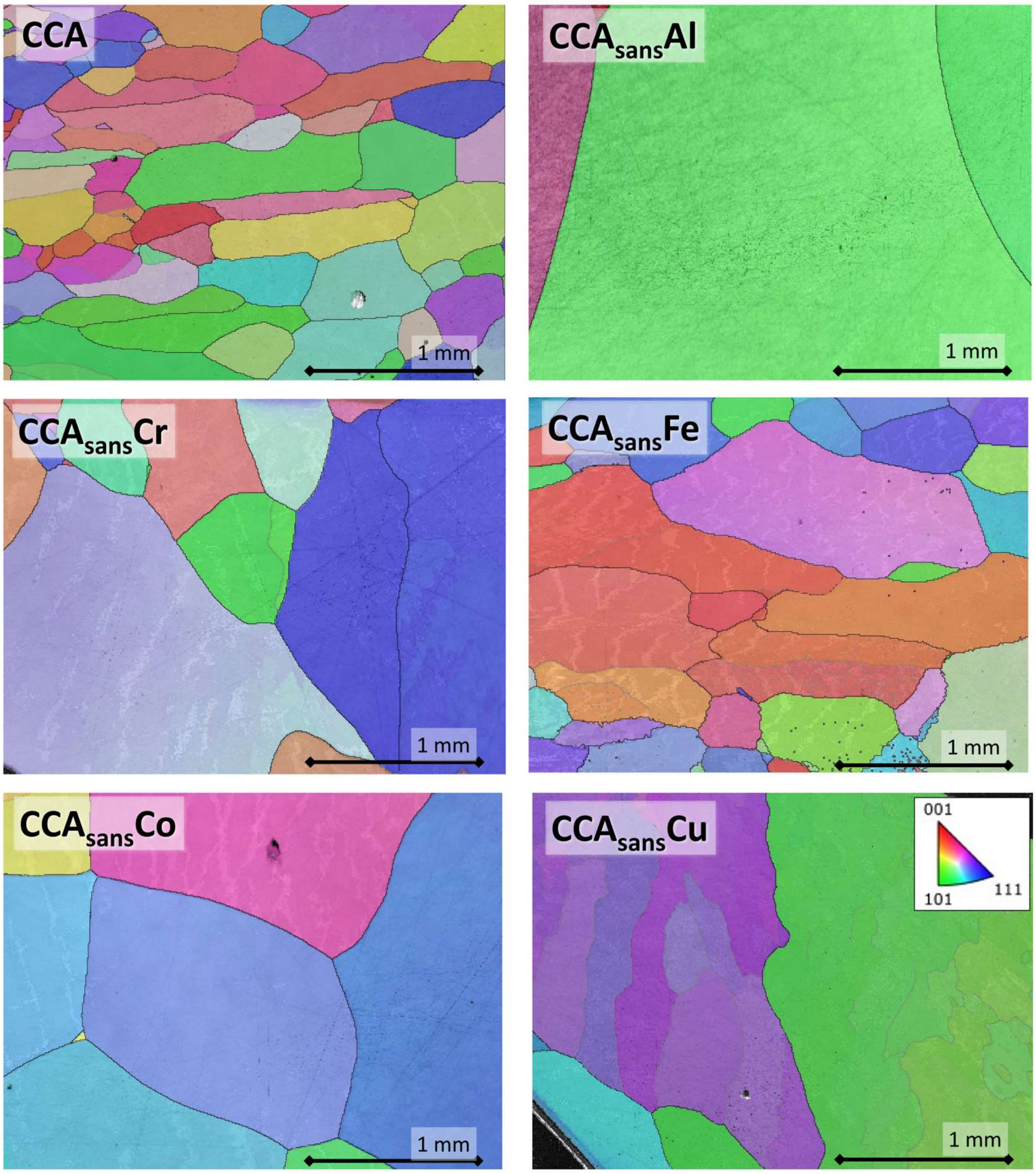

Figure S3. Overlapped normal direction EBSD inverse pole figure and grain boundaries maps of the investigated specimens. The colour code representing crystal orientation is given in the lower right frame.

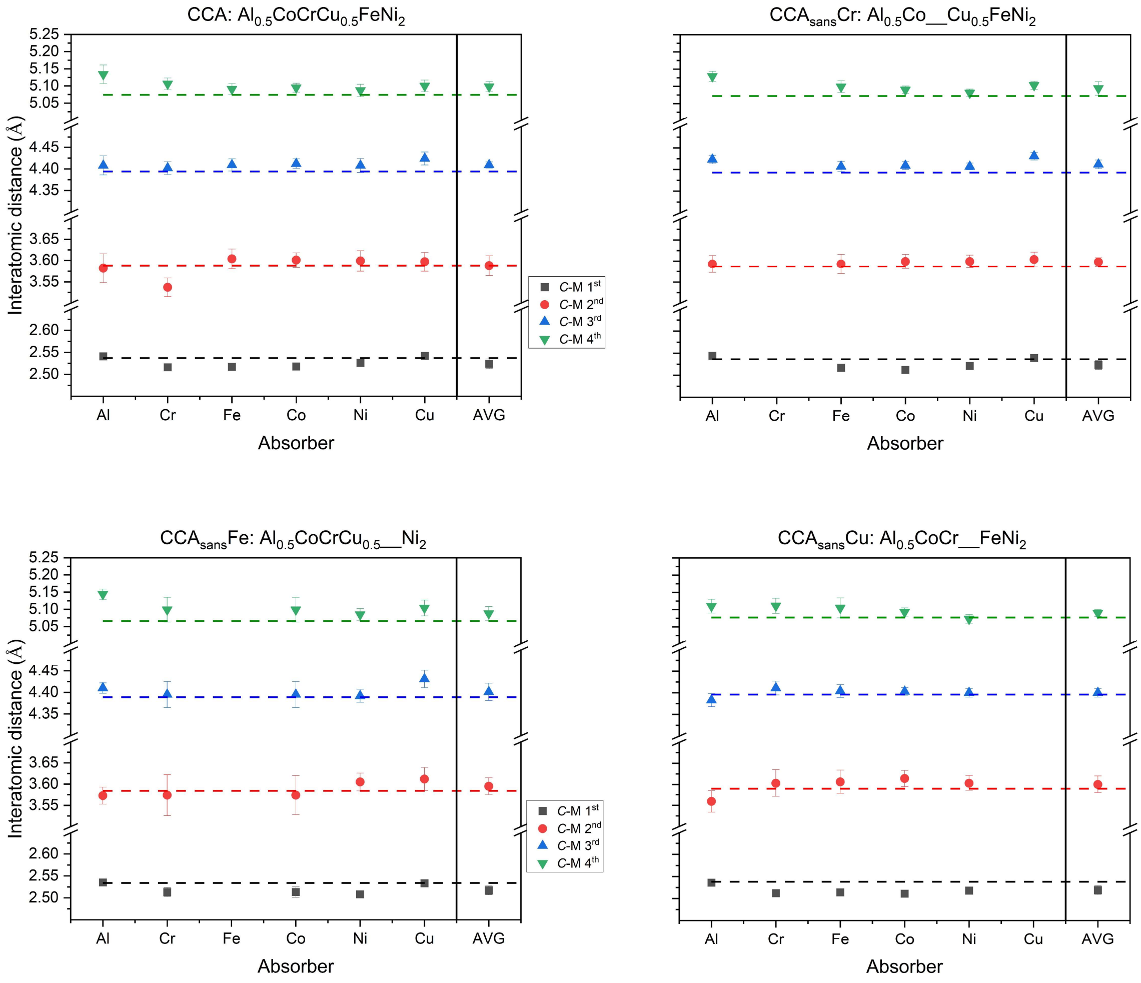

Figure S4. 1st to 4th shell refined distances in CCA$_{sans}$X (X: Ø, Cr, Fe, Cu) from XAS data, as a function of the absorber, or central atom C, increasing Z towards the right), together with the corresponding distances obtained by averaging all the XAS bond lengths according to the experimentally determined composition (AVG). Dashed lines are added representing the XRD 1st to 4th shell distances for comparison.

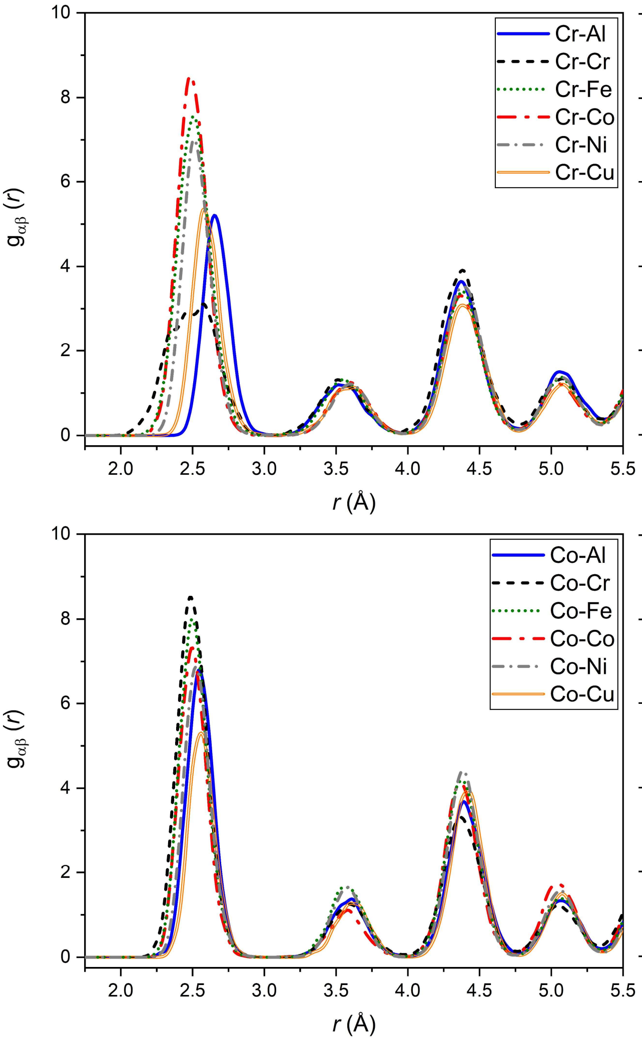

Figure S5. Simulated X-Y (X: Co, Cr, Fe, Ni; Y: Al, Cr, Fe, Co, Ni, Cu) pair correlation functions as a function of the distance r (Å) in the mother compound CCA ($Al_8Cr_{17}Co_{17}Cu_8Fe_{17}Ni_{33}$) in one of the eleven runs performed. Average values of all runs and standard deviations of $g_{\alpha\beta}$ and r are reported in the followingTable S2.

Table S2. Atomic pairs, distance at which the pair correlation intensity $g_{\alpha\beta}$ is maximum and the corresponding intensity maximum value in CCA ($Al_8Co_{17}Cr_{17}Cu_8Fe_{17}Ni_{33}$), ordered from top to bottom in alphabetical order. Data presented are an average of the eleven runs performed (brackets: calculated standard deviations).

**Pairs**	**r [Å]**	**$g_{\alpha\beta}(r)$**
**Al-Al**	2.69(2)	3.7(8)
**Al-Cr**	2.65(1)	6.9(5)
**Al-Co**	2.55(3)	5.3(3)
**Al-Cu**	2.55(2)	11(2)
**Al-Fe**	2.58(1)	6.2(6)
**Al-Ni**	2.51(0)	9.8(5)
**Cr-Co**	2.49(1)	8.6(4)
**Cr-Cr**	2.54(5)	3.3(5)
**Cr-Cu**	2.59(3)	6(1)
**Cr-Fe**	2.50(1)	7.6(2)
**Cr-Ni**	2.52(1)	7.1(3)
**Co-Co**	2.49(1)	7.5(7)
**Co-Cu**	2.55(2)	5.3(7)
**Co-Fe**	2.50(1)	8.0(6)
**Co-Ni**	2.52(1)	6.9(3)
**Cu-Cu**	2.52(2)	14(3)
**Cu-Fe**	2.56(1)	5(1)
**Cu-Ni**	2.53(1)	8.9(5)
**Fe-Fe**	2.53(1)	8(1)
**Fe-Ni**	2.52(0)	6.9(5)
**Ni-Ni**	2.52(1)	7.8(5)

Table S3. Warren-Cowley parameters in the CCA ($Al_8Co_{17}Cr_{17}Cu_8Fe_{17}Ni_{33}$) at T=1523 K.

	**Al**	**Co**	**Cr**	**Cu**	**Fe**	**Ni**
**Al**	0.56	0.07	0.24	-0.44	0.18	-0.28
**Co**		0.03	-0.26	0.29	-0.10	0.08
**Cr**			0.23	0.23	-0.23	0.01
**Cu**				-0.69	0.35	-0.16
**Fe**					-0.07	0.06
**Ni**						0.03

Table S4. Species swap acceptance rates in the CCA ($Al_8Co_{17}Cr_{17}Cu_8Fe_{17}Ni_{33}$). Attempts' number is labelled in brackets after the slash.

	**Al**	**Co**	**Cr**	**Cu**	**Fe**	**Ni**
**Al**	-	0.09 / (33)	0.03 / (29)	0.38 / (34)	0.10 / (42)	0.05 / (44)
**Co**		-	0.40 / (35)	0.16 / (31)	0.47 / (32)	0.61 / (36)
**Cr**			-	0.03 / (33)	0.08 / (39)	0.09 / (46)
**Cu**				-	0.07 / (29)	0.42 / (53)
**Fe**					-	0.22 / (27)
**Ni**						-

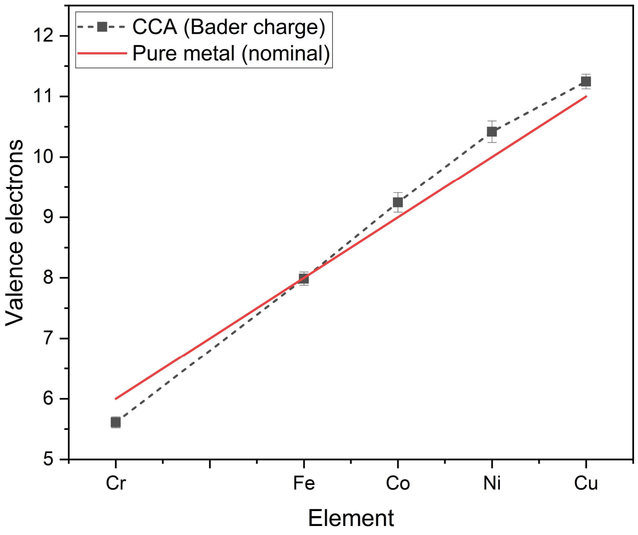

Figure S6. Bader charge analysis with 3d and 4s orbitals as valence electrons for 3d alloying elements in CCA ($Al_8Co_{17}Cr_{17}Cu_8Fe_{17}Ni_{33}$, dashed line with markers), compared to nominal values of pure elements.

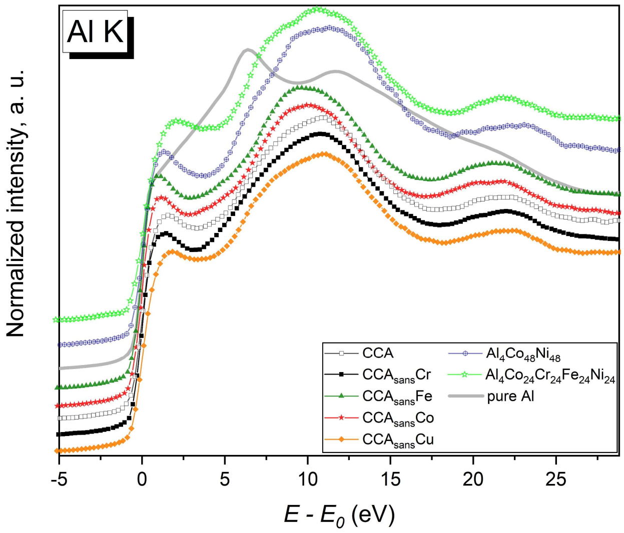

Figure S7. XANES region measured at Al K-edge for all $CCA_{sans}X$ (X = Ø, Cr, Fe, Co, Cu), pure Al and additional alloys: $Al_4Co_{48}Ni_{48}$, $Al_4Co_{24}Cr_{24}Fe_{24}Ni_{24}$. The energy scale is adjusted by the inflection point E_0 of the edge onset.

Table S5. Calculated enthalpy of formation in meV/atom (extracted from ref. [2] and Supporting Information therein) of the lowest energy structures of binary compounds relative to phase separation into pure elements

	**Al**	**Cr**	**Fe**	**Co**	**Ni**	**Cu**
**Al**	0	-138	-369	-629	-677	-224
**Cr**		0	-8	5	-30	108
**Fe**			0	-60	-97	65
**Co**				0	-21	54
**Ni**					0	-6
**Cu**						0

References

1. Daoud, H.M., et al., *Microstructure and Tensile Behavior of Al8Co17Cr17Cu8Fe17Ni33 (at.%) High-Entropy Alloy.* Jom, 2013. **65**(12): p. 1805-1814.
2. Troparevsky, M.C., et al., *Criteria for Predicting the Formation of Single-Phase High-Entropy Alloys.* Physical Review X, 2015. **5**(1).
